# Rotamer-Controlled Self-Immolative Linkers Enable Tunable Release of Neurosteroid Oxime Prodrugs

**DOI:** 10.1021/acsmedchemlett.5c00452

**Published:** 2025-09-25

**Authors:** Aletta E. van der Westhuyzen, Luke E. Hodson, Gouthami Pashikanti, Russell Fritzemeier, Sean B. Yeung, Andrea Mancia, Deston R. Lian, Paul Joseph Tholath, Alejandro Cubillos Paez, Lahu N. Chavan, Michael D’Erasmo, Yanli Yang, Ken Liu, Dennis C. Liotta

**Affiliations:** Department of Chemistry, 1371 Emory University, 1515 Dickey Drive, Atlanta, Georgia 30322, United States

**Keywords:** drug delivery, prodrugs, self-immolative spacers, neurosteroids, traumatic brain injury

## Abstract

In this work we present a modular prodrug strategy for the controlled release of neurosteroid oximes, exemplified through new synthetic routes for both progesterone and allopregnanolone derivatives. By systematically modifying the self-immolative linker with steric, electronic, heterocyclic, and angle-strained elements, we achieved control over *syn/anti*-rotamer populations and self-immolation kinetics. *In vitro* studies across different pH values, temperatures, and biological media revealed a broad range of release rates, while *in silico* modeling corroborated that *syn/anti*-conformer energy differences and p*K*_*a*_ values are key predictors of reactivity. Notably, heterocyclic and electronic designs maintained consistent behavior in both PBS and human plasma, whereas sterically hindered derivatives showed plasma-specific stabilization of the *anti*-conformer. Comparative studies in mouse and rat plasma revealed minimal interspecies differences. These findings clarify critical factors governing self-immolative prodrug behavior in complex environments and offer a framework for the rational design of next generation neurosteroid prodrugs.

## Graphical abstract

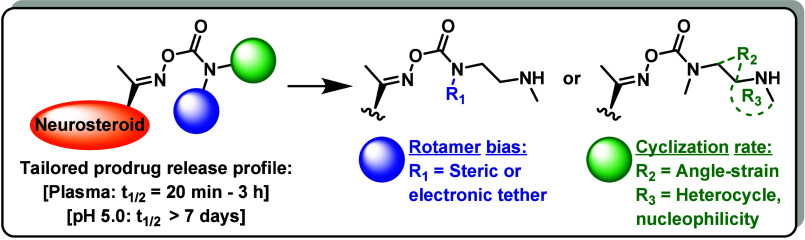

Although neurosteroid research spans decades, the field has experienced a significant resurgence in recent years,^1^ largely driven by the increasing prevalence of central nervous system (CNS) disorders.^2^ Neuroactive steroid (NAS) agents are being investigated as potential treatments for numerous neuropsychiatric and neurodegenerative conditions, including depression,^3^ Alzheimer’s disease,^4^ Parkinson’s disease,^5^ neuropathic pain,^6^ stroke,^7^ and traumatic brain injury.^8^ Their pleiotropic mechanisms – including modulating neuronal excitability, regulation of signaling pathways and gene transcription, and interaction with specific membrane receptors^9^ – has facilitated the advancement of several promising candidates into the clinical landscape.^10^ This includes Zuranolone,^11^ for the treatment of severe postpartum depression, and Ganaxolone^12^ for the treatment of cyclin-dependent kinase-like 5 (CDKL5) deficiency disorder. These breakthroughs are encouraging for the ongoing growth of this vibrant research field.

Among the NAS family, progesterone (**1**, Figure 1) and its active metabolite, allopregnanolone (**2**, Figure 1), have been widely studied in the CNS and periphery.^13^ Their neuroprotective, neuroregenerative and anti-inflammatory roles have been extensively documented in preclinical studies.^14^ Since the fortuitous discovery of progesterone’s activity in traumatic brain injury (TBI) models,^15^ substantial experimental evidence has supported its therapeutic potential in treating acute neuroinjury, for which no FDA-approved treatment currently exists.^16^ This has been attributed to progesterone’s ability to reduce edema, mitigate apoptosis, improve blood-brain barrier (BBB) integrity, and downregulate the inflammatory cascade.^17^ Despite promising efficacy,^18^ the development of **1** culminated in Phase III clinical trial failure^19^ due to several challenging factors including solubility, treatment window, clinical trial design and the complexity of TBI pathophysiology.^20^ Most notably, the hydrophobicity of the steroid resulted in practical limitations for formulating and administering **1**, especially for emergency use. Early prodrug strategies aimed at addressing this solubility impediment led to the discovery of a synthetically accessible C-20 oxime derivative, **EIDD-036** (renamed **NTS-105**, **3**, Figure 1).^21^ Remarkably, **3** retained comparable neuroprotection without converting to **1** *in vivo*.^22^ The oxime group also served as a synthetic handle, which enabled the attachment of solubilizing appendages and exploration of prodrug strategies (**4–6**, Figure 1). Although the introduction of amino acids via an ester-linkage (**4**) moderately improved solubility and released **3** *in vivo*, these prodrugs were susceptible to rapid hydrolysis and degradation in aqueous media.^21^ The critical window of medical intervention for brain injuries, including TBI and acute ischemic stroke, necessitates a field-ready agent for on-site administration.^23^ Accordingly, the phosphorousoxymethylene (POM) conjugate **EIDD-1723** (renamed **NTS-104**, **5**, Figure 1) was developed, exhibiting improved hydrolytic stability, high aqueous solubility and strong preclinical performance, warranting its progression to Phase I clinical trials.^24^

**Figure 1:**
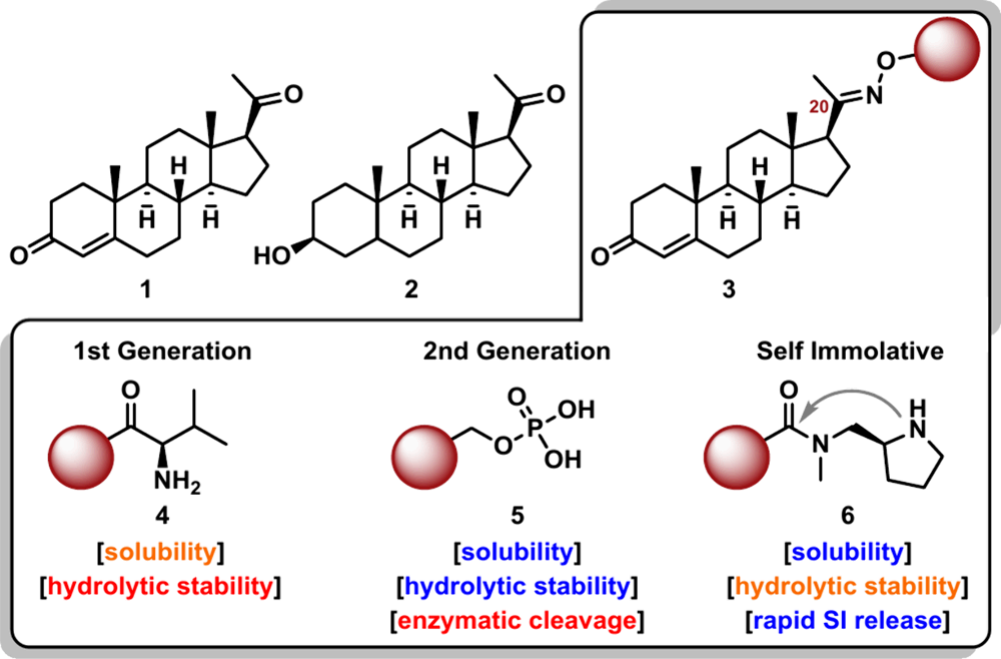
Neuroactive steroids progesterone (**1**) and its main metabolite allopregnanolone (**2**). An equipotent oxime-derivative of progesterone (**3**), with water-soluble prodrugs incorporating an amino acid (**4**), POM phosphate (**5**) and amine carbamate SI pyrrolidine promoiety (**6**).

Given the substrate-specific enzymatic activation of phosphate prodrug **5**, achieving optimal conversion proved challenging, prompting the exploration of a strategy less dependent on enzymatic activation to enhance exposure to **3**. Inspired by the work of Dal Corso et al.,^25^ our recent development of a pH-responsive prodrug incorporating an amine-based self-immolative (SI) promoiety (**6**, Figure 1) allowed for rapid release of the oxime payload **3** via an SI cyclization reaction, which was further validated in an *in vivo* efficacy model of TBI.^26^ The use of SI linkers as practical water-solubilizing promoieties presents a promising strategy for the delivery of carbamate-oxime bearing payloads.

In our previous work, although **6** demonstrated an attractive release profile, its hydrolytic stability could be improved for a field-ready formulation (Figure 2A). In contrast, prodrug **7** (Figure 2A) exhibited desirable storage stability, yet its slow *in vitro* release kinetics rendered it unsuitable for acute TBI applications. A valuable aspect of the SI construct is the potential to control and tune the rate of disassembly depending on the application requirements,^27^ with several parameters and structural considerations affecting the kinetics of the reaction. By design, control of the solution pH dictates whether the prodrug remains stabilized as protonated **8** (mildly acidic storage media, Figure 2A, blue) or converts to its active form **9** at physiological pH. This activation is governed by the acid dissociation constant (p*K*_*a*_) of **8**, which can be modulated through incorporation of electron-withdrawing or -donating groups. Beyond temperature and the reactive site chemistry, the rate of SI intramolecular cyclization is primarily governed by conformational factors, including the Thorpe-Ingold effect and reactive rotamer populations.^27^ Considering the intrinsic pseudo double bond character of the carbamate motif, the potential deconjugation of the system gives rise to coexisting *syn*- and *anti*-isomers (Figure 2A).^28^ The *anti*-rotamer is generally the energetically preferred conformation for steric and electrostatic reasons, but the orientation does not support SI cyclization (Figure 2A, red). However, amide *N*-substituents can perturb the *syn*/*anti* equilibrium and influence reactivity.^29^ Similarly, pH-dependent conformational switching and rotamer preference has been shown for amide bonds.^30^ Recently, Dal Corso and co-workers introduced an advanced pyrrolidine-carbamate SI spacer incorporating a tertiary amine handle to accelerate the cyclative cleavage of OH-bearing drugs *in vitro*.^31^ This modification altered the *syn*-*anti* carbamate rotamer equilibrium via intramolecular H-bonding and nucleophile p*K*_*a*_ perturbation, with *in silico* 3D analysis indicating that nucleophilic attack along the Bürgi-Dunitz trajectory favored the *syn*-rotamer. Guided by this theory and aiming to combine the rapid release rate of **6** with the hydrolytic stability of **7**, we developed derivatized pH-responsive amine-carbamate SI linkers with adjustable cyclization rates to create prodrugs with tailored release profiles (Figure 2B).

**Figure 2:**
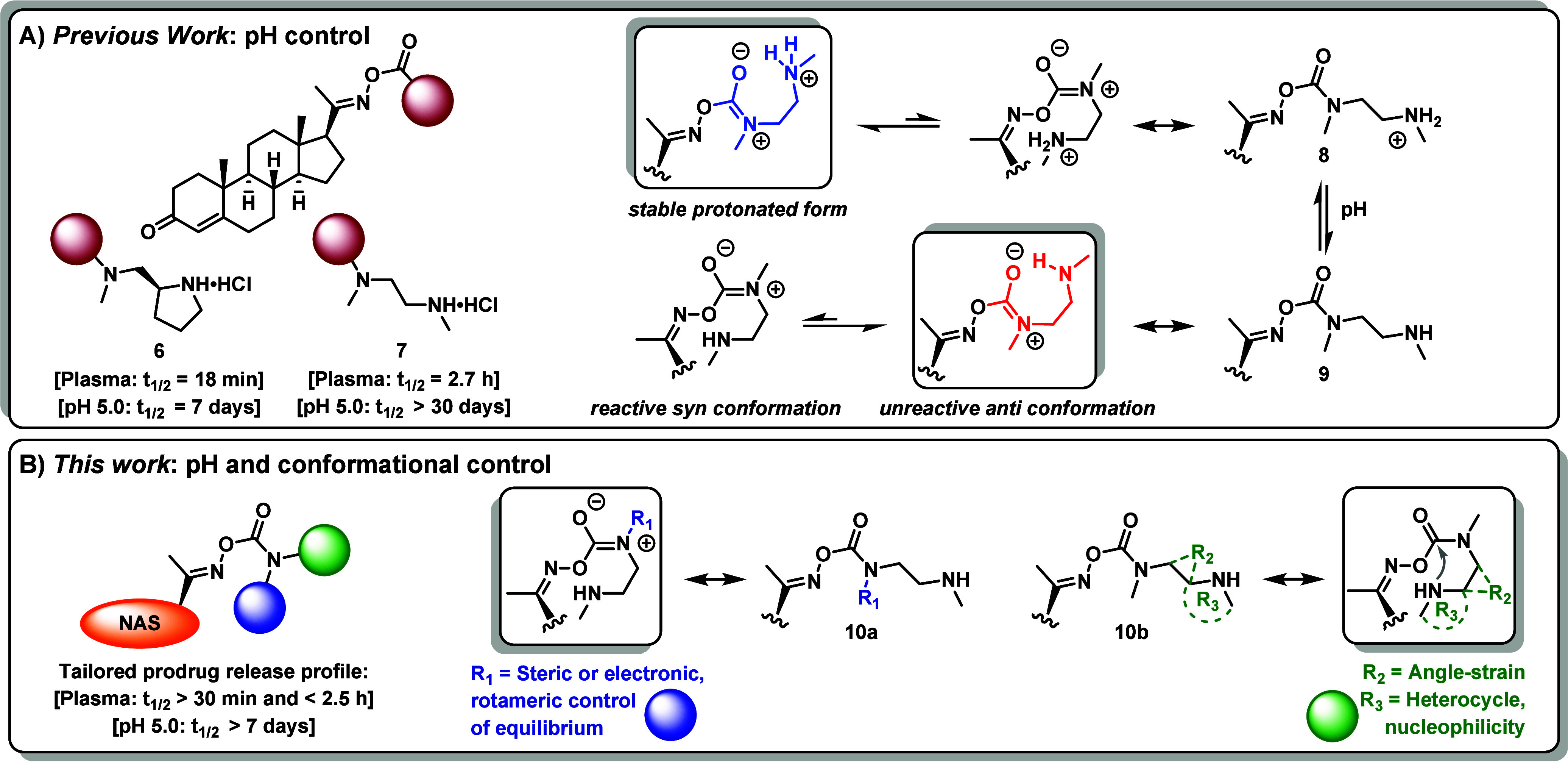
A) Structural representation of progesterone-oxime amine carbamate SI prodrugs comprising the fast-cyclizing pyrrolidine (**6**) and ethylene diamine SI construct (**7**). Schematic illustration of the oxime carbamate dimethyl ethylene diamine promoiety in its stable protonated form **8** vs the neutral species **9**, and the corresponding conformations that support the unreactive *anti*-rotamer and the (less favored) *syn*-rotamer capable of cyclization. B) Incorporating structural elements (**10a**–**b**) to alter the population of the reactive rotamer, amine nucleophilicity or angle strain through Thorpe-Ingold effects, thereby controlling the SI rate.

To achieve this, we proposed incorporating structural elements (**10a**–**b**, Figure 2B) designed to modulate the reactive rotamer population or overall reactivity, thereby controlling the SI cleavage rate. First, carbamate *N*-derivatization with groups leveraging steric or electronic interactions with the oxime-carbamate backbone (Figure 2B, dark blue) could shift the rotamer equilibrium toward the reactive *syn*-conformer. Steric bulk at R_1_ increases clash with the oxime, destabilizing the *anti*-conformer while in the electronic series, the *syn*-rotamer may be stabilized via H-bonding between a protonated tertiary amine tether and the carbonyl oxygen. Second, modulation of angle strain and amine nucleophilicity through linker and heterocyclic derivatization (Figure 2B, green) allowed fine-tuning of the intramolecular ring closure reaction, thereby controlling self-immolation. This strategy was applied to both progesterone and allopregnanolone oximes (Figure 2B, orange) highlighting its potential as a versatile NAS prodrug platform. Thus, whether rapid drug release is required at the time of injury or a slower, sustained delivery during neurorehabilitation to address long-term neurological sequelae and progressive neurodegeneration following TBI,^32^ the SI design can be synthetically tailored to meet specific therapeutic needs. Herein, we report the design and synthesis of a repertoire of SI constructs enabling either rapid or sustained release of oxime-bearing active species. Extended storage stability and *in vitro* degradation were assessed at various pH and temperatures in PBS and human plasma using an optimized pH-sensitive bioanalytical protocol. Differences in SI cleavage rates and reactivity were rationalized through *in silico* conformational analysis and p*K*_*a*_ calculations. Finally, interspecies plasma stability was evaluated for select prodrugs to identify suitable *in vivo* animal models for future pharmacokinetic and efficacy studies.

## Results and Discussion

### Chemistry

Building on our previous efforts, the amine-carbamate progesterone oxime SI targets were synthesized without significant challenges (Scheme 1).^26^ Starting with the commercially available pregnenolone (**11**, Scheme 1 **A**), the 4-nitrophenylcarbonate progesterone oxime **13** was synthesized through a sequence of established reactions, including condensation with hydroxylamine hydrochloride, Oppenauer oxidation, and coupling with 4-nitrophenyl chloroformate to yield the activated intermediate.^22^ The presence of the C3 hydroxyl group on the allopregnanolone scaffold (**12**, Scheme 1 **A**), capable of acting as a competing nucleophile, necessitated preliminary installation of a TBDMS protecting group. This was followed by oxime formation and subsequent acylation to afford the activated electrophile **14**. With these intermediates in hand, we shifted our synthetic efforts toward constructing a library of derivatized amine linkers (**17a–z**, Scheme 1B–C) designed for SI cyclization upon incorporation as the oxime-carbamate. For linkers featuring steric and electronic *N*-amine elements (Scheme 1B, blue), an efficient reductive amination was employed. Reaction of *tert*-butyl methyl­(2-oxoethyl)­carbamate (**15**) with amines **16b**–**k** in methanol, followed by imine hydrogenation using H_2_ and Pd/C, yielded linkers **17b–j** after filtration with no further purification required (**17a** commercially available). Linkers **17l–v** were designed to introduce heterocyclic modifications or induce angle strain (Scheme 1C, green). Commercially available amines **16l–t** were converted into linkers **17l–t** using a previously established *N*-methylation sequence.^26^ For **17u** and **17v**, an optimized three-step synthesis via Weinreb amide intermediate and reductive amination, respectively, were employed (details in Supporting Information). Lastly, linkers **17w–z** were selected to examine the influence of amine nucleophilicity on the rate of self-immolation (Scheme 1D). Penultimate coupling of the activated carbonate of progesterone oxime (**12**) with amine linkers 17 **a-z** and allopregnanolone (**14**) with **17a–d, j, k, l** proceeded smoothly with the addition of TEA in DCM, yielding the respective carbamates **18** and **19**. Finally, Boc and TBDMS deprotection of **18** and **19** was achieved by stirring in TFA/DCM, yielding the desired library of amine-carbamate SI linker prodrugs **20** and **21** as TFA salts.

**Scheme 1:**
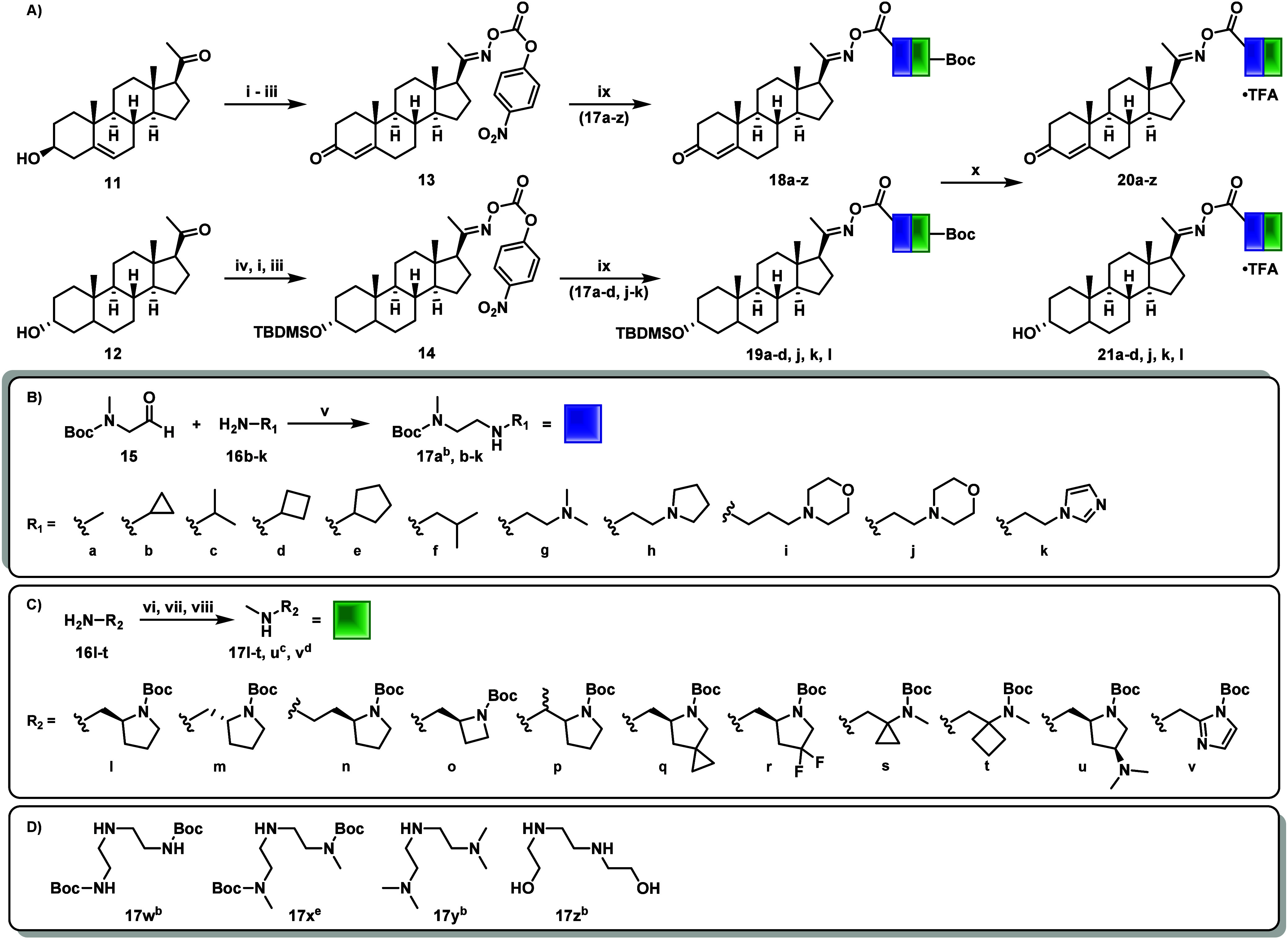
Synthesis of Progesterone and Allopregnanolone Amine-Carbamate SI Prodrugs^b^

a A). Synthetic layout of progesterone and allopregnanolone reactive intermediates, carbamate formation and deprotection reactions. B) Steric (**17a**-**f**) and electronic (**17g**-**k**) linker group synthesis (blue). C) Heterocyclic (**17l**-**v**) and angle strained linker (**17s, t**) group synthesis (green). D) Other linkers utilized toward the amine nucleophile class targets. Reagents and conditions: (i) NH_2_OH·HCl, NaOAc, and MeOH/H_2_O, reflux. (ii) Al­(OiPr)_3_, 1-methyl-4-piperidone, and toluene, reflux. (iii) 4-Nitrophenyl chloroformate, TEA, and DCM, 0 °C. (iv) TBDMS-OTf, DCM, 0 °C – rt. (v) *H*_2_(*g*), Pd/C, MeOH, rt. (vi) Cbz-Cl, K_2_CO_3_, THF, 0 °C – rt. (vii) MeI, NaH, DMF, 0 °C – rt. (viii) *H*_2_(*g*), Pd/C, MeOH, rt. (ix) amine, TEA, DCM, rt. (x) TFA, DCM, 0 °C – rt.
b Commercially available amine linkers.
c Synthesized over three steps from Boc-4-oxo-Pro-OH.
d Synthesized from *tert*-butyl 4-formyl-1*H*-imidazole-1-carboxylate.
e Synthesized over three steps from **17w**.

### *In Vitro* Evaluation of Prodrugs

Given the solubility impediment of progesterone and allopregnanolone, it was essential that the prodrugs exhibit acceptable aqueous solubility. For the solubility assessment, acetate buffer (pH 5) was selected as the media of choice to ensure prodrug stability during analysis. Pleasingly, most prodrugs displayed high aqueous solubility (>10 mg/mL for progesterone and >5 mg/mL for allopregnanolone) based on visual inspection of solid dissolution in media (see Table S23 in Supporting Information). Prodrug stability was first investigated at room temperature in acetate buffer and PBS using HPLC analysis. Here, the half-life (*t*_1/2_) was calculated from the percentage of intact carbamate over time based on UV detection. Unfortunately, the absence of UV activity excluded the allopregnanolone-based prodrugs from HPLC analysis. The corresponding results for select prodrugs are summarized in Table 1 (see Table S23 in Supporting Information for complete data set). Stability in acetate buffer serves as a practical indicator for prospective drug storage and highlights a key advantage of the pH-responsive prodrug strategy. Gratifyingly, most of the newly synthesized progesterone prodrugs, except for **20g**, **20h**, **20j** and **20u**, demonstrated stability exceeding 7 days at pH 5.0. As anticipated, incubation in PBS significantly reduced the parent prodrug half-lives, revealing distinct cyclization trends. This was particularly evident in the steric series (**20a–e**), where increasing steric bulk at the *N*-carbamate position led to progressively faster degradation. Notably, the half-life decreased from 46.9 h for **20a** (methyl) to 10.7 h for **20e** (cyclopentyl), supporting the hypothesis that greater steric demand favors the reactive *syn*-rotamer, thereby enhancing SI cyclization. Three representatives from the electronic series, **20g**, **20h** and **20j**, exhibited progressively slower release rates, with differences in half-life of 2.17, 7.23, and 21.77 h; respectively.

**Table 1:**
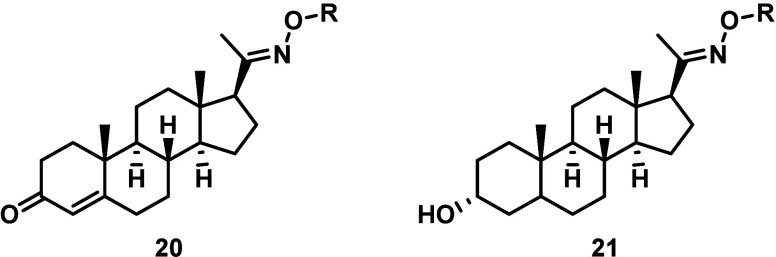

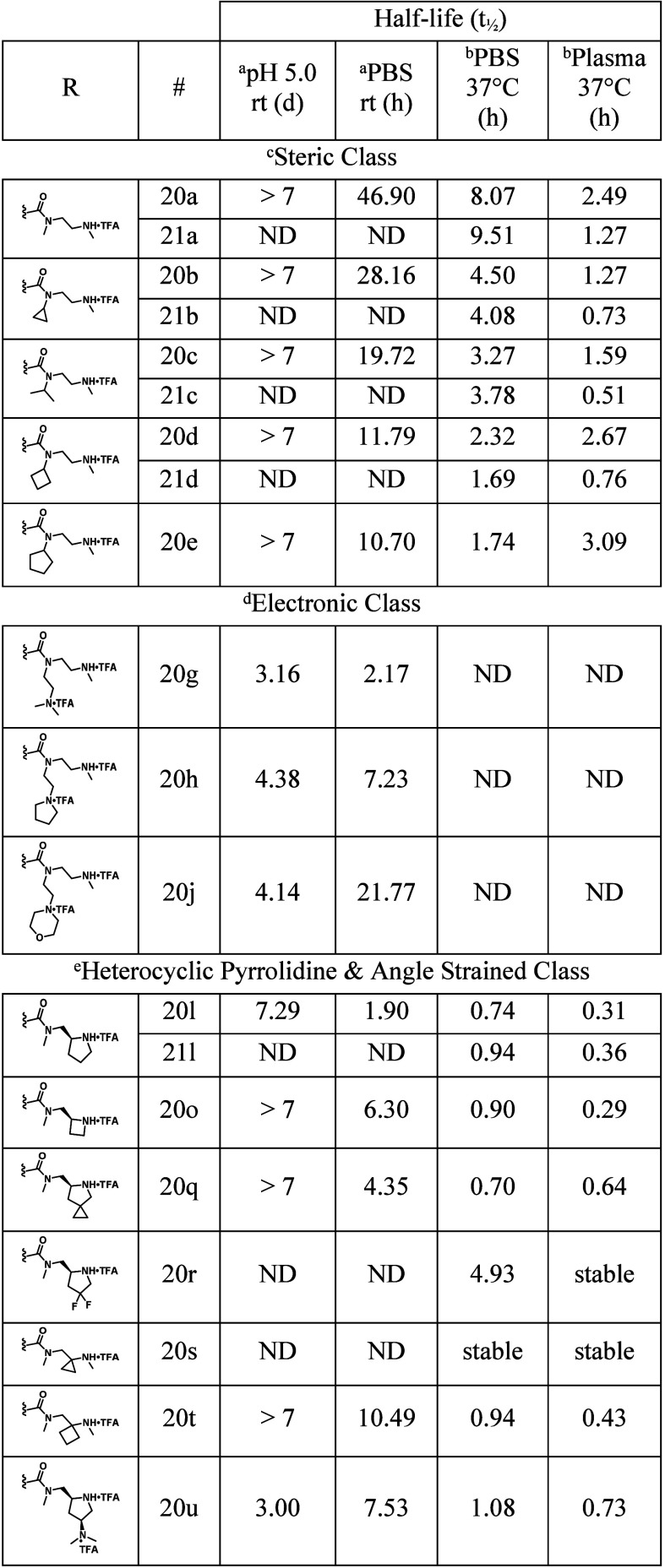
Prodrug Stability in Acetate, PBS and Plasma

a Analysis by HPLC (UV).

b Analysis by LC-MS/MS (ionization). Complete data set includes compounds.

c **20f**.

d **20i**, **20k**, **20w**–**z**.

e **20m**, **20n**, **20p**, **20v**.

These differences are attributed to decreasing amine p*K*_*a*_, and the ability of each prodrug to effectively enforce the reactive *syn*-confirmation via intramolecular H-bonding between the tertiary amine and sp^2^-hybridized oxime oxygen. To further modulate release kinetics, we focused on heterocyclic pyrrolidine and angle-strained derivatives to generate prodrugs with slower release rates than the fast-cleaving pyrrolidine **20l** (t_1_/_2_ = 1.90 h). Encouragingly, this series of prodrugs exhibited a range of intermediate cleavage profiles relative to previously characterized analogues, effectively bridging the gap between rapid- and slow-release rates. Substituting pyrrolidine with azetidine in **20o** increased the half-life to 6.30 h. Similarly, incorporation of a sterically hindered cyclopropane ring to the pyrrolidine scaffold in **20q** slowed the SI rate (*t*_1/2_ = 4.35 h). Borrowing from Dal Corso,^31a^ **20u** was synthesized to evaluate the effect of the basic amine handle on the cyclization rate. Although it was hypothesized that the tertiary amine would enhance reactivity by lowering the secondary nitrogen p*K*_*a*_, thereby increasing nucleophilicity, the observed cyclization kinetics were slower (*t*_1/2_ = 7.53 h). As anticipated, installation of two electronegative fluorine atoms in **20r** greatly reduced nucleophilicity, resulting in a highly stable, noncleaving prodrug which was not further pursued. The Thorpe-Ingold effect on intramolecular cyclization was also investigated by incorporating cyclopropane (**20s**) and cyclobutane (**20t**) rings at the β-carbon of the traditional dimethylethylenediamine linker. Remarkably, the small difference in ring strain had a pronounced impact on self-immolation: complete inhibition of cyclization was observed for **20s**, whereas the angle induced by the cyclobutane in **20t** facilitated cleavage (*t*_1/2_ = 10.49 h). The contribution of the α-methyl Thorpe-Ingold effect in **20p** was too severe, resulting in an unstable compound. While the observed stability profiles in PBS aligned with our theoretical framework, it is important to note that PBS does not fully mimic physiological conditions. To address this, prodrug stability was further evaluated in human plasma at 37 °C using liquid chromatography–tandem mass spectrometry (LC-MS/MS). For direct comparison, a parallel screen was conducted in PBS under identical conditions (Table 1). To prevent premature degradation prior to LC-MS/MS analysis, acetonitrile containing formic acid was used as a quenching solution to acidify and stabilize the prodrugs in vials. As expected, the degradation trends observed in PBS were consistent across analytical methods (HPLC vs LC-MS/MS) and conditions. Elevated physiological temperature substantially increased self-immolation rate, attributable to a lowered energetic barrier for accessing the reactive *syn*-rotamer, which facilitates cyclization and product release. However, a key observation was the marked difference in the SI kinetics between plasma and PBS, suggesting that enzymatic activity in plasma contributes to prodrug cleavage beyond what is observed in buffer alone. Although quantifying the percentage prodrug remaining and the corresponding half-lives (Table 1) provided a useful metric, certain prodrugs (including **20g**, **20h**, **20j**, **20w**, and **20x**) could not be detected via their precursor ions. This limitation in detection, along with the need for a more uniform and broadly applicable assessment across all compounds, prompted us to monitor the formation of the parent oxime as an alternative readout.

Given the greater biological relevance of plasma, we focused on quantifying the formation of progesterone and allopregnanolone oxime in human plasma at 37 °C over a 2 h period. Results for selected progesterone prodrugs are presented in Figure 3A–C, illustrating the broad range of plasma cleavage rates as a function of promoiety design (see Figure S13 in Supporting Information for complete data set). Complete stability data for synthesized allopregnanolone prodrugs (Figures S18–S20) are provided in the Supporting Information. Given that their trends closely paralleled the progesterone series, they were not discussed in detail in the main text. For visual clarity, prodrugs are grouped by class, with **20a** (PBS: *t*_1/2_ = 8.07 h; plasma: *t*_1/2_ = 2.49 h) and **20l** (PBS: *t*_1/2_ = 0.74 h; plasma: *t*_1/2_ = 0.31 h) included as slow- and fast-releasing controls, respectively, to enable direct comparison. Curiously, within the steric series (Figure 3A), all compounds except **20b** and **20c** showed reduced oxime formation in plasma compared to **20a**. Moreover, increasing steric bulk at the *N*-carbamate position led to slower self-immolation in plasma – an inverse trend to that observed in PBS. This unexpected result prompted further investigation using computational modeling, described in the following section. Conversely, the electronic series (Figure 3B) displayed similar, though slightly accelerated, cleavage in plasma relative to PBS. Notably, *in silico* calculated p*K*_*a*_ values of the tertiary amine tether (see Table S21 of Supporting Information) correlated with release rate. Higher p*K*_*a*_ values were associated with faster and more complete oxime formation, likely due to enhanced H-bonding which could stabilize the reactive *syn*-conformer. This effect was most pronounced for the dimethylamine (**20g**, p*K*_*a*_ = 8.19) and pyrrolidine (**20h**, p*K*_*a*_ = 8.45) derivatives, both of which showed rapid, first-order release. In contrast, the morpholine (**20i**, p*K*_*a*_ = 6.84; **20j**, p*K*_*a*_ = 6.08) and imidazole (**20k**, p*K*_*a*_ = 6.24) tethered prodrugs exhibited slower and less complete release of **3**, consistent with linear, zero-order kinetics. For the heterocyclic pyrrolidine and angle-strained series (Figure 3C), the release rates closely matched the experimentally determined prodrug half-lives (Table 1), which were shorter in human plasma than in PBS. As expected, the *R*-enantiomer **20m** produced a release profile nearly identical to that of **20l**. Similarly, azetidine **20o** showed comparable half-lives in PBS (*t*_1/2_ = 0.9 h) and plasma (*t*_1/2_ = 0.29 h), with a similar rate of oxime **3** formation in plasma compared to **20l**. Prodrugs **20q** and **20u** displayed modestly longer half-lives in plasma, consistent with the slower formation of oxime **3** observed in Figure 3C. The more stable prodrugs **20n**, **20r**, and **20v** generated low levels of oxime and were omitted from the figure. Although both **20s** and **20t** are capable of adopting the reactive *syn*-rotamer, their self-immolation reactivity diverged due to differences in linker geometry. The cyclopropane in **20s** induces angle strain that hinders nucleophilic attack, rendering the compound stable under both PBS and plasma conditions. In contrast, the cyclobutane geometry of **20t** promotes efficient self-immolation, resulting in a faster plasma half-life (*t*_1/2_ = 0.43 h) than in PBS (*t*_1/2_ = 0.94 h), and similar oxime formation to **20l** (Table 1, Figure 3C). This intriguing result warrants further investigation, with follow-up studies currently in progress. Finally, the nucleophilic capacity of the amine tethers in prodrugs **20w–20z** were evaluated based on their ability to cyclize in plasma, as detailed in the Supporting Information Figure S13.

**Figure 3:**
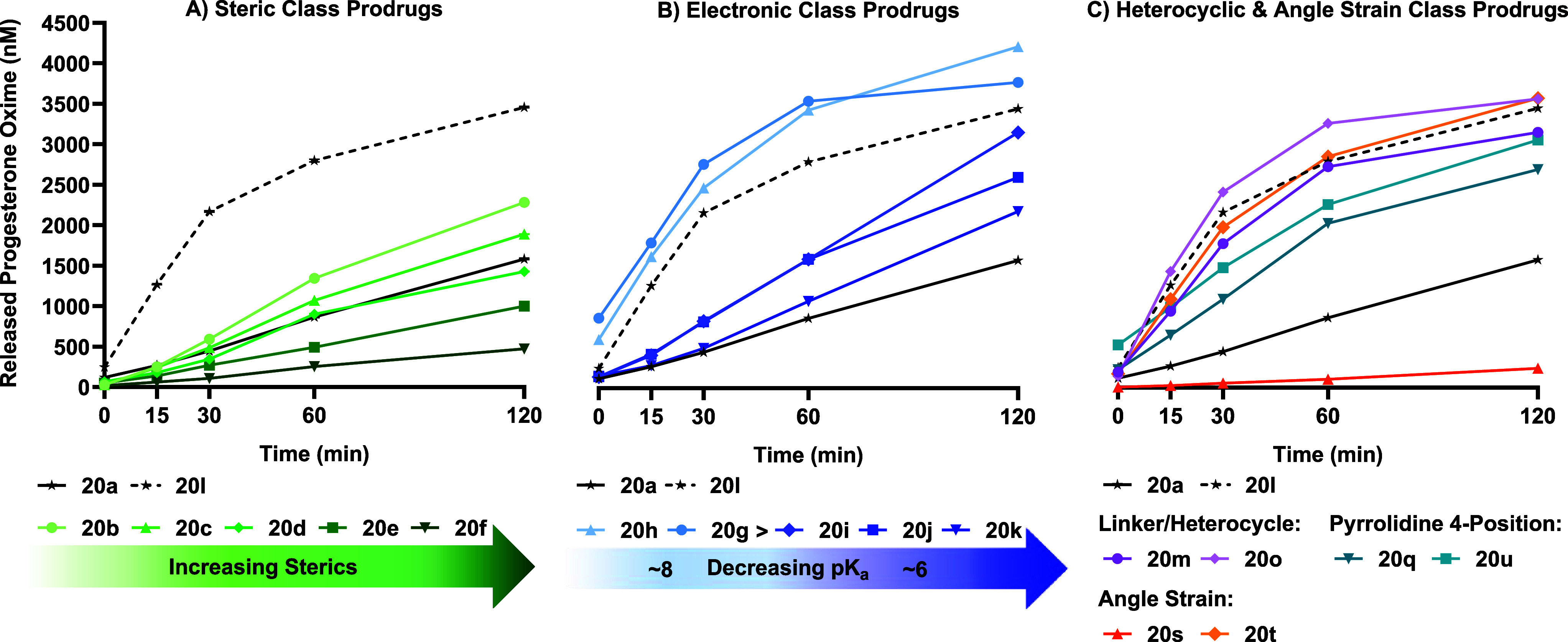
Formation of progesterone oxime **3** from respective prodrugs in human plasma over time. Concentration calculation of **3** is based upon the assumption that all tertiary amines are charged. Data presented as mean ± SD (*n* = 2).

### Computational Studies

While environmental pH acts as a trigger for self-immolation, the carbamate moiety introduces an additional molecular switch by enabling interconversion between *anti* and *syn* conformations. Typically, the *anti*-conformer is favored due to intramolecular H-bonding and minimized steric clash; however, nucleophilic attack and subsequent self-immolation can proceed only through the *syn*-conformer. To bridge chemical theory with the observed *in vitro* self-immolation rates, we performed *in silico* calculations to model the relative abundance of the *syn*-conformer. We focused our computational analysis on the steric series of prodrugs (**20a–f**), which showed notable discrepancies between PBS stability and plasma. For each promoiety, the *anti/syn* equilibrium was evaluated by identifying global minimum energy *anti* and *syn* conformers and calculating their relative energy differences (kcal/mol). All structures were modeled in their protonated acetoxime-abbreviated forms, followed by conformational searching and DFT optimization (B3LYP-D3/6–31G**) with implicit solvation. The resulting energy differences were then correlated with the percentage of prodrug remaining after 2 h of incubation in PBS and plasma at 37 °C, as depicted in Figure 4 (for complete details, see Supporting Information).

**Figure 4:**
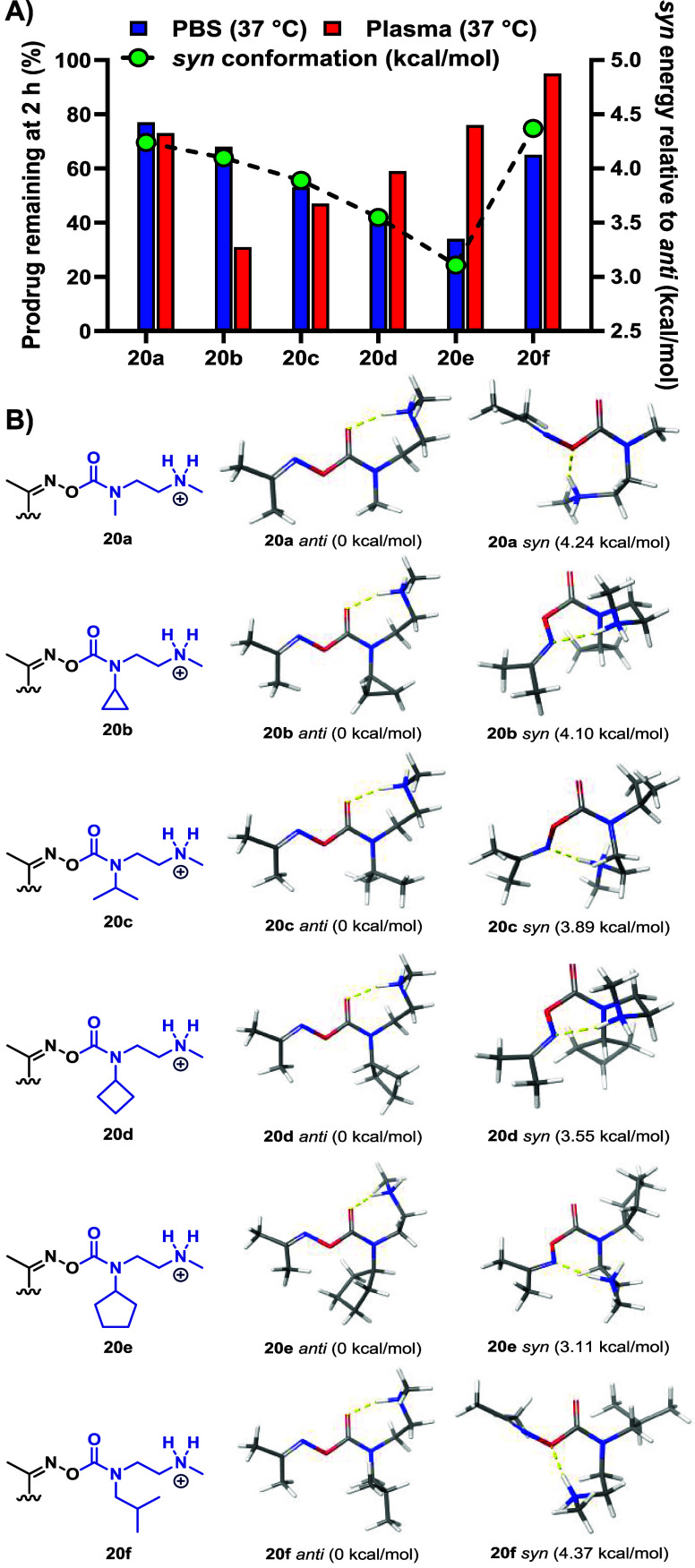
A) Percentage prodrug remaining after 2 h in PBS (blue) and plasma (red) at 37 °C vs calculated *syn* energies (relative to *anti*) (green). B) Optimized global minimum *anti*- and *syn*-conformers (B3LYP-D3/6–31G**) for **20a–f** in their protonated acetoxime-abbreviated forms.

Across the series (Figure 4B), the *anti*-conformation adopts a seven-membered ring via intramolecular H-bonding between the protonated amine and the carbonyl oxygen. In contrast, the *syn*-conformer enables H-bonding between the amine and either the oxime oxygen or nitrogen. Calculations showed that **20a** favors the *anti*-conformer by 4.24 kcal/mol. As steric bulk at the *N*-carbamate increases (**20a–e**), the *anti*/*syn* energy gap narrows, making the *syn*-conformer more accessible and increasing the population of the reactive species. This trend (Figure 4A, green) correlates well with the experimentally observed cleavage rate in PBS (Figure 4A, blue), suggesting that higher *syn*-conformer abundance promotes more efficient self-immolation. Despite its substantial bulk, **20f**’s steric demand is centered at the γ-position, which is distal from the oxime and therefore does not destabilize the *anti*-conformer to the same extent as other members of the series. The calculated conformational energy difference aligns well with experimental PBS data, supporting the predictive utility of this modeling approach under controlled conditions. However, this correlation between conformational equilibrium and self-immolation rate was not observed in plasma. While computational models accurately predict behavior in buffered systems like PBS, they do not account for enzymatic contributions and dynamic complexity in biological media. This limitation is particularly relevant for rotamer-activated systems, where conformational dynamics – not enzymatic cleavage – govern release. Interestingly, the steric series appeared especially susceptible to enzyme-mediated stabilization of the *anti*-conformer in plasma. While **20b** (cyclopropyl) and **20c** (isopropyl) showed improved release compared to **20a**, further increases in bulk – cyclobutyl (**20d**), cyclopentyl (**20e**), and isobutyl (**20f**) – led to slower cleavage. These findings suggest a steric threshold beyond which enzyme interactions override the intrinsic conformational bias, reducing the population of the *syn*-conformer. Conversely, the electronic and heterocyclic/angle-strained series retained their expected cleavage trends in both PBS and plasma, indicating that electronic effects and ring strain may help mitigate or overcome enzymatic stabilization of the *anti*-conformer. This highlights the delicate balance between steric effects and enzymatic modulation in determining prodrug activation in biological media. This also suggests that heterocyclic or angle-strained prodrugs may offer a more robust strategy for achieving predictable *in vivo* activation, whereas sterically hindered analogues will require additional optimization to improve performance in physiological environments.

Building on our observation that rotamer control in the steric series can be modulated by enzymatic stabilization or activation, we next evaluated whether this effect was species dependent. To investigate this, we performed comparative stability studies by incubating select prodrugs (**20a**, **20b**, **20l** and **20t**; Figure 5) in mouse and rat plasma – two of the most commonly used preclinical species in drug development. This approach allowed us to assess the potential influence of species-specific enzymes on rotamer equilibria and, by extension, prodrug activation rates. The self-immolation profiles for the sterically tuned linker series (**20a–b**) reveal discernible, species-dependent differences prodrug stability. Human plasma consistently exhibited the fastest release, with compound **20b** showing the greatest divergence – releasing more rapidly than **20a** in human plasma and markedly faster than in the corresponding murine models. These discrepancies suggest that human plasma may lack certain stabilizing interactions or enzymatic contributions present in murine systems, potentially due to differences in esterase expression or protein binding. While the precise biochemical drivers remain unclear, this observation warrants further investigation beyond the scope of this work. In contrast, compounds **20l** and **20t** demonstrated complete self-immolation across all species within the 2 h window, with only minor variations in first-order kinetic rates. Notably, **20t** stands out as a promising candidate, offering comparable rapid release to **20l** but with superior long-term storage stability. These results underscore the importance of evaluating prodrug performance across species to guide the selection of appropriate preclinical models for efficacy and pharmacokinetic studies.

**Figure 5:**
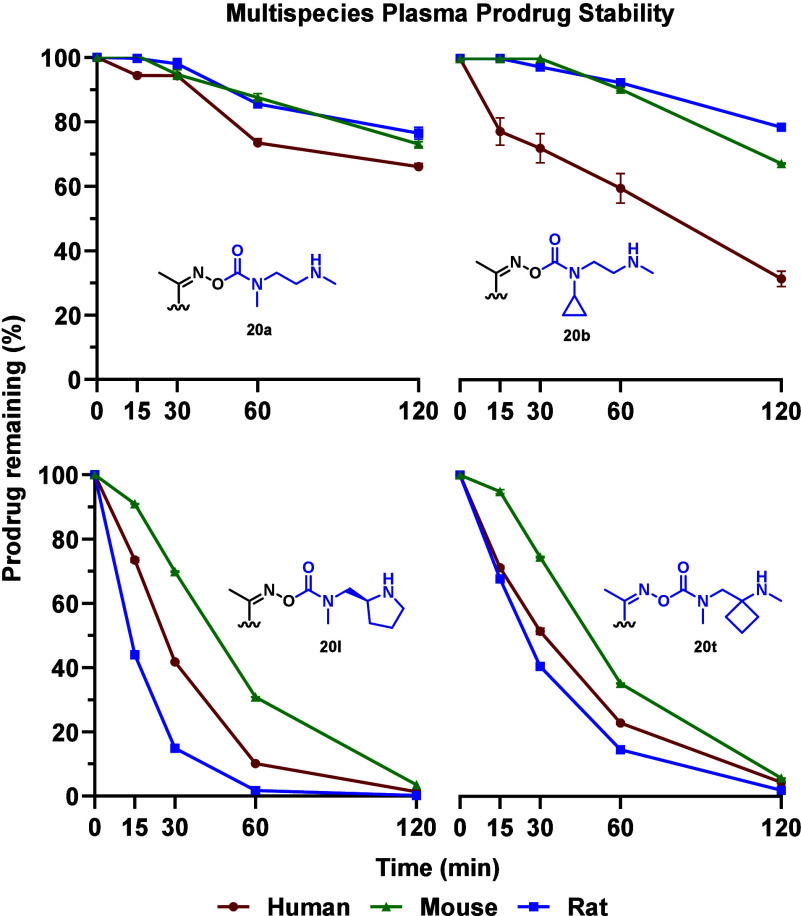
Plasma stability profiles for **20a**, **20b**, **20l** and **20t** across human, mouse, and rat plasma over a 2 h time course. Data presented as mean ± SD (*n* = 2).

In summary, we established a modular, pH-responsive self-immolative linker platform for the rational design of neurosteroid oxime prodrugs with tunable release kinetics. By systematically modifying the carbamate *N*-substituent with steric and electronic tethers, and exploring heterocyclic and angle-strained motifs, we achieved fine control over *syn*/*anti* rotamer populations. This approach demonstrated a broad range of release rates and enabled tailored prodrug performance. Experimental data across pH, temperature, and media demonstrated that electronic, heterocyclic and angle strain modifications reliably promoted efficient release in both PBS and plasma, while steric class prodrugs showed susceptibility to enzymatic stabilization. *In silico* modeling aligned with experimental trends in PBS, indicating that *syn/anti* energy differences and p*K*_a_ values are predictive of reactivity in buffered conditions. However, these predictions were only partially confirmed in plasma, likely due to additional enzyme-mediated and matrix-dependent effects not captured by current models. Moreover, minimal interspecies differences in plasma stability, aside from modest effects in the steric series, streamline preclinical model selection. Among the compounds tested, **20t** emerged as a particularly promising lead, combining rapid self-immolation with favorable storage stability – features that highlight its translational potential as a field-ready injectable for acute neuro-injury applications. While we mainly focused on progesterone analogs, we expect that allopregnanolone prodrugs will exhibit comparable behavior and therapeutic utility. Moreover, we demonstrate that this platform is readily adaptable to other neurosteroid scaffolds, supported by robust synthetic access that may also extend to additional drug candidates beyond those discussed here. This study advances our understanding of structure–reactivity relationships in SI linker systems and supports their broader application for the controlled delivery of neuroactive agents, particularly in contexts requiring tailored release during acute or sustained therapeutic windows.

## Supplementary Material



## Notes

Detailed and complete solubility and stability data, computational methods and experimental procedures, analytical and spectroscopic data (^1^H, ^13^C, HRMS) and NMR spectra of final compounds (PDF)

## Abbreviations

CNS: central nervous system
NAS: neuroactive steroid
CDKL5: cyclin-dependent kinase-like 5
TBI: traumatic brain injury
BBB: blood-brain barrier
SI: self-immolative
POM: phosphorousoxymethylene

## References

[1] a Baulieu, E. E. ; Robel, P. ; Schumacher, M. Neurosteroids: Beginning of the story; Academic Press, 2001. 10.1016/s0074-7742(01)46057-0. 11599297

[2] Steinmetz J. D., Seeher K. M., Schiess N., Nichols E., Cao B., Servili C., Cavallera V., Cousin E., Hagins H., Moberg M. E. (2024). Global, Regional, and National Burden of Disorders
Affecting the Nervous System, 1990–2021: a Systematic Analysis
for the Global Burden of Disease Study 2021. Lancet Neurol..

[3] Schüle C., Nothdurfter C., Rupprecht R. (2014). The Role of
Allopregnanolone in Depression
and Anxiety. Prog. Neurobiol..

[4] Hernandez G. D., Solinsky C. M., Mack W. J., Kono N., Rodgers K. E., Wu C.-Y., Mollo A. R., Lopez C. M., Pawluczyk S., Bauer G. (2020). Safety,
Tolerability, and Pharmacokinetics of Allopregnanolone
as a Regenerative Therapeutic for Alzheimer’s Disease: A Single
and Multiple Ascending Dose Phase 1b/2a Clinical Trial. Alzheimer’s Dement..

[5] Bourque M., Morissette M., Di Paolo T. (2024). Neuroactive Steroids and Parkinson’s
Disease: Review of Human and Animal Studies. Neurosci Biobehav Rev..

[6] González S. L., Meyer L., Raggio M. C., Taleb O., Coronel M. F., Patte-Mensah C., Mensah-Nyagan A. G. (2019). Allopregnanolone and Progesterone
in Experimental Neuropathic Pain: Former and New Insights with a Translational
Perspective. Cell Mol. Neurobiol..

[7] Xu J., Zhou Y., Yan C., Wang X., Lou J., Luo Y., Gao S., Wang J., Wu L., Gao X. (2022). Neurosteroids:
A Novel Promise for the Treatment of Stroke and Post-stroke
Complications. J. Neurochem..

[8] Sayeed, I. ; Stein, D. G. Progesterone as a Neuroprotective Factor in Traumatic and Ischemic Brain Injury. In Prog. Brain Res., Verhaagen, J. , Hol, E. M. , Huitenga, I. , Wijnholds, J. , Bergen, A. B. , Boer, G. J. , Swaab, D. F. , Eds.; Vol. 175; Elsevier, 2009; pp 219–237. 10.1016/S0079-6123(09)17515-5 19660659

[9] Verdoorn T. A., Parry T. J., Pinna G., Lifshitz J. (2023). Neurosteroid
Receptor Modulators for Treating Traumatic Brain Injury. Neurotherapeutics.

[10] Blanco M.
J., La D., Coughlin Q., Newman C. A., Griffin A. M., Harrison B. L., Salituro F. G. (2018). Breakthroughs in Neuroactive Steroid Drug Discovery. Bioorg. Med. Chem. Lett..

[11] Heo Y.-A. (2023). Zuranolone: First Approval. Drugs.

[12] Lamb Y. N. (2022). Ganaxolone: First Approval. Drugs.

[13] Schumacher M., Mattern C., Ghoumari A., Oudinet J. P., Liere P., Labombarda F., Sitruk-Ware R., De Nicola A. F., Guennoun R. (2014). Revisiting the Roles
of Progesterone and Allopregnanolone in the Nervous System: Resurgence
of the Progesterone Receptors. Prog. Neurobiol..

[14] Deutsch E. R., Espinoza T. R., Atif F., Woodall E., Kaylor J., Wright D. W. (2013). Progesterone’s
Role in Neuroprotection, a Review of the Evidence. Brain Res..

[15] Attella M.
J., Nattinville A., Stein D. G. (1987). Hormonal State Affects Recovery from Frontal Cortex
Lesions in Adult Female Rats. Behav Neural Biol..

[16] Stein D. G. (2008). Progesterone Exerts Neuroprotective
Effects After Brain Injury. Brain Res. Rev..

[17] Zhou Z., Li Y., Peng R., Shi M., Gao W., Lei P., Zhang J. (2024). Progesterone Induces
Neuroprotection Associated with Immune/inflammatory
Modulation in Experimental Traumatic Brain Injury. NeuroReport.

[18] Wright D. W., Kellermann A. L., Hertzberg V. S., Clark P. L., Frankel M., Goldstein F. C., Salomone J. P., Dent L. L., Harris O. A., Ander D. S. (2007). ProTECT: a Randomized Clinical Trial of Progesterone for Acute Traumatic
Brain Injury. Ann. Emerg Med..

[19] Skolnick B. E., Maas A. I., Narayan R. K., van der
Hoop R. G., MacAllister T., Ward J. D., Nelson N. R., Stocchetti N. (2014). A Clinical Trial of Progesterone for Severe Traumatic
Brain Injury. N Engl J. Med..

[20] b Stein, D. G. ; Howard, R. B. ; Sayeed, I. Chapter 1 - Why Did the Phase III Clinical Trials for Progesterone in TBI Fail? An Analysis of Three Potentially Critical Factors. In New Therapeutics for Traumatic Brain Injury, Heidenreich, K. A. , Ed.; Academic Press, 2017; pp 3–18.

[21] MacNevin C. J., Atif F., Sayeed I., Stein D. G., Liotta D. C. (2009). Development
and Screening of Water-Soluble Analogues of Progesterone and Allopregnanolone
in Models of Brain Injury. J. Med. Chem..

[22] Guthrie D.
B., Stein D. G., Liotta D. C., Lockwood M. A., Sayeed I., Atif F., Arrendale R. F., Reddy G. P., Evers T. J., Marengo J. R. (2012). Water-Soluble Progesterone Analogues Are Effective,
Injectable Treatments in Animal Models of Traumatic Brain Injury. ACS Med. Chem. Lett..

[23] Stein D. G. (2011). Progesterone
in the Treatment of Acute Traumatic Brain Injury: a Clinical Perspective
and Update. Neuroscience.

[24] Sayeed I., Wali B., Guthrie D. B., Saindane M. T., Natchus M. G., Liotta D. C., Stein D. G. (2019). Development
of a Novel Progesterone Analog in the Treatment of Traumatic Brain
Injury. Neuropharmacology.

[25] Dal
Corso A., Borlandelli V., Corno C., Perego P., Belvisi L., Pignataro L., Gennari C. (2020). Fast Cyclization of
a Proline-Derived Self-Immolative Spacer Improves the Efficacy of
Carbamate Prodrugs. Angew. Chem. Int. Ed..

[26] Fritzemeier R. G., van der Westhuyzen A. E., D’Erasmo M., Sharma S. K., Bartsch P., Hodson L. E., Liu K., Wali B., Sayeed I., Liotta D. C. (2023). Neurotherapeutic
Potential of Water-Soluble pH-Responsive Prodrugs of EIDD-036 in Traumatic
Brain Injury. J. Med. Chem..

[27] Alouane A., Labruère R., Le Saux T., Schmidt F., Jullien L. (2015). Self-Immolative
Spacers: Kinetic Aspects, Structure–Property Relationships,
and Applications. Angew. Chem. Int. Ed..

[28] Moraczewski A. L., Banaszynski L. A., From A. M., White C. E., Smith B. D. (1998). Using Hydrogen
Bonding to Control Carbamate C–N Rotamer Equilibria. J. Org. Chem..

[29] Ghosh A. K., Brindisi M. (2015). Organic Carbamates
in Drug Design and Medicinal Chemistry. J. Med.
Chem..

[30] Thurston R., Zantop V., Park K. S., Maid H., Seitz A., Heinrich M. R. (2022). pH-Dependent Conformational Switching of Amide Bonds—from
Full trans to Full cis and Vice Versa. Org.
Lett..

[31] a Dal Corso, A. ; Frigoli, M. ; Prevosti, M. ; Mason, M. ; Bucci, R. ; Belvisi, L. ; Pignataro, L. ; Gennari, C. Advanced Pyrrolidine-Carbamate Self-Immolative Spacer with Tertiary Amine Handle Induces Superfast Cyclative Drug Release. ChemMedChem. 2022, 17 (15). 10.1002/cmdc.202200279. PMC9544318 35620983

[32] b Ng, S. Y. ; Lee, A. Y. W. Traumatic Brain Injuries: Pathophysiology and Potential Therapeutic Targets. Front. Cell. Neurosci. 2019, 13. 10.3389/fncel.2019.00528. PMC6890857 31827423

