# Supplementary material for: Rotamer-Controlled Self-Immolative Linkers Enable Tunable Release of Neurosteroid Oxime Prodrugs
Source: ACS Med Chem Lett. 2025 Sep 25;16(10):2022–31. doi: 10.1021/acsmedchemlett.5c00452 (PMC12516379; doi:10.1021/acsmedchemlett.5c00452)
Supplement: Supplementary file 1 [file ml5c00452_si_001.pdf]

Supporting Information

Rotamer-Controlled Self-Immolative Linkers Enable Tunable Release of Neurosteroid Oxime Prodrugs

Aletta E. van der Westhuyzen,‡ Luke E. Hodson,‡ Gouthami Pashikanti, Russell Fritzemeier, Sean B. Yeung, Andrea Mancia, Deston R. Lian, Paul Joseph Tholath, Alejandro Cubillos Paez, Lahu N. Chavan, Michael D'Erasmo, Yanli Yang, Ken Liu and Dennis C. Liotta*

Department of Chemistry, Emory University, 1515 Dickey Drive, Atlanta, Georgia, 30322, United States

**Correspondence to: dliotta@emory.edu*

Table of Contents

1. Stability Studies S3

1.1 Aqueous Stability in PBS and Acetate Buffer *via* HPLC S3

1.2 Stability in Plasma and PBS *via* LCMS S20

2. Aqueous Solubility S45

3. Summary of Stability Data S46

4. Computational Studies S48

5. General Experimental S52

6. General Synthetic Procedures S53

6.1 General Procedure A: Reductive Amination S53

6.2 General Procedure B: Cbz Protection S53

6.3 General Procedure C: Methylation S53

6.4 General Procedure D: Cbz Deprotection S54

6.5 General Procedure E: Introduction of Amines S54

6.6 General Procedure F: Boc Deprotection S54

7. Synthetic Procedures and Characterization S55

7.1 Preparation of Activated C20-Oxime Intermediates 13 and 14 ...... S55

7.2 Preparation of Amine Linkers S57

7.3 Protected Prodrugs of Progesterone and Allopregnanolone C-20 Oxime S76

7.4 Preparation of Target Progesterone and Allopregnanolone C-20 Oxime Prodrugs S93

7.5 NMR Spectra of Final Compounds S112

7.6 Representative HPLC Traces of Select Final Compounds S145

8. References S150

1. Stability Studies

Stability studies were conducted in PBS and acetate buffer utilizing high performance liquid chromatography for potential drug storage considerations. For this purpose, progesterone prodrugs (1 mg/mL) were incubated at room temperature, detected and monitored via UV spectrometry. Stability experiments in human plasma and PBS were performed via liquid chromatography-tandem mass spectrometry (LC-MS/MS) to determine and compare rate of cleavage of progesterone prodrugs as well as selected allopregnanolone prodrugs (3 μM) when incubated at 37 °C. All experiments were performed in duplicate and results are presented as the average of two experimental replicates.

1.1 Aqueous Stability in PBS and Acetate Buffer *via* HPLC

Material and Instrumentation

HPLC grade acetonitrile (MeCN) was HPLC grade water (H_2O), and formic acid (FA) were purchased from Fisher Scientific. Filtration of undissolved material was achieved with Whatman Puradisc 4 mm PTFE (0.45 μm) syringe filters from Cytiva. Gibco® Dulbecco's phosphate-buffered saline (DPBS 1X, no calcium, no magnesium, pH = 7.03) and acetate buffer (pH = 5.0, 10 mM) were purchased directly from Thermo Fisher Scientific and Cytiva, respectively. Aqueous stability experiments were performed on an Agilent 1100 HPLC equipped with an Agilent G1315B diode array detector, an Agilent G1316A heated column compartment, and a Zorbax Eclipse XDB-C18 (4.6 x 150 mm, 5 μm) column.

Procedure for Aqueous Stability Experiments

Each sample was first weighed out in two separate 2.0 mL vials using an analytical balance. Unless otherwise specified, the test compounds were then dissolved in the aqueous media (PBS or acetate buffer) at a standard concentration of 1 mg/mL. All mixtures were subsequently sonicated for approximately 30 sec. With exception to extremely unstable molecules ($t_{1/2}$ < 30 min), precipitates observed in sample solutions were filtered with a PTFE syringe filter prior to analysis. Immediately after sample preparation, injection by the HPLC autosampler was performed, which was recorded as the initial time point (t = 0 min). Solutions were then kept in the autosampler at RT and reinjected at designated intervals without additional filtration or sample modification. Remaining prodrug was defined by the area under the curve (AUC) at 254 nm. Aqueous stability was ascertained by calculating the percentage of the parent compound remaining after each time point. Linear regression of the time (x-axis) vs. ln(%remaining) (y-axis) plot afforded the rate constant k (slope = $-k$). Pseudo first-order half-lives were then calculated by the following equation: $t_{1/2} = 0.693/k$.

Stability Results in PBS and Acetate Buffer for Storage Considerations

Table S1: Stability of Steric Control Class in PBS Buffer at RT

Time	Prodrug RT (min)	AUC @ 254 nm		Average AUC	Remaining (%)	ln(Prodrug %Remaining)
		A	B			
**20a**						
0	1.281	1215.62	1233.29	1224.46	100	4.605
60	1.281	1175.09	1184.29	1179.69	96.34	4.568
120	1.281	1145.67	1154.17	1149.92	93.91	4.542
240	1.279	1132.72	1113.97	1123.35	91.74	4.519
360	1.279	1097.61	1081.85	1089.73	89.00	4.489
480	1.28	1048.55	1051.66	1050.11	85.76	4.452
1440	1.279	845.25	841.30	843.28	68.87	4.232
y = -0.0002x + 4.5822<br>R^2 = 0.9907					t1/2 = 46.9 ± 0.77 h	
**20b**						
0	1.242	768.95	757.77	763.36	100	4.605
120	1.243	714.53	708.72	711.62	93.22	4.535
240	1.243	652.92	662.69	657.81	86.17	4.456
360	1.243	628.99	609.22	619.10	81.10	4.396
480	1.243	575.08	565.73	570.40	74.72	4.314
1440	1.242	417.14	413.41	415.28	54.40	3.996
y = -0.0004103x + 4.5642714<br>R^2 = 0.9742189					$t_{1/2}$ = 28.16 ± 0.04 h	
**20c**						
0	1.245	902.95	878.33	890.64	100.00	4.605
120	1.246	801.44	788.18	794.81	89.24	4.491
240	1.246	709.30	721.25	715.28	80.31	4.386
360	1.246	634.12	634.34	634.23	71.21	4.266
480	1.246	580.62	584.49	582.55	65.41	4.181
1440	1.244	368.84	371.51	370.18	41.56	3.727
y = -0.0005859x + 4.5337956<br>R^2 = 0.9670002					$t_{1/2}$ = 19.72 ± 0.38 h	
**20d**						
0	1.248	768.61	687.81	728.21	100.00	4.605
120	1.247	680.59	592.71	636.65	87.43	4.471
240	1.248	616.71	533.46	575.08	78.97	4.369
360	1.247	510.03	471.16	490.59	67.37	4.210
480	1.247	483.45	411.42	447.44	61.44	4.118
1440	1.247	193.89	158.51	176.20	24.20	3.186
y = -0.0009796x + 4.5909722					$t_{1/2}$ = 11.79 ± 0.51 h	

$R^2 = 0.9990585$						
**20e**						
0	1.253	855.41	714.37	784.89	100.00	4.605
120	1.254	720.68	601.40	661.04	84.22	4.433
240	1.254	668.46	528.46	598.46	76.25	4.334
360	1.253	597.12	487.45	542.29	69.09	4.235
480	1.254	507.68	396.47	452.07	57.60	4.053
1440	1.253	176.69	150.46	163.57	20.84	3.037
$y = -0.001080x + 4.591611$ $R^2 = 0.998436$					$t_{1/2} = 10.70 \pm 0.15$ h	
**20f**						
0	1.251	820.58	757.24	788.91	100.00	4.605
120	1.251	768.79	719.41	744.10	94.32	4.547
240	1.252	756.30	700.47	728.39	92.33	4.525
360	1.251	730.04	680.78	705.41	89.42	4.493
480	1.251	708.73	664.63	686.68	87.04	4.466
1440	1.251	568.01	550.28	559.15	70.88	4.261
$y = -0.0002278x + 4.5832068$ $R^2 = 0.9896693$					$t_{1/2} = 50.71 \pm 5.11$ h	

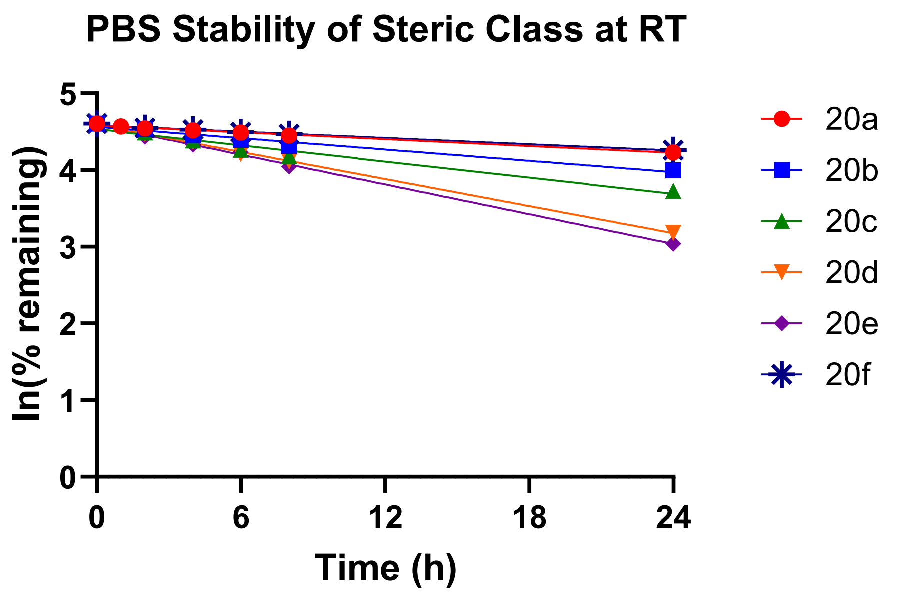

Figure S1: Stability of steric design progesterone prodrugs over time in PBS buffer.

Table S2: Stability of Electronic Control Class in PBS Buffer at RT

Time	Prodrug RT (min)	AUC @ 254 nm		Average AUC	Remaining (%)	ln(Prodrug %Remaining)
		A	B			

**20g**						
0	1.225	667.06	614.39	640.72	100.00	4.605
15	1.225	615.79	565.03	590.41	92.15	4.523
30	1.225	561.61	513.10	537.35	83.87	4.429
60	1.226	469.35	437.66	453.51	70.78	4.260
120	1.225	343.54	305.62	324.58	50.66	3.925
180	1.225	259.01	238.70	248.86	38.84	3.659
y = -0.005317x + 4.592574 R^2 = 0.997201					$t_{1/2}$ = 2.17 ± 0.02 h	
**20h**						
0	1.219	603.79	553.72	578.76	100.00	4.605
50	1.22	439.53	407.88	423.71	73.21	4.293
126	1.222	319.96	287.93	303.95	52.52	3.961
202	1.223	268.58	258.27	263.43	45.52	3.818
278	1.223	227.64	213.72	220.68	38.13	3.641
354	1.223	137.66	179.90	158.78	27.43	3.312
430	1.223	167.07	125.72	146.40	25.29	3.231
507	1.223	185.37	112.83	149.10	25.76	3.249
583	1.223	126.32	152.94	139.63	24.13	3.183
1518	1.214	60.82	21.49	41.16	7.11	1.962
y = -0.0015981x + 4.1723969 R^2 = 0.9064558					$t_{1/2}$ = 7.23 ± 1.9 h	
**20i**						
0	1.224	638.96	636.94	637.95	100.00	4.605
120	1.225	606.32	596.00	601.16	94.23	4.546
240	1.226	585.00	567.59	576.29	90.34	4.504
360	1.226	554.15	541.81	547.98	85.90	4.453
480	1.227	531.48	517.47	524.47	82.21	4.409
1440	1.225	379.75	362.27	371.01	58.16	4.063
y = -0.0003708x + 4.5931657 R^2 = 0.9986729					$t_{1/2}$ = 31.16 ± 1.48 h	
**20j**						
0	1.229	622.79	666.27	644.53	100.00	4.605
120	1.23	575.91	639.50	607.71	94.29	4.546
240	1.23	529.68	591.13	560.41	86.95	4.465
360	1.231	508.50	554.15	531.32	82.44	4.412
480	1.231	477.70	519.04	498.37	77.32	4.348
1440	1.229	287.91	311.78	299.85	46.52	3.840
y = -0.0005307x + 4.6029767 R^2 = 0.9995726					$t_{1/2}$ = 21.77 ± 0.19 h	
**20k**						
0	1.227	672.49	639.37	655.93	100.00	4.605
120	1.227	651.89	606.27	629.08	95.91	4.563
240	1.228	628.54	588.03	608.28	92.74	4.530
360	1.229	596.40	567.35	581.88	88.71	4.485

480	1.228	581.22	555.93	568.57	86.68	4.462
1440	1.227	463.89	448.88	456.38	69.58	4.242
y = -0.0002458x + 4.5895586<br>R² = 0.9922342					$t_{1/2}$ = 47.00 ± 2.84 h	

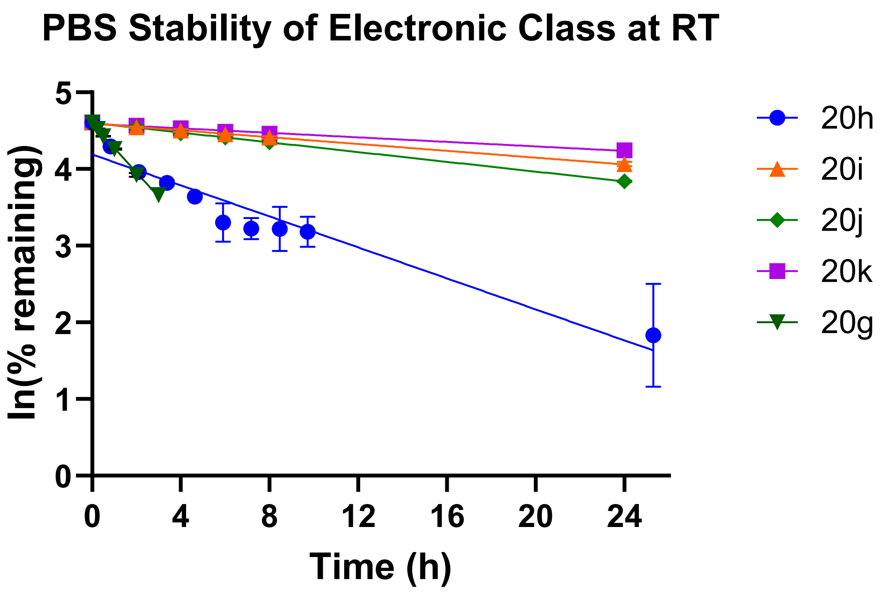

Figure S2: Stability of electronic design progesterone prodrugs over time in PBS buffer.

Table S3: Stability of Heterocyclic Pyrrolidine & Angle Strain Class in PBS Buffer at RT

Time	Prodrug RT (min)	AUC @ 254 nm		Average AUC	Remaining (%)	ln(Prodrug %Remaining)
		A	B			
**20l**						
0	1.285	1233.65	1173.69	1203.67	100.00	4.605
30	1.285	999.50	973.48	986.49	81.96	4.406
60	1.285	811.32	792.44	801.88	66.62	4.199
90	1.286	700.73	630.47	665.60	55.30	4.013
120	1.285	466.13	469.52	467.83	38.87	3.660
y = -0.0076x + 4.6334<br>R² = 0.983					t1/2 = 1.90 ± 0.12 h	
**20m**						
0	1.221	802.22	785.01	793.62	100.00	4.605
61	1.222	663.79	644.47	654.13	82.42	4.412
136	1.221	517.00	478.75	497.88	62.74	4.139
213	1.222	396.98	384.98	390.98	49.27	3.897
290	1.221	328.72	330.17	329.45	41.51	3.726
365	1.221	287.26	280.25	283.76	35.75	3.577
441	1.221	231.52	221.47	226.50	28.54	3.351

518	1.221	184.16	179.80	181.98	22.93	3.132
1483	1.218	46.08	43.99	45.04	5.67	1.736
y = -0.0018795x + 4.3518933<br>R^2 = 0.9599003					$t_{1/2}$ = 6.15 ± 0.02 h	
**20n**						
0	1.22	779.31	714.37	746.84	100.00	4.605
102	1.219	748.40	769.38	758.89	101.61	4.621
245	1.219	781.40	785.21	783.31	104.88	4.653
395	1.219	772.96	780.04	776.50	103.97	4.644
1416	1.219	735.32	765.03	750.18	100.45	4.610
y = -0.000009x + 4.630306<br>R^2 = 0.054551					$t_{1/2}$ = > 24 h	
**20o**						
0	1.222	698.84	484.68	591.76	100.00	4.605
75	1.221	550.62	385.86	468.24	79.13	4.371
151	1.221	419.33	306.46	362.90	61.32	4.116
228	1.222	313.89	241.02	277.46	46.89	3.848
304	1.222	268.31	191.54	229.93	38.85	3.660
380	1.222	213.35	156.96	185.16	31.29	3.443
456	1.221	182.03	124.45	153.24	25.90	3.254
532	1.221	155.20	106.40	130.80	22.10	3.096
1467	1.218	44.01	29.95	36.98	6.25	1.832
y = -0.0018340x + 4.3127839<br>R^2 = 0.9407466					$t_{1/2}$ = 6.30 ± 0.09 h	
**20u**						
0	1.22	548.84	564.59	564.59	100.00	4.605
90	1.223	464.35	462.67	462.67	81.95	4.406
166	1.223	401.96	387.55	387.55	68.64	4.229
242	1.223	310.23	336.16	336.16	59.54	4.087
319	1.223	277.95	275.96	275.96	48.88	3.889
394	1.224	257.92	249.35	249.35	44.16	3.788
470	1.223	226.36	225.45	225.45	39.93	3.687
547	1.223	183.99	196.60	196.60	34.82	3.550
1483	1.22	84.32	54.86	54.86	9.72	2.274
y = -0.00153468x + 4.46783848<br>R^2 = 0.98507870					$t_{1/2}$ = 7.53 ± 1.30 h	
**20v**						
0	1.216	56.79	52.96	54.88	100.00	4.605
102	1.216	37.96	48.86	43.41	79.11	4.371
246	1.213	28.85	44.23	36.54	66.59	4.199
395	1.213	26.28	29.04	27.66	50.41	3.920
1416	1.218	77.63	37.81	57.72	105.18	4.656
y = 0.0001753x + 4.2743794<br>R^2 = 0.1088653					$t_{1/2}$ = > 24 h	
**20q**						

0	1.227	677.20	773.90	725.55	100.00	4.605
34	1.227	558.38	647.01	602.70	83.07	4.420
51	1.226	503.93	580.18	542.05	74.71	4.314
68	1.225	437.75	526.30	482.02	66.44	4.196
1014	1.226	87.05	32.89	59.97	8.27	2.112
1075	1.22	22.74	32.93	27.84	3.84	1.345
y = 0.0027x + 4.492<br>R^2 = 0.9784					$t_{1/2}$ = 4.35 h	
**20t**						
0	1.222	762.15	844.43	803.29	100.00	4.605
17	1.226	742.72	806.44	774.58	96.43	4.569
34	1.226	712.69	774.08	743.38	92.54	4.528
51	1.226	692.40	731.87	712.14	88.65	4.485
68	1.225	631.36	705.05	668.21	83.18	4.421
167	1.224	462.61	391.25	426.93	53.15	3.973
420	1.232	309.31	429.68	369.50	46.00	3.829
1136	1.222	249.66	202.04	225.85	28.12	3.336
y = 0.0011x + 4.4788<br>R^2 = 0.8708					$t_{1/2}$ = 10.49 h	

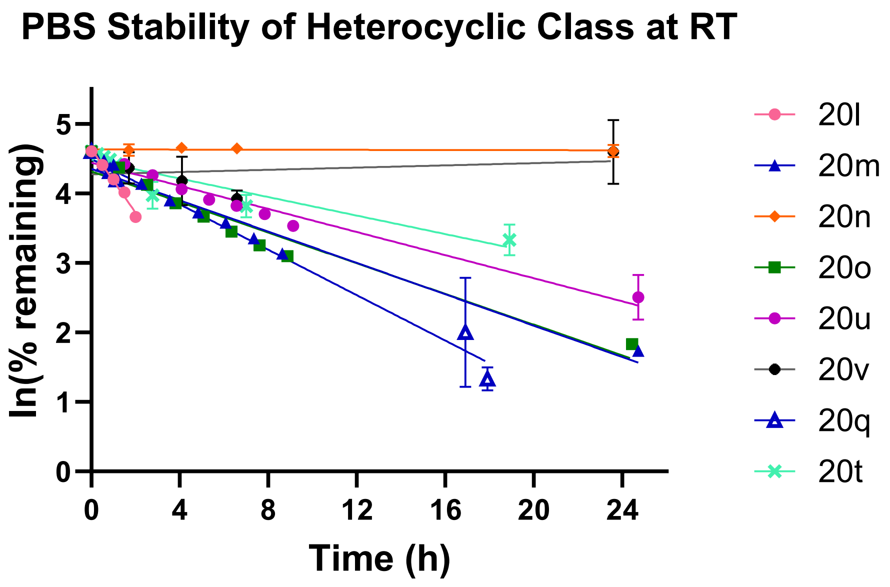

Figure S3: Stability of heterocyclic pyrrolidine design and angle strain progesterone prodrugs over time in PBS buffer.

Table S4: Stability of Nucleophile Design Class in PBS Buffer at RT

Time	Prodrug RT (min)	AUC @ 254 nm		Average AUC	Remaining (%)	ln(Prodrug %Remaining)
		A	B			
**20w**						

0	1.217	660.7	621.45	641.08	100	4.605
120	1.219	534.3	506.76	520.53	81.2	4.397
240	1.218	471.57	428.74	450.15	70.22	4.252
360	1.219	395.3	370.06	382.68	59.69	4.089
480	1.22	350.51	321.92	336.21	52.44	3.96
1440	1.225	128.03	109.8	118.91	18.55	2.92
y = -0.001140x + 4.538697<br>R^2 = 0.995608					$t_{1/2}$ = 10.13 ± 0.41 h	
**20x**						
0	1.219	693.68	675.74	684.71	100	4.605
120	1.219	504.28	479.89	492.09	71.87	4.275
240	1.22	409.43	386.29	397.86	58.11	4.062
360	1.22	354.23	343.3	348.76	50.94	3.931
480	1.221	240.92	275.18	258.05	37.69	3.629
1440	1.223	214.17	200.64	207.41	30.29	3.411
y = -0.007657x + 4.559775<br>R^2 = 0.988983					$t_{1/2}$ = 1.51 ± 0.05 h	
**20y**						
0	1.221	795.64	795.41	795.53	100	4.605
60	1.222	704.02	669.92	686.97	86.35	4.458
135	1.223	518.63	515.04	516.84	64.97	4.174
211	1.223	400.09	423.62	411.86	51.77	3.947
288	1.223	321.58	321.58	321.58	40.42	3.699
440	1.223	211.47	206.18	208.83	26.25	3.268
1440	1.22	68.97	77.91	73.44	9.23	2.223
y = -0.0015854x + 4.3507068<br>R^2 = 0.9197036					$t_{1/2}$ = 0.017 ± 0.002 h	
**20z**						
0	1.228	895.4	851.29	873.35	100	4.605
120	1.23	793.52	768.19	780.85	89.41	4.493
240	1.231	737.91	715.63	726.77	83.22	4.421
360	1.231	677.3	656.07	666.68	76.34	4.335
480	1.231	643.61	601.46	622.53	71.28	4.267
1440	1.229	498.59	441.23	469.91	53.81	3.985
y = -0.0004012x + 4.5276789<br>R^2 = 0.9369815					$t_{1/2}$ = 28.79 ± 3.16 h	

PBS Stability of Nucleophile Class at RT

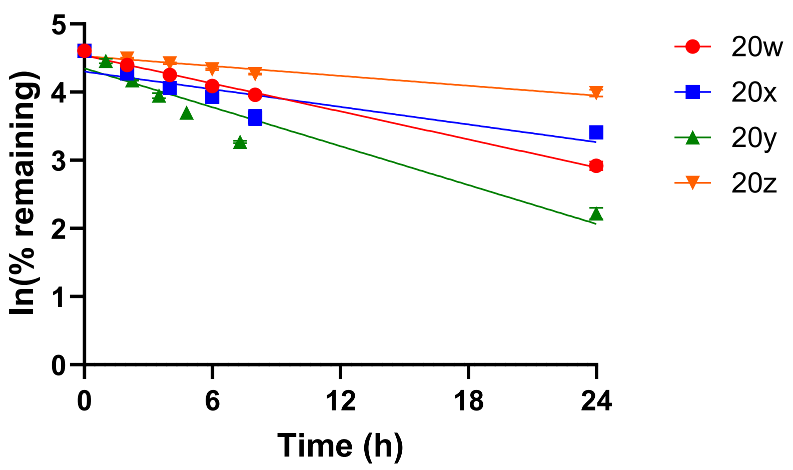

Figure S4: Stability of nucleophile design progesterone prodrugs over time in PBS buffer.

Table S5: Stability of Steric Control Class in Acetate Buffer at RT

Time	Prodrug RT (min)	AUC @ 254 nm		Average AUC	Remaining (%)	ln(Prodrug %Remaining)
		A	B			
**20a**						
0	1.198	1220.55	1192.41	1206.48	100.00	4.605
60	1.201	1233.14	1204.07	1218.61	101.00	4.615
120	1.201	1229.79	1196.34	1213.07	100.55	4.611
240	1.201	1225.69	1200.92	1213.31	100.57	4.611
360	1.202	1221.74	1206.57	1214.16	100.64	4.612
480	1.202	1225.51	1196.76	1211.14	100.39	4.609
1440	1.201	1204.59	1189.56	1197.08	99.22	4.597
2880	1.202	1175.80	1176.60	1176.20	97.49	4.580
4320	1.2	1188.03	1160.71	1174.37	97.34	4.578
7200	1.201	1149.18	1132.88	1141.03	94.58	4.549
y = -0.000006x + 4.6115<br>R^2 = 0.9658					t1/2 = >7d	
**20b**						
0	1.164	828.32	826.54	827.43	100.00	4.605
120	1.166	851.23	809.53	830.38	100.36	4.609
240	1.165	822.02	806.36	814.19	98.40	4.589
480	1.166	825.53	808.10	816.82	98.72	4.592
1440	1.167	827.26	805.81	816.53	98.68	4.592
2880	1.166	825.91	805.32	815.61	98.57	4.591
5760	1.166	795.99	784.98	790.49	95.54	4.559
10080	1.164	771.89	764.86	768.37	92.86	4.531

y = -0.000006929x + 4.601753950 R² = 0.931349865					$t_{1/2}$ = >7 d	
**20c**						
0	1.165	783.63	813.11	798.37	100.00	4.605
120	1.166	790.05	812.88	801.47	100.39	4.609
240	1.167	772.16	828.77	800.47	100.26	4.608
480	1.168	766.13	813.07	789.60	98.90	4.594
1440	1.167	762.98	804.18	783.58	98.15	4.586
2880	1.166	753.00	786.09	769.54	96.39	4.568
5760	1.168	733.62	777.65	755.63	94.65	4.550
10080	1.164	709.45	746.85	728.15	91.21	4.513
y = -0.000009280x + 4.603643213 R² = 0.974040977					$t_{1/2}$ = >7 d	
**20d**						
0	1.165	639.57	634.26	636.92	100.00	4.605
120	1.166	642.93	634.76	638.85	100.30	4.608
240	1.167	635.82	631.02	633.42	99.45	4.600
480	1.166	627.35	616.56	621.96	97.65	4.581
1440	1.166	629.73	617.70	623.72	97.93	4.584
2880	1.165	609.13	598.14	603.63	94.77	4.551
5760	1.167	591.61	573.99	582.80	91.50	4.516
10080	1.168	559.77	536.77	548.27	86.08	4.455
y = -0.00001470x + 4.60132082 R² = 0.98309094					$t_{1/2}$ = >7 d	
**20e**						
0	1.169	760.21	739.14	749.67	100.00	4.605
120	1.17	751.46	725.27	738.36	98.49	4.590
240	1.171	756.27	726.68	741.47	98.91	4.594
480	1.172	744.07	722.88	733.47	97.84	4.583
1440	1.171	736.75	709.68	723.22	96.47	4.569
2880	1.169	719.92	705.69	712.80	95.08	4.555
5760	1.171	688.60	668.54	678.57	90.51	4.506
10080	1.167	647.78	628.02	637.90	85.09	4.444
y = -0.00001522x + 4.59569278 R² = 0.99183011					$t_{1/2}$ = >7 d	
**20f**						
0	1.168	739.76	802.44	771.10	100.00	4.605
120	1.169	752.15	801.36	776.75	100.73	4.612
240	1.17	743.00	803.87	773.44	100.30	4.608
480	1.171	750.80	802.91	776.85	100.75	4.613
1440	1.171	740.30	799.92	770.11	99.87	4.604
2880	1.169	738.39	801.27	769.83	99.84	4.604
5760	1.171	736.54	796.26	766.40	99.39	4.599
10080	1.168	731.77	784.61	758.19	98.33	4.588
y = -0.000002052x + 4.609537059					$t_{1/2}$ = >7 d	

R² = 0.872292399	

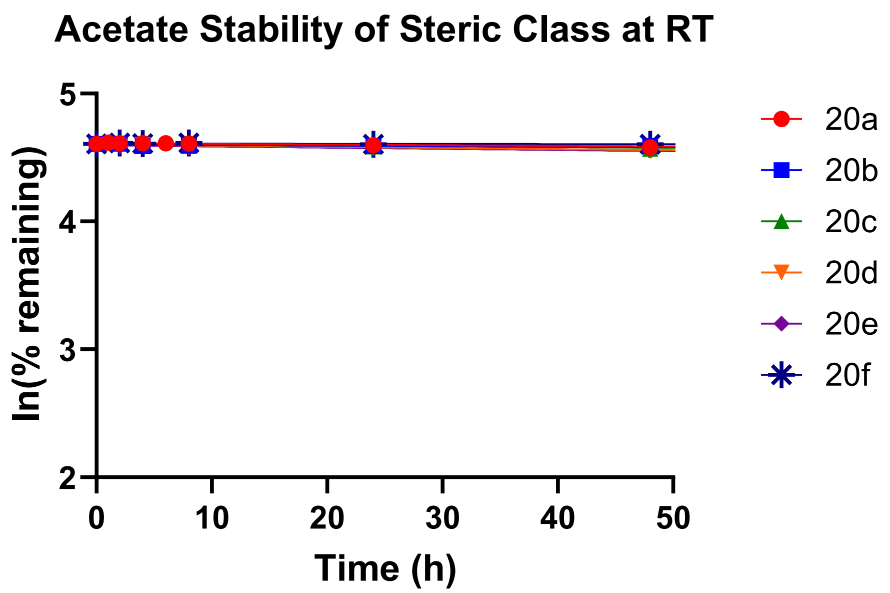

Figure S5: Stability of steric design progesterone prodrugs over time in acetate buffer.

Table S6: Stability of Electronic Control Class in Acetate Buffer at RT

Time	Prodrug RT (min)	AUC @ 254 nm		Average AUC	Remaining (%)	ln(Prodrug %Remaining)
		A	B			
**20g**						
0	1.088	653.24	559.92	606.58	100.00	4.605
120	1.09	648.89	545.76	597.32	98.47	4.590
240	1.09	635.31	528.32	581.81	95.92	4.563
480	1.091	605.68	521.25	563.47	92.89	4.531
1440	1.094	511.08	443.89	477.48	78.72	4.366
2880	1.102	415.57	355.26	385.42	63.54	4.152
5760	1.12	268.48	237.59	253.03	41.71	3.731
y = -0.0001524x + 4.6002942<br>R^2 = 0.9992103				$t_{1/2}$ = 3.16 ± 0.11 d		
**20h**						
0	1.088	586.50	551.12	568.81	100.00	4.605
114	1.09	573.87	550.42	562.15	98.83	4.593
221	1.092	568.01	543.67	555.84	97.72	4.582
412	1.093	560.67	527.27	543.97	95.63	4.561
679	1.093	531.39	504.46	517.93	91.05	4.511
1440	1.098	494.28	465.05	479.67	84.33	4.435
2880	1.106	421.46	398.17	409.82	72.05	4.277
4320	1.115	356.32	336.13	346.23	60.87	4.109

5760	1.121	301.06	288.06	294.56	51.79	3.947
7200	1.125	270.80	260.78	265.79	46.73	3.844
10080	1.13	202.32	187.24	194.78	34.24	3.533
y = -0.0001070x + 4.5944736<br>R^2 = 0.9978146					$t_{1/2}$ = 4.38 ± 0.02 d	
**20i**						
0	1.088	608.47	580.07	594.27	100.00	4.605
120	1.09	607.52	574.65	591.09	99.46	4.600
240	1.091	607.11	592.91	600.01	100.97	4.615
480	1.091	608.12	578.47	593.29	99.84	4.604
1440	1.092	593.38	563.93	578.65	97.37	4.579
2880	1.094	579.65	556.77	568.21	95.62	4.560
5760	1.099	537.32	527.67	532.50	89.60	4.495
10080	1.098	498.46	495.89	497.18	83.66	4.427
y = -0.00001838x + 4.60877602<br>R^2 = 0.99085590					$t_{1/2}$ = >7 d	
**20j**						
0	1.154	638.00	516.22	577.11	100.00	4.605
120	1.156	623.53	512.06	567.80	98.39	4.589
240	1.155	599.32	496.32	547.82	94.92	4.553
480	1.155	589.48	474.77	532.13	92.20	4.524
1440	1.156	523.99	427.68	475.83	82.45	4.412
2880	1.157	442.89	361.64	402.26	69.70	4.244
5760	1.159	323.61	263.33	293.47	50.85	3.929
y = -0.0001161x + 4.5892334<br>R^2 = 0.9975768					$t_{1/2}$ = 4.14 ± 0.01 d	
**20k**						
0	1.099	613.79	678.59	646.19	100.00	4.605
120	1.101	608.07	673.43	640.75	99.16	4.597
240	1.101	615.18	678.97	647.08	100.14	4.607
480	1.102	621.11	669.16	645.14	99.84	4.604
1440	1.103	600.56	662.19	631.37	97.71	4.582
2880	1.105	589.46	646.29	617.88	95.62	4.560
5760	1.111	562.17	621.87	592.02	91.62	4.518
10080	1.108	535.98	587.37	561.68	86.92	4.465
y = -0.00001419x + 4.60436981<br>R^2 = 0.99154157					$t_{1/2}$ = >7 d	

Acetate Stability of Electronic Class at RT

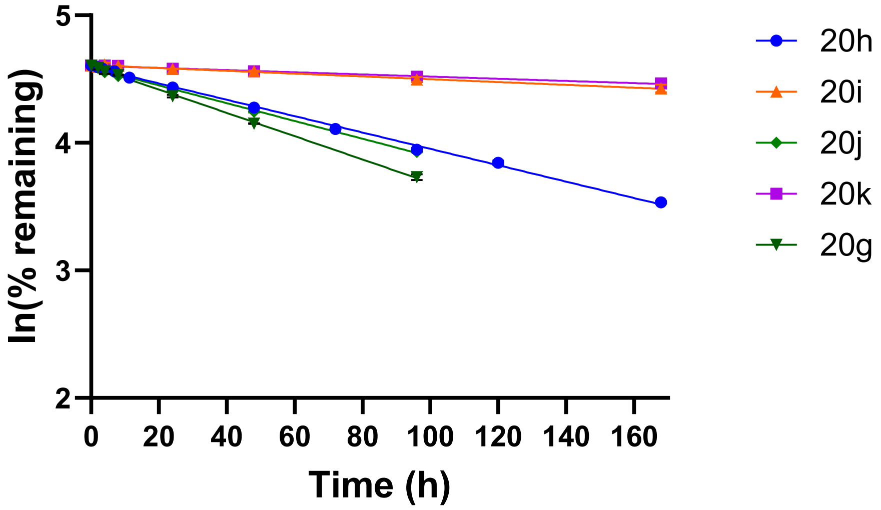

Figure S6: Stability of electronic design progesterone prodrugs over time in acetate buffer.

Table S7: Stability of Heterocyclic Pyrrolidine & Angle Strain Class in Acetate Buffer at RT

Time	Prodrug RT (min)	AUC @ 254 nm		Average AUC	Remaining (%)	ln(Prodrug %Remaining)
		A	B			
**20l**						
0	1.199	1314.63	1355.42	1335.03	100.00	4.605
60	1.202	1292.41	1328.82	1310.61	98.17	4.587
120	1.203	1292.15	1324.93	1308.54	98.02	4.585
240	1.204	1296.89	1322.78	1309.83	98.11	4.586
360	1.203	1276.50	1309.47	1292.98	96.85	4.573
480	1.203	1274.61	1306.61	1290.61	96.67	4.571
1440	1.202	1198.89	1232.01	1215.45	91.04	4.511
2880	1.203	1080.41	1110.03	1095.22	82.04	4.407
4320	1.2	990.36	994.42	992.39	74.33	4.309
y = -0.00007x + 4.5995<br>R^2 = 0.997				t1/2 = 7.29 ± 0.49 d		
**20m**						
0	1.149	776.65	789.45	783.05	100.00	4.605
111	1.151	782.39	794.42	788.41	100.68	4.612
218	1.152	781.34	777.10	779.22	99.51	4.600
409	1.15	759.30	793.98	776.64	99.18	4.597
676	1.149	753.88	774.82	764.35	97.61	4.581
1440	1.149	717.76	755.21	736.49	94.05	4.544

2880	1.148	682.57	703.30	692.94	88.49	4.483
4320	1.148	650.45	651.55	651.00	83.14	4.420
5760	1.148	609.15	825.80	717.48	91.63	4.518
7200	1.147	580.03	582.17	581.10	74.21	4.307
10080	1.147	526.51	532.97	529.74	67.65	4.214
y = -0.0000369x + 4.6094488<br>R^2 = 0.8984248					$t_{1/2}$ = >7 d	
**20n**						
0	1.15	822.12	750.31	786.22	100.00	4.605
121	1.153	835.51	732.09	783.80	99.69	4.602
228	1.152	816.80	749.55	783.18	99.61	4.601
419	1.151	845.23	749.83	797.53	101.44	4.619
690	1.154	808.11	751.50	779.81	99.18	4.597
1440	1.151	822.82	746.39	784.61	99.80	4.603
2880	1.15	802.28	754.59	778.44	99.01	4.595
4320	1.158	816.63	739.98	778.31	98.99	4.595
5760	1.15	814.27	727.14	770.71	98.03	4.585
7200	1.159	729.74	735.17	732.46	93.16	4.534
10080	1.158	794.02	730.22	762.12	96.94	4.574
y = -0.000005x + 4.606954<br>R^2 = 0.576741					$t_{1/2}$ = >7 d	
**20o**						
0	1.149	752.07	720.74	736.41	100.00	4.605
111	1.15	758.40	737.51	747.96	101.57	4.621
218	1.151	764.67	733.88	749.28	101.75	4.622
409	1.15	737.58	727.72	732.65	99.49	4.600
676	1.15	751.57	721.03	736.30	99.99	4.605
1440	1.15	728.87	689.27	709.07	96.29	4.567
2880	1.15	620.21	642.73	631.47	85.75	4.451
4320	1.149	681.41	599.42	640.42	86.97	4.466
5760	1.148	774.27	571.88	673.08	91.40	4.515
7200	1.148	543.82	551.88	547.85	74.40	4.309
10080	1.147	492.75	487.39	490.07	66.55	4.198
y = -0.0000390x + 4.6227322<br>R^2 = 0.8991371					$t_{1/2}$ = >7 d	
**20q**						
0	1.33	372.37	348.57	360.47	100.00	4.605
60	1.34	417.49	318.09	367.79	102.03	4.625
120	1.333	410.07	338.05	374.06	103.77	4.642
273	1.339	410.28	343.76	377.02	104.59	4.650
372	1.342	416.83	352.23	384.53	106.67	4.670
1478	1.343	411.06	348.11	379.59	105.30	4.657
					$t_{1/2}$ = >7 d	
**20t**						

0	1.15	750.18	696.46	723.32	100.00	4.605
60	1.151	692.82	620.06	656.44	90.75	4.508
120	1.15	472.46	401.48	436.97	60.41	4.101
273	1.146	329.63	325.74	327.69	45.30	3.813
372	1.147	378.15	267.03	322.59	44.60	3.798
1478	1.145	241.35	297.12	269.24	37.22	3.617
$y = -0.00005x + 4.6088$<br>$R^2 = 0.9194$					$t_{1/2}$ = >7 d	
**20u**						
0	1.147	532.48	595.29	563.89	100.00	4.605
107	1.149	542.09	578.56	560.33	99.37	4.599
213	1.149	509.10	568.48	538.79	95.55	4.560
405	1.148	501.74	552.85	527.30	93.51	4.538
580	1.154	478.29	525.28	501.79	88.99	4.488
1440	1.148	434.90	442.73	438.82	77.82	4.354
2880	1.148	328.68	340.87	334.78	59.37	4.084
4320	1.148	252.68	269.26	260.97	46.28	3.835
5760	1.149	187.61	257.30	222.46	39.45	3.675
7200	1.149	157.15	176.80	166.98	29.61	3.388
10080	1.148	119.00	114.18	116.59	20.68	3.029
$y = -0.00016033x + 4.58580189$<br>$R^2 = 0.99595207$					$t_{1/2}$ = 3.00 ± 0.020 d	
**20v**						
0	1.15	750.18	696.46	723.32	100.00	4.605
121	1.151	692.82	620.06	656.44	90.75	4.508
228	1.15	472.46	401.48	436.97	60.41	4.101
419	1.146	329.63	325.74	327.69	45.30	3.813
690	1.147	378.15	267.03	322.59	44.60	3.798
1440	1.145	241.35	297.12	269.24	37.22	3.617
2880	1.144	202.71	183.51	193.11	26.70	3.285
4320	1.144	184.63	181.78	183.21	25.33	3.232
5760	1.144	168.14	172.88	170.51	23.57	3.160
7200	1.144	212.31	229.99	221.15	30.57	3.420
10080	1.144	192.09	240.07	216.08	29.87	3.397
$y = -0.0001023x + 4.0297210$<br>$R^2 = 0.4859423$					$t_{1/2}$ = 4.71 ± 1.0 d	

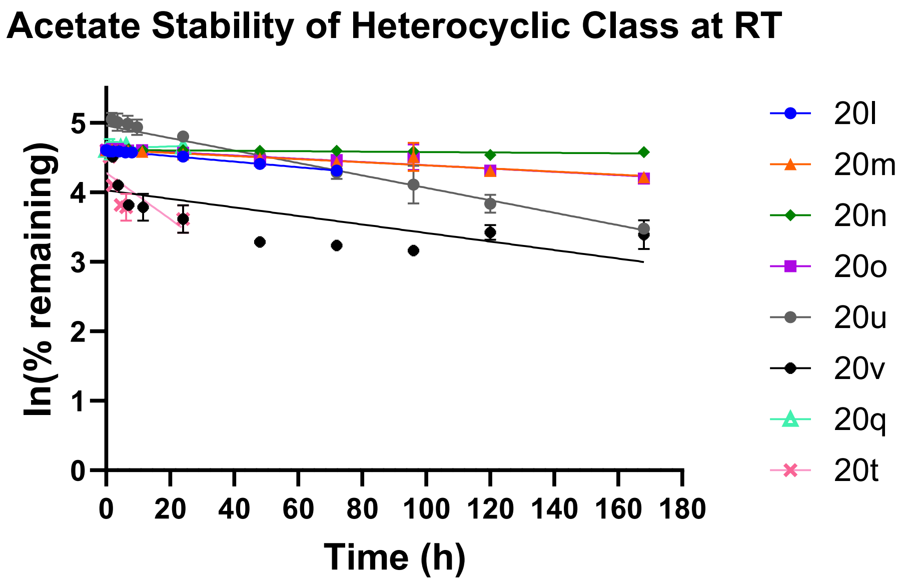

Figure S7: Stability of heterocyclic pyrrolidine design and angle strain progesterone prodrugs over time in acetate buffer.

Table S8: Stability of Nucleophile Design Class in Acetate Buffer at RT

Time	Prodrug RT (min)	AUC @ 254 nm		Average AUC	Remaining (%)	ln(Prodrug %Remaining)
		A	B			
**20w**						
0	1.08	561.18	699.86	630.52	100	4.605
120	1.082	561.67	690.57	626.12	99.3	4.598
240	1.082	564.21	695.28	629.74	99.88	4.604
480	1.082	552.69	693.3	622.99	98.81	4.593
1440	1.083	535.34	657.33	596.33	94.58	4.549
2880	1.084	517.8	631.35	574.58	91.13	4.512
5760	1.088	476.17	582.9	529.54	83.98	4.431
10080	1.088	428.99	530.14	479.57	76.06	4.332
y = -0.00002766x + 4.60064384<br>R^2 = 0.99185897					$t_{1/2}$ = >7 d	
**20x**						
0	1.084	671.5	740.01	705.75	100	4.605
120	1.086	659.34	729.38	694.36	98.39	4.589
240	1.086	642.07	708.72	675.39	95.7	4.561
480	1.086	621.58	693.08	657.33	93.14	4.534
1440	1.089	518.58	565.86	542.22	76.83	4.342
2880	1.094	401.55	454.38	427.97	60.64	4.105
5760	1.109	265.23	302.81	284.02	40.24	3.695
y = -0.0001601x + 4.5970517					$t_{1/2}$ = 3.01 ± 0.08 d	

$R^2 = 0.9965588$						
**20y**						
0	1.1	895.11	888.04	891.58	100	4.605
115	1.103	905.68	871.7	888.69	99.68	4.602
221	1.104	869.45	855.15	862.3	96.72	4.572
412	1.105	849.98	826.67	838.33	94.03	4.544
679	1.104	795.86	783.81	789.84	88.59	4.484
1440	1.107	676.05	678.51	677.28	75.96	4.33
2880	1.113	511.2	516.04	513.62	57.61	4.054
4320	1.12	373.86	391.44	382.65	42.92	3.759
5760	1.128	278.4	288.01	283.21	31.76	3.458
7200	1.134	212.31	224.73	218.52	24.51	3.199
10080	1.137	112.55	126.07	119.31	13.38	2.594
$y = -0.0001999x + 4.6198534$<br>$R^2 = 0.9997943$					$t_{1/2}$ = 7.93± 0.07 min	
**20z**						
0	1.158	927.46	851.49	889.47	100	4.605
120	1.161	968.59	854.26	911.43	102.47	4.63
240	1.161	942.14	841.76	891.95	100.28	4.608
480	1.16	943.65	833.32	888.48	99.89	4.604
1440	1.161	921.1	836.98	879.04	98.83	4.593
2880	1.158	916.66	829.72	873.19	98.17	4.587
5760	1.159	892.35	798.77	845.56	95.06	4.555
10080	1.158	868.98	788	828.49	93.14	4.534
$y = -0.000008239x + 4.611061528$<br>$R^2 = 0.913730604$					$t_{1/2}$ = >7 d	

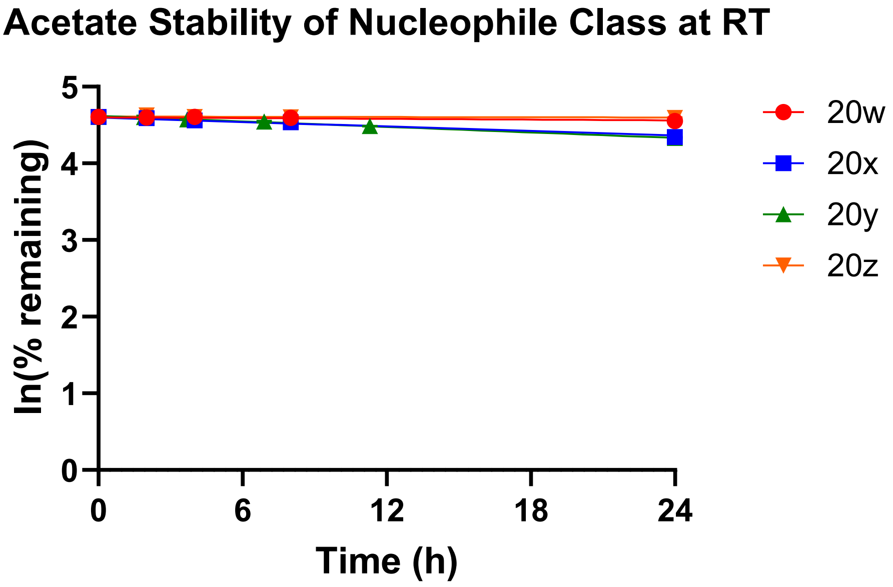

Figure S8: Stability of nucleophile design progesterone prodrugs over time in acetate buffer.

1.2 Stability in Plasma and PBS *via* LCMS

General Experimental

Procaine (positive control) was purchased from Sigma-Aldrich. HPLC grade MeCN, MeOH, H_2O, and FA were purchased from Fischer Scientific. Human plasma (cat. No. HUMANPLLH-0101484), mouse plasma (cat. No. MSE02PLNH-0106426) and rat plasma (RAT00PLLH-0102242) were obtained from BIOIVT. DPBS 1X (no calcium, no magnesium, pH = 7.03) was purchased from Thermo Fischer Scientific. Stock solutions were prepared in 100% DMSO, and suitable dilutions of target analytes were prepared in 0.1% FA /MeCN for method development. For PBS assay Gibco® Dulbecco's phosphate-buffered saline (DPBS 1X, no calcium, no magnesium, pH = 7.03) was utilized. All experiments were performed in duplicate and results are presented as the average of two experimental replicates.

Procedure for Plasma Stability Assay

Test compounds and positive control (i.e. procaine) were dissolved in DMSO to make a 10 mM stock solution and subsequently diluted to 500 µM in 50% MeCN and PBS. This intermediate stock solution was prepared and added immediately prior to the start of each test compound experiment. Plasma was thawed at ambient temperature and aliquoted (663.0 µL) into 1.5 mL Eppendorf tubes. Plasma was incubated at 37 °C for 10 minutes in an incubator shaker at 150 rpm, and the reactions were initiated by addition of the test compound or positive control (4.0 µL, 3 µM). The spiked plasma samples were incubated at 37 °C. For designated time points, 75 µL aliquots were removed from the incubation mixture and immediately quenched in 150 µL of MeCN/0.1%FA with internal standard (ISTD, 2 µM d_5-7-ethoxy coumarin). After completion of experiments, all samples were centrifuged at 15000 rpm for 15 min at 4 °C. The supernatant was transferred to an LC-MS vial and analyzed by LC/MS-MS. Procaine was run with each batch as a positive control for esterase activity in plasma (data not included).

Procedure for PBS Stability Assay

Test compounds were treated in the exact same manner as for the plasma stability assay using 663 µL of PBS instead of plasma.

Instrumentation and Method Development

LC–MS/MS analysis was performed using an Agilent 1260 Infinity II HPLC, coupled with an Agilent G6460 triple quadrupole mass spectrometer (Agilent Technologies, USA). All the data were acquired employing Agilent 6460 Quantitative Analysis data processing software. Reverse-phase HPLC separation for each compound was achieved on either an Agilent Porshell Infinity 120–EC18 column (2.1 X 50 mm, 2.7 µm) or Agilent Porshell Infinity 120–EC8 column (2.1 X 50

mm, 2.7 μm) with a mobile phase composed of MeCN in H_2O spiked with FA (0.1%) at a flow rate of 0.5 mL/min. Each method was developed in the presence of d_5-7-ethoxy coumarin as ISTD. The column temperature was maintained at 40 °C. The detection was operated in Agilent Jet-Stream electrospray positive ionization, using multiple reaction monitoring mode (MRM). All the MRM transitions are reported in Table **S7**. Other MS conditions were as follows: dwell time 100 ms; gas flow 10 L/min; nebulizer pressure 45 psi; delta EMV 200 V; fragmentor voltage and collision energy for individual compounds are shown in the Table S9. The cell accelerator was set to 4 V.

Table S9: Scan parameters for tested compounds and associated transitions in multiple reaction monitoring mode.

Compound #	Precursor Ion MS1	Product Ion MS2	Dwell (ms)	Fragmentor Voltage (V)	Collision Energy (V)
**20a**	444.3	330.2<br>253.1	100	104	9<br>17
**20b**	470.3	253.1<br>141	100	92	21<br>9
**20c**	472.4	253.1<br>143.1	100	96	21<br>9
**20d**	484.4	113.1<br>55.2	200	76	70<br>78
**20e**	498.4	271.1<br>253.1	100	80	13<br>21
**20f**	486.4	253.1<br>157.1	100	82	21<br>9
**20i**	557.4	228.1<br>141	100	100	13<br>50
**20k**	524.4	195.1<br>127	100	128	13<br>37
**20l**	470.3	253.2<br>141.1	100	108	25<br>9
**20m**	470.3	330.3<br>141.1	100	106	17<br>9
**20n**	484.4	253.2<br>155.1	200	130	21<br>13
**20o**	456.3	271.2<br>127	200	106	9<br>9
**20p**	484.36	155.1<br>98.1	100	84	13<br>50
**20q**	496.36	167.1<br>99.1	100	128	9<br>50
**20r**	506.32	177.1	100	82	25

		86.1			50
**20s**	470.34	253.2<br>141.1	100	102	17<br>9
**20t**	484.36	253.1<br>155.1	100	90	25<br>9
**20u**	513.4	253.2<br>184.1	200	110	25<br>17
**20y**	515.4	253.2<br>186.2	100	110	25<br>13
**20z**	504.4	175.1<br>157.1	100	114	13<br>41
**21a**	448.4	334.2<br>115	200	82	9<br>9
**21b**	474.4	334.3<br>141.1	200	102	21<br>9
**21d**	488.4	334.2<br>155	200	72	17<br>9
**21i**	561.4	228.1<br>141.1	200	90	13<br>41
**21k**	528.4	257.2	200	110	17
**21l**	474.4	334.2<br>141.1	200	102	13<br>9

Stability Results of Progesterone Prodrugs in Human Plasma and PBS Buffer

Table S10: Stability of Steric Control Class in Human Plasma at 37 °C

Time (min)	Response		Average Resp.	Remaining (%)	ln(Prodrug %Remaining)	Oxime Formation (nM)	
	A	B				A	B
**20a**							
0	1094952	1106511	1100732	100	4.605	118	118
15	1291624	1299099	1295362	118	4.768	266	264
30	1239307	1243928	1241618	113	4.726	445	444
60	1120962	1128024	1124493	102	4.627	871	855
120	803163	800081	801622	73	4.288	1587	1575
$y = -0.0047x + 4.6419$<br>$R^2 = 0.9733$						$t_{1/2} = 2.45$	
**20b**							
0	761356	679493	720425	100	4.605	27	26
15	565904	545602	555753	77	4.346	252	251
30	524609	511582	518096	72	4.275	589	595

60	429154	427205	428180	59	4.085	1341	1341
120	226598	225161	225880	31	3.445	2279	2284
			y = -0.0091x + 4.5616<br>R^2 = 0.9819			$t_{1/2}$ = 1.27 h	
				**20c**			
0	1234460	1227306	1230883	100	4.605	29	25
15	1466439	1453776	1460108	119	4.776	243	243
30	1234015	1233411	1233713	100	4.607	487	486
60	1017781	1020990	1019386	83	4.417	1068	1069
120	575625	569633	572629	47	3.840	1885	1893
			y = -0.0073x + 4.7759<br>R^2 = 0.8988			$t_{1/2}$ = 1.59 h	
				**20d**			
0	52639	53684	53162	100	4.605	66	65
15	51413	51332	51373	97	4.571	177	178
30	50063	50111	50087	94	4.546	343	348
60	47758	49120	48439	91	4.512	907	890
120	31705	31134	31420	59	4.079	1429	1424
			y = -0.0043x + 4.6572<br>R^2 = 0.8935			$t_{1/2}$ = 2.67 h	
				**20e**			
0	77570	76935	77253	100	4.605	51	48
15	85992	84991	85492	111	4.707	137	137
30	83685	84188	83937	109	4.688	268	268
60	72508	72759	72634	94	4.544	494	490
120	58406	58890	58648	76	4.330	1003	997
			y = -0.0029x + 4.704<br>R^2 = 0.8068			$t_{1/2}$ = 4.02 h	
				**20f**			
0	1739295	1739463	1739379	100	4.605	18	15
15	2138634	2145036	2141835	123	4.813	62	61
30	2047493	2029662	2038578	117	4.764	106	105
60	2068972	2066814	2067893	119	4.778	251	253
120	1651204	1640675	1645940	95	4.550	470	473
			y = -0.0024x + 4.8622<br>R^2 = 0.8822			$t_{1/2}$ = 4.78 h	

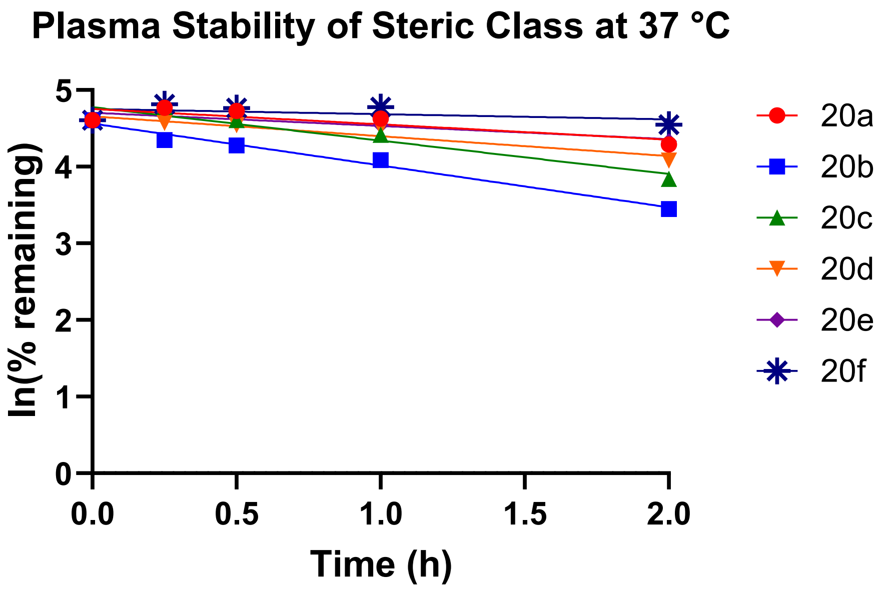

Figure S9: Stability of steric design progesterone prodrugs over time in plasma.

Table S11: Stability of Electronic Control Class in Human Plasma at 37 °C

Time (min)	Response		Average Resp.	Remaining (%)	ln(Prodrug %Remaining)	Oxime Formation (nM)	
	A	B				A	B
**20i**							
0	173335	171784	172560	100	4.605	141	139
15	191345	191484	191415	111	4.709	409	405
30	204784	203388	204086	118	4.773	832	826
60	152076	150557	151317	88	4.474	1594	1595
120	97678	98953	98316	57	4.043	3160	3166
$y = -0.0055x + 4.7696$<br>$R^2 = 0.8175$						$t_{1/2}$ = 2.09 h	
**20k**							
0	697643	703853	700748	100	4.605	153	149
15	774376	773283	773830	110	4.704	277	281
30	693674	686983	690329	99	4.590	497	491
60	630430	627534	628982	90	4.497	1079	1071
120	479539	478777	479158	68	4.225	2180	2190
$y = -0.0037x + 4.6888$<br>$R^2 = 0.8992$						$t_{1/2}$ = 3.16 h	

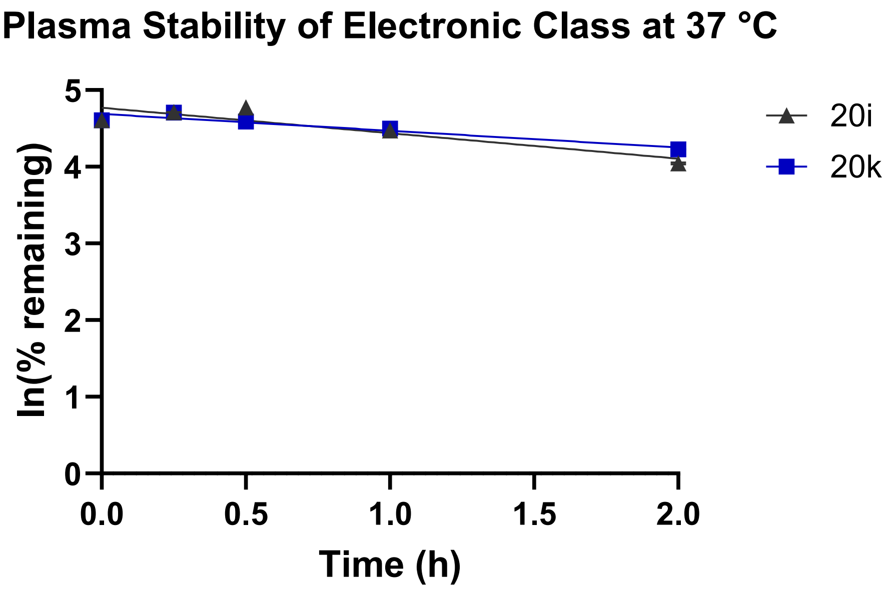

Figure S10: Stability of electronic design progesterone prodrugs over time in plasma.

Table S12: Stability of Heterocyclic Control & Angle Strain Class in Human Plasma at 37 °C

Time (min)	Response		Average Resp.	Remaining (%)	ln(Prodrug %Remaining)	Oxime Formation (nM)	
	A	B				A	B
**20l**							
0	1436691	1427428	1432060	100	4.605	246	244
15	1048480	1056631	1052556	73	4.297	1269	1263
30	597192	598907	598050	42	3.732	2175	2159
60	144679	144429	144554	10	2.312	2779	2813
120	19861	19626	19743.5	1	0.321	3448	3461
y = -0.037x + 4.7203<br>R^2 = 0.9934						$t_{1/2}$ = 0.31 h	
**20m**							
0	1525909	1528774	1527342	100	4.605	195	190
15	1244885	1256119	1250502	82	4.405	936	954
30	844782	845571	845177	55	4.013	1761	1800
60	303116	300344	301730	20	2.983	2733	2734
120	33307	33100	33204	2	0.777	3141	3175
y = -0.033x + 4.8397<br>R^2 = 0.9887						$t_{1/2}$ = 0.35 h	
**20n**							
0	3076165	3088562	3082364	100	4.605	NA	

15	3230435	3179949	3205192	103.98488	4.644		
30	3129630	3098401	3114016	101.026874	4.615		
60	3106771	3072783	3089777	100.240513	4.608		
120	3034827	3013070	3023948.5	98.1048634	4.586		
			y = -0.0005x + 4.6402<br>R^2 = 0.8497			stable	
			**20o**				
0	774758	772475	773617	100	4.605	144	140
15	713862	715181	714522	92	4.526	1434	1441
30	396197	396769	396483	51	3.937	2412	2423
60	98358	97574	97966	13	2.539	3279	3256
120	8591	8645	8618	1	0.108	3530	3611
			y = -0.0395x + 4.9222<br>R^2 = 0.9871			$t_{1/2}$ = 0.29 h	
			**20p**				
0	68617	67937	68277	100	4.605	2237	2248
15	1150	1129	1140	1.7	0.512	3232	3218
30	579	611	595	0.87	-0.138	3160	3155
60	485	472	478.5	0.70	-0.356	3307	3316
120	374	395	384.5	0.56	-0.574	3294	3309
						unstable during storage	
			**20q**				
0	1861612	1836931	1849272	100	4.605	226	222
15	1656830	1684590	1670710	90	4.504	648	650
30	1327843	1323274	1325559	72	4.272	1092	1098
60	872386	868134	870260	47	3.851	2033	2032
120	273076	273790	273433	15	2.694	2702	2694
			y = -0.0163x + 4.7184<br>R^2 = 0.9865			$t_{1/2}$ = 0.65 h	
			**20r**				
0	38371	39209	38790	100	4.605	131	131
15	44540	44635	44588	115	4.744	156	156
30	45990	45780	45885	118	4.773	162	170
60	44898	43896	44397	114	4.740	180	183
120	44510	45170	44840	116	4.750	229	233
			y = 0.0007x + 4.6916<br>R^2 = 0.2385			stable	
			**20s**				
0	1861299	1855218	1858259	100	4.605	9	7
15	1853503	1844948	1849226	112	4.721	27	26
30	1918844	1921781	1920313	112	4.718	56	57
60	1826314	1816639	1821477	110	4.698	105	105

120	1893322	1905643	1899483	102	4.627	239	237
y = -0.0003x + 4.6203<br>R^2 = 0.6226						$t_{1/2}$ = > 24 h	
**20t**							
0	2560891	2513711	2537301	100	4.605	165	179
15	1812233	1797862	1805048	71	4.265	1090	1099
30	1298267	1310723	1304495	51	3.940	2004	1965
60	577124	580321	578723	23	3.127	2869	2844
120	109802	109588	109695	4	1.464	3588	3569
y = -0.0264x + 4.668<br>R^2 = 0.9982						$t_{1/2}$ = 0.44 h	
**20u**							
0	857516	843473	850495	100	4.605	546	509
15	872045	884334	878190	103	4.637	1005	982
30	742672	746147	744410	88	4.472	1490	1474
60	444650	439983	442317	52	3.951	2262	2270
120	144850	146816	145833	17	2.842	3094	3031
y = -0.0157x + 4.8066<br>R^2 = 0.9661						$t_{1/2}$ = 0.74 h	
**20v**							
0	576370	570137	573253.5	100	4.605		
15	586448	583294	584871	102.02659	4.625		
30	593543	578708	586125.5	102.245429	4.627	NA	
60	614389	606582	610485.5	106.494858	4.668		
120	583214	592141	587677.5	102.516164	4.630		
						stable	

Plasma Stability of Heterocyclic Class at 37 °C

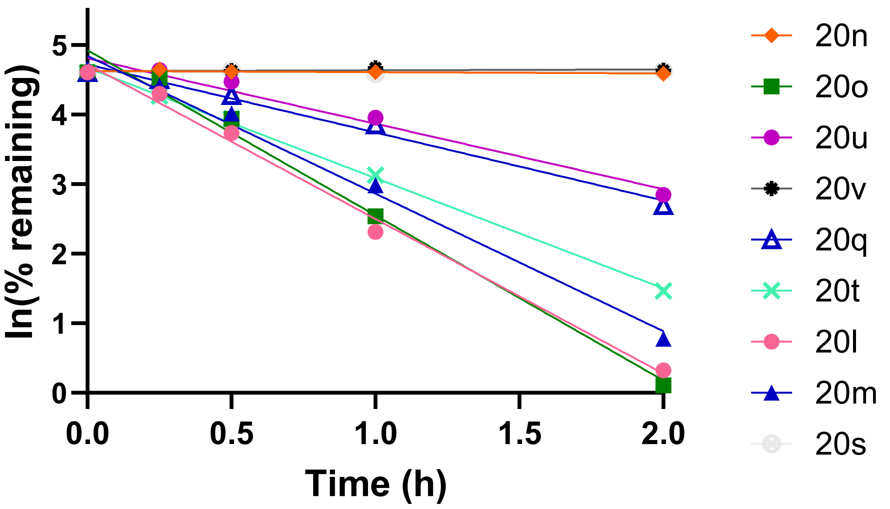

Figure S11: Stability of heterocyclic control and angle strain design progesterone prodrugs over time in plasma.

Table S13: Stability of Nucleophile Design Class in Plasma at 37 °C

Time (min)	AUC @ 254 nm		Average AUC	Remaining (%)	ln(Prodrug %Remaining)	Oxime Formation (nM)	
	A	B				A	B
**20y**							
0	545868	572430	559149	100	4.605	NA	NA
30	504698	532778	518738	93	4.530		
60	518588	522010	520299	93	4.533		
120	471680	472870	472275	84	4.436		
y = -0.0013x + 4.5945<br>R^2 = 0.9278						$t_{1/2}$ = 8.88 h	
**20z**							
0	606800	593751	600276	100	4.605	42	42
15	725935	720335	723135	120	4.791	187	185
30	706626	689860	698243	116	4.756	431	425
60	550921	561104	556013	93	4.529	930	922
120	426858	429139	427999	71	4.267	1904	1913
y = -0.005x + 4.8113<br>R^2 = 0.9629						$t_{1/2}$ = 2.30 h	

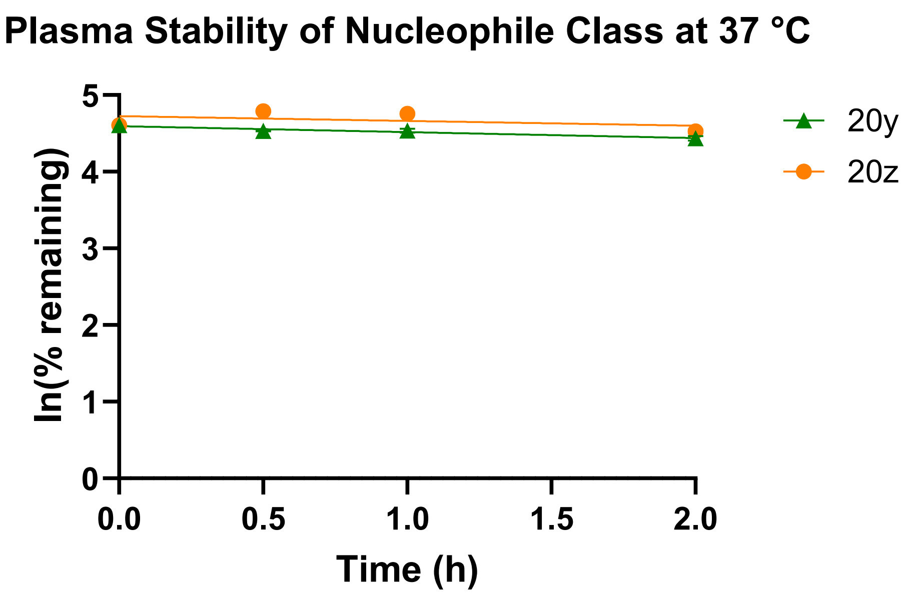

Figure S12: Stability of nucleophile design progesterone prodrugs over time in plasma.

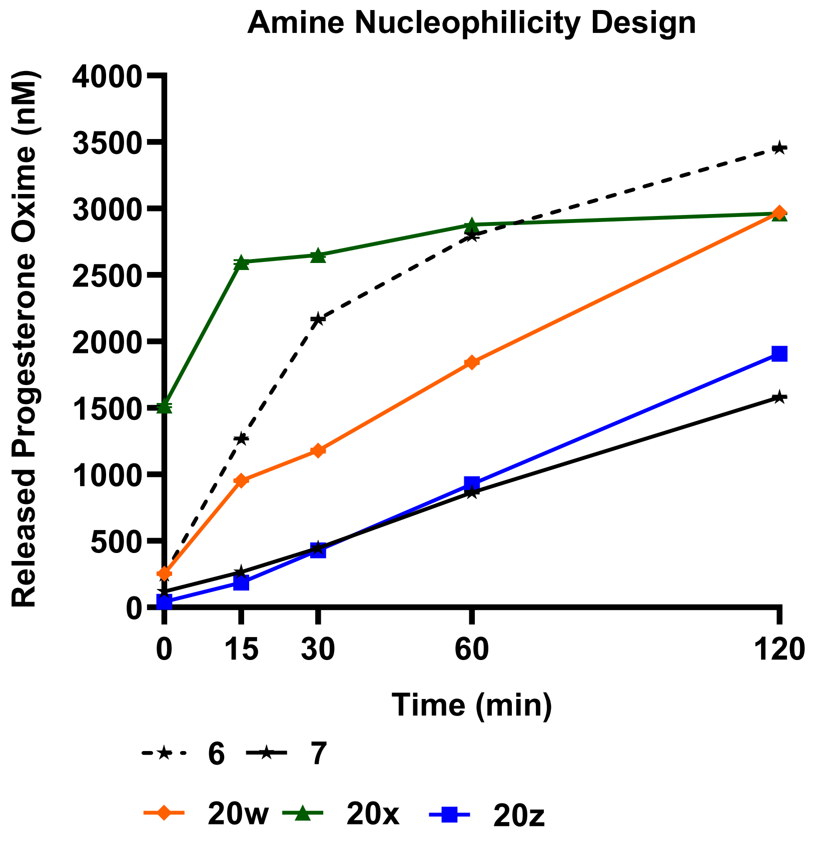

Figure S13: Formation of progesterone oxime **3** from prodrugs in plasma over time.

Amine Nucleophile Linker Design Trends

By incorporating symmetrical amine tethers as demonstrated in **20w–y**, a reactive confirmation is sustained. The primary amines in **20x** allows for more rapid cleavage to parent oxime **3** compared to **20a** (see above figure). However, the increased nucleophilicity in **20x** is detrimental in terms of stability, showing degradation to **3** at time zero. On the other hand, the presence of the tertiary amine tethers in **20y** led to poor nucleophilic cyclization towards **3** in plasma. Moreover, in PBS **20y** suffered from hydrolytic instability (not forming **3**) as well as inadequate aqueous solubility. The addition of the ethylene hydroxyl groups in **20z** revealed a similar cleavage profile in both PBS ($t_{1/2}$ = 6.27 h) and plasma ($t_{1/2}$ = 2.30 h; see also Figure 3C) compared to the *N*-methyl containing **20a**. The mild electron withdrawing effect of the hydroxyl group could shift the equilibrium towards the free base by reducing the amine pK_a but simultaneously oppose cyclization by lowering the nucleophilicity and induce steric hinderance through the tethers.

Table S14: Stability of Steric Control Class in PBS at 37 °C

Time (min)	Response A	Response B	Average Resp.	Remaining (%)	ln(Prodrug %Remaining)
**20a**					
0	965807	966740	966274	100	4.605
15	788930	786844	787887	82	4.401
30	838393	841956	840175	87	4.465
60	824151	814349	819250	85	4.440
120	740936	750577	745757	77	4.346
240	632553	627545	630049	65	4.178
y = -0.0014x + 4.5168<br>R^2 = 0.8316				$t_{1/2}$ = 8.07 h	
**20b**					
0	513252	499257	506255	100	4.605
15	426557	421162	423860	84	4.428
30	425812	424352	425082	84	4.430
60	380191	379293	379742	75	4.318
120	346931	342931	344931	68	4.221
240	253814	252799	253307	50	3.913
y = -0.0026x + 4.518<br>R^2 = 0.9544				$t_{1/2}$ = 4.50 h	
**20c**					
0	2716319	2708643	2712481	100	4.605
15	2362073	2336359	2349216	87	4.461
30	2264722	2264196	2264459	83	4.425

60	2024682	2029380	2027031	75	4.314
120	1452332	1449278	1450805	53	3.979
240	1154697	1142477	1148587	42	3.746
$y = -0.0035x + 4.5285$ $R^2 = 0.9512$				$t_{1/2} = 3.27$ h	
**20d**					
0	49626	52003	50815	100	4.605
15	45850	46527	46189	91	4.510
30	40237	40013	40125	79	4.369
60	39002	38843	38923	77	4.339
120	22104	22288	22196	44	3.777
240	15774	15934	15854	31	3.440
$y = -0.005x + 4.5592$ $R^2 = 0.9524$				$t_{1/2} = 2.32$ h	
**20e**					
0	77299	75169	76234	100	4.605
15	62454	62491	62473	82	4.406
30	40092	39250	39671	52	3.952
60	33123	33021	33072	43	3.770
120	26121	25711	25916	34	3.526
240	13255	13554	13405	18	2.867
$y = -0.0066x + 4.3691$ $R^2 = 0.9123$				$t_{1/2} = 1.74$ h	
**20f**					
0	1736843	1734924	1735884	100	4.605
15	1275825	1279913	1277869	74	4.299
30	1331730	1330177	1330954	77	4.340
60	1315166	1307589	1311378	76	4.325
120	956042	949869	952956	55	4.005
240	918950	911871	915411	53	3.965
$y = -0.0023x + 4.4333$ $R^2 = 0.7486$				$t_{1/2} = 5.07$ h	

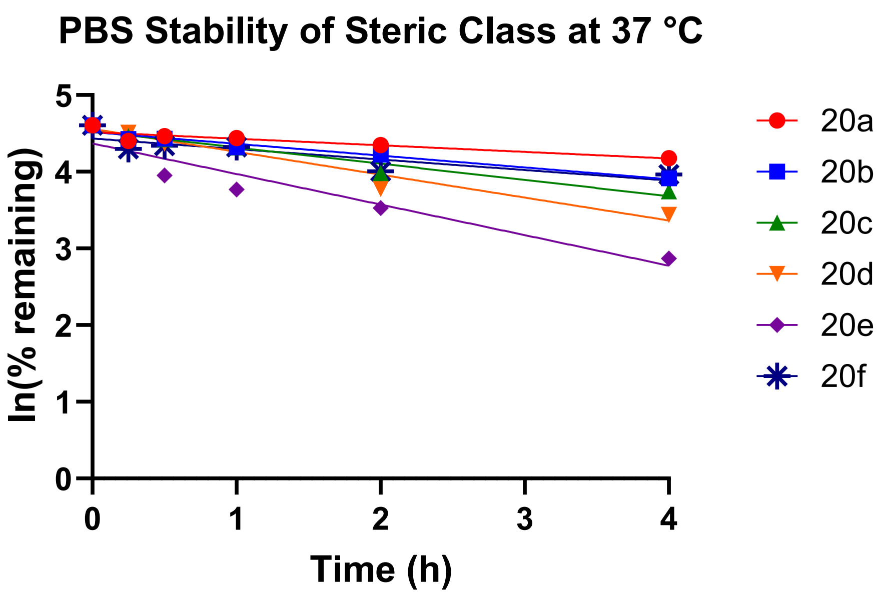

Figure S14: Stability of steric design progesterone prodrugs over time in PBS buffer.

Table S15: Stability of Electronic Control Class in PBS at 37 °C

Time (min)	Response A	Response B	Average Resp.	Remaining (%)	ln(Prodrug %Remaining)
**20i**					
0	102036	102531	102284	100	4.605
15	88577	89618	89098	87	4.467
30	91414	90665	91040	89	4.489
60	94754	94033	94394	92	4.525
120	78948	77883	78416	77	4.339
240	58791	58403	58597	57	4.048
	$y = -0.0021x + 4.5765$<br>$R^2 = 0.9297$			$t_{1/2} = 5.45$ h	
**20k**					
0	354566	358594	356580	100	4.605
15	327004	327763	327384	92	4.520
30	313555	314844	314200	88	4.479
60	284757	285792	285275	80	4.382
120	246695	249052	247874	70	4.242
240	221163	219481	220322	62	4.124
	$y = -0.0019x + 4.5412$<br>$R^2 = 0.9221$			$t_{1/2} = 5.99$ h	

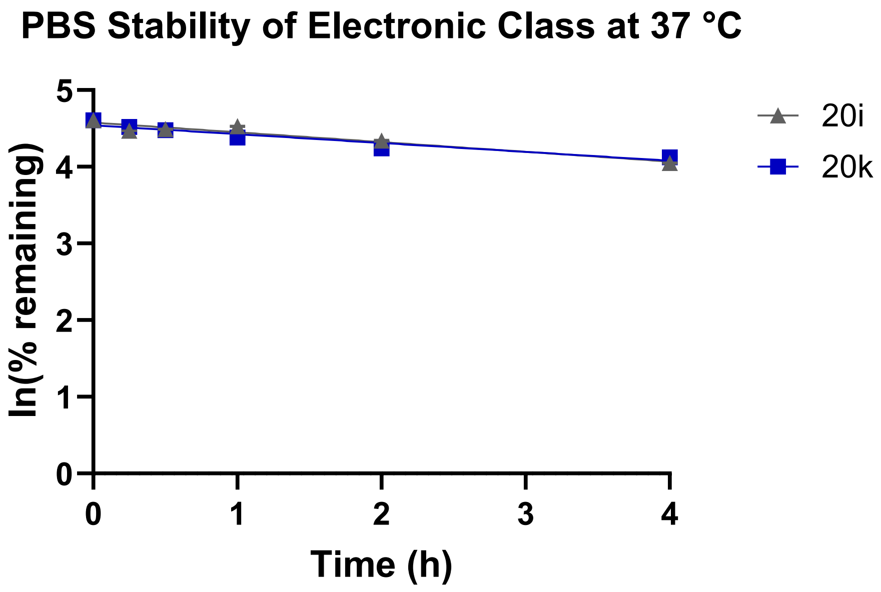

Figure S15: Stability of electronic design progesterone prodrugs over time in PBS buffer.

Table S16: Stability of Heterocyclic Control & Angle Strain Class in PBS at 37 °C

Time (min)	Response A	Response B	Average Resp.	Remaining (%)	ln(Prodrug %Remaining)
**20l**					
0	1507600	1495743	1501672	100	4.605
15	932701	934566	933634	62	4.130
30	659114	660073	659594	44	3.782
60	349385	352119	350752	23	3.151
120	115682	114447	115065	8	2.036
240	32475	32573	32524	2	0.773
y = -0.0157x + 4.2943 R^2 = 0.9667				$t_{1/2}$ = 0.74 h	
**20m**					
0	1456447	1470621	1463534	100	4.605
15	946766	948363	947565	65	4.170
30	773440	780851	777146	53	3.972
60	443500	440282	441891	30	3.408
120	167127	165350	166239	11	2.430
240	42526	43510	43018	3	1.078
y = -0.0144x + 4.3961 R^2 = 0.9843				$t_{1/2}$ = 0.80 h	
**20n**					
0	1475140	1405415	1440278	100	4.605
15	812378	839446	825912	57	4.049

30	1052005	1209613	1130809	79	4.363
60	1165459	1131502	1148481	80	4.379
120	1077323	1151278	1114301	77	4.349
240	1001116	1026806	1013961	70	4.254
	y = -0.0004x + 4.3669<br>R^2 = 0.0471			$t_{1/2}$ = 26.51 h	
		**20o**			
0	706807	700943	703875	100	4.605
15	514419	505582	510001	72	4.283
30	368212	367770	367991	52	3.957
60	210787	210274	210531	30	3.398
120	101166	101144	101155	14	2.665
240	29937	29862	29900	4	1.446
	y = -0.0129x + 4.3899<br>R^2 = 0.9771			$t_{1/2}$ = 0.90 h	
		**20p**			
0	190364	193490	191927	100.00	4.605
15	7763	7618	7691	4.01	1.388
30	1510	1535	1523	0.79	-0.232
60	944	912	928	0.48	-0.727
120	524	541	533	0.28	-1.282
240	303	332	318	0.17	-1.799
	y = -0.0188x + 1.7862<br>R^2 = 0.5168			$t_{1/2}$ = 0.61 h	
		**20q**			
0	1595103	1613065	1604084	100	4.605
15	936250	944066	940158	59	4.071
30	632846	629560	631203	39	3.672
60	320906	316218	318562	20	2.989
120	77102	75729	76416	5	1.561
240	29008	28758	28883	2	0.588
	y = -0.0166x + 4.2023<br>R^2 = 0.9349			$t_{1/2}$ = 0.70 h	
		**20r**			
0	29428	29768	29598	100	4.605
15	25166	25618	25392	86	4.452
30	25357	25285	25321	86	4.449
60	23638	24051	23845	81	4.389
120	20924	21949	21437	72	4.283
240	15739	15781	15760	53	3.975
	y = -0.0023x + 4.5404<br>R^2 = 0.9648			$t_{1/2}$ = 4.93 h	
		**20s**			

0	1571756	1576724	1574240	100	4.605
15	1507792	1500583	1504188	96	4.560
30	1622768	1623425	1623097	103	4.636
60	1661302	1668112	1664707	106	4.661
120	1469369	1469791	1469580	93	4.536
240	1363583	1370392	1366988	87	4.464
	y = -0.0006x + 4.6255<br>R^2 = 0.6115			$t_{1/2}$ = 18.47 h	
**20t**					
0	2274759	2285969	2280364	100	4.605
15	1580355	1577562	1578959	69	4.238
30	1425081	1415496	1420289	62	4.132
60	929682	928762	929222	41	3.707
120	323008	321114	322061	14	2.648
240	120781	120368	120575	5	1.665
	y = -0.0123x + 4.4511<br>R^2 = 0.9743			$t_{1/2}$ = 0.94 h	
**20u**					
0	65494	66460	65977	100	4.605
15	47514	47835	47675	72	4.280
30	46664	49839	48252	73	4.292
60	28931	28095	28513	43	3.766
120	13695	13346	13521	20	3.020
240	5127	4893	5010	8	2.027
	y = -0.0107x + 4.4965<br>R^2 = 0.9826			$t_{1/2}$ = 1.08 h	
**20v**					
0	451513	454565	453039	100	4.605
15	324795	320338	322566.5	71	4.266
30	319203	307333	313268	69	4.236
60	325120	329785	327452.5	72	4.281
120	315892	314118	315005	70	4.242
	y = -0.0021x + 4.421<br>R^2 = 0.402			$t_{1/2}$ = 6.34 h	

PBS Stability of Heterocyclic Class at 37 °C

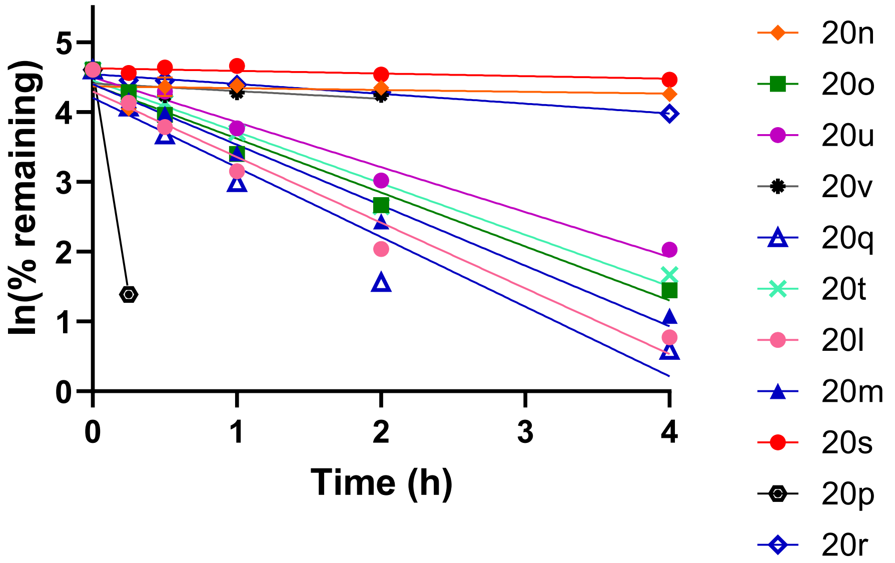

Figure S16: Stability of heterocyclic and angle strain design progesterone prodrugs over time in PBS buffer.

Table S17: Stability of Nucleophile Design Control Class in PBS at 37 °C

Time (min)	AUC @ 254 nm A	AUC @ 254 nm B	Average AUC	Remaining (%)	ln(Prodrug %Remaining)
**20y**					
0	81860	80654	81257	100	4.605
15	62771	56103	59437	73	4.292
30	53688	53857	53773	66	4.192
60	43874	43466	43670	54	3.984
120	26900	30284	28592	35	3.561
240	16698	16846	16772	21	3.027
	$y = -0.0062x + 4.4214$ $R^2 = 0.9605$			$t_{1/2} = 1.87$ h	
**20z**					
0	676801	670208	673505	100	4.605
15	658717	656735	657726	98	4.581
30	636114	634981	635548	94	4.547
60	654055	651147	652601	97	4.574
120	531262	522520	526891	78	4.360
240	439939	438976	439458	65	4.178
	$y = -0.0018x + 4.6171$ $R^2 = 0.9568$			$t_{1/2} = 6.27$ h	

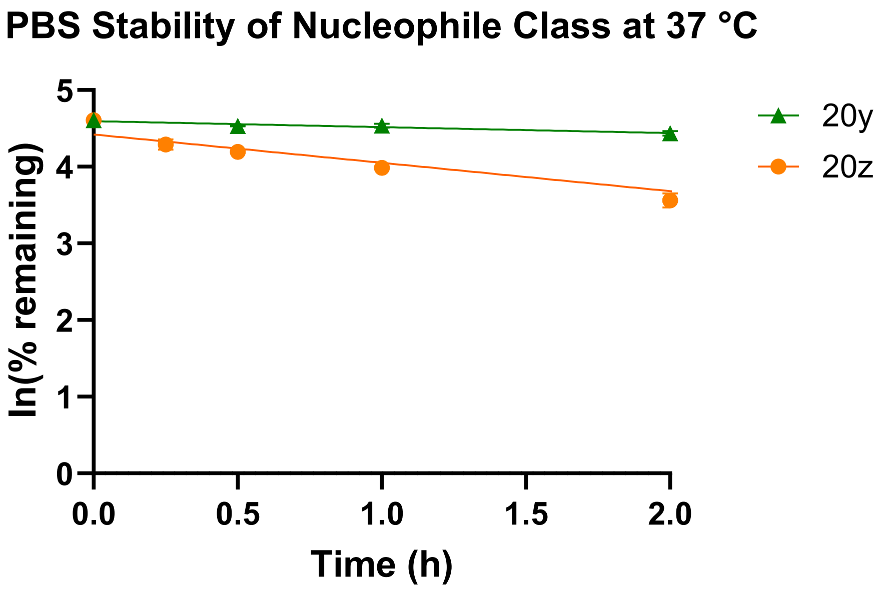

Figure S17: Stability of nucleophile design progesterone prodrugs over time in PBS buffer.

Stability Comparison in Human, Rat and Mouse Plasma of Selected Progesterone Prodrugs

Table S18: Stability of Selected Prodrugs in Human Plasma

Time (min)	Response		Average Resp.	Remaining (%)	ln(Prodrug %Remaining)	Oxime Formation (AUC)	
	A	B				A	B
**20a**							
0	28939	29550	29244.5	100	4.605	6260	6213
15	27186	28064	27625	94	4.548	1620	1614
30	27236	27960	27598	94	4.547	3708	3817
60	21532	21426	21479	73	4.297	7628	7794
120	19335	19329	19332	66	4.191	16981	17106
$y = -0.0037x + 4.6032$ $R^2 = 0.9156$						$t_{1/2}$ = 3.14 h	
**20b**							
0	761356	679493	720425	100	4.605	2380	2407
15	565904	545602	555753	77	4.346	25748	26022
30	524609	511582	518096	72	4.275	59422	59853
60	429154	427205	428180	59	4.085	131927	131537
120	226598	225161	225880	31	3.445	226462	226874
$y = -0.0091x + 4.5616$ $R^2 = 0.9819$						$t_{1/2}$ = 1.27 h	
**20l**							
0	1436691	1427428	1432060	100	4.605	20385	20045

15	1048480	1056631	1052556	73	4.297	107557	107010
30	597192	598907	598050	42	3.732	184301	183577
60	144679	144429	144554	10	2.312	241812	244404
120	19861	19626	19743.5	1	0.321	279189	278252
y = -0.037x + 4.7203<br>R^2 = 0.9934						$t_{1/2}$ = 0.31 h	
**20t**							
0	2560891	2513711	2537301	100	4.605	13515	13543
15	1812233	1797862	1805048	71	4.265	78211	79412
30	1298267	1310723	1304495	51	3.940	143039	146398
60	577124	580321	578723	23	3.127	231097	231958
120	109802	109588	109695	4	1.464	289537	286555
y = -0.0264x + 4.668<br>R^2 = 0.9982						$t_{1/2}$ = 0.44 h	

Table S19: Stability of Selected Prodrugs in Rat Plasma

Time (min)	Response		Average Resp.	Remaining (%)	ln(Prodrug %Remaining)	Oxime Formation (AUC)	
	A	B				A	B
**20a**							
0	25508	25306	25407	100	4.605	305	309
15	25076	26032	25554	101	4.611	971	965
30	23889	24234	24061.5	95	4.551	2288	2303
60	22557	21936	22246.5	88	4.472	5826	5704
120	18792	18362	18577	73	4.292	12385	12275
y = -0.003x + 4.6491<br>R^2 = 0.9978						$t_{1/2}$ = 3.88 h	
**20b**							
0	572883	575370	574126.5	100	4.605	6054	5982
15	603982	611103	607542.5	106	4.662	25199	25274
30	586607	580394	583500.5	102	4.621	53452	53981
60	520329	518432	519380.5	90	4.505	121626	122404
120	384908	388281	386594.5	67	4.210	257378	259162
y = -0.005x + 4.8113<br>R^2 = 0.9629						$t_{1/2}$ = 2.30 h	
**20l**							
0	353357	353188	353272.5	100	4.605	3082	3144
15	321291	321414	321352.5	91	4.510	7530	7614
30	247350	246123	246736.5	70	4.246	12631	12671
60	108828	109039	108933.5	31	3.429	20643	20693
120	12245	12387	12316	3	1.249	27009	27258
y = -0.029x + 4.9136						$t_{1/2}$ = 0.40 h	

R² = 0.9697							
**20t**							
0	2769686	2797135	2783411	100	4.605	33996	33807
15	2640425	2640941	2640683	95	4.553	128565	130268
30	2069295	2078946	2074121	75	4.311	253334	257183
60	977694	979496	978595	35	3.560	437694	439137
120	157761	156581	157171	6	1.731	580799	581853
y = -0.0225x + 4.8782<br>R² = 0.9716						$t_{1/2}$ = 0.46 h	

Table S20: Stability of Selected Prodrugs in Mouse Plasma

Time (min)	Response		Average Resp.	Remaining (%)	ln(Prodrug %Remaining)	Oxime Formation (AUC)	
	A	B				A	B
**20a**							
0	27463	27939	27701	100.00	4.605	5822	5901
15	27295	27952	27623.5	99.72	4.602	1122	1093
30	27253	27066	27159.5	98.05	4.585	2334	2368
60	23427	23968	23697.5	85.55	4.449	4994	5190
120	21367	20980	21173.5	76.44	4.336	10857	10701
y = -0.0025x + 4.6263<br>R² = 0.9543						$t_{1/2}$ = 4.70 h	
**20b**							
0	671480	671095	671287.5	100.00	4.605	4829	4787
15	687104	674312	680708	101.40	4.619	19655	19445
30	658993	649067	654030	97.43	4.579	38936	38469
60	614960	626644	620802	92.48	4.527	85583	85227
120	533529	522042	527785.5	78.62	4.365	176098	176289
y = -0.0021x + 4.6352<br>R² = 0.9576						$t_{1/2}$ = 5.40 h	
**20l**							
0	435988	431656	433822	100.00	4.605	2004	1996
15	191772	190266	191019	44.03	3.785	17713	17986
30	64593	64832	64712.5	14.92	2.702	24498	24795
60	7532	7584	7558	1.74	0.555	27859	28251
120	866	833	849.5	0.20	-1.631	30529	30606
y = -0.029x + 4.9136<br>R² = 0.9697						$t_{1/2}$ = 0.40 h	
**20t**							
0	3797576	3725827	3761702	100.00	4.605	26065	25664
15	2573482	2522928	2548205	67.74	4.216	262774	258885
30	1522731	1519002	1520867	40.43	3.700	421322	425344

60	548984	544734	546859	14.54	2.677	582897	579045
120	71753	69908	70830.5	1.88	0.633	645027	640521
$y = -0.0335x + 4.6739$<br>$R^2 = 0.9992$						$t_{1/2} = 0.34$ h	

Stability Results of Allopregnanolone Prodrugs in Human Plasma and PBS Buffer

Table S21: Stability of Allopregnanolone Prodrugs in Human Plasma at 37 °C

Time (min)	Response A	Response B	Average Resp.	Remaining (%)	ln(Prodrug %Remaining)	Oxime Formation (nM) A	Oxime Formation (nM) B
**21a**							
0	974687.5	973421.5	974055	100	4.605	41	43
15	844791.5	866440	855616	88	4.476	369	375
30	680570.5	675753	678162	70	4.243	908	658
60	422306	407474	414890	43	3.752	1268	1216
120	131561	130779	131170	13	2.600	1914	1826
240	9131	9646.5	9389	1	-0.037	2202	2248
$y = -0.0171x + 4.7044$<br>$R^2 = 0.9919$						$t_{1/2} = 0.59$	
**21b**							
0	371191	361132.5	366162	100	4.605	68	77
15	307700	312671.5	310186	85	4.439	360	393
30	245233	261764.5	253499	69	4.237	685	762
60	165104.5	169446.5	167276	46	3.822	1271	1363
120	58080.5	56461	57271	16	2.750	1986	**
240	9369.5	8428	8899	2	0.888	2230	2213
$y = -0.0028x + 4.5169$<br>$R^2 = 0.8795$						$t_{1/2} = 0.73$	
**21c**							
0	1399126	1422515	1410821	100	4.605	70	75
15	1122541	1191105.5	1156823	82	4.407	466	476
30	874768.5	879079.5	876924	62	4.130	950	940
60	441085.5	447049.5	444068	31	3.449	1553	1587
120	81521	87113.5	84317	6	1.788	1980	2139
240	7192	7370.5	7281	1	-0.661	2181	2203
$y = -0.024x + 4.7535$<br>$R^2 = 0.9891$						$t_{1/2} = 0.51$	
**21d**							
0	796430	764919	780675	100	4.605	41	61
15	663466.5	646457	654962	84	4.430	471	453
30	530406	503649.5	517028	66	4.193	908	802

60	334988.5	332010.5	333500	43	3.755	1508	1550
120	124671.5	131794.5	128233	16	2.799	2149	2465
240	20557	20851.5	20704	3	0.975	2623	2764
y = -0.0153x + 4.6427 R^2 = 0.9997						$t_{1/2}$ = 0.76	
**21i**							
0	44125.5	43885.5	44006	100	4.605	211	237
15	40773	40467.5	40620	92	4.525	565	579
30	38739.5	38291	38515	88	4.472	576	936
60	30059	31514	30787	70	4.248	1522	1652
120	16315.5	13430.5	14873	34	3.520	2178	2376
240	4594.5	4700.5	4648	11	2.357	3164	3024
y = -0.0097x + 4.7047 R^2 = 0.992						$t_{1/2}$ = 1.19	
**21k**							
0	111493	107817.5	109655	100	4.605	80	102
15	67997	68841.5	68419	62	4.133	431	451
30	78711.5	52672.5	65692	60	4.093	811	793
60	38170	36512.5	37341	34	3.528	1373	1373
120	15846.5	15514.5	15681	14	2.660	2052	2043
240	3185.5	3273	3229	3	1.080	2620	2464
y = -0.0143x + 4.4601 R^2 = 0.9937						$t_{1/2}$ = 0.81	
**21l**							
0	1430382	1399908.5	1415145.25	100	4.605	309	344
15	479810.5	455979.5	467895	33	3.498	1451	1480
30	128875.5	128188	128531.75	9	2.206	1849	1845
60	31958.5	32161	32059.75	2	0.818	1961	2039
120	27277.5	25654.5	26466	2	0.626	2005	1848
240	26202.5	25905.5	26054	2	0.610	2013	1997
y = -0.014x + 3.1443 R^2 = 0.5574						$t_{1/2}$ = 0.36	

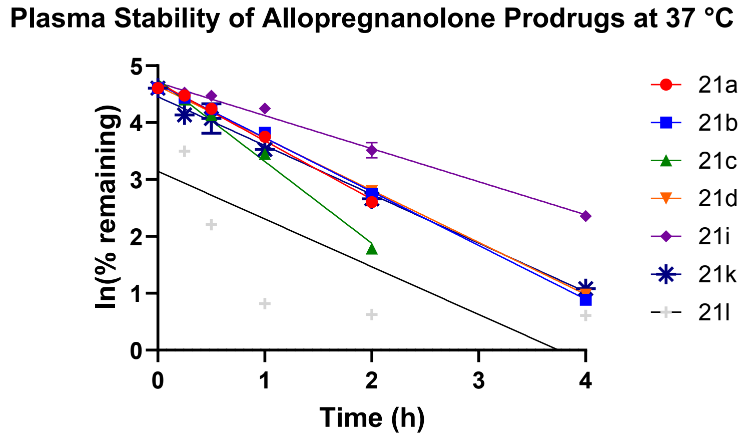

Figure S18: Stability of allopregnanolone prodrugs over time in human plasma.

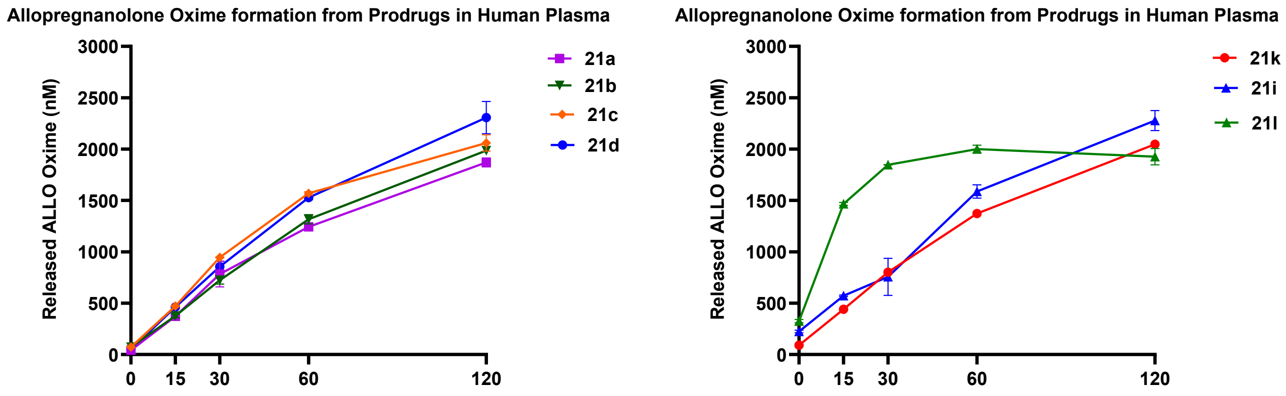

Figure S19: Formation of allopregnanolone oxime from prodrugs over time in human plasma.

Table S22: Stability of Allopregnanolone Prodrugs in PBS at 37 °C

Time (min)	Response A	Response B	Average Resp.	Remaining (%)	ln(Prodrug %Remaining)
**21a**					
0	966999	959857	963428	100	4.605
15	910394	915343	774216	80	4.387
30	837445	844766	912869	95	4.551
60	856095	843750	841106	87	4.469
120	777923	770508	849923	88	4.480
240	696632	703962	700297	73	4.286
	y = -0.0009x + 4.5365<br>R² = 0.5585			$t_{1/2}$ = 12.19	
**21b**					
0	434132	439833	436983	100	4.605
15	332288	330612	331450	76	4.329

30	365650	359594	362622	83	4.419
60	337538	340055	338797	78	4.351
120	320683	318401	319542	73	4.292
240	190332	196568	193450	44	3.790
$y = -0.0028x + 4.5169$<br>$R^2 = 0.8795$				$t_{1/2} = 4.08$	
**21c**					
0	1216590	1223199	1219895	100	4.605
15	976802	964969	970886	80	4.377
30	961946	978013	969980	80	4.376
60	879421	858953	869187	71	4.266
120	827566	824524	826045	68	4.215
240	518958	519635	519297	43	3.751
$y = -0.0031x + 4.5018$<br>$R^2 = 0.9323$				$t_{1/2} = 3.78$	
**21d**					
0	1009478	1004099	1006789	100	4.605
30	598241	599455	598848	59	4.086
60	522954	531019	526987	52	3.958
120	387934	385352	386643	38	3.648
240	168686	168340	168513	17	2.818
$y = -0.0068x + 4.4385$<br>$R^2 = 0.9679$				$t_{1/2} = 1.69$	
**21i**					
0	44132	44668	44400	100	4.605
15	37090	37010	37050	83	4.424
30	32033	31829	31931	72	4.276
60	34100	34703	34402	77	4.350
120	29172	28948	29060	65	4.181
240	23268	21793	22531	51	3.927
$y = -0.0024x + 4.4782$<br>$R^2 = 0.87$				$t_{1/2} = 4.86$	
**21k**					
0	59800	62016	60908	100	4.605
15	43055	43712	43384	71	4.266
30	41770	48852	45311	74	4.309
60	39748	39213	39481	65	4.172
120	38262	37820	38041	62	4.134
240	26217	26201	26209	43	3.762
$y = -0.0028x + 4.4256$<br>$R^2 = 0.8499$				$t_{1/2} = 4.12$	
**21l**					

0	1066385	**	1066385	100	4.605
15	779190	770652	774921	73	4.286
30	827231	826185	826708	78	4.351
60	381363	382239	381801	36	3.578
120	171406	170103	170755	16	2.773
240	42191	41785	41988	4	1.371
y = -0.0123x + 5.0231<br>R^2 = 0.9457				$t_{1/2}$ = 0.94	

PBS Stability of Allopregnanolone Prodrugs at 37 °C

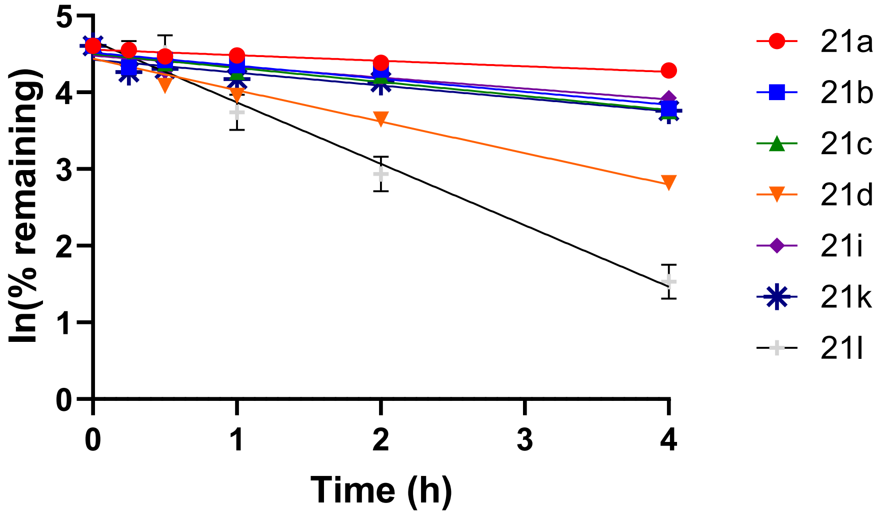

Figure S20: Stability of allopregnanolone prodrugs over time in PBS buffer.

2. Aqueous Solubility

High aqueous solubility was determined by visual inspection of 5 mg solid material in 0.5 mL sodium acetate buffer solution. This media was selected to ensure stability of prodrugs during solubility assessment. Most prodrugs met this solubility criteria (10 mg/mL) with some reaching dissolution in 50 mg/mL aqueous media. Prodrugs **20r** and **20y** demonstrated lower solubility than the prerequisite < 10mg/mL, whereas the poor stability of **20p** and **20x** in aqueous media and subsequent release of insoluble oxime species jeopardized the aqueous solubility. Overall, the allopregnanolone prodrugs demonstrated lower solubility compared to their progesterone counterparts.

3. Summary of Stability Data

Table S23: Prodrug Stability Data *In Vitro*

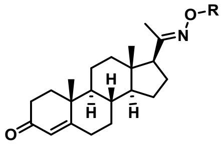

20

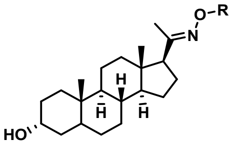

21

R	#	Solubility pH 5.0, rt (mg/mL)	$t_{½}$: pH 5.0, rt (days)	$t_{½}$: PBS, rt (h)	PBS, 37 °C		Plasma, 37 °C	
					$t_{½}$ (h)	%rem. at 2h	$t_{½}$ (h)	%rem. at 2h
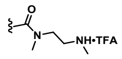	20a	> 10	> 7	46.90	8.07	77	2.45	73
	21a	> 5	ND	ND	12.19	88	0.59	13
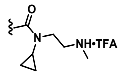	20b	> 10	> 7	28.16	4.50	68	1.27	31
	21b	> 5	ND	ND	4.08	73	0.73	16
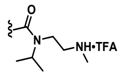	20c	> 10	> 7	19.72	3.27	53	1.59	47
	21c	> 5	ND	ND	3.78	68	0.76	16
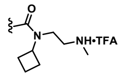	20d	> 10	> 7	11.79	2.32	44	2.67	59
	21d	> 5	ND	ND	1.69	38	0.76	16
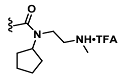	20e	> 10	> 7	10.70	1.74	34	4.02	76
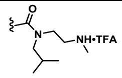	20f	> 10	> 7	> 24	5.07	55	4.78	95
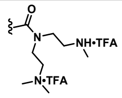	20g	< 10	3.16	2.17	NA	NA	NA	NA
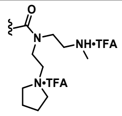	20h	< 10 (degrades)	4.38	7.23	NA	NA	NA	NA
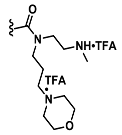	20i	> 10	> 7	31.16	5.45	77	2.09	57
	21i	> 5	ND	ND	4.86	65	1.19	34
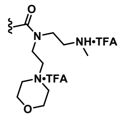	20j	> 10	4.14	21.77	NA	NA	NA	NA
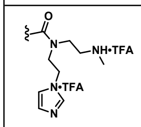	20k	> 10	> 7	47.00	5.99	70	3.16	68
	21k	> 5	ND	ND	4.12	62	0.81	14
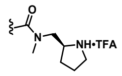	20l	> 10	7.29	1.90	0.74	8	0.31	1
	21l	> 5	ND	ND	0.94	16	0.36	2
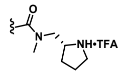	20m	> 10	> 7	6.15	0.80	11	0.35	2
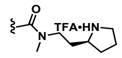	20n	> 10	> 7	> 24	26.50	77	>24	stable

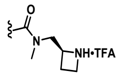	20o	> 10	> 7	6.30	0.90	14	0.29	1
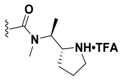	20p	NA	NA	NA	NA	NA	NA	NA
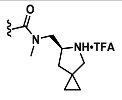	20q	> 10	> 7	4.35	0.70	5	0.65	15
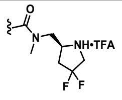	20r	<< 10	ND	ND	4.93	72	>24	stable
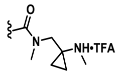	20s	> 10	ND	ND	18.47	93	>24	stable
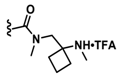	20t	> 10	> 7	10.49	0.94	14	0.44	4
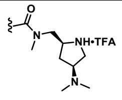	20u	> 10	3.00	7.53	1.08	20	0.74	17
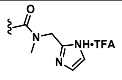	20v	<< 10	> 7	> 7	6.34	70	> 24	stable
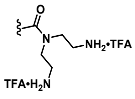	20w	> 10	> 7	10.13	NA	NA	NA	NA
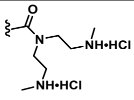	20x	< 10 (degrades)	> 7	1.51	NA	NA	NA	NA
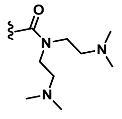	20y	<<10	7.93 min	0.02	1.87	35	8.88	84
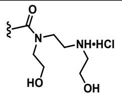	20z	> 10	> 7	28.79	6.27	78	2.30	71

4. Computational Studies

4.1 Computational Methodology

All calculations were run using the Schrödinger suite of programs (2025-1) through the Maestro graphical interface. To determine the minimum energy conformations of synthesized sterically controlled compounds, aldoximes were drawn connected to protonated self-immolative promoieties.

Rapid Torsional Scan.[1] To investigate the rotational barrier of *anti*/*syn* conformational switching, the carbamate C-N bond was rotated every 10 degrees prior to molecular mechanics minimization with water generalized Born/surface area (GB/SA) continuum implicit solvation model.

Conformational Search.[2, 3] Without further molecular mechanic minimization, mixed torsional/low-mode sampling conformational search was initiated for all structures within Macromodel, using OPLS4 force field, water GB/SA implicit solvation model. For each search, 100 torsional steps were taken per rotatable bond, conformations were minimized until the gradient was <0.05 kJÅ-1mol-1 using the PRCG (Polak-Ribier Conjugate Gradient) algorithm (2,00 maximum iterations), and mirror-image conformations were retained.

Molecular Mechanics Minimization.[2] Outputs were further subjected to minimization with TNCG (Truncated Newton Conjugate Gradient) algorithm until the gradient was <0.01 kJÅ-1mol-1 (10000 maximum iterations) and FMNR (Full Matrix Newton Raphson) algorithm until the gradient was <0.001 kJÅ-1mol-1 (1000 maximum iterations), respectively, before comparison of all atoms and elimination of redundant conformers using a maximum atom deviation cutoff of 0.5 Å.

Density Function Theory (DFT) Optimization.[4] Remaining conformations were optimized with DFT calculations using B3LYP-D3/6-31G** level of theory in a spherical coordinate system using Jaguar in gas phase, then using water c-PCM implicit solvation model to determine solvation energies. For the lowest energy *anti*-conformer and *syn*-conformer, vibrational frequencies were checked with the same level of theory including water c-PCM implicit solvation to ensure that stationary points were true minima on the potential energy surface.

ML pK_a Prediction.[5] Epik machine learning model was applied to drawn compounds within a pH range of 8.0 ± 2.5.

Table S24: Comparison of Dal Corso *et al.* Methods and Revised Methods for This Work

	Dal Corso *et al.* (2022)	This Work
Schrodinger Release	2016-1	2025-1
Elimination Product	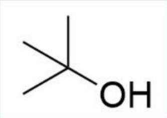 tert-Butanol	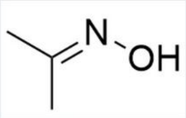 Aldoxime
Conformational Search	OPLS3<br>Monte Carlo/Energy Minimization	OPLS4<br>Mixed Torsional/Low-Mode sampling
MM Minimization	PRCG	PRCG, TNCG, FMNR
QM Optimization (DFT)	B3LYP/6-31G*<br>PBF solvation	B3LYP-D3/6-31G**<br>c-PCM solvation

4.2 Additional Considerations

Upon a change to physiological pH, the dominant protonated *anti*-conformer must convert to the deprotonated *syn*-conformer for the rate-determining addition reaction (**Figure S21A**). Deprotonation can occur following or preceding conformational switching (**Figure S21B**). Prior *in silico* work by Dal Corso *et al.* revealed pK_as ~2 units lower for the *syn*-conformer (B3LYP/6-31G*, PBF)[6] which suggests that deprotonation following conformational switching would be energetically preferred in solution.

To validate that the rotational barrier mediating *anti*/*syn* conformational switching is accessible and negligible at room temperature, molecular mechanics torsional scan was conducted about the carbamate C-N bond of **20e** in increments of 10 degrees (**Figure S21C**). **20e** was selected as it has the greatest steric contribution. The output revealed a torsional barrier under 14 kcal/mol. Experimental data from literature agrees with this, as the C-N bond of carbamates, which exhibits quick *anti*/*syn* interconversion in NMR studies, is seen to have a rotational barrier of approx. 15 kcal/mol.[7, 8] Thus, the kinetic barrier is not rate-limiting, and can be neglected.

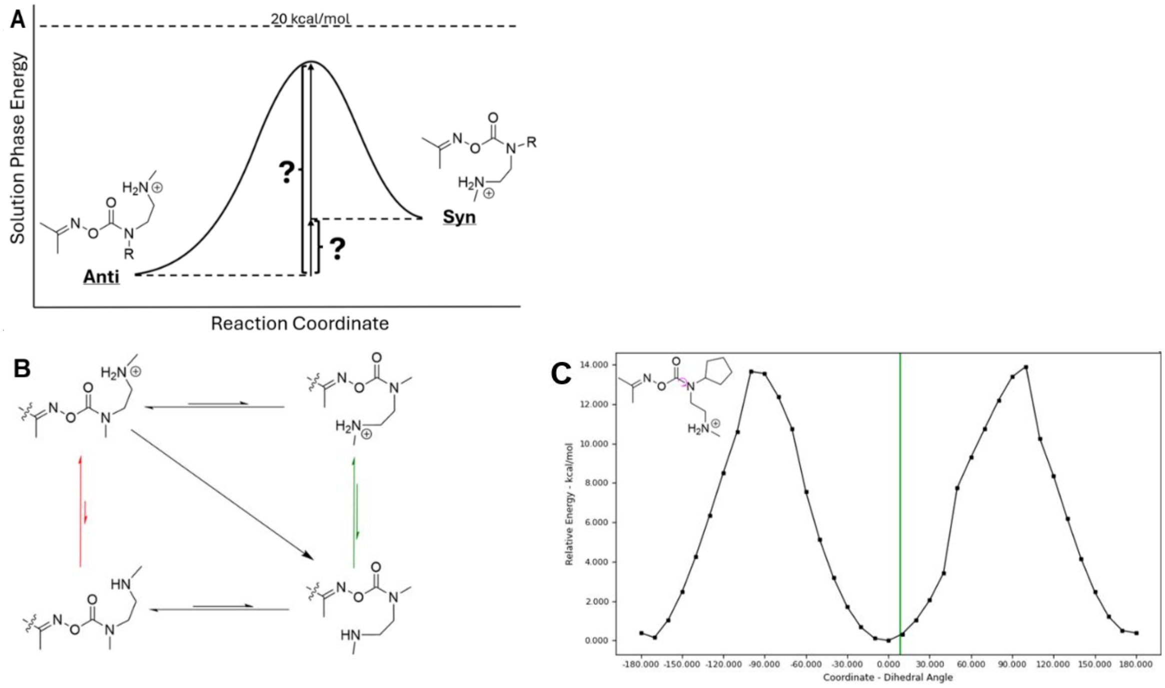

Figure S21: A) Reaction coordinate diagram for *anti*/*syn* conformational switching. B) Avenues of conversion from the dominant protonated *anti*-conformer to the deprotonated *syn*-conformer. C) Torsional scan of protonated, aldoxime-abbreviated **20e** about the carbamate C-N bond.

The *syn*/*anti* energy differences for the electronic series did not align well with the observed cleavage rates since the computational model utilizes protonated structures (Table 26). The tertiary amine tether is modeled as always protonated and capable of *syn*-stabilizing H-bonding. However, in solution the protonation state is dependent on the tertiary amine pK_a. Thus, the amine tethers' proton affinity precedes the energetic difference between conformers (Table 26).

4.3 Results

Table S25: Computational Results against Experimental Data for Steric Group

#	R_1:	*Syn* energy relative to *Anti* (kcal/mol)	% Remaining @ 2h, PBS, 37 °C	$t_{1/2}$ (h) PBS, 37 °C
**20a**	Me	+4.24	77%	8.07
**20b**	*c*Pr	+4.10	68%	4.50
**20c**	*i*Pr	+3.89	53%	3.27
**20d**	*c*Bu	+3.55	44%	2.32
**20e**	*c*Pe	+3.11	34%	1.74
**20f**	*i*Bu	+4.37	55%	5.07

Table S26: Computational Results and pKa against Experimental Data for Electronic Group

#	*Syn* energy relative to *Anti* (kcal/mol)	% Remaining @ 2h, PBS, 37 °C	$t_{1/2}$ (h) PBS 37 °C	Tether pK_a
**20g**	ND	ND	ND	8.19±0.47
**20h**	0.67	ND	ND	8.45±0.52
**20i**	0.59	77%	5.45	6.84±0.20
**20j**	0.81	ND	ND	6.08±0.34
**20k**	ND	70%	5.99	6.24±0.86

5. General Experimental

Chemistry. *Materials and Methods.* All chemicals were purchased from commercial vendors at the highest commercial quality and used without further purification. Dichloromethane (DCM), acetonitrile (MeCN), toluene, dimethylformamide (DMF), tetrahydrofuran (THF) and triethylamine (TEA) were purchased anhydrous in septum-sealed bottles from Sigma Aldrich. 4-Nitrophenyl chloroformate was purchased from Ambeed. Commercially available amines employed include *tert*-Butyl methyl(2-(methylamino)ethyl)carbamate (**16a**), 1,7-Bis-boc-1,4,7-triazaheptane (**17w**), (2-([2-(Dimethylamino)ethyl]amino)ethyl)dimethylamine (**17y**), N,N'-Bis(2-hydroxyethyl)ethylenediamine (**17z**) from Combi-Blocks. Intermediates **13, 18a, 18l** and prodrugs **6** and **7** were synthesized as before.[9] All reactions were conducted using oven-dried glassware under an inert atmosphere of argon unless noted otherwise. Isolated yields refer to chromatographically and spectroscopically (^{1}H NMR, ^{13}C NMR) homogeneous material, unless otherwise stated. Thin layer chromatography (TLC) was utilized to monitor reaction progress using Merck silica gel 60 F254 aluminum-backed plates. TLC spots were visualized with UV, $KMnO_4$, PMA, or ninhydrin stains. Normal and reverse phase flash chromatography was performed using a Teledyne Isco CombiFlash Rf system using RediSep® Rf silica gel disposable flash columns (60 Å pore size, 40-60 µm particle size) and RediSep Gold® C18Aq Reversed Phase Columns (20-40 µm particle size). NMR spectra were acquired using a 400 MHz Varian INOVA or 600 MHz Bruker Avance III. Chemical shifts are reported in δ ppm and referenced using residual solvent peaks ($CDCl_3$, DMSO-d_6, MeOD). Rotamer signals are denoted with *. High resolution mass spectrometry (HRMS) was performed on a Thermo Exactive Plus Orbitrap Mass Spectrometer using APCI or ESI ionization methods. Note compounds **20g**, **20h**, **20j**, **20w** and **20x** do not ionize well and their HRMS could not be detected. Liquid chromatography-mass spectrometry (LC-MS) was performed on an Agilent 1200 HPLC equipped with da 6120 Quadrupole mass spectrometer (ESI), Diode-array detector, and an Agilent InfinityLab Poroshell 120 EC-C18 (2.1 mm x 50 mm, 2.7 µm) column heated to 35 °C. Mobile Phase: water/MeCN (0.1% FA). Purity was assessed as % of AUC_{total} at 254 nm. All final compounds were determined to be ≥95% pure unless stated otherwise.

6. General Synthetic Procedures

6.1 General Procedure A: Reductive Amination

An oven dried 2-neck round bottom flask, equipped with stirrer bar and molecular sieves (3Å), was charged with *tert*-butyl methyl(2-oxoethyl)carbamate **15** (1 equiv.) and anhydrous MeOH (6mL/mmol). To this was added amine nucleophile **16b-k** (2 equiv.) and the reaction mixture stirred for 12–18 hr (dependent on amine nucleophile utilized) at RT. For amine hydrochloride nucleophiles, additional triethylamine (2 equiv.) was added. Following complete consumption of the starting material and formation of imine intermediate, as monitored by TLC, the reaction was charged with palladium on carbon (15 mol%), purged and stirred under H_2 atmosphere for 4 hr. Following complete consumption of the imine intermediate monitored by TLC, the crude reaction mixture was filtered through celite, evaporated *in vacuo* and purified by flash chromatography.

6.2 General Procedure B: Cbz Protection

An oven-dried 100 mL Schlenk tube was charged with a stirrer bar, specific precursor (**17m_i, 17n_i, 17o_i**) and placed under argon. Anhydrous THF (5 mL/mmol) was added followed by potassium carbonate (2 equiv.). The mixture was then cooled to 0 °C in a brine ice bath. Benzyl chloroformate (1 equiv.) was then added dropwise, and the mixture was allowed to warm to RT and stirred overnight. Afterwards, the reaction was quenched with water and extracted with ethyl acetate. The organic layer was separated and washed with saturated ammonium chloride solution and brine. The organic layer was then dried over anhydrous sodium sulfate and concentrated *in vacuo* and purified using flash chromatography.

6.3 General Procedure C: Methylation

An oven-dried 100 mL Schlenk tube was charged with a stirrer bar, Cbz-protected precursor (**17m_ii, 17n_ii, 17o_i**i) and placed under argon. Anhydrous THF (6 mL/mmol) was then added, and the mixture was cooled to 0 °C in a brine ice bath. Sodium hydride (1-2 equiv.) was then added to the mixture. After stirring for 30 minutes, methyl iodide (1-2 equiv.) was added dropwise to mixture at 0 °C, and the resulting mixture was allowed to warm to RT and stirred overnight. Afterward, the reaction mixture was quenched by adding a few drops of DI water and then pouring the mixture into saturated ammonium chloride solution. The organic layer was then washed with brine and dried over anhydrous sodium sulfate. Following filtration, the solvent was concentrated *in vacuo* and purified using flash chromatography.

6.4 General Procedure D: Cbz Deprotection

An oven-dried 100 mL round bottom flask was charged with a stirrer bar, precursor (**17m_iii, 17n_iii, 17o_iii**), palladium on carbon (20 mol%), and anhydrous methanol (5 mL/mmol) under argon. A T-joint bearing a H_2 balloon and Schlenk line connection was affixed to the flask. The reaction flask was then briefly evacuated and filled with H_2. This process of H_2 filling was repeated 3 times. The mixture was then allowed to stir at RT under H_2 for 2 h. Afterward, the mixture was filtered over celite, with the celite further washed with methanol. The combined eluants were then concentrated *in vacuo* to afford a colorless oil that was immediately carried forward to the next reaction.

6.5 General Procedure E: Introduction of Amine

A flame-dried 100 mL round bottom flask was charged with a stirrer bar, intermediate **13** or **14** (1 equiv.), anhydrous DCM (5 mL/mmol), amine (1.2 equiv.) and TEA (or DIPEA) (2.5 equiv.) under argon. The resulting mixture was allowed to stir for 1 h. Afterward, the solution was diluted in EtOAc (50 mL) and washed with brine (5 × 100 mL brine). The organic layer was then dried over anhydrous sodium sulfate and concentrated *in vacuo* to afford a yellow oil. Flash chromatography (ethyl acetate:DCM) afforded the product as a white solid. In cases where the amine is more sterically hindered, 4-dimethylaminopyridine (1.5 equiv.) was also added and the reaction was allowed to stir for longer (monitored by TLC) at RT.

6.6 General Procedure F: Boc Deprotection

To a flame-dried vial containing a stirred bar and Boc-protected amine (1 equiv.) was added anhydrous DCM (2 mL/mmol) under argon. The resulting solution was then cooled to 0 °C in a brine ice bath. Trifluoroacetic acid (TFA, 1 mL) was then added dropwise, and the resulting solution was stirred at 0 °C for 1 h. The resulting solution was then concentrated *in vacuo* to afford a pink oil which was immediately purified by column chromatography (5–30% MeOH/DCM).

7. Synthetic Procedures and Characterization

7.1 Preparation of Activated C20-Oxime Intermediates 13 and 14

Starting from commercially available pregnenolone **11**, the 4-nitrophenylcarbonate of progesterone C20-oxime (**13**) was synthesized as previously (**Scheme S1a**).[9] Similarly, starting from allopregnanolone **12**, **14** was successfully prepared *via* the silyl-protected intermediate **12i** and C-20 oxime precursor **12ii** (**Scheme S1b**).

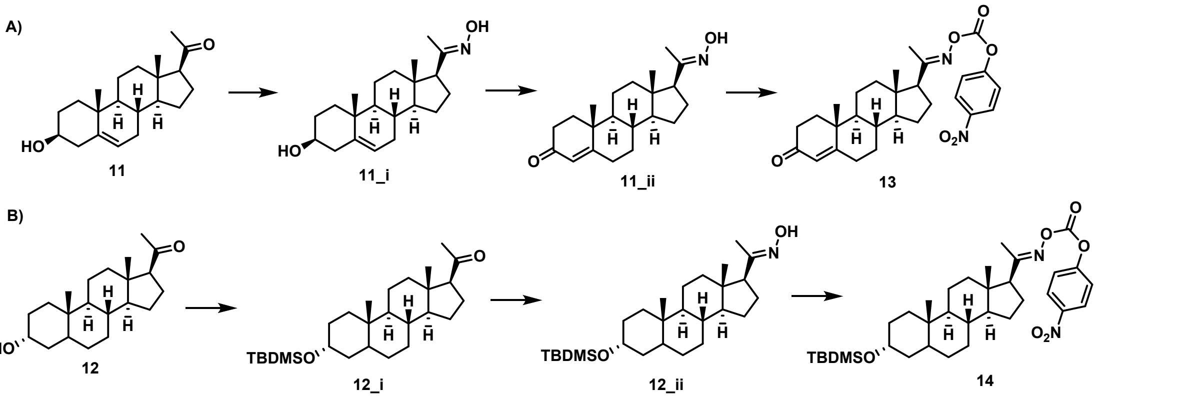

Scheme S1: Synthetic layout towards **13**[9] and **14**. See below for experimental details of **12i**, **12ii** and **14**.

Preparation of 1-((3*R*,8*R*,9*S*,10*S*,13*S*,14*S*,17*S*)-3-((tert-butyldimethylsilyl)oxy)-10,13-dimethylhexadecahydro-1H-cyclopenta[a]phenanthren-17-yl)ethan-1-one (12i)

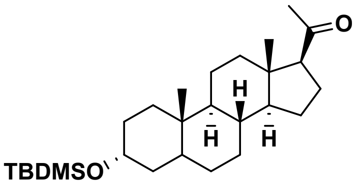

To an oven dried two-neck round bottomed flask, equipped with stirrer bar, was added allopregnanolone **12** (4 g, 12.56 mmol, 1 equiv), 2,6-lutidine (4.4 mL, 37.68 mmol, 3 equiv.) and anhydrous DCM (6mL/mmol of steroid). The reaction mixture was cooled to 0 °C in an ice-bath, and TBDMSOTf (4.33 mL, 18.84 mmol, 1.5 equiv.) diluted in anhydrous DCM (2 mL/mmol silyl triflate 0.5M) was added dropwise. The reaction was stirred and allowed to acclimate to RT overnight. Upon completion, the mixture was quenched with H_2O, diluted with DCM and transferred to a separatory funnel. The organic layer was washed with H_2O, followed by brine and dried on anhydrous magnesium sulfate. After filtration the crude product was dried in vacuo and purified using column chromatography to afford 1-((3*R*,8*R*,9*S*,10*S*,13*S*,14*S*,17*S*)-3-((tert-butyldimethylsilyl)oxy)-10,13-dimethylhexadecahydro-1H-cyclopenta[a]phenanthren-17-yl)ethan-1-one **12i** (5.15 g, 11.90 mmol, 95% yield).

^{1}H NMR (600 MHz, CDCl$_3$) δ 3.96 (t, *J* = 2.8 Hz, 1H), 2.52 (t, *J* = 9.1 Hz, 1H), 2.18 – 2.11 (m, 1H), 2.11 (s, 3H), 2.02 – 1.96 (m, 1H), 1.69 – 1.45 (m, 7H), 1.45 – 1.30 (m, 5H), 1.30 – 1.12 (m, 6H), 0.99 – 0.91 (m, 1H), 0.89 (s, 9H), 0.80 – 0.76 (m, 1H), 0.75 (s, 3H), 0.59 (s, 3H), 0.02 (d, *J* = 1.7 Hz, 6H); **^{13}C NMR (151 MHz, CDCl$_3$)** δ 210.0, 67.0, 64.1, 56.9, 54.4, 44.5, 39.3, 39.2, 36.9, 36.1, 35.7, 32.6, 32.2, 31.7, 29.9, 28.7, 26.0, 24.5, 22.9, 21.0, 18.3, 13.6, 11.5, -4.7; **HRMS (APCI+)** $[M+H]^+$ calc. for $C_{27}H_{49}O_2Si$, 433.3496, observed, 433.3497. ^{1}H and ^{13}C NMR corresponds well to that reported in the literature.[10]

Preparation of (*E*)-1-((3*R*,8*R*,9S,10*S*,13*S*,14*S*,17*S*)-3-((*tert*-butyldimethylsilyl)oxy)-10,13-dimethylhexadecahydro-*1H*-cyclopenta[a]phenanthren-17-yl)ethan-1-one oxime (12ii)

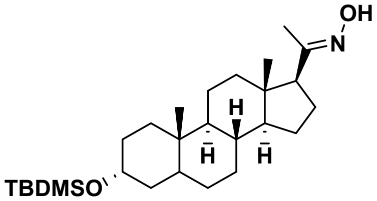

To a 3-neck 250 mL round bottomed flask with a stir bar and equipped with a condenser, was added 1-((3*R*,8*R*,9*S*,10*S*,13*S*,14*S*,17*S*)-3-((tert-butyldimethylsilyl)oxy)-10,13-dimethylhexadecahydro-*1H*-cyclopenta[a]phenanthren-17-yl)ethan-1-one (**12i**) (3.20 g, 7.40 mmol) in methanol (80 mL) and heated to reflux for 10 min to ensure dissolution of the suspended solid. A separate solution containing sodium acetate (1.34 g, 16.28 mmol, 2.2 equiv.), hydroxylamine hydrochloride (1.03 g, 14.79 mmol, 2 equiv.), and water (5 mL) was then slowly added dropwise (ca. 30 minutes). After addition, the mixture was allowed to reflux until complete conversion as observed by TLC (4 hours). A thick white precipitate formed during the reaction. After completion, the mixture was allowed to cool to rt and an additional 70 mL of water was added, followed by 30 minutes of stirring. The reaction mixture was then subjected to vacuum filtration, and the resulting white solid was washed with dI water 3 x 50 mL. Drying of the solid *in vacuo* afforded (*E*)-1-((3*R*,8*R*,9S,10*S*,13*S*,14*S*,17*S*)-3-((*tert*-butyldimethylsilyl)oxy)-10,13-dimethylhexadecahydro-*1H*-cyclopenta[a]phenanthren-17-yl)ethan-1-one oxime (**12ii**) (3.05 g, 6.811 mmol, 92 % yield) as a white solid.

^{1}H NMR (600 MHz, CDCl$_3$) δ 3.96 (t, *J* = 2.8 Hz, 1H), 2.52 (t, *J* = 9.1 Hz, 1H), 2.13 – 2.00 (m, 1H), 1.88 (s, 3H), 1.87 – 1.82 (m, 1H), 1.70 – 1.46 (m, 8H), 1.42 – 1.10 (m, 11H), 1.00 – 0.90 (m, 1H), 0.89 (s, 9H), 0.80 – 0.76 (m, 1H), 0.76 (s, 3H), 0.61 (s, 3H), 0.02 (d, *J* = 1.5 Hz, 6H); **^{13}C NMR (151 MHz, CDCl$_3$)** δ 159.2, 67.0, 57.1, 56.1, 54.5, 44.2, 39.2, 39.1, 36.9, 36.2, 35.9, 32.6, 32.2, 29.9, 28.7, 26.0, 24.3, 23.2, 20.9, 18.3, 15.3, 13.5, 11.6, -4.7; **HRMS (APCI+)** $[M+H]^+$ calc. for $C_{27}H_{50}O_2Si$, 448.3605, observed, 448.3605.

Preparation of (*E*)-1-((3*R*,8*R*,9*S*,10*S*,13*S*,14*S*,17*S*)-3-((*tert*-butyldimethylsilyl)oxy)-10,13-dimethylhexadecahydro-*1H*-cyclopenta[a]phenanthren-17-yl)ethan-1-one O-((4-nitrophenoxy)carbonyl) oxime (14)

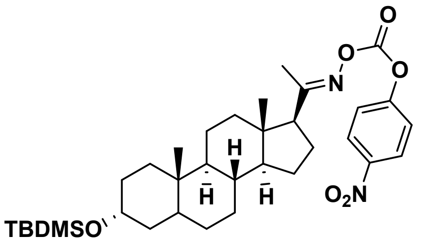

To a flame-dried round bottom flask, equipped with a stir bar, was added (*E*)-1-((3*R*,8*R*,9S,10*S*,13*S*,14*S*,17*S*)-3-((*tert*-butyldimethylsilyl)oxy)-10,13-dimethylhexadecahydro-*1H*-cyclopenta[a]phenanthren-17-yl)ethan-1-one oxime (**12ii**) (2.60 g, 5.81 mmol), DIPEA (1.52 mL, 8.71 mmol, 1.5 equiv.) and anhydrous DCM (100 mL) under argon. The mixture was then cooled 0 °C in a brine ice bath. 4-Nitrophenyl chloroformate (1.76 g, 8.71 mmol, 1.5 equiv.) was then added portion wise and the resulting mixture was allowed to stir at 0 °C for 1.5 hours. Afterwards, the mixture was concentrated *in vacuo,* adsorbed onto celite, and subjected to flash chromatography to afford (*E*)-1-((3*R*,8*R*,9*S*,10*S*,13*S*,14*S*,17*S*)-3-((*tert*-butyldimethylsilyl)oxy)-10,13-dimethylhexadecahydro-*1H*-cyclopenta[a]phenanthren-17-yl)ethan-1-one O-((4-nitrophenoxy)carbonyl) oxime (**14**) (3.312 g, 7.372 mmol, 93% yield) as a white solid.

^{1}H NMR (600 MHz, $CDCl_3$) δ 8.29 (d, *J* = 9.4 Hz, 2H), 7.44 (d, *J* = 9.4 Hz, 2H), 3.96 (p, *J* = 2.8 Hz, 1H), 2.38 (t, 1H), 2.30 – 2.20 (m, 1H), 2.05 (s, 3H), 1.88 (dt, 1H), 1.80 – 1.65 (m, 3H), 1.65 – 1.57 (m, 2H), 1.56 – 1.47 (m, 2H), 1.42 – 1.31 (m, 5H), 1.29 – 1.12 (m, 6H), 1.01 – 0.91 (m, 1H), 0.89 (s, 9H), 0.82 – 0.77 (m, 1H), 0.76 (s, 3H), 0.69 (s, 3H), 0.02 (s, 6H); **^{13}C NMR (151 MHz, $CDCl_3$)** δ 168.6, 155.6, 151.4, 145.6, 125.5, 121.9, 67.0, 57.2, 56.3, 54.4, 44.7, 39.2, 39.1, 36.9, 36.2, 35.9, 32.6, 32.2, 29.9, 28.7, 26.0, 24.3, 23.1, 20.9, 18.3, 17.3, 13.7, 11.6, -4.7. 29; **HRMS (APCI+)** $[M+H]^+$ calc. for $C_{34}H_{53}O_6N_2Si_{28}$, 613.3667, observed, 613.3669.

7.2 Preparation of Amine Linkers

Preparation of *tert*-butyl (2-(cyclopropylamino)ethyl)(methyl)carbamate (17b)

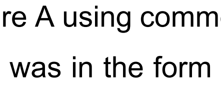

Prepared according to General Procedure A using commercially available cyclopropanamine. Rf = 0.57 (15% MeOH/DCM). The product was in the form of an oil (2224 mg, 10.378 mmol, 81% yield). **^{1}H NMR (400 MHz, $CDCl_3$)** δ 3.39 – 3.24 (m, 2H), 2.86 (s, 3H), 2.84 – 2.81 (m, 2H), 2.22 – 2.05 (m, 2H), 1.44 (s, 9H), 0.48 – 0.37 (m, 2H), 0.37 – 0.29 (m, 2H); **^{13}C NMR (101 MHz, $CDCl_3$)** δ 156.1, 79.6, 48.9, 47.3, 34.8, 30.2, 28.0, 6.4.

Preparation of *tert*-butyl (2-(isopropylamino)ethyl)(methyl)carbamate (17c)

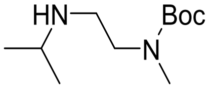

Prepared according to General Procedure A using commercially available propan-2-amine. Rf = 0.45 (15% MeOH/DCM). The product was in the form of an oil (507 mg, 2.344 mmol, 52% yield). **^{1}H NMR (400 MHz, CDCl3)** δ 3.33 – 3.25 (m, 2H), 2.84 (s, 3H), 2.81 – 2.77 (m, 1H), 2.72 (t, J = 6.7 Hz, 2H), 2.03 (br s, 1H), 1.42 (s, 9H), 1.03 (d, J = 6.3 Hz, 6H); **^{13}C NMR (101 MHz, CDCl3)** δ 156.1, 79.7, 49.1, 48.5, 45.0, 34.8, 28.1, 22.8.

Preparation of *tert*-butyl (2-(cyclobutylamino)ethyl)(methyl)carbamate (17d)

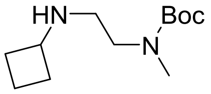

Prepared according to General Procedure A using commercially available cyclobutanamine. Rf = 0.52 (15% MeOH/DCM). The product was in the form of an oil (870 mg, 3.81 mmol, 66% yield). **^{1}H NMR (400 MHz, $CDCl_3$)** δ 3.33 – 3.19 (m, 3H), 2.85 (s, 3H), 2.67 (t, *J* = 6.9 Hz, 2H), 2.25 – 2.15 (m, 2H), 1.90 (s, 1H), 1.74 – 1.55 (m, 4H), 1.44 (s, 9H); **^{13}C NMR (101 MHz, $CDCl_3$)** δ 156.0, 79.6, 53.9, 49.2, 44.7, 34.9, 31.1, 28.6, 14.6.

Preparation of *tert*-butyl (2-(cyclopentylamino)ethyl)(methyl)carbamate (17e)

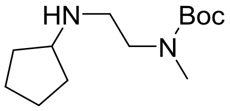

Prepared according to General Procedure A using commercially available cyclopentanamine. Rf = 0.50 (15% MeOH/DCM). The product was in the form of an oil (966 mg, 3.984 mmol, 69% yield). **^{1}H NMR (400 MHz, $CDCl_3$)** δ 3.35 – 3.22 (m, 2H), 3.08 (p, *J* = 6.8 Hz, 1H), 2.85 (s, 3H), 2.73 (t, *J* = 6.7 Hz, 2H), 2.05 (br s, 1H), 1.89 – 1.76 (m, 2H), 1.73 – 1.59 (m, 2H), 1.58 – 1.46 (m, 2H), 1.43 (s, 9H), 1.34 – 1.27 (m, 2H); **^{13}C NMR (101 MHz, $CDCl_3$)** δ 156.1, 79.7, 59.6, 49.1, 46.3, 34.9, 33.1, 28.6, 24.1.

Preparation of *tert*-butyl (2-(isobutylamino)ethyl)(methyl)carbamate (17f)

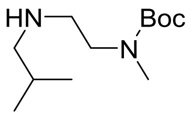

Prepared according to General Procedure A using commercially available 2-methylpropan-1-amine. Rf = 0.56 (15% MeOH/DCM). The product was in the form of an oil (958 mg, 4.157 mmol,

72% yield). **^{1}H NMR (400 MHz, CDCl$_3$)** δ 3.39 – 3.24 (m, 2H), 2.86 (s, 3H), 2.79 – 2.69 (m, 2H), 2.42 (d, *J* = 6.8 Hz, 2H), 1.91 (br s, 1H), 1.73 (hept, *J* = 6.6 Hz, 1H), 1.44 (s, 9H), 0.89 (d, *J* = 6.7 Hz, 6H); **^{13}C NMR (101 MHz, CDCl$_3$)** δ 156.1, 79.6, 57.8, 48.9, 47.9, 34.9, 28.6, 28.4, 20.7.

Preparation of *tert*-butyl (2-((2-(dimethylamino)ethyl)amino)ethyl)(methyl)carbamate (17g)

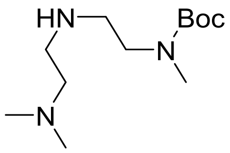

Prepared according to General Procedure A using commercially available *N*^{1},*N*^{1}-dimethylethane-1,2-diamine. Rf = 0.21 (15% MeOH/DCM). The product was in the form of an oil (978 mg, 3.984 mmol, 69% yield). **^{1}H NMR (400 MHz, CDCl$_3$)** δ 3.36 – 3.26 (m, 2H), 2.85 (s, 3H), 2.75 (br s, 2H), 2.73 – 2.65 (m, 2H), 2.40 (t, *J* = 6.1 Hz, 2H), 2.29 – 2.23 (m, 1H), 2.20 (s, 6H), 1.43 (s, 9H); **^{13}C NMR (101 MHz, CDCl$_3$)** δ 156.1, 79.6, 59.0, 49.0, 48.0, 47.3, 45.6, 34.9, 27.2.

Preparation of *tert*-butyl methyl(2-((2-(pyrrolidin-1-yl)ethyl)amino)ethyl)carbamate (17h)

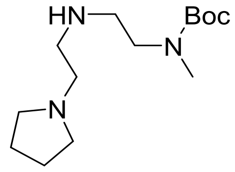

Prepared according to General Procedure A using commercially available 2-(pyrrolidin-1-yl)ethan-1-amine. Rf = 0.25 (20% MeOH/DCM). The product elutes at 25 – 50% MeOH/DCM and was in the form of an oil (631 mg, 2.325 mmol, 40% yield). **^{1}H NMR (400 MHz, DMSO-d$_6$)** δ 3.20 (t, *J* = 6.7 Hz, 2H), 2.77 (br s, 3H), 2.65– 2.56 (m, 4H), 2.49 – 2.33 (m, 6H), 1.72 – 1.58 (m, 4H), 1.38 (s, 9H). NH not observed; **^{13}C NMR (101 MHz, DMSO-D6)** δ 154.9, 78.2, 55.6, 53.7, 48.3 (br s), 48.0, 47.7 (br s), 47.3 (br s), 47.0 (br s), 34.2 (br s), 28.1, 23.09.

Preparation of *tert*-butyl methyl(2-((3-morpholinopropyl)amino)ethyl)carbamate (17i)

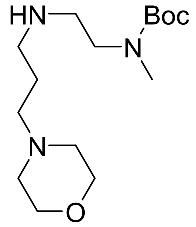

Prepared according to General Procedure A using commercially available 3-morpholinopropan-1-amine. Rf = 0.42 (10% MeOH/DCM). The product elutes at 10 – 15% MeOH/DCM and was in the form of an oil (1950 mg, 6.468 mmol, 56% yield). **^{1}H NMR (400 MHz, DMSO-d$_6$)** δ 3.69 (t, *J*

= 4.7 Hz, 4H), 3.40 – 3.28 (m, 2H), 2.87 (s, 3H), 2.80 – 2.64 (m, 4H), 2.46 – 2.33 (m, 6H), 1.75 – 1.65 (m, 2H), 1.44 (s, 9H). NH not observed; **^{13}C NMR (101 MHz, $CDCl_3$)** δ 156.1, 79.8, 77.4, 67.1, 57.5, 53.9, 53.8 (br s), 48.5, 48.0 (br s), 47.5 (br s), 35.1 (br s), 29.3, 25.7 (br s).

Preparation of *tert*-butyl methyl(2-((2-morpholinoethyl)amino)ethyl)carbamate (17j)

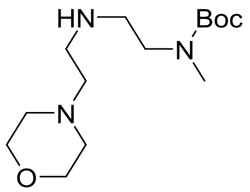

Prepared according to General Procedure A using commercially available 2-morpholinoethan-1-amine. Rf = 0.48 (20% MeOH/DCM). The product elutes at 10 – 20% MeOH/DCM and was in the form of an oil (1219 mg, 4.241 mmol, 49% yield). **^{1}H NMR (400 MHz, DMSO-d_6)** δ 3.68 – 3.62 (m, 2H), 3.33 – 3.24 (m, 2H), 2.83 (s, 3H), 2.78 – 2.63 (m, 4H), 2.48 – 2.35 (m, 5H), 2.21 – 2.17 (m, 3H), 1.41 (s, 9H). NH not observed; **^{13}C NMR (101 MHz, $CDCl_3$)** δ 156.0, 79.5, 67.0, 58.6*, 53.79, 48.5*, 47.7*, 47.0*, 45.82, 45.5, 34.8, 28.5.

Preparation of *tert*-butyl (2-((2-(1*H*-imidazol-1-yl)ethyl)amino)ethyl)(methyl)carbamate (17k)

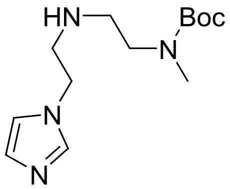

Prepared according to General Procedure A using commercially available 2-(1*H*-imidazol-1-yl)ethan-1-amine. The product was in the form of an oil (1924 mg, 7.2765 mmol, 63% yield). **^{1}H NMR (400 MHz, DMSO-d_6)** δ 7.53 (s, 1H), 7.01 (s, 1H), 6.95 (s, 1H), 4.07 (t, J = 5.9 Hz, 2H), 3.36 – 3.22 (m, 2H), 3.04 – 2.95 (m, 2H), 2.85 – 2.79 (m, 3H), 2.79 – 2.70 (m, 2H), 2.71 – 2.59 (m, 2H), 1.42 (s, 9H); **^{13}C NMR (101 MHz, $CDCl_3$)** δ 156.2, 137.4, 129.5, 119.1, 79.8, 49.6*, 48.6, 47.6*, 47.1, 34.9, 28.5.

Preparation of *tert*-butyl (*R*)-2-((methylamino)methyl)pyrrolidine-1-carboxylate (17m)

Tert-butyl (*R*)-2-((methylamino)methyl)pyrrolidine-1-carboxylate (**17m**) was prepared according to **Scheme S2**.

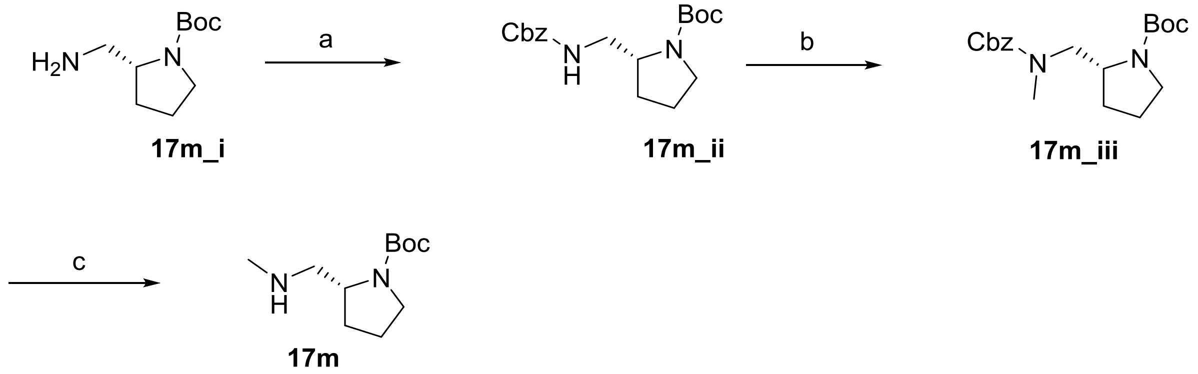

Scheme S2: (a) Cbz-Cl, K_2CO_3, THF, 0 °C–rt. (b) MeI, NaH, THF, 0 °C–rt. (c) $H_{2(g)}$, Pd/C, MeOH, rt.

Preparation of *tert*-butyl (*R*)-2-((((benzyloxy)carbonyl)amino)methyl)pyrrolidine-1-carboxylate (17m_ii)

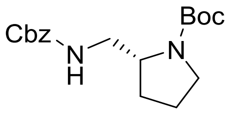

Prepared according to General Procedure B using commercially available *tert*-butyl (*R*)-2-(aminomethyl)pyrrolidine-1-carboxylate (**17m_i**). The product was in the form of an oil (1431 mg, 4.284 mmol, 86% yield). **^{1}H NMR (400 MHz, $CDCl_3$) δ** 7.37 – 7.27 (m, 5H), 6.02 (br s, 1H), 5.09 (s, 2H), 3.92 (br s, 1H), 3.47 – 3.16 (m, 4H), 2.01 – 1.89 (m, 1H), 1.89 – 1.76 (m, 2H), 1.76 – 1.65 (m, 1H), 1.45 (s, 9H); **^{13}C NMR (101 MHz, $CDCl_3$) δ** 156.8, 155.6, 136.9, 128.6, 128.1, 80.0, 77.4, 66.7, 57.0, 47.0, 45.6, 29.4, 28.6, 23.7.

Preparation of *tert*-butyl (*R*)-2-((((benzyloxy)carbonyl)(methyl)amino)methyl)pyrrolidine-1-carboxylate (17m_iii)

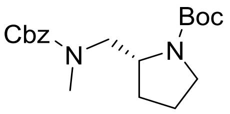

Prepared according to General Procedure C using *tert*-butyl (*R*)-2-((((benzyloxy)carbonyl)amino)methyl)pyrrolidine-1-carboxylate (**17_ii**). The product was in the form of an oil (1011 mg, 2.904 mmol, 78% yield). **^{1}H NMR (400 MHz, $CDCl_3$)** δ 7.38 – 7.27 (m, 5H), 5.17 – 5.05 (m, 2H), 4.01 – 3.92 (m, 1H), 3.62 – 3.38 (m, 1H), 3.37 – 3.12 (m, 3H), 2.96 (s, 3H), 1.98 – 1.66 (m, 4H), 1.46 (s, 9H), 1.45* (s, 9H); **^{13}C NMR (101 MHz, $CDCl_3$)** δ 157.0, 154.7, 137.0, 128.6, 128.3, 127.9, 79.5, 77.4, 67.2, 55.4, 50.7, 46.5, 34.7, 28.6, 28.1, 23.3. One extra ^{13}C signal present and multiple peak splitting.

Preparation of *tert*-butyl (*R*)-2-((methylamino)methyl)pyrrolidine-1-carboxylate (17m)

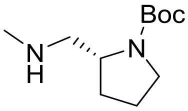

Prepared according to General Procedure D using *tert*-butyl (*R*)-2-((((benzyloxy)carbonyl)(methyl)amino)methyl)pyrrolidine-1-carboxylate (**17m_iii**). The product was in the form of an oil (665 mg, 3.107 mmol, 76% yield). **^{1}H NMR (400 MHz, DMSO-d_6) δ** 3.71 (s, 1H), 3.19 (s, 2H), 2.98 – 2.74 (m, 1H), 2.65 – 2.53 (m, 1H), 2.46 – 2.30 (m, 1H), 2.27 (s, 3H), 1.85 – 1.63 (m, 4H), 1.39 (s, 9H); **^{13}C NMR (101 MHz, DMSO-d_6)** δ 153.5, 78.1, 56.1, 54.2, 46.1, 36.2, 28.8, 28.1, 23.2. Multiple peak splitting in ^{13}C spectrum.

Preparation of *tert*-butyl (*S*)-2-(2-(methylamino)ethyl)pyrrolidine-1-carboxylate (17n)

Tert-butyl (*S*)-2-(2-(methylamino)ethyl)pyrrolidine-1-carboxylate (**17n**) was prepared according to **Scheme S3**.

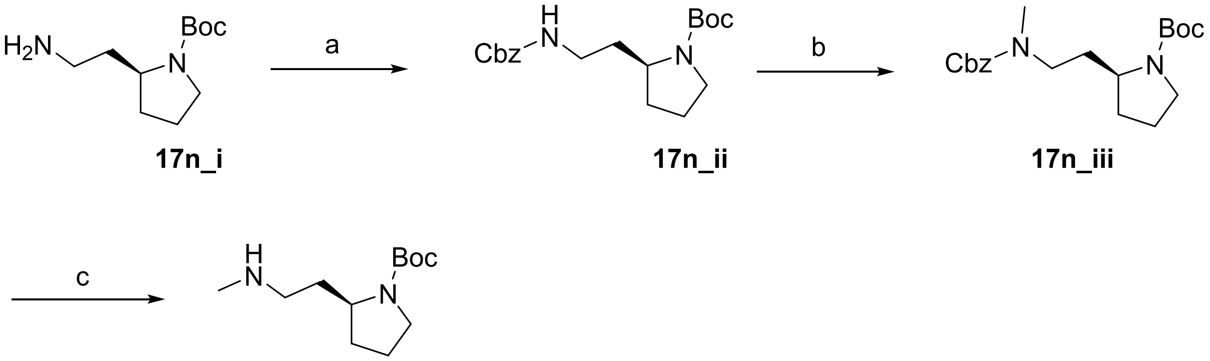

17n_i **17n_ii** **17n_iii**

17n

Scheme S3: (a) Cbz-Cl, K_2CO_3, THF, 0 °C–rt. (b) MeI, NaH, THF, 0 °C–rt. (c) $H_{2(g)}$, Pd/C, MeOH, rt.

Preparation of *tert*-butyl (*S*)-2-(2-(((benzyloxy)carbonyl)amino)ethyl)pyrrolidine-1-carboxylate (17n_ii)

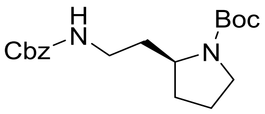

Prepared according to General Procedure B using commercially available *tert*-butyl (*S*)-2-(2-aminoethyl)pyrrolidine-1-carboxylate (**17n_i**). Flash chromatography (0–50% ethyl acetate/hexanes) afforded *tert*-butyl (*S*)-2-(2-(((benzyloxy)carbonyl)amino)ethyl)pyrrolidine-1-carboxylate (**18**) (799 mg, 2.293 mmol, 98% yield) as a colorless oil. **^{1}H NMR (400 MHz, DMSO-d_6)** δ 7.39 – 7.27 (m, 5H), 5.05 – 4.95 (m, 2H), 3.67 (br s, 1H), 3.27 – 3.10 (m, 2H), 3.08 – 2.87 (m, 3H), 1.91 – 1.68 (m, 5H), 1.65 – 1.53 (m, 1H), 1.38 (s, 9H); **^{13}C NMR (101 MHz, DMSO-d_6)**

δ 156.0, 153.5, 137.3, 128.3, 127.7, 127.4, 78.1, 65.1, 54.7, 45.8, 37.9, 34.7, 30.3, 28.1, 22.6. Rotamers present.

Preparation of *tert*-butyl (*S*)-2-(2-(((benzyloxy)carbonyl)(methyl)amino)ethyl)pyrrolidine-1-carboxylate (17n_iii)

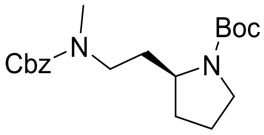

Prepared according to General Procedure C using *tert*-butyl (*S*)-2-(2-(((benzyloxy)carbonyl)amino)ethyl)pyrrolidine-1-carboxylate (**17n_ii**). Flash chromatography (0–50% ethyl acetate in hexanes) afforded *tert*-butyl (*S*)-2-(2-(((benzyloxy)carbonyl)(methyl)amino)ethyl)pyrrolidine-1-carboxylate (**19**) (685 g, 1.890 mmol, 85% yield) as a yellow oil. **^{1}H NMR (600 MHz, $CDCl_3$)** δ 7.36 – 7.33 (m, 4H), 7.32 – 7.28 (m, 1H), 5.11 (s, 2H), 3.72 (s, 1H), 3.38 – 3.21 (m, 4H), 2.92 (s, 3H), 2.07 – 1.88 (m, 1H), 1.88 – 1.65 (m, 5H), 1.44 (s, 9H); **^{13}C NMR (151 MHz, $CDCl_3$)** δ 156.3, 154.7, 137.1, 128.6, 128.1, 128.0, 79.3, 67.1, 55.2, 46.5, 46.4, 34.7, 32.8, 30.6, 28.7, 23.5.

Preparation of *tert*-butyl (*S*)-2-(2-(methylamino)ethyl)pyrrolidine-1-carboxylate (17n)

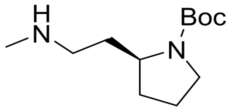

Prepared according to General Procedure D using *tert*-butyl (*S*)-2-(2-(((benzyloxy)carbonyl)(methyl)amino)ethyl)pyrrolidine-1-carboxylate (**17n_iii**). Concentrated *in vacuo* to afford a colorless oil. **^{1}H NMR (600 MHz, DMSO-d_6) δ** 3.70 (br s, 1H), 3.26 – 2.88 (m, 4H), 2.44 – 2.35 (m, 2H), 2.24 (s, 3H), 1.90 – 1.69 (m, 4H), 1.61 (s, 1H), 1.39 (s, 9H); **^{13}C NMR (151 MHz, DMSO-d_6) δ** 153.5, 78.0, 55.0, 48.8, 45.7*, 36.2, 34.3*, 30.4*, 28.2, 23.3*. Multiple peak splitting in ^{13}C spectrum.

Preparation of *tert*-butyl (*S*)-2-((methylamino)methyl)azetidine-1-carboxylate (17o)

Tert-butyl (*S*)-2-((methylamino)methyl)azetidine-1-carboxylate (**17o**) was prepared according to **Scheme S4.**

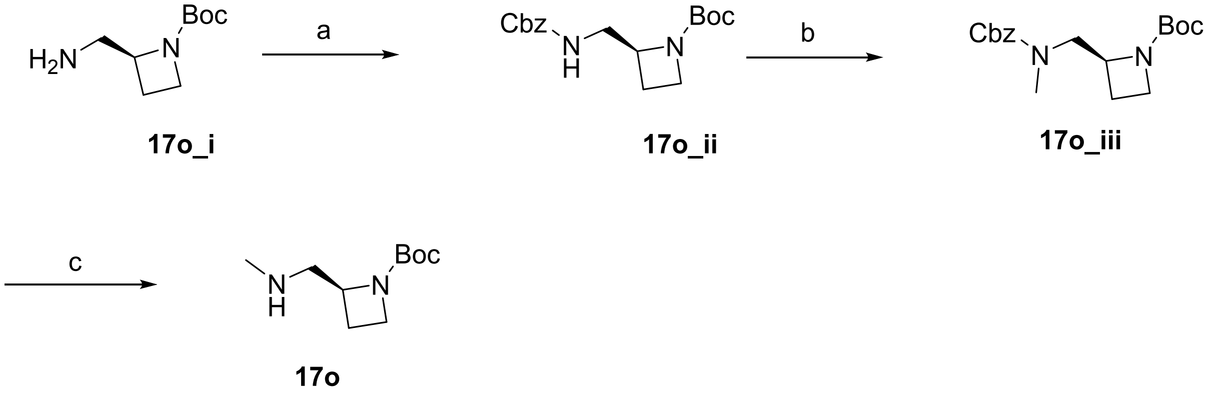

Scheme S4: (a) Cbz-Cl, K_2CO_3, THF, 0 °C–rt. (b) MeI, NaH, THF, 0 °C–rt. (c) $H_{2(g)}$, Pd/C, MeOH, rt.

Preparation of *tert*-butyl (*S*)-2-((((benzyloxy)carbonyl)amino)methyl)azetidine-1-carboxylate (17o_ii)

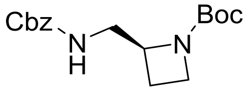

Prepared according to General Procedure B using commercially available *tert*-butyl (*S*)-2-(aminomethyl)azetidine-1-carboxylate (**17o_i**). The product was in the form of an oil (1440 mg, 4.495mmol, 84% yield). **^{1}H NMR (400 MHz, CDCl$_3$)** δ 7.38 – 7.27 (m, 5H), 5.87 (br s, 1H), 5.11 (s, 2H), 4.36 – 4.27 (m, 1H), 3.88 – 3.79 (m, 1H), 3.79 – 3.71 (m, 1H), 3.54 (d, *J* = 13.7 Hz, 1H), 3.37 (dd, *J* = 13.7, 7.3 Hz, 1H), 2.29 – 2.16 (m, 1H), 2.00 – 1.87 (m, 1H), 1.44 (s, 9H); **^{13}C NMR (101 MHz, CDCl$_3$)** δ 156.9, 156.8, 136.8, 128.6, 128.2, 128.1, 80.3, 66.8, 61.7, 46.8, 46.1, 28.5, 19.3.

Preparation of *tert*-butyl (*S*)-2-((((benzyloxy)carbonyl)(methyl)amino)methyl)azetidine-1-carboxylate (17o_iii)

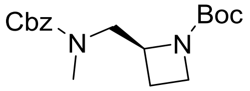

Prepared according to General Procedure C using *tert*-butyl (*S*)-2-((((benzyloxy)carbonyl)amino)methyl)azetidine-1-carboxylate (**17o_ii**). The product was in the form of an oil (1100 mg, 3.289 mmol, 81% yield). **^{1}H NMR (400 MHz, DMSO-d$_6$)** δ 7.41 – 7.26 (m, 6H), 5.06 (d, *J* = 4.5 Hz, 2H), 4.31 (p, *J* = 6.1 Hz, 1H), 3.77 – 3.56 (m, 3H), 3.41 – 3.28 (m, 1H), 2.93 (s, 3H), 2.89* (s, 3H), 2.26 – 2.13 (m, 1H), 1.93 – 1.78 (m, 1H), 1.36 (s, 9H), 1.33* (s, 9H); **^{13}C NMR (101 MHz, DMSO-d$_6$)** δ 155.8, 155.4, 137.0, 128.4, 127.7, 127.5, 78.6, 66.2, 59.9, 52.2, 46.1, 35.5, 35.1*, 28.0, 19.5, 19.4*.

Preparation of *tert*-butyl (*S*)-2-((methylamino)methyl)azetidine-1-carboxylate (17o)

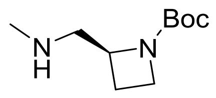

Prepared according to General Procedure D using *tert*-butyl (*S*)-2-((((benzyloxy)carbonyl)(methyl)amino)methyl)azetidine-1-carboxylate (**17o_iii**). The product was in the form of an oil (571 mg, 2.851 mmol, 91% yield). Used crude product immediately for the subsequent reaction.

Preparation of *tert*-butyl (*R*)-2-((*S*)-1-(methylamino)ethyl)pyrrolidine-1-carboxylate (17p)

Tert-butyl (*R*)-2-((*S*)-1-(methylamino)ethyl)pyrrolidine-1-carboxylate (**17p**) was prepared according to Scheme S5.

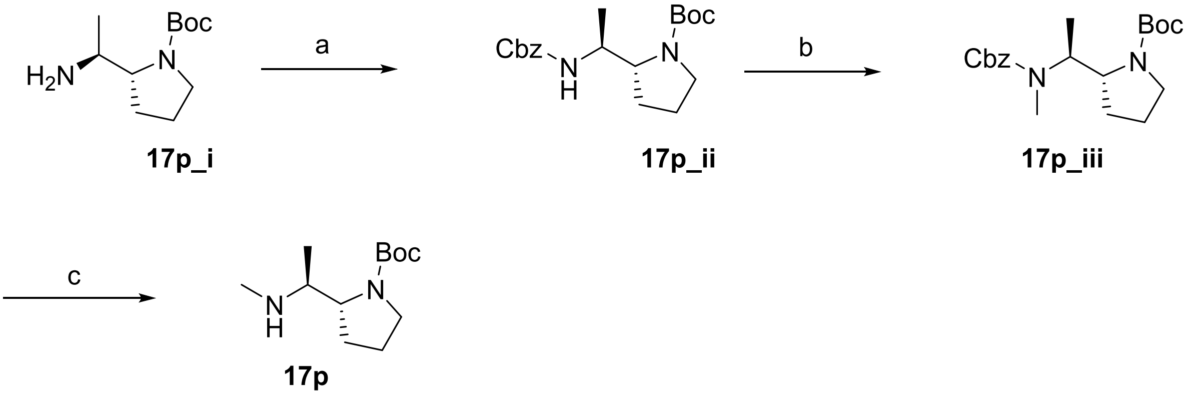

Scheme S5: a) Cbz-Cl, K_2CO_3, THF, 0 °C–rt. (b) MeI, NaH, DMF, rt. (c) $H_{2(g)}$, Pd/C, MeOH, rt.

Preparation of *tert*-butyl (*S*)-2-((*S*)-1-(((benzyloxy)carbonyl)amino)ethyl)pyrrolidine-1-carboxylate (17p_ii)

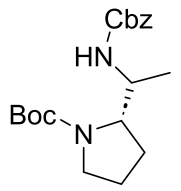

Prepared according to general Procedure B using commercially available *tert*-butyl (*S*)-2-((*S*)-1-aminoethyl)pyrrolidine-1-carboxylate (**17p_i**). The product was in the form of an oil (1330mg, 3.82mmol, 82% yield). **^{1}H NMR (600 MHz, $CDCl_3$)** δ 7.39 – 7.28 (m, 5H), 6.78 (s, 1H), 5.11 – 5.05 (m, 2H), 3.98 – 3.72 (m, 2H), 3.53 (s, 1H), 3.23 – 3.15 (m, 1H), 1.96 – 1.78 (m, 2H), 1.74 – 1.58 (m, 2H), 1.46 (s, 9H), 1.13 – 1.00 (m, 3H); **^{13}C NMR ($CDCl_3$)** δ 156.1, 154.8, 136.8, 128.5, 128.2, 80.4, 66.6, 62.0, 47.3, 46.7, 28.6, 28.5, 23.8, 17.3.

Preparation of *tert*-butyl (*S*)-2-((*S*)-1-(((benzyloxy)carbonyl)(methyl)amino)ethyl)pyrrolidine-1-carboxylate (17p_iii)

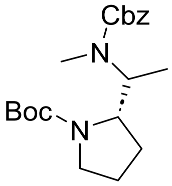

Prepared according to General Procedure C using *tert*-butyl (*S*)-2-((*S*)-1-(((benzyloxy)carbonyl)amino)ethyl)pyrrolidine-1-carboxylate (**17p_ii**) and anhydrous DMF instead of THF. The product was in the form of an oil (864mg, 2.38 mmol, 64% yield). **^{1}H NMR (600 MHz, $CDCl_3$)** δ 7.38 – 7.27 (m, 5H), 5.20 – 5.05 (m, 2H), 4.20 – 3.88 (m, 2H), 3.53 – 3.18 (m, 2H), 2.80 (s, 3H), 1.91 – 1.62 (m, 4H), 1.46 (s, 9H), 1.16 (d, *J* = 6.9 Hz, 3H); **^{13}C NMR ($CDCl_3$)** δ 156.4, 155.2, 136.9, 128.1, 128.0, 79.8, 67.2, 60.5, 53.3, 46.6, 29.8, 28.6, 28.2, 23.7, 14.3.

Preparation of *tert*-butyl (*S*)-2-((*S*)-1-(methylamino)ethyl)pyrrolidine-1-carboxylate (17p)

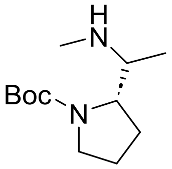

Prepared according to General Procedure D using tert-butyl (S)-2-((S)-1-(((benzyloxy)carbonyl)(methyl)amino)ethyl)pyrrolidine-1-carboxylate (**17p_iii**). The product was in the form of an oil and was immediately used for the subsequent reaction.

Preparation of *tert*-butyl (*R*)-6-((methylamino)methyl)-5-azaspiro[2.4]heptane-5-carboxylate (17q) and *tert*-butyl (*R*)-4,4-difluoro-2-((methylamino)methyl)pyrrolidine-1-carboxylate (17r)

Tert-butyl (*R*)-6-((methylamino)methyl)-5-azaspiro[2.4]heptane-5-carboxylate (**17q**) and *tert*-butyl (*R*)-4,4-difluoro-2-((methylamino)methyl)pyrrolidine-1-carboxylate (**17r**) were prepared respectively according to **Scheme S6**.

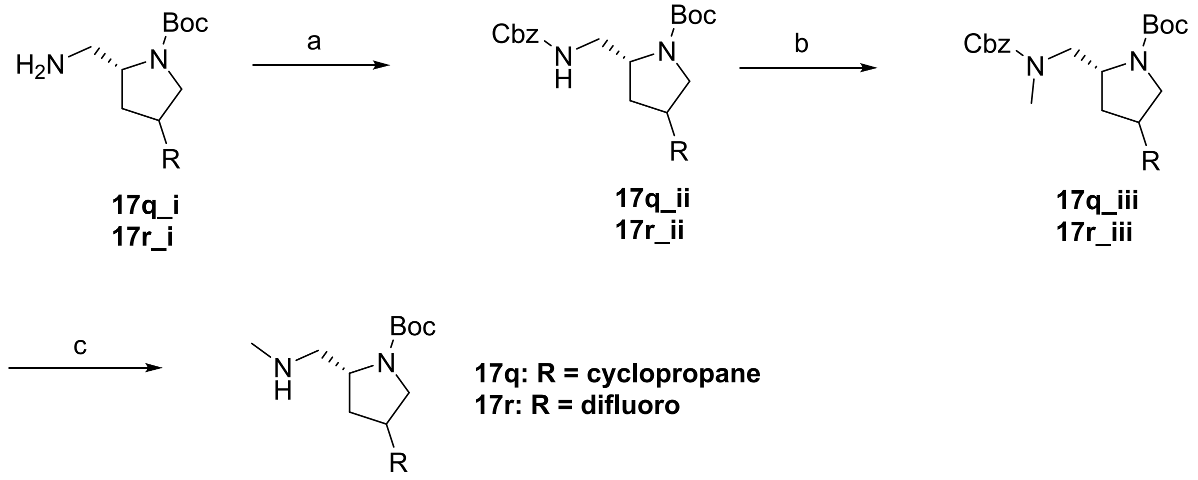

17q_i
17r_i

17q_ii
17r_ii

17q_iii
17r_iii

17q: R = cyclopropane
17r: R = difluoro

Scheme S6: a) CbzCl, K_2CO_3, THF, 0 °C–rt. (b) MeI, NaH, DMF, rt. (c) $H_{2(g)}$, Pd/C, MeOH, rt.

Preparation of *tert*-butyl (*S*)-6-((((benzyloxy)carbonyl)amino)methyl)-5-azaspiro[2.4]heptane-5-carboxylate (17q_ii)

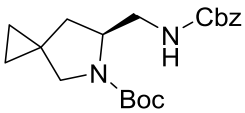

Prepared according to general Procedure B using commercially available *tert*-butyl (*S*)-6-(aminomethyl)-5-azaspiro[2.4]heptane-5-carboxylate (**17q_i**). The product was in the form of an oil (1370mg, 3.80mmol, 86%). **^{1}H NMR (600 MHz, $CDCl_3$)** δ 7.38 – 7.28 (m, 5H), 5.98 (s, 1H), 5.09 (s, 2H), 4.13 – 3.97 (m, 1H), 3.55 – 3.37 (m, 3H), 3.08 – 2.99 (m, 1H), 2.97 – 2.86 (m, 1H), 2.26 – 2.10 (m, 1H), 1.48 – 1.43 (m, 9H), 0.66 – 0.59 (m, 2H), 0.57 – 0.51 (m, 2H).**^{13}C NMR ($CDCl_3$)** δ 157.0, 156.0, 137.0, 128.6, 128.1, 128.0, 80.3, 66.8, 58.0, 54.4, 45.0, 37.9, 28.6, 22.8, 14.3.

Preparation of *tert*-butyl (*S*)-6-((((benzyloxy)carbonyl)(methyl)amino)methyl)-5-azaspiro[2.4]heptane-5-carboxylate (17q_iii)

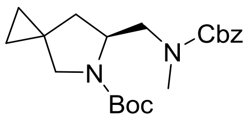

Prepared according to General Procedure C using tert-butyl (S)-6-((((benzyloxy)carbonyl)amino)methyl)-5-azaspiro[2.4]heptane-5-carboxylate (**17q_ii**) and anhydrous DMF instead of THF. The product was in the form of an oil (798mg, 2.13mmol, 59%). **^{1}H NMR (600 MHz, $CDCl_3$)** δ 7.36 – 7.27 (m, 5H), 5.15 – 5.06 (m, 2H), 4.14 – 4.01 (m, 1H), 3.85 – 3.56 (m, 1H), 3.53 – 3.15 (m, 2H), 3.04 – 2.83 (m, 5H), 2.17 – 1.99 (m, 1H), 1.50 – 1.40 (m, 9H), 0.64 – 0.28 (m, 4H); **^{13}C NMR ($CDCl_3$)** δ 157.0, 154.3, 136.6,128.6, 79.8, 67.4, 54.5, 54.0, 51.6, 36.8, 36.1, 28.6, 6.2.

Preparation of *tert*-butyl (S)-6-((methylamino)methyl)-5-azaspiro[2.4]heptane-5-carboxylate (17q)

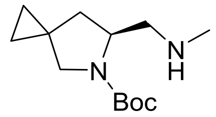

Prepared according to General Procedure D using tert-butyl (S)-6-((((benzyloxy)carbonyl)(methyl)amino)methyl)-5-azaspiro[2.4]heptane-5-carboxylate (**17q_iii**). The product was in the form of an oil and was immediately used for the subsequent reaction.

Preparation of *tert*-butyl (*S*)-2-((((benzyloxy)carbonyl)amino)methyl)-4,4-difluoropyrrolidine-1-carboxylate (17r_ii)

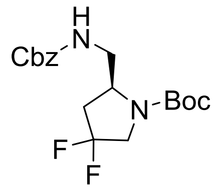

Prepared according to general Procedure B using commercially available *tert*-butyl (*S*)-2-(aminomethyl)-4,4-difluoropyrrolidine-1-carboxylate (**17r_i**). The product was in the form of an oil (1535mg, 4.15mmol, 98% yield). **^{1}H NMR (600 MHz, $CDCl_3$)** δ 7.37 – 7.29 (m, 5H), 5.66 (s, 1H), 5.10 (s, 2H), 4.18 – 4.09 (m, 1H), 3.95 – 3.73 (m, 1H), 3.61 (q, *J* = 12.6 Hz, 1H), 3.51 – 3.33 (m, 2H), 2.55 – 2.44 (m, 1H), 2.29 – 2.19 (m, 1H), 1.46 (s, 9H); **^{13}C NMR ($CDCl_3$)** δ 156.8, 155.0, 136.6, 128.6, 128.2, 126.8, 81.3, 66.9, 56.0, 53.7, 37.7, 28.4; **^{19}F NMR (565 MHz, $CDCl_3$)** δ -93.20 – -103.08 (m).

Preparation of *tert*-butyl (*S*)-2-((((benzyloxy)carbonyl)(methyl)amino)methyl)-4,4-difluoropyrrolidine-1-carboxylate (17r_iii)

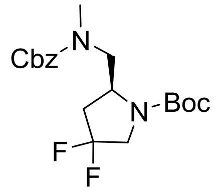

Prepared according to General Procedure C using tert-butyl (S)-2-((((benzyloxy)carbonyl)amino)methyl)-4,4-difluoropyrrolidine-1-carboxylate (**17q_ii**) and anhydrous DMF instead of THF. The product was in the form of an oil (951mg, 2.47mmol, 61%). **^{1}H NMR (600 MHz, $CDCl_3$)** δ 7.39 – 7.30 (m, 5H), 5.13 (s, 2H), 4.30 – 4.20 (m, 1H), 3.95 – 3.70 (m, 1H), 3.69 – 3.58 (m, 1H), 3.58 – 3.39 (m, 2H), 2.98 (s, 3H), 2.49 – 2.16 (m, 2H), 1.46 (s, 9H); **^{13}C NMR ($CDCl_3$)** δ 157.1, 154.0, 136.7, 128.2, 125.8, 80.8, 67.4, 54.2, 53.2, 51.9, 37.4, 35.1, 28.5; **^{19}F NMR (565 MHz, $CDCl_3$)** δ -93.75 (dt, *J* = 234.7, 17.9 Hz), -95.30 – -96.28 (m), -97.12

(dt, J = 234.5, 15.0 Hz), -99.02 (dt, J = 234.1, 13.4 Hz), -99.42 – -100.58 (m).

Preparation of *tert*-butyl (*S*)-4,4-difluoro-2-((methylamino)methyl)pyrrolidine-1-carboxylate (17r)

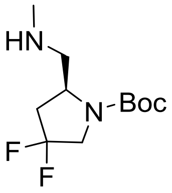

Prepared according to General Procedure D using *tert*-butyl (*S*)-2-((((benzyloxy)carbonyl)(methyl)amino)methyl)-4,4-difluoropyrrolidine-1-carboxylate (**17r_iii**). The product was in the form of an oil and was immediately used for the subsequent reaction.

Preparation of *tert*-butyl methyl(1-((methylamino)methyl)cyclopropyl)carbamate (17s) and *tert*-butyl methyl(1-((methylamino)methyl)cyclobutyl)carbamate(17t)

Tert-butyl methyl(1-((methylamino)methyl)cyclopropyl)carbamate (**17s**) and *tert*-butyl methyl(1-((methylamino)methyl)cyclobutyl)carbamate (**17t**) were prepared respectively according to Scheme S7.

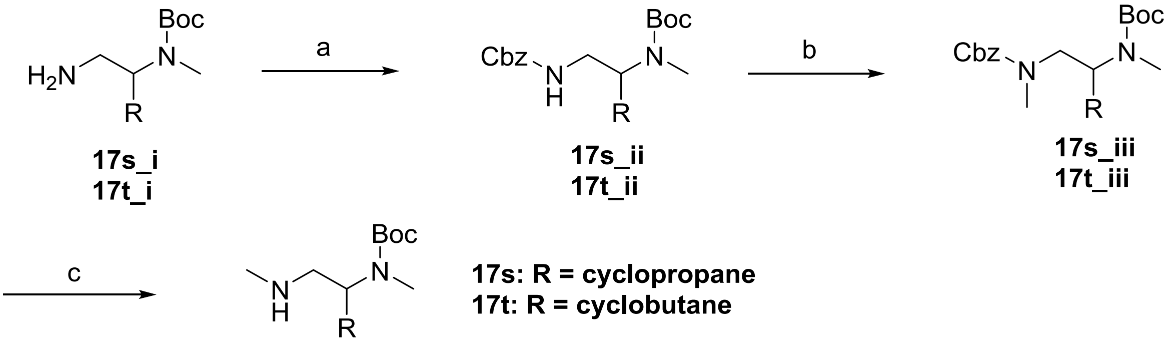

Scheme S7: (a) Cbz-Cl, K_2CO_3, THF, 0 °C–rt. (b) MeI, NaH, DMF, rt. (c) $H_{2(g)}$, Pd/C, MeOH, rt.

Preparation of *tert*-butyl (1-(aminomethyl)cyclopropyl)(methyl)carbamate (17s_ii)

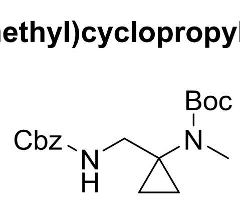

Prepared according to General Procedure B using commercially available *tert*-butyl (1-(aminomethyl)cyclopropyl)(methyl)carbamate (**17s_i**). The product was in the form of an oil (701 mg, 2.1 mmol, 84% yield). Used crude product immediately for the subsequent reaction.

Preparation of *tert*-butyl (1-((((benzyloxy)carbonyl)(methyl)amino)methyl)cyclopropyl)(methyl)carbamate (17s_iii)

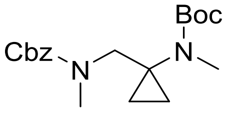

Prepared according to General Procedure C using *tert*-butyl (1-(aminomethyl)cyclopropyl)(methyl)carbamate (**17s_ii**) in DMF. The product was in the form of an oil (510 mg, 1.46 mmol, 70% yield. **^{1}H NMR (600 MHz, DMSO-d_6)** δ 7.41 – 7.26 (m, 5H), 5.09 – 4.94 (m, 2H), 3.32 (d, *J* = 13.8 Hz, 2H), 2.92 (d, *J* = 22.8 Hz, 3H), 2.68 (d, *J* = 47.6 Hz, 3H), 1.38 – 1.25 (m, 9H), 1.06 – 0.50 (m, 4H); **^{13}C NMR (151 MHz, DMSO-d_6)** δ 155.45 (d, *J* = 25.9 Hz), 155.23 (d, *J* = 9.0 Hz), 137.2, 137.0, 136.9, 128.4, 128.3, 127.8, 127.7, 127.7, 127.4, 78.4, 78.3, 78.2, 66.31 (d, *J* = 6.0 Hz), 66.02 (d, *J* = 13.9 Hz), 52.7, 52.4, 52.2, 52.1, 38.3, 35.6, 35.2, 35.1, 34.8, 34.36 (d, *J* = 8.2 Hz), 33.5, 33.4, 28.0, 16.1, 11.7; **HRMS** (ESI+) $[M+H]^+$ calc. $C_{19}H_{29}O_4N_2$, 349.2122, observed, 349.2129.

Preparation of *tert*-butyl methyl(1-((methylamino)methyl)cyclopropyl)carbamate (17s)

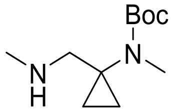

Prepared according to General Procedure D using *tert*-butyl (1-((((benzyloxy)carbonyl)(methyl)amino)methyl)cyclopropyl)(methyl)carbamate (**17s_iii**). The product was in the form of an oil (282 mg, 1.32 mmol, 90% yield). Used crude product immediately for the subsequent reaction.

Preparation of *tert*-butyl (1-((((benzyloxy)carbonyl)amino)methyl)cyclobutyl)(methyl)carbamate (17t_ii)

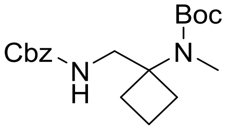

Prepared according to General Procedure B using commercially available *tert*-butyl (1-(aminomethyl)cyclobutyl)(methyl)carbamate (**17t_i**). The product was in the form of an oil (666 mg, 1.915 mmol, 82% yield). **^{1}H NMR (600 MHz, $CDCl_3$)** δ 7.36 – 7.27 (m, 5H), 5.09 (s, 2H), 3.50 – 3.45 (m, 2H), 2.67 (s, 3H), 2.28 – 2.18 (m, 2H), 2.04 (s, 2H), 1.77 – 1.70 (m, 2H), 1.43 (s, 9H); **^{13}C NMR (151 MHz, $CDCl_3$)** δ 157.1, 155.4, 136.8, 128.5, 128.0, 80.1, 66.6, 60.4, 46.2, 31.0, 30.4, 28.6, 14.0; **HRMS** (ESI+) $[M-H]^+$ calc. for $C_{19}H_{27}O_4N_2$, 347.1976, observed, 347.1982.

Preparation of *tert*-butyl (1-((((benzyloxy)carbonyl)(methyl)amino)methyl)cyclobutyl)(methyl)carbamate (17_iii)

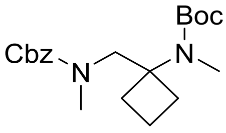

Prepared according to General Procedure C using *tert*-butyl (1-((((benzyloxy)carbonyl)amino)methyl)cyclobutyl)(methyl)carbamate (**17_ii**) in DMF. The product was in the form of an oil (424 mg, 1.172 mmol, 68% yield). **^{1}H NMR (400 MHz, $CDCl_3$)** δ 7.40 – 7.29 (m, 5H), 5.13 (s, 2H), 3.73 – 3.53 (m, 2H), 2.95 (d, *J* = 11.0 Hz, 3H), 2.68 (d, *J* = 25.2 Hz, 3H), 2.25 – 2.00 (m, 4H), 1.83 – 1.58 (m, 2H), 1.43 (s, 9H); **^{13}C NMR (151 MHz, $CDCl_3$) δ** 157.5, 156.8, 155.2, 137.0, 136.7, 128.6, 128.5, 128.2, 128.1, 127.9, 79.7, 67.6, 67.3, 61.9, 61.6, 53.3, 52.7, 52.5, 52.1, 37.6, 36.8, 31.4, 30.7, 30.6, 29.8, 28.7, 14.0, 13.9; **HRMS** (ESI+) $[M+Na]^+$ calc. for $C_{20}H_{30}O_4N_2Na$, 385.2095, observed, 385.2098.

Preparation of *tert*-butyl methyl(1-((methylamino)methyl)cyclobutyl)carbamate (17t)

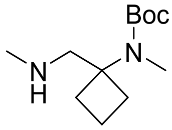

Prepared according to General Procedure D using *tert*-butyl (1-((((benzyloxy)carbonyl)(methyl)amino)methyl)cyclobutyl)(methyl)carbamate (**17t_iii**). The product was in the form of an oil (138 mg, 0.604 mmol, 89% yield). Used crude product immediately for the subsequent reaction.

Preparation of tert-butyl (2S,4S)-4-(dimethylamino)-2-((dimethylamino)methyl)pyrrolidine-1-carboxylate (17u)

Tert-butyl (2*S*,4*S*)-4-(dimethylamino)-2-((dimethylamino)methyl)pyrrolidine-1-carboxylate (**17u**) was prepared according to **Scheme S8**.

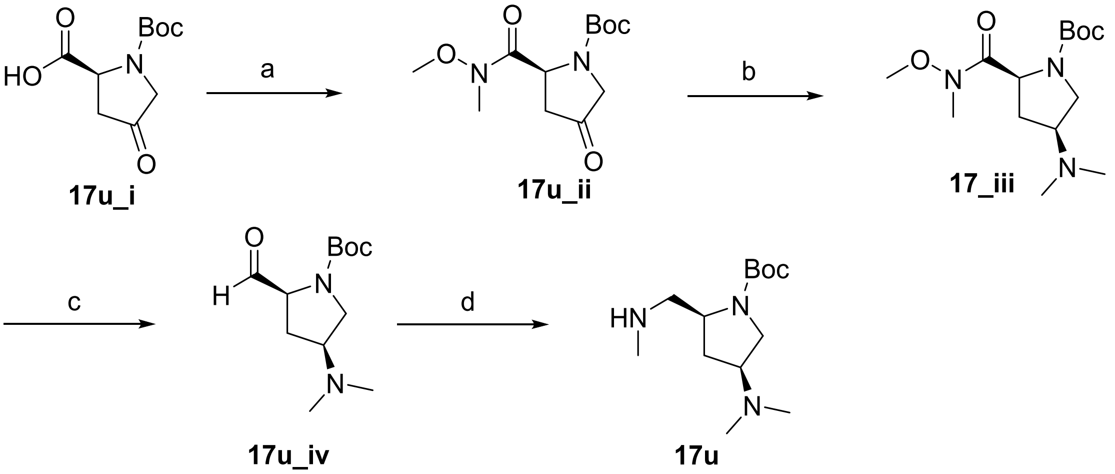

Scheme S8: (a) N, O-dimethyl hydroxylamine HCl, EDCI, HOBT, TEA, DCM, RT, 8h. (b) NMe_2 in MeOH, $NaBH_3CN$, AcOH, THF, RT, 2h. (c) $LiAlH_4$, THF, 0 °C, 30 min. (d) MeI, $NaBH(OAc)_3$, AcOH, DCM, rt, overnight.

Preparation of *tert*-butyl (*S*)-2-(methoxy(methyl)carbamoyl)-4-oxopyrrolidine-1-carboxylate (17u_ii)

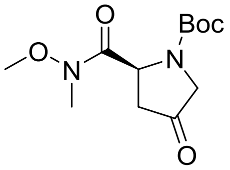

An oven-dried 250 mL 2-neck round bottom flask was charged with a stirrer bar and (*S*)-1-(tert-butoxycarbonyl)-4-oxopyrrolidine-2-carboxylic acid (**17u_i**) (2.50 g, 10.8 mmol) in DCM (80 mL) under argon. To the solution was then added EDCI (3.14 g, 16.36 mmol, 1.5 equiv.), HOBT (1.47 g, 10.9 mmol, 1 equiv.) and TEA (3.8 mL, 27.25 mmol, 2.5 equiv.) and stirred for 30 min at RT. N, O-dimethyl hydroxylamine HCl (1.28 g, 13.09 mmol, 1.2 equiv.) was then added and the reaction mixture was stirred for a further 8 h at RT. Upon completion, the reaction mixture was extracted with H_2O (100 mL x 2), followed by brine. After drying over magnesium sulfate and filtering, the solvent was reduced *in vacuo* and carried forward without further purification. **^{1}H NMR (400 MHz, $CDCl_3$)** δ 5.13 (dd, *J* = 56.4, 9.9 Hz, 1H), 4.05 – 3.87 (m, 2H), 3.82 (s, 3H), 3.20* (s, 3H), 2.98 – 2.79 (m, 1H), 2.52 – 2.39 (m, 1H), 1.47 (s, 9H), 1.45* (s, 9H); **^{13}C NMR (101 MHz, $CDCl_3$)** δ 209.3, 172.3, 154.6, 81.1, 77.4, 61.7, 54.6, 53.7, 41.0, 32.3, 28.5. One extra ^{13}C signal present and multiple peak splitting.

Preparation of *tert*-butyl (2S,4S)-4-(dimethylamino)-2-(methoxy(methyl)carbamoyl)pyrrolidine-1-carboxylate (17u_iii)

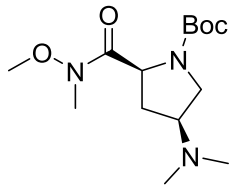

An oven-dried 100 mL 2-neck round bottom flask was charged with a stirrer bar and *tert*-butyl (*S*)-2-(methoxy(methyl)carbamoyl)-4-oxopyrrolidine-1-carboxylate (**17u_ii**) (1.00 g, 3.67 mmol) in THF (20 mL) under argon. Dimethylamine (2M in MeOH) (7.5 mL, 15 mmol, 4 equiv.), and AcOH (6.31 mL, 5.505 mmol, 1.5 equiv.) were then added, followed by the addition of $NaBH_3CN$. The reaction mixture was stirred at RT for 2 h. Finally, the THF was reduced *in vacuo* and the crude solid was dissolved in EtOAc, washed with saturated sodium bicarbonate and then brine. After drying over magnesium sulfate and filtering, the solvent was reduced *in vacuo* and the product was purified by column chromatography (1 – 4 % MeOH/DCM) affording a yellow solid (929 mg, 3.08 mmol). **^{1}H NMR (400 MHz, $CDCl_3$)** δ 4.62 (dt, *J* = 32.1, 8.5 Hz, 1H), 3.87 (dd, *J* = 10.2, 6.9 Hz, 1H), 3.75 (s, 3H), 3.69* (s, 3H), 3.31 – 3.21 (m, 1H), 3.18 (s, 3H), 2.90 – 2.70 (m, 1H), 2.55 – 2.42 (m, 1H), 2.29 (d, *J* = 8.0 Hz, 6H), 1.85 – 1.69 (m, 1H), 1.44 (s, 9H), 1.38* (s, 9H); **^{13}C NMR (101 MHz, $CDCl_3$)** δ 153.7, 80.2, 77.4, 63.8, 61.5, 56.4, 49.9, 43.9, 34.6, 32.6, 28.5. Multiple peak splitting in ^{13}C spectrum.

Preparation of *tert*-butyl (2*S*,4*S*)-4-(dimethylamino)-2-formylpyrrolidine-1-carboxylate (17u_iv)

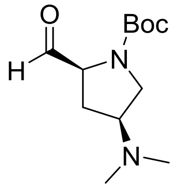

An oven-dried 50 mL 2-neck round bottom flask was charged with a stirrer bar and *tert*-butyl (2S,4S)-4-(dimethylamino)-2-(methoxy(methyl)carbamoyl)pyrrolidine-1-carboxylate (**17u_iii**) (400 mg, 1.33 mmol) in THF (5 mL) under argon. The solution was cooled to 0 °C and treated dropwise with $LiAlH_4$ (2M in THF) (0.8 mL, 1.59, 1.2 equiv.). After 30 min of stirring the reaction was quenched with H_2O and extracted with EtOAc (x 2). The organic layers were further washed with 15% NaOH, H_2O (x 3) and brine. After drying over magnesium sulfate and filtering, the solvent was reduced *in vacuo* and carried forward without further purification. **^{1}H NMR (400 MHz, $CDCl_3$)** δ 9.41 (d, *J* = 28.4, 3.0 Hz, 1H), 4.05 – 3.97 (m, 1H), 3.79 – 3.58 (m, 2H), 2.91 – 2.72 (m, 1H), 2.31 (d, *J* = 13.2 Hz, 6H), 2.27 – 2.19 (m, 1H), 2.05 – 1.93 (m, 1H), 1.54 – 1.40 (m, 9H).

Preparation of *tert*-butyl (2*S*,4*S*)-4-(dimethylamino)-2-((dimethylamino)methyl)pyrrolidine-1-carboxylate (17u)

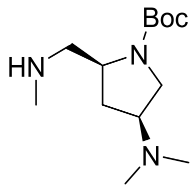

To a 2-neck round bottomed flask was added aldehyde **17u_iv** (300 mg, 1.24 mmol) which was dissolved in DCM/AcOH (2 mL + 0.1 mL) under an inert atmosphere. The solution was then treated with methylamine (33% wt in EtOH, 4 equiv.) and $NaBH(OAc)_3$ (5 equiv.). The reaction mixture was stirred at rt overnight. The mixture was then diluted with DCM and a solution of 10% Na_2CO_3 was added, followed by 2M NaOH to adjust the pH ~ 12. The mixture was extracted with DCM (3 x 25 mL). The combined organic phase was then washed with brine, dried and concentrated *in vacuo*. The crude product was used directly in the next step without further purification. **^{1}H NMR (400 MHz, $CDCl_3$)** δ 4.07 – 3.59 (m, 2H), 3.19 – 2.99 (m, 4H), 2.98 – 2.85 (m, 2H), 2.55 – 2.42 (m, 4H), 2.39 – 2.27 (m, 1H), 2.23 (s, 6H), 1.77 – 1.58 (m, 1H), 1.44 (s, 9H).

Preparation of di-*tert*-butyl (azanediylbis(ethane-2,1-diyl))bis(methylcarbamate) (17x)

Di-*tert*-butyl (azanediylbis(ethane-2,1-diyl))bis(methylcarbamate) (17x) was prepared according to **Scheme S9**.

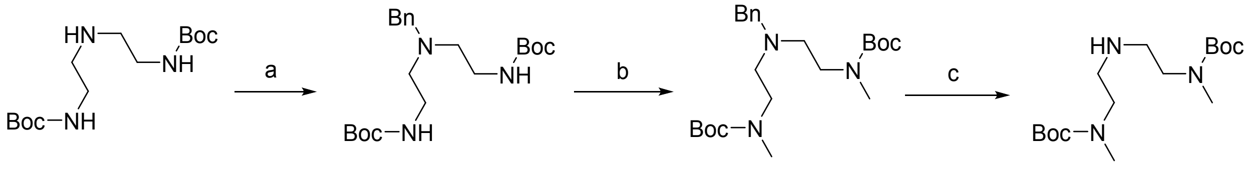

17x_i **17x_ii** **17x_iii** **17x**

Scheme S9: (a) BnBr, K_2CO_3, MeCN, rt. (b) MeI, NaH, THF, 0 °C–rt. (c) $H_{2(g)}$, Pd/C, MeOH, rt.

Preparation of di-*tert*-butyl ((benzylazanediyl)bis(ethane-2,1-diyl))dicarbamate (17x_ii)

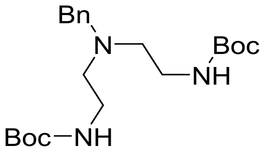

To an oven-dried 2-neck round bottom flask was added commercially available di-*tert*-butyl (azanediylbis(ethane-2,1-diyl))dicarbamate (**17x_i**) (1000 mg, 3.296 mmol) and anhydrous MeCN (30 mL). The solution was then treated with K_2CO_3 at room temperature and stirred for 15 minutes. Benzyl bromide was then added drop-wise and the reaction mixture was stirred overnight. Following filtration, the solvent was removed *in vacuo* and flash chromatography (20–50% ethyl acetate/hexane) afforded the product di-*tert*-butyl ((benzylazanediyl)bis(ethane-2,1-diyl))dicarbamate (**14**) (1260 mg, 3.202 mmol, 97%) as a clear viscous oil. **^{1}H NMR (600 MHz, DMSO-d_6)** δ 7.32 (d, *J* = 7.8 Hz, 2H), 7.29 – 7.25 (m, 2H), 7.24 – 7.18 (m, 1H), 6.66 – 6.62 (m,

1H), 3.55 (s, 2H), 2.99 (q, J = 6.3 Hz, 4H), 2.43 (t, J = 6.6 Hz, 4H), 1.36 (s, 18H); **^{13}C NMR (151 MHz, DMSO-d_6)** δ 155.6, 139.6, 128.6, 127.9, 126.7, 77.4, 58.0, 53.3, 38.0, 28.3.

Preparation of di-*tert*-butyl ((benzylazanediyl)bis(ethane-2,1-diyl))bis(methylcarbamate) (17x_iii)

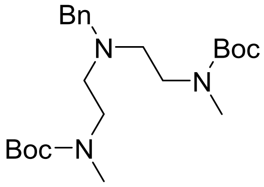

An oven-dried 100 mL 2-neck round bottom flask was charged with a stirrer bar and di-*tert*-butyl ((benzylazanediyl)bis(ethane-2,1-diyl))dicarbamate (**17x_ii**) (1.24 g, 3.15 mmol) under argon. Anhydrous THF (30 mL) was then added, and the mixture was cooled to 0 °C in a brine ice bath. Sodium hydride (502 mg, 12.604 mmol) was then added to the mixture. After stirring for 30 minutes, methyl iodide (1.18 mL, 18.906 mmol) was added dropwise to mixture at 0 °C, and the resulting mixture was allowed to warm to room temperature and stirred overnight (18 h). Afterward, the reaction mixture was quenched by adding a few drops of DI water and then pouring the mixture into saturated ammonium chloride solution (150 mL). The organic layer was then washed with brine and dried over anhydrous sodium sulfate. The solvent was removed *in vacuo* and the product was purified by flash chromatography (20–50% ethyl acetate/hexanes) to afford di-*tert*-butyl ((benzylazanediyl)bis(ethane-2,1-diyl))bis(methylcarbamate) (**15**) (1.169 g, 2.772 mmol, 88% yield) as an oil. **^{1}H NMR (600 MHz, DMSO-d_6)** δ 7.33 – 7.26 (m, 4H), 7.24 – 7.19 (m, 1H), 3.60 (s, 2H), 3.27 – 3.15 (m, 4H), 2.73 (s, 6H), 2.58 – 2.51 (m, 4H), 1.38 (s, 9H), 1.30* (s, 9H); **^{13}C NMR (151 MHz, DMSO-d_6)** δ 154.6, 139.5, 128.4, 128.1, 126.8, 78.3, 58.1, 51.2, 46.4, 45.67*, 34.4, 28.1*, 28.0.

Preparation of di-*tert*-butyl (azanediylbis(ethane-2,1-diyl))bis(methylcarbamate) (17x)

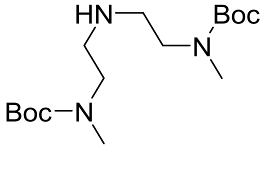

An oven-dried 100 mL round bottom flask was charged with a stirrer bar, di-*tert*-butyl ((benzylazanediyl)bis(ethane-2,1-diyl))bis(methylcarbamate) (**17x_iii**) (450.00 mg, 1.140 mmol), palladium on carbon (30 mg, 0.285 mmol), and anhydrous methanol (6 mL) under argon. A T-joint bearing a hydrogen balloon and Schlenk line connection was affixed to the flask. The reaction flask was then briefly evacuated and filled with hydrogen. This process of hydrogen filling was repeated 3 times. The mixture was then allowed to stir at room temperature under hydrogen for 5 h. Afterward, the mixture was filtered over celite, with the celite further washed with

methanol. The combined eluants were then concentrated *in vacuo* to afford a colorless oil that was immediately carried forward to the next reaction. **^{1}H NMR (600 MHz, $CDCl_3$)** δ 3.35 – 3.23 (m, 4H), 2.85 (s, 6H), 2.76 (s, 4H), 1.84 (br s, 1H), 1.43 (s, 18H); **^{13}C NMR (151 MHz, $CDCl_3$)** δ 156.2, 79.6, 49.1, 47.6, 35.1, 28.6. Multiple peak splitting in ^{13}C spectrum.

7.3 Preparation of Protected Prodrugs of Progesterone and Allopregnanolone C20-oxime

Boc-protected prodrugs of progesterone C20-oxime were prepared from intermediate **13** or **14** according to the general procedure E.

Preparation of *tert*-butyl (2-(cyclopropyl(((((*E*)-1-((8*S*,9*S*,10*R*,13*S*,14*S*,17*S*)-10,13-dimethyl-3-oxo-2,3,6,7,8,9,10,11,12,13,14,15,16,17-tetradecahydro-1*H*-cyclopenta[a]phenanthren-17-yl)ethylidene)amino)oxy)carbonyl)amino)ethyl)(methyl)carbamate (18b)

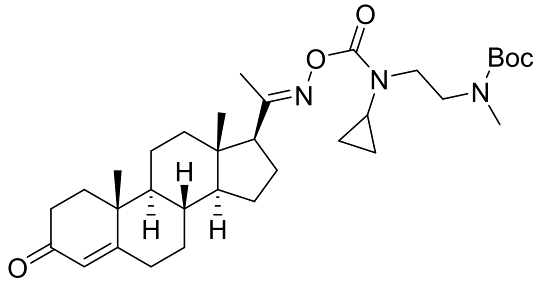

Prepared according to General Procedure E using amine nucleophile **17b**. Rf = 0.25 (20% ethyl acetate/DCM). The product was in the form of a white solid (673 mg, 1.181 mmol, 83% yield). **^{1}H NMR (400 MHz, DMSO-d_6)** δ 5.63 (s, 1H), 3.44– 3.27 (m, 4H), 2.78 (s, 3H), 2.72 – 2.52 (m, 1H), 2.47 – 2.32 (m, 3H), 2.30 – 2.10 (m, 3H), 2.02 –1.75 (m, 6H), 1.72 – 1.48 (m, 5H), 1.44 – 1.38 (m, 1H), 1.36 (s, 9H), 1.34 –1.16 (s, 3H), 1.15 (s, 3H), 1.05 – 0.89 (m, 2H), 0.81 – 0.70 (m, 2H), 0.64 (s, 3H), 0.63 – 0.60 (m, 2H). **^{13}C NMR (101 MHz, DMSO-d_6)** δ 198.0, 170.9, 163.9, 163.8, 154.7, 123.2, 78.6, 55.9, 54.7, 53.1, 45.2 (2C), 43.4, 38.2, 37.8, 35.1, 35.1, 33.6, 32.0, 31.6, 28.0, 27.9 (3C), 23.7, 22.6, 20.6, 16.9, 16.8, 13.1, 7.8 (2C). One ^{13}C signal not observed. **HRMS** (APCI+) $[M+H]^+$ calc. for $C_{33}H_{52}N_3O_5$, 570.3902, observed, 570.3909.

Preparation of *tert*-butyl (2-((((((*E*)-1-((8*S*,9*S*,10*R*,13*S*,14*S*,17*S*)-10,13-dimethyl-3-oxo-2,3,6,7,8,9,10,11,12,13,14,15,16,17-tetradecahydro-1*H*-cyclopenta[a]phenanthren-17-yl)ethylidene)amino)oxy)carbonyl)(isopropyl)amino)ethyl)(methyl)carbamate (18c)

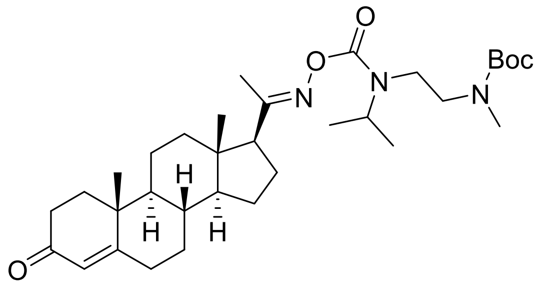

Prepared according to General Procedure E using amine nucleophile **17c**. Rf = 0.32 (20% ethyl acetate/DCM). The product was in the form of a white solid (630 mg, 1.102 mmol, 70% yield). **^{1}H NMR (400 MHz, DMSO-d$_6$)** δ 5.63 (s, 1H), 4.12 (br s, 1H), 3.35 – 3.26 (m, 2H), 3.24 – 3.16 (m, 2H), 2.84 – 2.77 (m, 3H), 2.47 – 2.32 (m, 3H), 2.29 – 2.10 (m, 3H), 2.02 – 1.75 (m, 6H), 1.70 – 1.50 (m, 5H), 1.38 (s, 9H), 1.35 – 1.29 (m, 2H), 1.29 – 1.16 (m, 2H), 1.15 (s, 3H), 1.14 – 1.07 (m, 6H), 1.04 – 0.88 (m, 2H), 0.65 (s, 3H); **^{13}C NMR (101 MHz, DMSO-d$_6$)** δ 198.0, 170.9, 163.8, 154.7, 153.4, 123.2, 78.6, 55.9, 54.7, 53.1, 47.8 (2C), 43.4, 38.2, 37.8, 35.1, 35.1, 34.8, 34.1, 33.6, 32.0, 31.6, 28.0 (3C), 23.7, 22.6, 20.6, 20.4, 20.1, 16.9 (2C), 13.1; **HRMS** (APCI+) $[M+H]^+$ calc. for $C_{33}H_{54}N_3O_5$, 572.4058, observed, 572.4066.

Preparation of tert-butyl (2-(cyclobutyl(((((*E*)-1-((8*S*,9*S*,10*R*,13*S*,14*S*,17*S*)-10,13-dimethyl-3-oxo-2,3,6,7,8,9,10,11,12,13,14,15,16,17-tetradecahydro-1*H*-cyclopenta[a]phenanthren-17-yl)ethylidene)amino)oxy)carbonyl)amino)ethyl)(methyl)carbamate (18d)

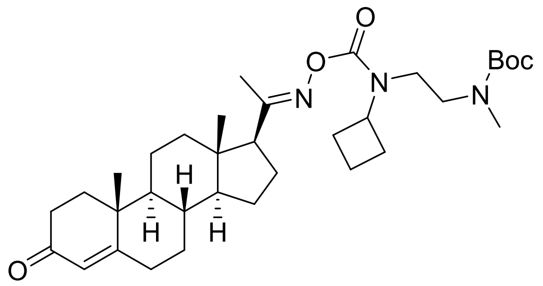

Prepared according to General Procedure E using amine nucleophile **17d**. Rf = 0.31 (20% ethyl acetate/DCM). The product was in the form of a white solid (473 mg, 0.810 mmol, 80% yield). **^{1}H NMR (400 MHz, DMSO-d$_6$)** δ 5.63 (s, 1H), 4.36 – 4.10 (m, 1H), 3.32 – 3.17 (m, 4H), 2.79 (s, 3H), 2.47 – 2.31 (m, 3H), 2.29 – 2.02 (m, 7H), 2.02 – 1.93 (m, 1H), 1.91 (s, 3H), 1.90 – 1.76 (m, 2H), 1.71 – 1.46 (m, 7H), 1.38 (s, 9H), 1.34 – 1.26 (m, 2H), 1.17 (s, 2H), 1.15 (s, 3H), 1.05 – 0.88 (m, 2H), 0.64 (s, 3H); **^{13}C NMR (101 MHz, DMSO-d$_6$)** δ 197.8, 170.6, 163.7, 154.5, 153.3, 123.2, 78.5, 56.0, 54.7, 53.1, 50.8, 47.4, 43.4, 38.1, 37.8, 35.1, 35.1, 34.2, 33.5, 32.0, 31.5, 28.0, 23.6,

22.5, 20.5, 16.9, 16.8, 14.0, 13.0. Two ^{13}C signal not observed. **HRMS** (APCI+) $[M+H]^+$ calc. for $C_{34}H_{54}N_3O_5$, 584.4058, observed, 584.4066.

Preparation of *tert*-butyl (2-(cyclopentyl(((((*E*)-1-((8*S*,9*S*,10*R*,13*S*,14*S*,17*S*)-10,13-dimethyl-3-oxo-2,3,6,7,8,9,10,11,12,13,14,15,16,17-tetradecahydro-1*H*-cyclopenta[a]phenanthren-17-yl)ethylidene)amino)oxy)carbonyl)amino)ethyl)(methyl)carbamate (18e)

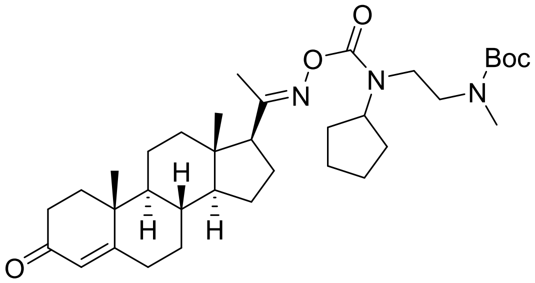

Prepared according to General Procedure E using amine nucleophile **17e**. Rf = 0.30 (10% ethyl acetate/DCM). The product was in the form of a white solid (520 mg, 0.870 mmol, 61% yield). **^{1}H NMR (400 MHz, DMSO-d$_6$)** δ 5.63 (s, 1H), 4.21 – 4.08 (m, 1H), 3.26 – 3.20 (m, 4H), 2.80 (s, 3H), 2.48 – 2.31 (m, 3H), 2.29 – 2.09 (m, 3H), 2.02 – 1.93 (m, 1H), 1.93 (s, 3H), 1.89 – 1.84 (m, 1H), 1.84 – 1.70 (m, 3H), 1.72 – 1.46 (m, 11H), 1.37 (s, 9H), 1.35– 1.16 (m, 4H), 1.15 (s, 3H), 1.06 – 0.88 (m, 2H), 0.64 (s, 3H); **^{13}C NMR (101 MHz, DMSO-d$_6$)** δ 198.0, 170.9, 163.6, 154.6, 153.6, 123.2, 78.7, 57.9, 55.9, 54.7, 53.1, 47.5, 43.4, 41.6, 38.2, 37.8, 35.1, 35.1, 34.9, 34.3, 33.6, 32.0, 31.6, 28.9, 28.0, 23.7, 23.2, 22.6, 20.6, 16.9, 13.1; **HRMS** (APCI+) $[M+H]^+$ calc. for $C_{35}H_{56}N_3O_5$, 598.4215, observed, 598.4215.

Preparation of *tert*-butyl (2-((((((*E*)-1-((8*S*,9*S*,10*R*,13*S*,14*S*,17*S*)-10,13-dimethyl-3-oxo-2,3,6,7,8,9,10,11,12,13,14,15,16,17-tetradecahydro-1*H*-cyclopenta[a]phenanthren-17-yl)ethylidene)amino)oxy)carbonyl)(isobutyl)amino)ethyl)(methyl)carbamate (18f)

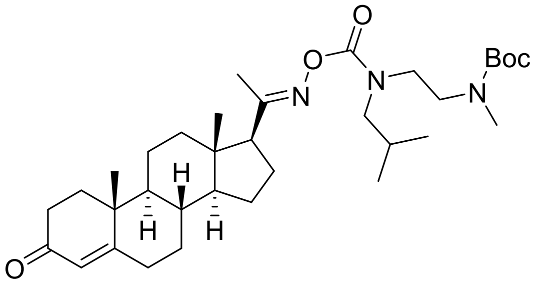

Prepared according to General Procedure E using amine nucleophile **17f**. Rf = 0.45 (20% ethyl acetate/DCM). The product was in the form of a white solid (330 mg, 0.564 mmol, 70% yield). **^{1}H NMR (400 MHz, DMSO-d$_6$)** δ 5.63 (s, 1H), 3.50 – 3.35 (m, 4H), 3.05 – 2.99 (m, 2H), 2.78 (s, 3H), 2.46 – 2.31 (m, 3H), 2.29 – 2.10 (m, 3H), 2.02 – 1.75 (m, 7H), 1.70 – 1.48 (m, 5H), 1.36 (s, 9H), 1.33 – 1.17 (m, 4H), 1.14 (s, 3H), 1.05 – 0.89 (m, 2H), 0.84 (d, *J* = 6.6 Hz, 6H), 0.63 (s, 3H); **^{13}C**

NMR (101 MHz, DMSO-d$_6$) δ 198.0, 170.8, 163.8, 154.6, 153.9, 123.2, 78.4, 55.9, 54.7, 53.1, 45.9, 44.8, 43.4, 38.2, 37.8, 35.1, 35.1, 33.8, 33.6, 31.9, 31.6, 27.9, 27.3, 26.6, 23.7, 22.5, 20.6, 19.8,16.9, 16.8, 13.1; **HRMS** (APCI+) $[M+H]^+$ calc. for $C_{34}H_{56}N_3O_5$, 586.4215, observed, 586.4225.

Preparation of *tert*-butyl (2-((((((*E*)-1-((8*S*,9*S*,10*R*,13*S*,14*S*,17*S*)-10,13-dimethyl-3-oxo-2,3,6,7,8,9,10,11,12,13,14,15,16,17-tetradecahydro-1*H*-cyclopenta[a]phenanthren-17-yl)ethylidene)amino)oxy)carbonyl)(2-(dimethylamino)ethyl)amino)ethyl)(methyl)carbamate (18g)

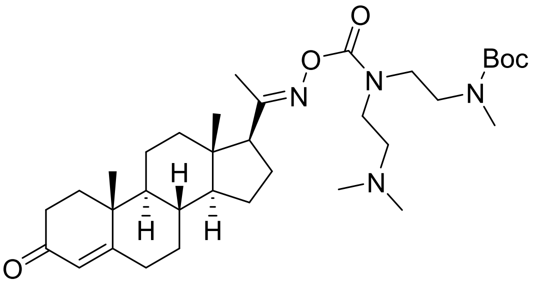

Prepared according to General Procedure E using amine nucleophile **17g**. Rf = 0.28 (40% ethyl acetate/DCM). The product was in the form of a white solid (365 mg, 0.608 mmol, 77% yield). **^{1}H NMR (400 MHz, DMSO-d$_6$)** δ 5.63 (s, 1H), 3.37 – 3.21 (m, 6H), 2.78 (s, 3H), 2.47 – 2.30 (m, 5H), 2.28 – 2.20 (m, 1H), 2.19 – 2.16 (m, 2H), 2.15 (s, 6H), 2.02 – 1.75 (m, 6H), 1.69 – 1.51 (m, 5H), 1.36 (s, 9H), 1.35 – 1.18 (m, 4H), 1.15 (s, 3H), 1.06 – 0.88 (m, 2H), 0.64 (s, 3H); **^{13}C NMR (101 MHz, DMSO-d$_6$)** δ 198.0, 170.9, 163.9, 154.7, 153.5, 123.2, 78.7, 56.9, 55.9, 54.7, 53.1, 45.4, 43.4, 38.2, 37.8, 35.1, 35.1, 33.6, 32.0, 31.6, 27.9, 23.7, 22.5, 20.6, 16.9, 16.8, 13.1. Two ^{13}C signals not observed; **HRMS** (APCI+) $[M+H]^+$ calc. for $C_{34}H_{57}N_4O_5$, 601.4324, observed, 601.4331.

Preparation of *tert*-butyl (2-((((((*E*)-1-((8*S*,9*S*,10*R*,13*S*,14*S*,17*S*)-10,13-dimethyl-3-oxo-2,3,6,7,8,9,10,11,12,13,14,15,16,17-tetradecahydro-1*H*-cyclopenta[a]phenanthren-17-yl)ethylidene)amino)oxy)carbonyl)(2-(pyrrolidin-1-yl)ethyl)amino)ethyl)(methyl)carbamate (18h)

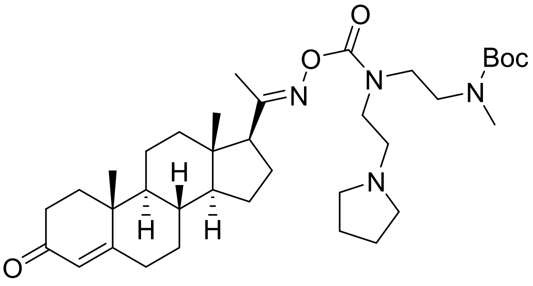

Prepared according to General Procedure E using amine nucleophile **17h**. The product was in

the form of a white solid (365 mg, 0.608 mmol, 77% yield). **^{1}H NMR (600 MHz, DMSO-d$_6$)** δ 5.64 (s, 1H), 3.42 – 3.22 (m, 6H), 2.79 (s, 3H), 2.59 – 2.51 (m, 2H), 2.50 – 2.40 (m, 5H), 2.39 – 2.32 (m, 2H), 2.29 – 2.21 (m, 1H), 2.22 – 2.11 (m, 2H), 2.01 – 1.94 (m, 1H), 1.94 – 1.77 (m, 5H), 1.71 – 1.63 (m, 6H), 1.63 – 1.50 (m, 3H), 1.45 – 1.28 (m, 2H), 1.37 (s, 9H), 1.26 – 1.18 (m, 2H), 1.15 (s, 3H), 1.06 – 0.89 (m, 2H), 0.64 (s, 3H); **^{13}C NMR (101 MHz, DMSO-d$_6$)** δ 198.0, 170.9, 163.9, 154.6, 153.5, 123.2, 78.4, 55.9, 54.7, 54.1, 53.7, 53.6, 53.1, 46.1, 45.4, 45.0, 43.4, 38.2, 37.8, 35.1, 35.1, 33.8, 33.6, 32.0, 31.6, 27.9 (2C), 23.7, 23.1, 22.5, 20.6, 16.9, 16.8, 13.1; **HRMS** (APCI+) [M+H]$^+$ calc. for $C_{36}H_{59}N_5O_4$, 627.4407, observed, 627.4487.

Preparation of *tert*-butyl (2-((((((*E*)-1-((8*S*,9*S*,10*R*,13*S*,14*S*,17*S*)-10,13-dimethyl-3-oxo-2,3,6,7,8,9,10,11,12,13,14,15,16,17-tetradecahydro-1*H*-cyclopenta[a]phenanthren-17-yl)ethylidene)amino)oxy)carbonyl)(3-morpholinopropyl)amino)ethyl)(methyl)carbamate (18i)

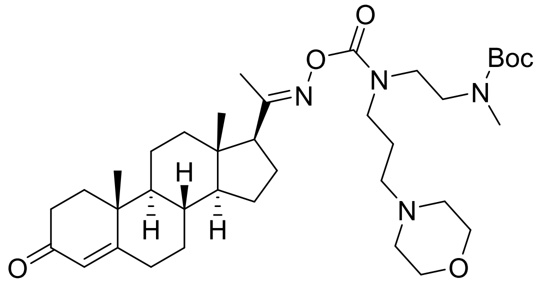

Prepared according to General Procedure E using amine nucleophile **17i**. product was in the form of a white solid (558 mg, 0.849 mmol, 84% yield). **^{1}H NMR (600 MHz, DMSO-d$_6$)** δ 5.63 (s, 1H), 3.58 – 3.53 (m, 4H), 3.40 – 3.28 (m, 5H), 3.26 – 3.15 (m, 2H), 2.79 (s, 3H), 2.45 – 2.30 (m, 6H), 2.28 – 2.21 (m, 3H), 2.19 – 2.12 (m, 2H), 2.00 – 1.94 (m, 1H), 1.92 – 1.88 (m, 3H), 1.88 – 1.83 (m, 1H), 1.83 – 1.77 (m, 1H), 1.69 – 1.59 (m, 5H), 1.60 – 1.50 (m, 2H), 1.40 – 1.28 (m, 2H), 1.36 (s, 9H), 1.25 – 1.17 (m, 2H), 1.15 (s, 3H), 1.03 – 0.90 (m, 2H), 0.63 (s, 3H); **^{13}C NMR (151 MHz, DMSO-d$_6$)** δ 198.0, 170.9, 163.6, 154.7, 153.4, 123.2, 78.4, 66.2, 55.9, 55.3, 54.7, 53.3, 53.1, 46.1, 45.5, 44.4, 43.4, 40.1, 38.2, 37.8, 35.1, 35.1, 33.6, 31.9, 31.6, 28.0, 27.9, 25.1, 24.3, 23.7, 22.5, 20.6, 16.9, 16.8, 13.1; **HRMS** (APCI+) [M+H]$^+$ calc. for $C_{37}H_{61}N_4O_6$, 657.4586, observed, 657.4593.

Preparation of *tert*-butyl (2-((((((*E*)-1-((8*S*,9*S*,10*R*,13*S*,14*S*,17*S*)-10,13-dimethyl-3-oxo-2,3,6,7,8,9,10,11,12,13,14,15,16,17-tetradecahydro-1*H*-cyclopenta[a]phenanthren-17-yl)ethylidene)amino)oxy)carbonyl)(2-morpholinoethyl)amino)ethyl)(methyl)carbamate (18j)

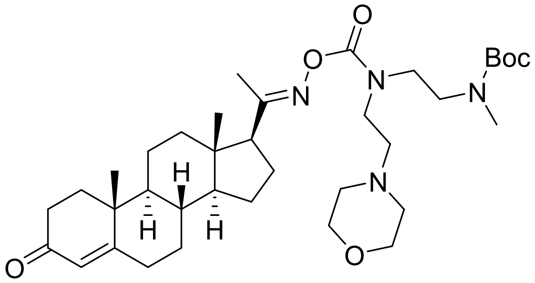

Prepared according to General Procedure E using amine nucleophile **17j**. The product was in the form of a white solid (330 mg, 0.608 mmol, 77% yield). **^{1}H NMR (600 MHz, DMSO-d_6)** δ 5.63 (s, 1H), 3.57 – 3.53 (m, 4H), 3.40 – 3.26 (m, 7H), 2.81 – 2.76 (m, 3H), 2.48 – 2.33 (m, 8H), 2.28 – 2.21 (m, 1H), 2.19 – 2.11 (m, 2H), 2.00 – 1.94 (m, 1H), 1.93 – 1.84 (m, 4H), 1.83 – 1.77 (m, 1H), 1.69 – 1.50 (m, 5H), 1.42 – 1.27 (m, 2H), 1.36 (s, 9H), 1.26 – 1.11 (m, 2H), 1.15 (s, 3H), 1.03 – 0.90 (m, 2H), 0.64 (s, 3H); **^{13}C NMR (151 MHz, DMSO-d_6)** δ 198.0, 170.7, 164.0, 154.7, 153.5, 123.2, 78.4, 66.2, 56.6, 56.1, 55.9, 54.7, 53.5, 53.1, 44.6, 43.4, 40.1, 38.2, 37.8, 35.1, 35.1, 33.8, 33.6, 31.9, 31.6, 28.0, 28.0, 23.7, 22.5, 20.6, 16.9, 16.8, 13.1; **HRMS** (APCI+) $[M+H]^+$ calc. for $C_{36}H_{59}N_4O_6$, 643.4429, observed, 643.4438.

Preparation of *tert*-butyl (2-((2-(1*H*-imidazol-1-yl)ethyl)(((((*E*)-1-((8*S*,9*S*,10*R*,13*S*,14*S*,17*S*)-10,13-dimethyl-3-oxo-2,3,6,7,8,9,10,11,12,13,14,15,16,17-tetradecahydro-1*H*-cyclopenta[a]phenanthren-17-yl)ethylidene)amino)oxy)carbonyl)amino)ethyl)(methyl)carbamate (18k)

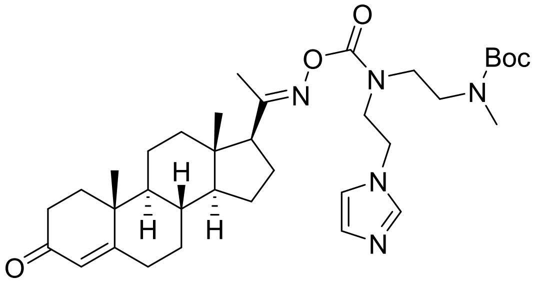

Prepared according to General Procedure E using amine nucleophile **17k**. The product was in the form of a white solid (370 mg, 0.593 mmol, 59% yield). **^{1}H NMR (600 MHz, DMSO-d_6)** δ 7.60 (s, 1H), 7.17 (d, J = 18.3 Hz, 1H), 6.88 (d, J = 13.1 Hz, 1H), 5.63 (s, 1H), 4.14 (t, J = 6.2 Hz, 2H), 3.57 – 3.37 (m, 2H), 3.56 – 3.21 (m, 3H), 3.20 – 2.97 (m, 2H), 2.74 (s, 3H), 2.45 – 2.32 (m, 3H), 2.28 – 2.21 (m, 1H), 2.19 – 2.10 (m, 2H), 2.01 – 1.94 (m, 1H), 1.93 – 1.77 (m, 5H), 1.68 – 1.50 (m, 5H), 1.42 – 1.27 (m, 2H), 1.33 (s, 9H), 1.25 – 1.17 (m, 1H), 1.15 (s, 3H), 1.03 – 0.90 (m, 2H), 0.63 (s, 3H); **^{13}C NMR (151 MHz, DMSO-d_6)** δ 198.0, 170.9, 164.4, 154.6, 153.5, 137.5, 128.6, 123.2, 119.5, 78.4, 55.9, 54.7, 53.1, 48.1, 45.8, 44.6, 43.4, 40.1, 38.2, 37.7, 35.1, 35.1, 33.6, 31.9, 31.6, 28.0, 27.9, 23.7, 22.5, 20.6, 16.9, 16.8, 13.1; **HRMS** (APCI+) $[M+H]^+$ calc. for

$C_{35}H_{54}N_5O_5$, 624.4120, observed, 624.4127.

Preparation of *tert*-butyl (*R*)-2-(((((((E)-1-((8S,9S,10R,13S,14S,17S)-10,13-dimethyl-3-oxo-2,3,6,7,8,9,10,11,12,13,14,15,16,17-tetradecahydro-1H-cyclopenta[a]phenanthren-17-yl)ethylidene)amino)oxy)carbonyl)(methyl)amino)methyl)pyrrolidine-1-carboxylate (18m)

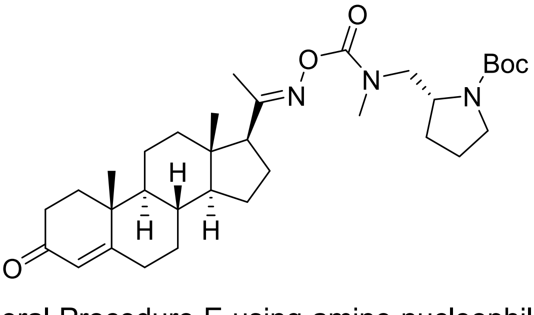

Prepared according to General Procedure E using amine nucleophile **17m**. The product was in the form of a white solid (387 mg, 0.679 mmol, 84% yield). **^{1}H NMR (400 MHz, DMSO-d_6)** δ 5.63 (s, 1H), 4.06 – 3.91 (m, 1H), 3.32 (s, 2H), 3.29 – 3.15 (m, 3H), 2.95 – 2.79 (m, 3H), 2.47 – 2.31 (m, 3H), 2.28 – 2.21 (m, 1H), 2.20 – 2.09 (m, 2H), 2.02 – 1.93 (m, 1H), 1.90 (s, 3H), 1.87 – 1.75 (m, 5H), 1.73 – 1.48 (m, 5H), 1.38 (s, 9H), 1.45 – 1.27 (m, 2H), 1.27 – 1.08 (m, 2H), 1.15 (s, 3H), 1.06 – 0.88 (m, 2H), 0.64 (s, 3H); **^{13}C NMR (101 MHz, DMSO-d_6)** δ 198.0, 170.9, 163.9, 153.5, 123.2, 78.3, 55.9, 54.8, 54.7, 53.1, 51.3, 50.7, 45.7, 43.4, 38.2, 37.8, 35.1, 35.1, 33.6, 32.0, 31.6, 28.0, 27.8, 23.7, 23.1, 22.5, 22.1, 20.6, 16.9, 16.7, 13.1; **HRMS** (APCI+) $[M+H]^+$ calc. for $C_{33}H_{52}N_3O_5$, 570.3902, observed, 570.3907.

Preparation of *tert*-butyl (*S*)-2-(2-((((((*E*)-1-((8*S*,9*S*,10*R*,13*S*,14*S*,17*S*)-10,13-dimethyl-3-oxo-2,3,6,7,8,9,10,11,12,13,14,15,16,17-tetradecahydro-1*H*-cyclopenta[a]phenanthren-17-yl)ethylidene)amino)oxy)carbonyl)(methyl)amino)ethyl)pyrrolidine-1-carboxylate (18n)

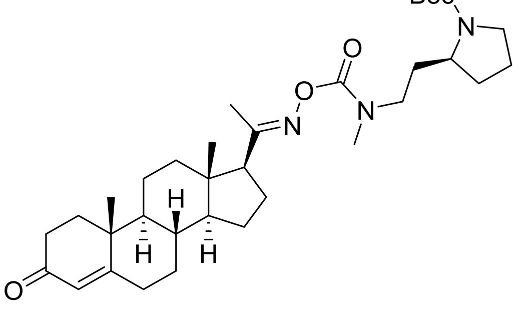

Prepared according to General Procedure E using amine nucleophile **17n**. The product was in the form of an off-white solid (887 mg, 1.519 mmol, 92% yield). **^{1}H NMR (600 MHz, DMSO-d_6)** δ 5.63 (s, 1H), 3.64 (s, 1H), 3.30 – 3.07 (m, 4H), 2.92 – 2.81 (m, 3H), 2.44 – 2.32 (m, 3H), 2.28 – 2.21 (m, 1H), 2.20 – 2.11 (m, 2H), 2.01 – 1.70 (m, 11H), 1.68 – 1.43 (m, 7H), 1.38 (s, 9H), 1.38

– 1.28 (m, 2H), 1.24 – 1.16 (m, 1H), 1.15 (s, 3H), 1.03 – 0.90 (m, 2H), 0.63 (s, 3H); **^{13}C NMR (151 MHz, DMSO-d$_6$)** δ 198.0, 170.9, 163.8, 153.6, 153.4, 123.2, 78.1, 55.9, 54.7, 54.6, 53.1, 46.1, 45.9, 45.8, 43.4, 38.2, 37.8, 35.1, 35.1, 33.6, 32.0, 31.6, 30.1, 29.5, 28.2 (3C), 23.7, 23.4, 22.5, 20.6, 16.9, 16.7, 13.1; **HRMS** (APCI+) [M+H]$^+$ calc. for $C_{34}H_{54}N_3O_5$, 584.4058, observed, 584.4067.

Preparation of *tert*-butyl (*S*)-2-((((((*E*)-1-((8S,9S,10R,13S,14S,17S)-10,13-dimethyl-3-oxo-2,3,6,7,8,9,10,11,12,13,14,15,16,17-tetradecahydro-1H-cyclopenta[a]phenanthren-17-yl)ethylidene)amino)oxy)carbonyl)(methyl)amino)methyl)azetidine-1-carboxylate (18o)

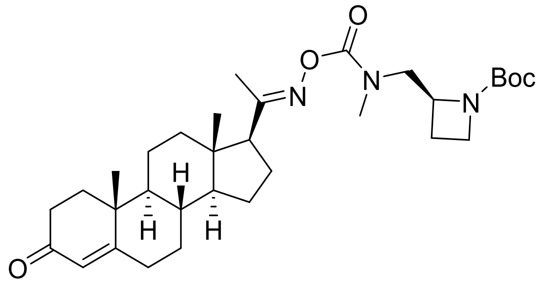

Prepared according to General Procedure E using amine nucleophile **17o**. Rf = 0.30 (30% ethyl acetate/DCM). The product was in the form of a white solid (299 mg, 0.538 mmol, 86% yield). **^{1}H NMR (400 MHz, DMSO-d$_6$)** δ 5.63 (s, 1H), 4.39 – 4.28 (m, 1H), 3.77 – 3.60 (m, 3H), 3.39 – 3.26 (m, 3H), 2.96 – 2.87 (m, 2H), 2.47 – 2.30 (m, 3H), 2.30 – 2.08 (m, 4H), 2.02 – 1.93 (m, 1H), 1.90 (s, 3H), 1.88 – 1.76 (m, 2H), 1.71 – 1.47 (m, 5H), 1.35 (s, 9H), 1.41 – 1.28 (m, 2H), 1.26 – 1.18 (m, 2H), 1.15 (s, 3H), 1.06 – 0.88 (m, 2H), 0.64 (s, 3H); **^{13}C NMR (101 MHz, DMSO-d$_6$)** δ 198.0, 170.9, 163.8, 155.7, 153.8, 123.2, 78.6, 59.5, 55.9, 54.7, 53.1, 52.6, 46.2, 43.4, 38.2, 37.8, 35.1, 35.1, 33.6, 32.0, 31.6, 28.0, 23.7, 22.5, 20.6, 19.5, 16.9, 16.7, 13.1. One ^{13}C signal not observed; **HRMS** (APCI+) [M+H]$^+$ calc. for $C_{32}H_{50}N_3O_5$, 556.3745, observed, 556.3751.

Preparation of *tert*-butyl (*R*)-2-((*S*)-1-((((((*E*)-1-((8S,9S,10R,13S,14S,17S)-10,13-dimethyl-3-oxo-2,3,6,7,8,9,10,11,12,13,14,15,16,17-tetradecahydro-1H-cyclopenta[a]phenanthren-17-yl)ethylidene)amino)oxy)carbonyl)(methyl)amino)ethyl)pyrrolidine-1-carboxylate (18p)

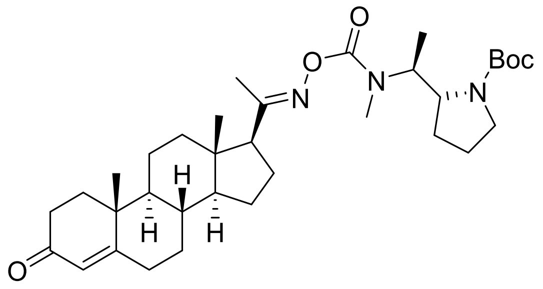

Prepared according to General Procedure E using amine nucleophile **17p**. The product was in the form of a white solid (244 mg, 0.419 mmol, 69% yield). **^{1}H NMR (600 MHz, CDCl$_3$)** δ 5.72 (s,

1H), 4.23 – 4.11 (m, 1H), 4.04 – 3.85 (m, 1H), 3.52 – 3.24 (m, 2H), 2.83 (s, 3H), 2.46 – 2.22 (m, 6H), 2.06 – 1.97 (m, 2H), 1.92 (d, *J* = 22.1 Hz, 4H), 1.89 – 1.63 (m, 8H), 1.63 – 1.50 (m, 2H), 1.50 – 1.38 (m, 10H), 1.34 (dt, *J* = 12.9, 6.3 Hz, 1H), 1.29 – 1.10 (m, 7H), 1.09 – 1.00 (m, 1H), 0.96 (ddd, *J* = 12.3, 10.7, 4.1 Hz, 1H), 0.72 (s, 3H); **^{13}C NMR (151 MHz, CDCl$_3$)** δ 199.6, 171.2, 164.9, 164.7, 155.8, 155.5, 155.2, 155.1, 124.0, 79.7, 79.4, 59.0, 58.6, 57.0, 56.9, 55.5, 55.5, 53.8, 53.6, 46.6, 46.0, 44.1, 38.7, 38.7, 38.6, 35.9, 35.8, 34.1, 32.9, 32.0, 31.8, 28.6, 28.0, 27.9, 24.2, 23.6, 23.1, 22.7, 21.1, 21.1, 17.5, 17.2, 15.8, 15.1, 13.6; **HRMS** (APCI+) $[M+H]^+$ calc. for $C_{34}H_{54}O_5N_3$, 584.4058, observed, 584.4067.

Preparation of *tert*-butyl (*R*)-6-((((((*E*)-1-((8*S*,9*S*,10*R*,13*S*,14*S*,17*S*)-10,13-dimethyl-3-oxo-2,3,6,7,8,9,10,11,12,13,14,15,16,17-tetradecahydro-1H-cyclopenta[a]phenanthren-17-yl)ethylidene)amino)oxy)carbonyl)(methyl)amino)methyl)-5-azaspiro[2.4]heptane-5-carboxylate (18q)

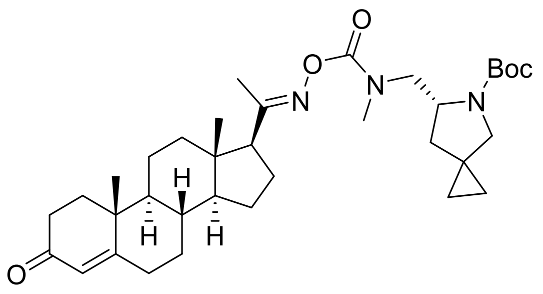

Prepared according to General Procedure E using amine nucleophile **17q**. The product was in the form of a white solid (266 mg, 0.437 mmol, 72% yield). **^{1}H NMR (600 MHz, CDCl$_3$)** δ 5.73 (s, 1H), 4.22 – 3.98 (m, 1H), 3.96 – 3.76 (m, 1H), 3.69 – 3.11 (m, 3H), 3.01 (d, *J* = 21.1 Hz, 3H), 2.98 – 2.88 (m, 1H), 2.46 – 2.25 (m, 6H), 2.22 – 2.06 (m, 1H), 2.04 – 1.98 (m, 1H), 1.94 (s, 3H), 1.88 – 1.81 (m, 1H), 1.80 – 1.65 (m, 4H), 1.63 – 1.51 (m, 3H), 1.50 – 1.43 (m, 9H), 1.43 – 1.39 (m, 1H), 1.34 (td, *J* = 12.6, 4.0 Hz, 1H), 1.29 – 1.21 (m, 1H), 1.18 (s, 3H), 1.17 – 1.10 (m, 1H), 1.09 – 1.00 (m, 1H), 0.97 (ddd, *J* = 12.3, 10.7, 4.1 Hz, 1H), 0.71 (s, 3H), 0.65 – 0.45 (m, 4H); **^{13}C NMR (151 MHz, CDCl$_3$)** δ 199.6, 171.3, 171.3, 165.0, 164.7, 164.5, 155.7, 155.5, 154.7, 154.3, 124.0, 79.7, 79.4, 56.9, 56.7, 56.5, 55.5, 54.5, 54.1, 53.9, 51.9, 51.0, 44.2, 38.7, 38.7, 36.8, 36.7, 36.0, 35.9, 35.8, 35.5, 34.8, 34.4, 34.1, 32.9, 32.0, 28.6, 24.3, 23.1, 21.1, 20.2, 19.5, 17.5, 17.1, 14.9, 13.6, 6.2, 5.8; **HRMS** (APCI+) $[M+H]^+$ calc. for $C_{35}H_{54}O_5N_3$, 596.4058, observed, 596.4075.

Preparation of *tert*-butyl (*R*)-2-((((((*E*)-1-((8*S*,9*S*,10*R*,13*S*,14*S*,17*S*)-10,13-dimethyl-3-oxo-2,3,6,7,8,9,10,11,12,13,14,15,16,17-tetradecahydro-1H-cyclopenta[a]phenanthren-17-yl)ethylidene)amino)oxy)carbonyl)(methyl)amino)methyl)-4,4-difluoropyrrolidine-1-carboxylate (18r)

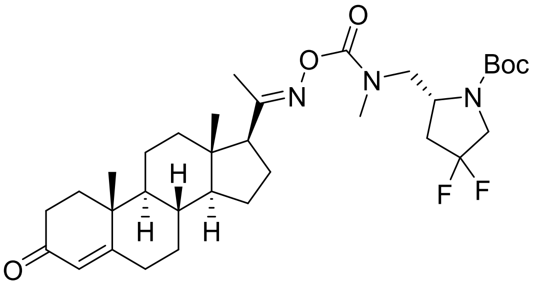

Prepared according to General Procedure E using amine nucleophile **17r**. The product was in the form of a white solid (242 mg, 0.40 mmol, 66% yield). **^{1}H NMR (600 MHz, CDCl$_3$)** δ 5.70 (s, 1H), 4.25 (s, 1H), 3.94 – 3.42 (m, 4H), 2.99 (s, 3H), 2.50 – 2.21 (m, 8H), 2.04 – 1.97 (m, 1H), 1.92 (s, 3H), 1.90 – 1.84 (m, 2H), 1.79 – 1.63 (m, 3H), 1.55 (dtd, *J* = 25.7, 12.3, 3.6 Hz, 2H), 1.48 – 1.37 (m, 10H), 1.35 – 1.20 (m, 2H), 1.16 (s, 3H), 1.15 – 1.10 (m, 1H), 1.08 – 0.99 (m, 1H), 0.98 – 0.92 (m, 1H), 0.70 (s, 3H); **^{13}C NMR (151 MHz, CDCl$_3$)** δ 199.6, 171.2, 164.9, 156.01 – 154.62 (m), 154.46 – 152.71 (m), 131.22 – 124.04 (m), 124.0, 80.95 (t, *J* = 37.6 Hz), 56.8, 55.46, 54.87 – 52.86 (m), 52.2, 51.2, 50.2, 44.1, 38.7, 38.6, 37.5, 35.8 (d, *J* = 4.5 Hz), 35.6, 34.8, 34.0, 32.9, 31.9, 28.4, 24.2, 23.1, 21.1, 17.5, 17.0, 13.6; **^{19}F NMR (565 MHz, CDCl$_3$)** δ -91.46 – -102.00 (m); **HRMS** (APCI+) [M+H]$^+$ calc. for $C_{33}H_{50}O_5N_3F_2$, 606.3713, observed, 606.3726.

Preparation of *tert*-butyl (1-((((((*E*)-1-((8*S*,9*S*,10*R*,13*S*,14*S*,17*S*)-10,13-dimethyl-3-oxo-2,3,6,7,8,9,10,11,12,13,14,15,16,17-tetradecahydro-1H-cyclopenta[a]phenanthren-17-yl)ethylidene)amino)oxy)carbonyl)(methyl)amino)methyl)cyclopropyl)(methyl)carbamate (18s)

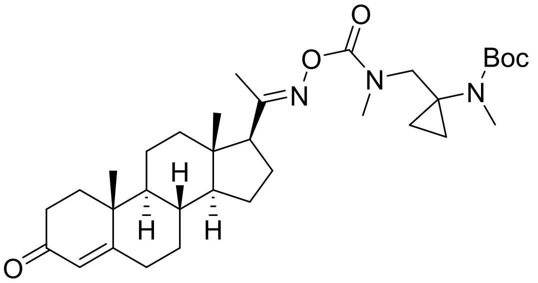

Prepared according to General Procedure E using amine nucleophile **17s**. The product was in the form of a white solid (248 mg, 0.435 mmol, 72% yield**)**. **^{1}H NMR (600 MHz, DMSO-d$_6$)** δ 5.63 (s, 1H), 2.92 (d, *J* = 30.2 Hz, 4H), 2.74 (s, 3H), 2.39 (qd, *J* = 17.0, 6.7 Hz, 4H), 2.20 (dd, *J* = 54.0, 15.3 Hz, 5H), 1.97 – 1.77 (m, 7H), 1.70 – 1.51 (m, 6H), 1.35 (d, *J* = 22.2 Hz, 9H), 1.26 – 1.19 (m, 2H), 1.15 (s, 3H), 1.03 – 0.90 (m, 4H), 0.63 (s, 3H); **^{13}C NMR (151 MHz, DMSO-d$_6$)** δ 198.0,

170.9, 155.2, 123.2, 55.9, 54.7, 53.1, 38.2, 35.1(2C), 33.6, 31.9, 31.6, 28.0, 23.7, 22.5, 20.8, 16.9, 13.1. One ^{13}C signal not observed ; **HRMS** (APCI+) $[M+H]^+$ calc. for $C_{33}H_{52}O_5N_3$, 570.3902, observed, 570.3912.

Preparation of *tert*-butyl (1-((((((*E*)-1-((8*S*,9*S*,10*R*,13*S*,14*S*,17*S*)-10,13-dimethyl-3-oxo-2,3,6,7,8,9,10,11,12,13,14,15,16,17-tetradecahydro-1H-cyclopenta[a]phenanthren-17-yl)ethylidene)amino)oxy)carbonyl)(methyl)amino)methyl)cyclobutyl)(methyl)carbamate (18t)

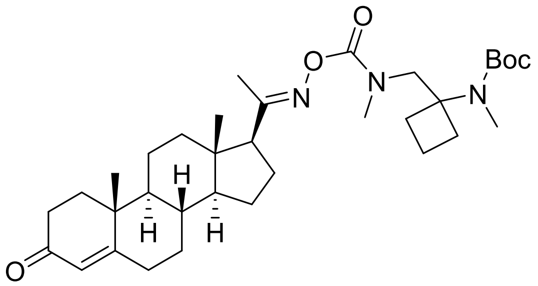

Prepared according to General Procedure E using amine nucleophile **17t**. The product was in the form of a white solid (262 mg, 0.449 mmol, 74% yield). **^{1}H NMR (400 MHz, $CDCl_3$)** δ 5.72 (d, J = 1.7 Hz, 1H), 3.76 – 3.52 (m, 2H), 2.95 (d, J = 13.0 Hz, 3H), 2.73 (s, 3H), 2.49 – 2.07 (m, 10H), 2.05 – 1.98 (m,1H), 1.93 (s, 4H), 1.89 – 1.64 (m, 7H), 1.54 (tdd, J = 23.2, 10.2, 3.9 Hz, 2H), 1.43 (s, 9H), 1.39 – 1.21 (m, 2H), 1.17 (s, 4H), 1.08 – 0.91 (m, 2H), 0.72 (s, 3H); **^{13}C NMR (151 MHz, $CDCl_3$)** δ 199.6, 171.2, 164.5 (d, *J* = 19.5 Hz), 156.1, 155.6 – 154.2 (m), 124.0, 80.8 – 78.5 (m), 62.6 – 61.2 (m), 60.5, 56.9, 55.5, 53.8, 53.6, 52.8 (d, *J* = 32.5 Hz), 44.1, 38.7, 38.6, 37.6, 36.5, 35.8 (d, *J* = 4.2 Hz), 34.1, 32.9, 31.0, 31.3, 30.8, 28.7, 24.3, 23.2, 21.1, 17.5, 17.1, 14.2, 13.9, 13.6; **HRMS** (ESI+) $[M+Na]^+$ calc. for $C_{34}H_{53}O_5N_3Na$, 606.3877, observed, 606.3879.

Preparation of *tert*-butyl (2*S*,4*S*)-2-((((((*E*)-1-((8*S*,9*S*,10*R*,13*S*,14*S*,17*S*)-10,13-dimethyl-3-oxo-2,3,6,7,8,9,10,11,12,13,14,15,16,17-tetradecahydro-1H-cyclopenta[a]phenanthren-17-yl)ethylidene)amino)oxy)carbonyl)(methyl)amino)methyl)-4-(dimethylamino)pyrrolidine-1-carboxylate (18u)

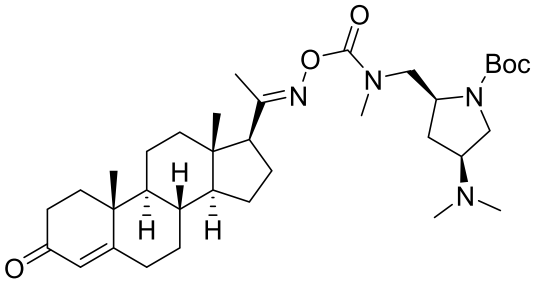

Prepared according to General Procedure E using amine nucleophile **17u**. Rf = 0.22 (50% ethyl acetate/DCM). The product was in the form of a white solid (363 mg, 0.6592 mmol, 77% yield).

^{1}H NMR (600 MHz, DMSO-d$_6$) δ 5.63 (s, 1H), 4.11 – 3.91 (m, 1H), 3.81 – 3.61 (m, 1H), 3.57 – 3.40 (m, 1H), 3.32 (s, 2H), 2.97 – 2.80 (m, 4H), 2.45 – 2.33 (m, 4H), 2.28 – 2.21 (m, 1H), 2.21 – 2.13 (m, 3H), 2.11 (s, 6H), 2.01 – 1.93 (m, 1H), 1.93 (s, 3H), 1.87 – 1.83 (m, 1H), 1.82 – 1.77 (m, 1H), 1.69 – 1.50 (m, 5H), 1.38 (s, 9H), 1.42 – 1.27 (m, 2H), 1.26 – 1.18 (m, 1H), 1.19 – 1.10 (m, 1H), 1.15 (s, 3H), 1.03 – 0.90 (m, 2H), 0.63 (s, 3H); **^{13}C NMR (151 MHz, DMSO-d$_6$)** δ 198.0, 170.9, 163.8, 153.5, 123.2, 78.9, 63.8, 55.9, 54.7, 53.1, 52.4, 51.0, 49.8, 44.2, 43.4, 40.1, 38.2, 37.8, 35.4, 35.1, 35.1, 34.3, 33.6, 31.9, 31.6, 28.1, 23.7, 22.5, 20.6, 16.9, 16.7, 13.1; **HRMS** (APCI+) $[M+H]^+$ calc. for $C_{35}H_{57}N_4O_5$, 613.4324, observed, 613.4330.

Preparation of *tert*-butyl 5-((((((*E*)-1-((8*S*,9*S*,10*R*,13*S*,14*S*,17*S*)-10,13-dimethyl-3-oxo-2,3,6,7,8,9,10,11,12,13,14,15,16,17-tetradecahydro-1H-cyclopenta[a]phenanthren-17-yl)ethylidene)amino)oxy)carbonyl)(methyl)amino)methyl)-1H-imidazole-1-carboxylate (18v)

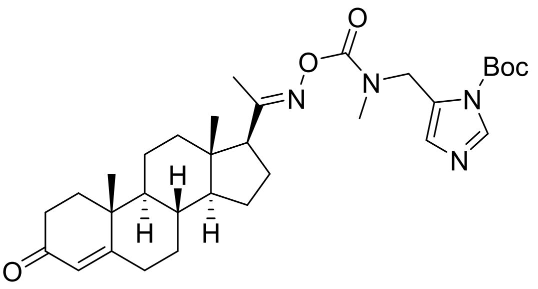

Prepared according to General Procedure E using **17v**. Due to Boc-cleavage of the product, the intermediate (**18v**) was carried forward without further purification or characterization.

Preparation of *tert*-butyl (2-(cyclopropyl(((((*E*)-1-((8*S*,9*S*,10*R*,13*S*,14*S*,17*S*)-10,13-dimethyl-3-oxo-2,3,6,7,8,9,10,11,12,13,14,15,16,17-tetradecahydro-1*H*-cyclopenta[a]phenanthren-17-yl)ethylidene)amino)oxy)carbonyl)amino)ethyl)(methyl)carbamate (18w)

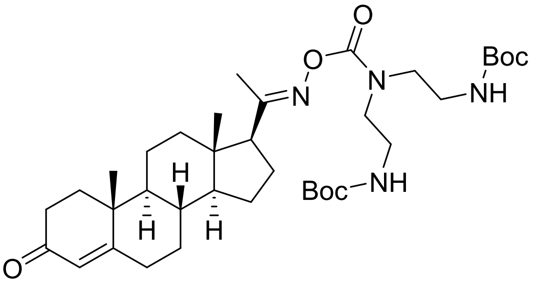

Prepared according to General Procedure E using commercially available di-*tert*-butyl (azanediylbis(ethane-2,1-diyl))dicarbamate **17w**. Rf = 0.30 (20% ethyl acetate/DCM). The product was in the form of a white solid (405 mg, 0.615 mmol, 76% yield). **^{1}H NMR (600 MHz, DMSO-d$_6$)** δ 6.94 (br s, 1H), 6.87 (br s, 1H), 5.63 (s, 1H), 3.27 – 3.19 (m, 4H), 3.09 – 3.03 (m, 4H), 2.45 – 2.32 (m, 3H), 2.28 – 2.21 (m, 1H), 2.19 – 2.11 (m, 2H), 2.01 – 1.94 (m, 1H), 1.91 (s,

3H), 1.88 – 1.77 (m, 2H), 1.69 – 1.50 (m, 5H), 1.36 (s, 18H), 1.33 – 1.19 (m, 4H), 1.15 (s, 3H), 1.03 – 0.90 (m, 2H), 0.65 (s, 3H); **^{13}C NMR (151 MHz, DMSO-d$_6$)** δ 198.0, 170.9, 164.1, 155.6, 153.7, 123.2, 77.6, 55.9, 54.7, 53.1, 48.0, 47.4, 43.4, 38.5, 38.2, 38.0, 37.8, 35.1, 35.1, 33.6, 31.9, 31.6, 28.2, 28.0, 23.7, 22.6, 20.5, 16.9, 16.8, 13.1; **HRMS** (APCI+) $[M+H]^+$ calc. for $C_{36}H_{59}N_4O_7$, 659.4378, observed, 659.4379.

Preparation of di-*tert*-butyl (((((((*E*)-1-((8*S*,9*S*,10*R*,13*S*,14*S*,17*S*)-10,13-dimethyl-3-oxo-2,3,6,7,8,9,10,11,12,13,14,15,16,17-tetradecahydro-1*H*-cyclopenta[a]phenanthren-17-yl)ethylidene)amino)oxy)carbonyl)azanediyl)bis(ethane-2,1-diyl))bis(methylcarbamate) (18x)

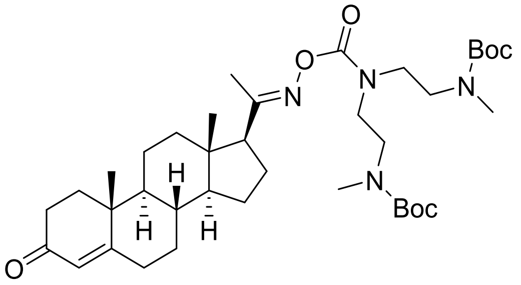

Prepared according to General Procedure E using amine nucleophile 1**7x**. Rf = 0.32 (30% ethyl acetate/DCM). The product was in the form of a white solid (202 mg, 0.294 mmol, 73% yield). **^{1}H NMR (600 MHz, DMSO-d$_6$)** δ 5.63 (s, 1H), 3.39 – 3.23 (m, 6H), 2.78 (s, 6H), 2.45 – 2.33 (m, 3H), 2.28 – 2.21 (m, 1H), 2.19 – 2.12 (m, 2H), 2.00 – 1.94 (m, 1H), 1.93 – 1.87 (m, 3H), 1.87 – 1.77 (m, 2H), 1.70 – 1.50 (m, 5H), 1.36 (s, 18H), 1.33 –1.17 (m, 6H), 1.15 (s, 3H), 1.03 – 0.90 (m, 2H), 0.64 (s, 3H); **^{13}C NMR (151 MHz, DMSO-d$_6$)** δ 198.0, 170.9, 163.5, 154.7, 153.3, 123.2, 78.4, 55.9, 54.7, 53.1, 46.8, 46.0, 43.4, 38.2, 37.8, 35.1, 35.1, 33.8, 33.6, 31.9, 31.6, 27.9, 23.7, 22.5, 20.5, 16.9, 16.8, 13.1; **HRMS** (APCI+) $[M+H]^+$ calc. for $C_{38}H_{63}N_4O_7$, 687.4691, observed, 687.4691.

Preparation of *tert*-butyl (2-((((((E)-1-((3R,8R,9S,10S,13S,14S,17S)-3-((tert-butyldimethylsilyl)oxy)-10,13-dimethylhexadecahydro-1H-cyclopenta[a]phenanthren-17-yl)ethylidene)amino)oxy)carbonyl)(methyl)amino)ethyl)(methyl)carbamate (19a)

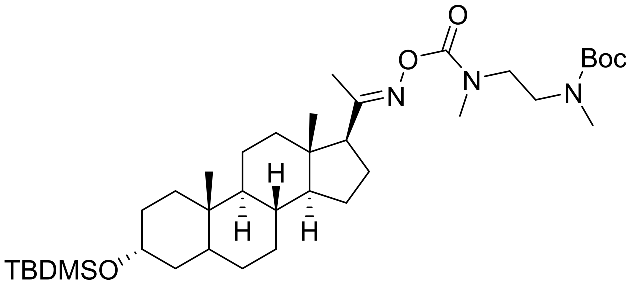

Prepared according to General Procedure E using amine nucleophile **17a**. The product was in the form of a white solid (285 mg, 0.431 mmol, 88%). **^{1}H NMR (600 MHz, CDCl$_3$)** δ 3.95 (t, *J* = 2.7 Hz, 1H), 3.41 (s, 4H), 2.98 (s, 3H), 2.89 (s, 3H), 2.36 – 2.31 (m, 1H), 2.27 (q, *J* = 10.1 Hz, 1H), 1.92 (s, 3H), 1.85 (dt, *J* = 11.7, 3.3 Hz, 1H), 1.75 – 1.64 (m, 4H), 1.62 – 1.56 (m, 2H), 1.55 – 1.48 (m, 2H), 1.45 (s, 9H), 1.41 – 1.38 (m, 1H), 1.37 – 1.32 (m, 3H), 1.31 – 1.25 (m, 1H), 1.25 – 1.20 (m, 2H), 1.19 – 1.13 (m, 3H), 1.00 – 0.90 (m, 1H), 0.89 (s, 9H), 0.79 – 0.76 (m, 1H), 0.75 (s, 3H), 0.66 (s, 3H), 0.01 (d, *J* = 1.3 Hz, 6H); **^{13}C NMR (151 MHz, CDCl$_3$)** δ ^{13}C NMR (151 MHz, CDCl$_3$) δ 165.2, 155.7, 79.7, 67.0, 57.2, 56.2, 54.4, 44.5, 39.2, 39.1, 36.9, 36.2, 35.8, 34.9, 32.6, 32.2, 29.9, 28.7, 28.5, 26.0, 24.3, 23.2, 20.3, 18.3, 17.1, 13.8, 11.6, -4.7, -4.7; **HRMS** (APCI+) [M+H]+ calc. for $C_{37}H_{68}O_5N_3Si_{28}$, 662.4934, observed, 662.4923.

Preparation of *tert*-butyl (2-((((((E)-1-((3R,8R,9S,10S,13S,14S,17S)-3-((tert-butyldimethylsilyl)oxy)-10,13-dimethylhexadecahydro-1H-cyclopenta[a]phenanthren-17-yl)ethylidene)amino)oxy)carbonyl)(cyclopropyl)amino)ethyl)(methyl)carbamate (19b)

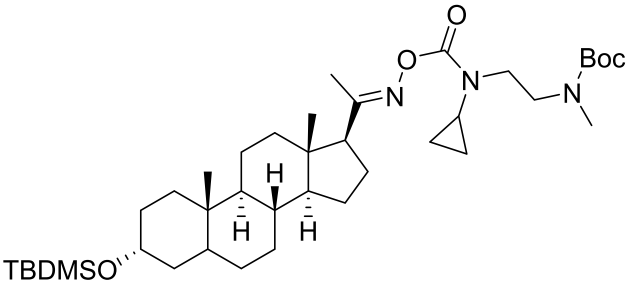

Prepared according to General Procedure E using amine nucleophile **17b**. The product was in the form of a white solid (290 mg, 0.421 mmol, 86%). **^{1}H NMR (400 MHz, CDCl$_3$)** δ 3.95 (t, *J* = 2.8 Hz, 1H), 3.41 (d, *J* = 6.4 Hz, 4H), 2.88 (d, *J* = 5.1 Hz, 3H), 2.37 – 2.22 (m, 2H), 1.94 (s, 3H), 1.89 – 1.81 (m, 1H), 1.75 – 1.48 (m, 8H), 1.47 – 1.41 (m, 9H), 1.39 – 1.30 (m, 4H), 1.28 – 1.11 (m, 7H), 1.00 – 0.92 (m, 1H), 0.88 (s, 9H), 0.81 – 0.73 (m, 6H), 0.67 (d, *J* = 13.8 Hz, 5H), 0.01 (s, 6H); **^{13}C NMR (101 MHz, CDCl$_3$)** δ 165.1, 155.9, 155.8, 79.8, 79.4, 67.0, 57.2, 56.2, 54.4, 47.5, 46.9, 46.4, 45.5, 44.5, 39.1, 39.1, 36.9, 36.1, 35.8, 34.8, 34.7, 32.5, 32.2, 31.7, 29.9, 28.7, 28.5, 26.0, 24.3, 23.2, 22.8, 20.9, 18.2, 17.3, 14.2, 13.8, 11.5, 8.3, -4.7, -4.7; **HRMS** (APCI+) [M+Na]+ calc. for $C_{39}H_{69}O_5N_3Na_{23}Si_{28}$, 710.4899, observed, 710.4901.

Preparation of *tert*-butyl (2-((((((*E*)-1-((3*R*,8*R*,9*S*,10*S*,13*S*,14*S*,17*S*)-3-((tert-butyldimethylsilyl)oxy)-10,13-dimethylhexadecahydro-1H-cyclopenta[a]phenanthren-17-yl)ethylidene)amino)oxy)carbonyl)(isopropyl)amino)ethyl)(methyl)carbamate (19c)

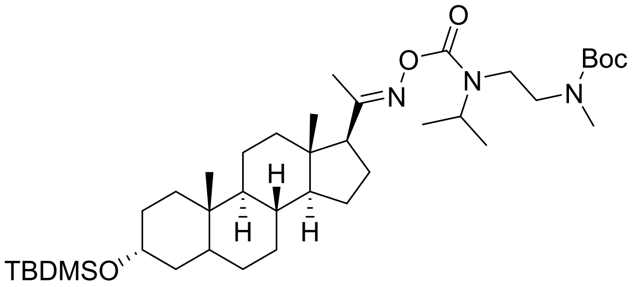

Prepared according to General Procedure E using amine nucleophile **17c**. The product was in the form of a white solid (287 mg, 0.416 mmol, 85%). **^{1}H NMR (600 MHz, CDCl$_3$)** δ 3.95 (t, *J* = 2.8 Hz, 1H), 3.38 (s, 2H), 3.33 – 3.20 (m, 2H), 2.89 (d, *J* = 17.7 Hz, 3H), 2.36 – 2.32 (m, 1H), 2.31 – 2.24 (m, 1H), 1.93 (s, 3H), 1.86 (dd, *J* = 11.8, 3.1 Hz, 1H), 1.76 – 1.49 (m, 8H), 1.46 (d, *J* = 4.3 Hz, 9H), 1.41 – 1.12 (m, 17H), 0.95 (dtd, *J* = 19.4, 11.8, 5.5 Hz, 1H), 0.88 (s, 9H), 0.80 – 0.74 (m, 4H), 0.67 (s, 3H), 0.01 (d, *J* = 1.2 Hz, 6H); **^{13}C NMR (151 MHz, CDCl$_3$)** δ 165.2, 155.7, 154.8, 79.7, 67.0, 57.3, 56.2, 54.5, 48.9, 47.8, 44.5, 40.6, 39.1, 36.9, 36.1, 35.9, 35.0, 32.6, 32.2, 29.9, 28.7, 28.6, 26.0, 24.3, 23.2, 21.1, 20.9, 20.6, 18.3, 17.4, 13.8, 11.5, -4.7, -4.7; **HRMS** (APCI+) [M+H]+ calc. for $C_{39}H_{72}O_5N_3Si_{28}$, 690.5247, observed, 690.5238.

Preparation of *tert*-butyl (2-((((((*E*)-1-((3*R*,8*R*,9*S*,10*S*,13*S*,14*S*,17*S*)-3-((tert-butyldimethylsilyl)oxy)-10,13-dimethylhexadecahydro-1*H*-cyclopenta[a]phenanthren-17-yl)ethylidene)amino)oxy)carbonyl)(cyclobutyl)amino)ethyl)(methyl)carbamate (19d)

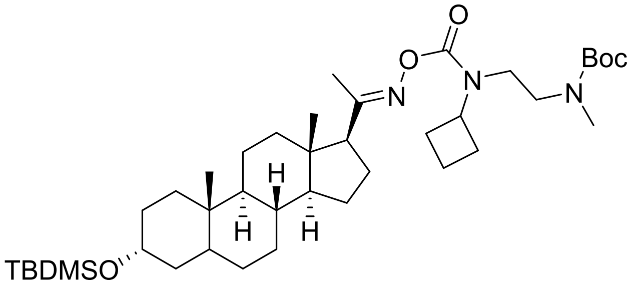

Prepared according to General Procedure E using amine nucleophile **17d**. The product was in the form of a white solid (289 mg, 0.411 mmol, 84%). **^{1}H NMR (600 MHz, CDCl$_3$)** δ 3.95 (t, *J* = 2.7 Hz, 1H), 3.40 (d, *J* = 7.0 Hz, 2H), 3.34 (dd, *J* = 8.4, 5.8 Hz, 2H), 2.89 (s, 3H), 2.34 (dd, *J* = 9.9, 8.4 Hz, 1H), 2.27 (dtd, *J* = 13.1, 10.6, 2.8 Hz, 1H), 2.21 – 2.10 (m, 4H), 1.94 (s, 3H), 1.85 (dt, *J* = 11.7, 3.3 Hz, 1H), 1.80 – 1.64 (m, 5H), 1.63 – 1.49 (m, 5H), 1.46 (s, 9H), 1.42 – 1.28 (m, 5H), 1.25 – 1.12 (m, 6H), 1.00 – 0.91 (m, 1H), 0.89 (s, 9H), 0.80 – 0.76 (m, 1H), 0.75 (s, 3H), 0.66 (s, 3H), 0.01 (d, *J* = 1.3 Hz, 6H); **^{13}C NMR (151 MHz, CDCl$_3$)** δ 165.2, 155.8, 154.7, 79.7, 67.0, 57.3, 56.2, 54.4, 51.4, 48.1, 44.5, 41.6, 39.2, 39.1, 36.9, 36.1, 35.9, 35.7, 32.6, 32.2, 32.2, 29.9, 29.3, 28.7, 28.6, 26.0, 24.3, 23.2, 20.9, 18.3, 17.3, 14.7, 13.8, 11.6, -4.7, -4.7.

Preparation of *tert*-butyl (2-((((((*E*)-1-((3*R*,8*R*,9*S*,10*S*,13*S*,14*S*,17*S*)-3-((tert-butyldimethylsilyl)oxy)-10,13-dimethylhexadecahydro-1*H*-cyclopenta[a]phenanthren-17-yl)ethylidene)amino)oxy)carbonyl)(2(dimethylamino)ethyl)amino)ethyl)(methyl)carbamate (19g)

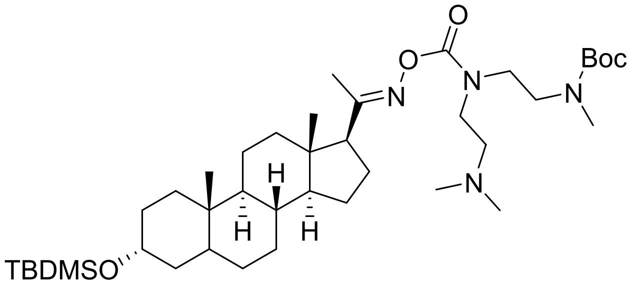

Prepared according to General Procedure E using amine nucleophile **17g**. The product was in the form of a white solid (303 mg, 0.421 mmol, 86%). **^{1}H NMR (600 MHz, $CDCl_3$)** δ 3.95 (t, *J* = 2.8 Hz, 1H), 3.42 (d, *J* = 24.4 Hz, 6H), 2.89 (s, 3H), 2.62 (d, *J* = 28.3 Hz, 1H), 2.47 (s, 1H), 2.39 – 2.30 (m, 3H), 2.26 (d, *J* = 8.6 Hz, 4H), 1.92 (s, 3H), 1.87 – 1.81 (m, 1H), 1.77 – 1.63 (m, 3H), 1.63 – 1.41 (m, 13H), 1.40 – 1.11 (m, 12H), 0.99 – 0.89 (m, 1H), 0.88 (s, 9H), 0.80 – 0.75 (m, 1H), 0.74 (s, 3H), 0.65 (s, 3H), 0.01 (d, *J* = 1.3 Hz, 6H); **^{13}C NMR (151 MHz, $CDCl_3$)** δ 165.3, 165.1, 155.7, 154.9, 154.7, 80.0, 79.7, 79.5, 67.0, 58.1, 57.9, 57.2, 56.2, 54.4, 47.8, 47.5, 47.1, 46.7, 46.6, 46.4, 45.9, 45.4, 44.5, 39.1, 39.1, 36.8, 36.1, 35.8, 34.8, 32.5, 32.1, 29.8, 28.7, 28.5, 26.0, 24.3, 23.1, 20.9, 18.2, 17.3, 13.8, 11.5, -4.7, -4.7; **HRMS** (APCI+) [M+H]+ calc. for $C_{40}H_{75}O_5N_4Si_{28}$, 719.5512, observed, 719.5498.

Preparation of *tert*-butyl (2-((((((*E*)-1-((3*R*,8*R*,9*S*,10*S*,13*S*,14*S*,17*S*)-3-((tert-butyldimethylsilyl)oxy)-10,13-dimethylhexadecahydro-1*H*-cyclopenta[a]phenanthren-17-yl)ethylidene)amino)oxy)carbonyl)(3-morpholinopropyl)amino)ethyl)(methyl)carbamate (19i)

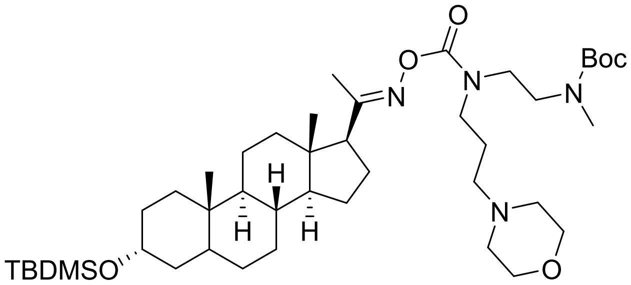

Prepared according to General Procedure E using amine nucleophile **17i**. The product was in the form of a white solid (311 mg, 0.401 mmol, 82%). **^{1}H NMR (600 MHz, CDCl$_3$)** δ 3.95 (t, *J* = 2.8 Hz, 1H), 3.68 (t, *J* = 4.7 Hz, 4H), 3.49 – 3.25 (m, 6H), 2.89 (s, 3H), 2.48 – 2.37 (m, 4H), 2.33 (t, *J* = 9.0 Hz, 3H), 2.29 – 2.16 (m, 1H), 1.91 (s, 3H), 1.87 – 1.81 (m, 1H), 1.81 – 1.62 (m, 4H), 1.62 – 1.46 (m, 4H), 1.44 (s, 9H), 1.40 – 1.11 (m, 12H), 0.99 – 0.90 (m, 1H), 0.88 (s, 9H), 0.80 – 0.75 (m, 1H), 0.74 (s, 3H), 0.65 (s, 3H), 0.01 (d, *J* = 1.3 Hz, 6H); **^{13}C NMR (151 MHz, CDCl$_3$)** δ 165.2, 165.0, 156.0, 155.7, 155.1, 154.8, 80.0, 79.7, 79.5, 67.1, 67.1, 67.0, 57.2, 56.2, 56.1, 54.4, 53.8, 47.9, 47.1, 46.4, 45.8, 45.5, 45.3, 44.5, 39.1, 39.1, 36.7, 36.1, 35.8, 34.8, 34.8, 32.5, 32.1, 29.8, 28.7, 28.5, 24.3, 23.1, 20.9, 18.2, 17.2, 13.8, 11.5, -4.7, -4.7; **HRMS** (APCI+) [M+H]+ calc. for $C_{43}H_{79}O_6N_4Si_{28}$, 775.5774, observed, 775.5753.

Preparation of *tert*-butyl (2-((2-(1*H*-imidazol-1-yl)ethyl)(((((*E*)-1-((3*R*,8*R*,9*S*,10*S*,13*S*,14*S*,17*S*)-3-((tert-butyldimethylsilyl)oxy)-10,13-dimethylhexadecahydro-1*H*-cyclopenta[a]phenanthren-17 yl)ethylidene)amino)oxy)carbonyl)amino)ethyl)(methyl)carbamate (19k)

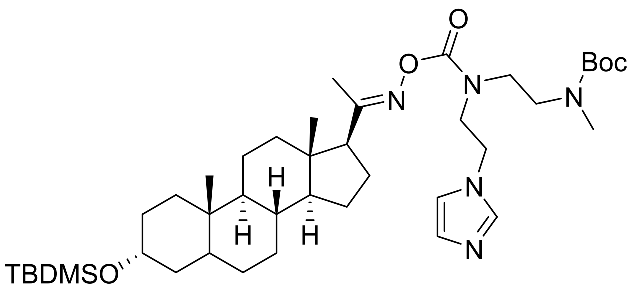

Prepared according to General Procedure E using amine nucleophile **17k**. The product was in the form of a white solid (291 mg, 0.392 mmol, 80%). **^{1}H NMR (600 MHz, CDCl$_3$)** δ 7.46 (s, 1H), 7.06 (d, *J* = 13.9 Hz, 1H), 6.98 – 6.85 (m, 1H), 4.20 (d, *J* = 6.4 Hz, 1H), 3.95 (t, *J* = 2.8 Hz, 1H), 3.64 – 3.45 (m, 2H), 3.38 – 3.16 (m, 2H), 3.09 – 2.89 (m, 1H), 2.86 – 2.71 (m, 3H), 2.34 (t, *J* = 9.2 Hz, 1H), 2.30 – 2.19 (m, 1H), 1.97 – 1.89 (m, 3H), 1.87 – 1.80 (m, 2H), 1.78 – 1.63 (m, 3H), 1.62 – 1.45 (m, 4H), 1.41 (s, 9H), 1.39 – 1.27 (m, 5H), 1.27 – 1.08 (m, 6H), 1.00 – 0.90 (m, 1H), 0.88 (s, 9H), 0.74 (s, 4H), 0.66 (s, 3H), 0.00 (d, *J* = 1.3 Hz, 6H); **^{13}C NMR (151 MHz, CDCl$_3$)** δ 165.9, 156.1, 155.4 154.5, 137.7, 137.3, 130.3, 130.1, 119.1, 118.9, 80.2, 80.0, 79.7, 66.9, 57.2, 56.2, 54.4, 53.5, 50.9, 49.8, 48.5, 47.7, 47.0, 46.7, 46.0, 45.8, 45.5, 45.1, 44.5, 39.1, 39.1, 36.8, 36.1, 35.8, 35.5, 35.0, 34.8, 32.5, 32.1, 29.84, 28.7, 28.5, 26.0, 23.1, 20.9, 18.2, 17.3, 13.8, 11.5, -4.7, -4.7; **HRMS** (APCI+) [M+H]+ calc. for $C_{41}H_{72}O_5N_5Si_{28}$, 742.5308, observed, 742.5298.

7.4 Preparation of Target Progesterone and Allopregnanolone C20-Oxime Prodrugs

Preparation of (*E*)-9-((8*S*,9*S*,10*R*,13*S*,14*S*,17*S*)-10,13-dimethyl-3-oxo-2,3,6,7,8,9,10,11,12,13,14,15,16,17-tetradecahydro-1*H*-cyclopenta[a]phenanthren-17-yl)-5-methyl-7-oxa-2,5,8-triazadec-8-en-6-one (20a)

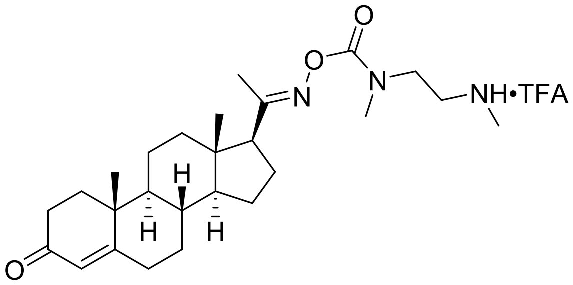

Prepared according to General Procedure F to afford the TFA salt. The product was in the form of a white solid (310 mg, 0.573 mmol, 91% yield). **^{1}H NMR (600 MHz, DMSO-d$_6$)** δ 8.58 (br s, 2H), 5.64 (s, 1H), 3.52 (s, 2H), 3.09 (t, *J* = 6.1 Hz, 2H), 2.91 (s, 3H), 2.59 (s, 3H), 2.45 – 2.34 (m, 3H), 2.28 – 2.21 (m, 1H), 2.21 – 2.11 (m, 2H), 2.00 – 1.95 (m, 1H), 1.93 (s, 3H), 1.90 – 1.83 (m, 1H), 1.83 – 1.77 (m, 1H), 1.70 – 1.49 (m, 5H), 1.42 – 1.29 (m, 2H), 1.25 – 1.13 (m, 3H), 1.15 (s, 3H), 1.03 – 0.89 (m, 2H), 0.65 (s, 3H); **^{13}C NMR (151 MHz, DMSO-d$_6$)** δ 198.0, 170.9, 164.7, 158.1 (J^2_{CF} = 31.7 Hz), 154.4, 123.2, 117.4 (J^1_{CF} = 302.0 Hz), 55.9, 54.7, 53.1, 46.2, 45.1, 43.4, 38.2, 37.8, 35.1, 35.1, 33.9, 33.6, 32.8, 32.0, 31.6, 23.7, 22.6, 20.6, 16.9, 16.8, 13.1; **HRMS** (APCI+) $[M+H]^+$ calc. for $C_{26}H_{42}O_3N_3$, 444.3221, observed, 444.3222

Preparation of (*E*)-5-cyclopropyl-9-((8*S*,9*S*,10*R*,13*S*,14*S*,17*S*)-10,13-dimethyl-3-oxo-2,3,6,7,8,9,10,11,12,13,14,15,16,17-tetradecahydro-1*H*-cyclopenta[a]phenanthren-17-yl)-7-oxa-2,5,8-triazadec-8-en-6-one 2,2,2-trifluoroacetate (20b)

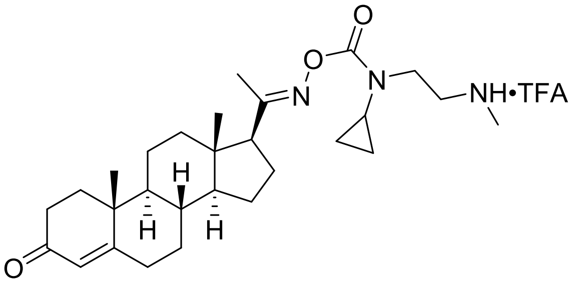

Prepared according to General Procedure F to afford the TFA salt. The product was in the form of a white solid (290 mg, 0.514 mmol, 94% yield). **^{1}H NMR (600 MHz, DMSO-d$_6$)** δ 8.56 (br s, 2H), 5.64 (s, 1H), 3.51 (t, *J* = 6.4 Hz, 2H), 3.11 – 3.06 (m, 2H), 2.70 (sept, *J* = 6.8 Hz, 1H), 2.59 (s, 3H), 2.45 – 2.34 (m, 3H), 2.28 – 2.22 (m, 1H), 2.20 – 2.12 (m, 2H), 2.01 – 1.97 (m, 1H), 1.96 (s, 3H), 1.91 – 1.85 (m, 1H), 1.84 – 1.77 (m, 1H), 1.71 – 1.50 (m, 5H), 1.43 – 1.29 (m, 2H), 1.26 – 1.16 (m, 2H), 1.15 (s, 3H), 1.04 – 0.90 (m, 2H), 0.79 – 0.74 (m, 2H), 0.73 – 0.69 (m, 2H), 0.65

(s, 3H); **^{13}C NMR (151 MHz, DMSO-d$_6$)** δ 198.0, 170.8, 164.7, 158.1 (J^2_{CF} = 31.7 Hz), 155.2, 123.2, 117.4 (J^1_{CF} = 302.0 Hz), 55.9, 54.7, 53.1, 46.9, 43.9, 43.4, 38.2, 37.8, 35.1, 35.1, 33.6, 32.9, 31.9, 31.6, 28.8, 23.7, 22.6, 20.6, 17.0, 16.9, 13.1, 7.7, 7.7; **HRMS** (APCI+) $[M+H]^+$ calc. for $C_{28}H_{44}N_3O_3$, 470.3377, observed, 470.3384.

Preparation of (*E*)-9-((8*S*,9*S*,10*R*,13*S*,14*S*,17*S*)-10,13-dimethyl-3-oxo-2,3,6,7,8,9,10,11,12,13,14,15,16,17-tetradecahydro-1*H*-cyclopenta[a]phenanthren-17-yl)-5-isopropyl-7-oxa-2,5,8-triazadec-8-en-6-one 2,2,2-trifluoroacetate (20c)

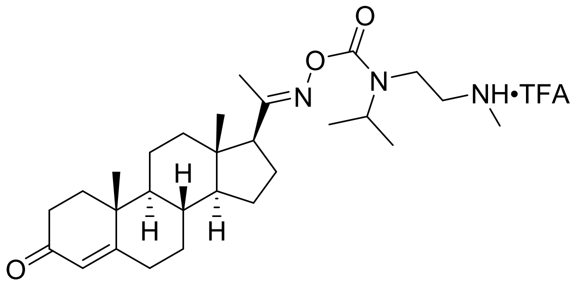

Prepared according to General Procedure F to afford the TFA salt. The product was in the form of a white solid (193 mg, 0.329 mmol, 94% yield). **^{1}H NMR (600 MHz, DMSO-d$_6$)** δ 8.52 (br s, 2H), 5.63 (s, 1H), 4.12 (sept, *J* = 6.8 Hz, 1H), 3.42 (t, *J* = 7.0 Hz, 2H), 3.06 – 3.01 (m, 2H), 2.60 (s, 3H), 2.45 – 2.35 (m, 3H), 2.28 – 2.22 (m, 1H), 2.19 – 2.11 (m, 2H), 2.01 – 1.94 (m, 1H), 1.93 (s, 3H), 1.90 – 1.85 (m, 1H), 1.83 – 1.77 (m, 1H), 1.70 – 1.50 (m, 5H), 1.42 – 1.28 (m, 2H), 1.25 – 1.17 (m, 2H), 1.15 (s, 3H), 1.15 (s, 6H), 1.03 – 0.90 (m, 2H), 0.65 (s, 3H); **^{13}C NMR (151 MHz, DMSO-d$_6$)** δ 198.0, 170.8, 164.8, 158.1 (J^2_{CF} = 31.7 Hz), 154.3, 123.2, 117.4 (J^1_{CF} = 302.0 Hz), 55.9, 54.7, 53.1, 48.2, 47.9, 43.4, 38.2, 37.7, 35.1, 35.1, 33.6, 33.0, 31.9, 31.6, 23.7, 22.6, 20.6, 20.3, 17.0, 16.9, 13.1. One ^{13}C signal not observed; **HRMS** (APCI+) $[M+H]^+$ calc. for $C_{28}H_{46}N_3O_3$, 472.3534, observed, 472.3541.

Preparation of (*E*)-5-cyclobutyl-9-((8*S*,9*S*,10*R*,13*S*,14*S*,17*S*)-10,13-dimethyl-3-oxo-2,3,6,7,8,9,10,11,12,13,14,15,16,17-tetradecahydro-1*H*-cyclopenta[a]phenanthren-17-yl)-7-oxa-2,5,8-triazadec-8-en-6-one 2,2,2-trifluoroacetate (20d)

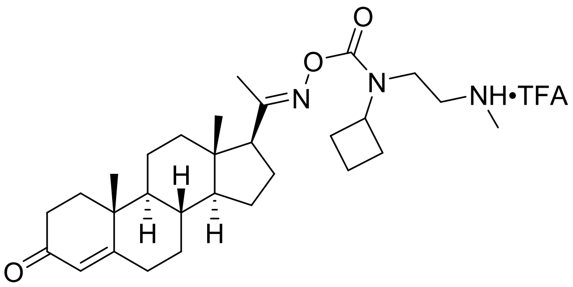

Prepared according to General Procedure F to afford the TFA salt. The product was in the form of a white solid (450 mg, 0.753 mmol, 96% yield). **^{1}H NMR (600 MHz, DMSO-d$_6$)** δ 8.59 (br s,

2H), 5.63 (s, 1H), 4.30 – 4.24 (m, 1H), 3.54 (t, J = 6.8 Hz, 2H), 3.01 – 2.98 (m, 2H), 2.60 (s, 3H), 2.45 – 2.34 (m, 3H), 2.28– 2.21 (m, 1H), 2.19 – 2.07 (m, 6H), 2.01 – 1.95 (m, 1H), 1.94 (s, 3H), 1.90 – 1.84 (m, 1H), 1.84 – 1.77 (m, 1H), 1.70 – 1.50 (m, 7H), 1.42 – 1.28 (m, 2H), 1.26 – 1.17 (m, 2H), 1.15 (s, 3H), 1.03 – 0.90 (m, 2H), 0.65 (s, 3H); **^{13}C NMR (151 MHz, DMSO-d_6)** δ 198.0, 170.8, 164.9, 158.1 (J^2_{CF} = 31.7 Hz), 154.1, 123.2, 117.4 (J^1_{CF} = 302.0 Hz), 55.9, 54.7, 53.1, 50.6, 47.8, 43.4, 38.2, 37.7, 35.1, 35.1, 33.6, 33.0, 31.9, 31.6, 28.5, 23.7, 22.5, 20.6, 17.0, 16.9, 14.1, 13.1. One ^{13}C signal not observed. **HRMS** (APCI+) $[M+H]^+$ calc. for $C_{29}H_{46}N_3O_3$, 484.3534, observed, 484.3541.

Preparation of (*E*)-5-cyclopentyl-9-((8*S*,9*S*,10*R*,13*S*,14*S*,17*S*)-10,13-dimethyl-3-oxo-2,3,6,7,8,9,10,11,12,13,14,15,16,17-tetradecahydro-1*H*-cyclopenta[a]phenanthren-17-yl)-7-oxa-2,5,8-triazadec-8-en-6-one 2,2,2-trifluoroacetate (20e)

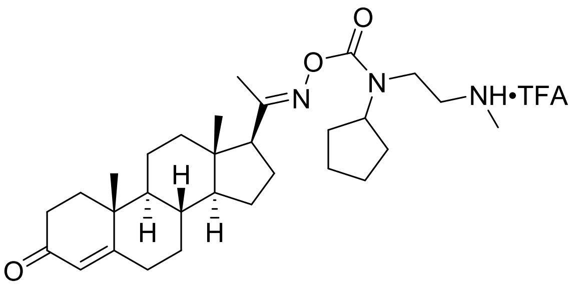

Prepared according to General Procedure F to afford the TFA salt. The product was in the form of a white solid (298 mg, 0.487 mmol, 95% yield). **^{1}H NMR (600 MHz, DMSO-d_6)** δ 8.62 (br s, 2H), 5.63 (s, 1H), 4.16 (p, J = 8.5 Hz, 1H), 3.42 (t, J = 7.1 Hz, 2H), 3.06 – 3.00 (m, 2H), 2.60 (s, 3H), 2.45 – 2.33 (m, 3H), 2.28 – 2.22 (m, 1H), 2.21 – 2.11 (m, 2H), 2.00 – 1.94 (m, 1H), 1.93 (s, 3H), 1.90 – 1.85 (m, 1H), 1.85 – 1.77 (m, 3H), 1.71 – 1.47 (m, 11H), 1.42 – 1.28 (m, 2H), 1.26 – 1.16 (m, 2H), 1.15 (s, 3H), 1.03 – 0.90 (m, 2H), 0.65 (s, 3H); **^{13}C NMR (151 MHz, DMSO-d_6)** δ 198.0, 170.8, 164.9, 158.1 (J^2_{CF} = 31.7 Hz), 154.3, 123.2, 117.4 (J^1_{CF} = 302.0 Hz), 57.8, 55.9, 54.7, 53.1, 47.8, 43.4, 38.2, 37.7, 35.1, 35.1, 33.6, 33.0, 31.9, 31.6, 28.9, 23.7, 23.1, 22.6, 20.6, 17.0, 16.9, 13.1. One ^{13}C signal not observed; **HRMS** (APCI+) $[M+H]^+$ calc. for $C_{30}H_{48}N_3O_3$, 498.3690, observed, 498.3695.

Preparation of (*E*)-9-((8*S*,9*S*,10*R*,13*S*,14*S*,17*S*)-10,13-dimethyl-3-oxo-2,3,6,7,8,9,10,11,12,13,14,15,16,17-tetradecahydro-1*H*-cyclopenta[a]phenanthren-17-yl)-5-isobutyl-7-oxa-2,5,8-triazadec-8-en-6-one 2,2,2-trifluoroacetate (20f)

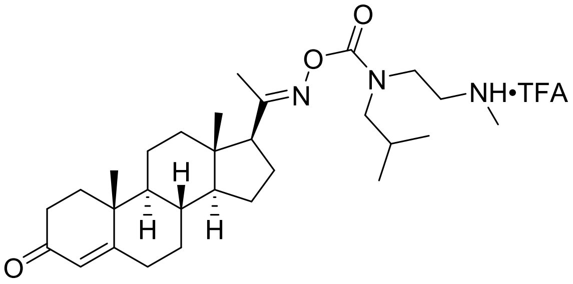

Prepared according to General Procedure F to afford the TFA salt. The product was in the form of a white solid (285 mg, 0.475 mmol, 93% yield). **^{1}H NMR (600 MHz, DMSO-d$_6$)** δ 8.68 (br s, 1H), 8.52 (br s, 1H), 5.64 (s, 1H), 3.55 – 3.45 (m, 2H), 3.12 – 3.02 (m, 4H), 2.59 (s, 3H), 2.45 – 2.33 (m, 3H), 2.28 – 2.22 (m, 1H), 2.21 – 2.11 (m, 2H), 2.00 – 1.84 (m, 6H), 1.84 – 1.77 (m, 1H), 1.70 – 1.50 (m, 5H), 1.43 – 1.28 (m, 2H), 1.26 – 1.16 (m, 2H), 1.15 (s, 3H), 1.03 – 0.90 (m, 2H), 0.85 (d, *J* = 6.7 Hz, 6H), 0.64 (s, 3H); **^{13}C NMR (151 MHz, DMSO-d$_6$)** δ 198.0, 170.8, 164.3, 158.1 (J^2_{CF} = 31.7 Hz), 154.5, 123.2, 117.4 (J^1_{CF} = 302.0 Hz), 55.9, 54.7, 54.1, 53.1, 46.5, 43.9, 43.4, 38.2, 37.7, 35.1, 35.1, 33.6, 32.9, 31.9, 31.6, 27.0, 23.7, 22.5, 20.5, 19.8, 19.8, 16.9 (2C), 13.1. **HRMS** (APCI+) $[M+H]^+$ calc. for $C_{29}H_{48}N_3O_3$, 486.3690, observed, 486.3698.

Preparation of (*E*)-9-((8*S*,9*S*,10*R*,13*S*,14*S*,17*S*)-10,13-dimethyl-3-oxo-2,3,6,7,8,9,10,11,12,13,14,15,16,17-tetradecahydro-1*H*-cyclopenta[a]phenanthren-17-yl)-2-methyl-5-(2-(methylamino)ethyl)-7-oxa-2,5,8-triazadec-8-en-6-one 2,2,2-trifluoroacetate (20g)

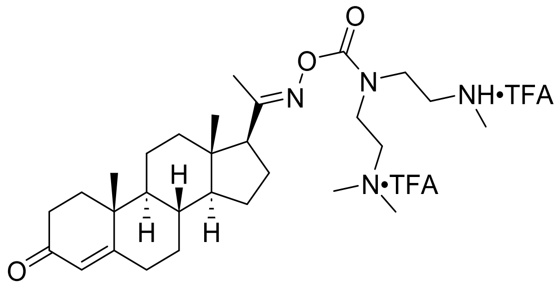

Prepared according to General Procedure F to afford the TFA salt. The product was in the form of a white solid (325 mg, 0.529 mmol, 89% yield). **^{1}H NMR (600 MHz, DMSO-d$_6$)** δ 9.10 – 8.50 (m, 2H), 5.64 (s, 1H), 3.64 – 3.52 (m, 4H), 3.25 – 3.16 (m, 2H), 3.12 (t, *J* = 6.3 Hz, 2H), 2.86 – 2.70 (m, 6H), 2.60 (s, 3H), 2.45 – 2.36 (m, 3H), 2.28 – 2.22 (m, 1H), 2.21 – 2.11 (m, 2H), 2.02 – 1.97 (m, 1H), 1.96 (s, 3H), 1.89 – 1.84 (m, 1H), 1.84 – 1.77 (m, 1H), 1.71 – 1.50 (m, 6H), 1.43 – 1.30 (m, 2H), 1.26 – 1.17 (m, 1H), 1.15 (s, 3H), 1.03 – 0.90 (m, 2H), 0.65 (s, 3H); **^{13}C NMR (151 MHz, DMSO-d$_6$)** δ 198.0, 170.8, 165.3, 158.4 (J^2_{CF} = 31.7 Hz), 154.0, 123.2, 117.1 (J^1_{CF} = 302.0

Hz), 55.9, 54.7, 54.3, 53.1, 46.4, 43.5, 42.8, 42.3, 38.2, 37.7, 35.1, 35.1, 33.6, 32.8, 31.9, 31.6, 23.7, 22.5, 20.5, 17.0, 16.9, 13.1. One ^{13}C signal not observed; **HRMS** (APCI+) $[M+H]^+$ calc. for $C_{29}H_{49}N_4O_3$, 501.3799, observed, 501.3805.

Preparation of (*E*)-9-((8*S*,9*S*,10*R*,13*S*,14*S*,17*S*)-10,13-dimethyl-3-oxo-2,3,6,7,8,9,10,11,12,13,14,15,16,17-tetradecahydro-1*H*-cyclopenta[a]phenanthren-17-yl)-5-(2-(pyrrolidin-1-yl)ethyl)-7-oxa-2,5,8-triazadec-8-en-6-one 2,2,2-trifluoroacetate (20h)

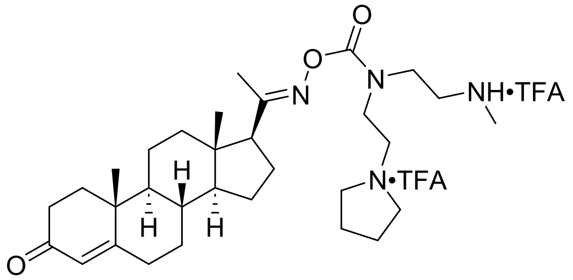

Prepared according to General Procedure F to afford the TFA salt. The product was in the form of a white solid (440 mg, 0.689 mmol, 90% yield). **^{1}H NMR (600 MHz, DMSO-d$_6$)** δ 8.94 (br s, 1H), 8.67 (br s, 1H), 5.64 (s, 1H), 3.64 – 3.50 (m, 6H), 3.43 – 3.30 (m, 6H), 3.15 – 3.10 (m, 2H), 3.10 – 3.00 (m, 1H), 2.60 (s, 3H), 2.45 – 2.35 (m, 3H), 2.28 – 2.22 (m, 1H), 2.19 – 2.11 (m, 2H), 2.06 – 1.97 (m, 2H), 1.96 (s, 3H), 1.92 – 1.83 (m, 3H), 1.83 – 1.77 (m, 1H), 1.70 – 1.50 (m, 5H), 1.42 – 1.30 (m, 2H), 1.26 – 1.19 (m, 1H), 1.18 – 1.12 (m, 1H), 1.15 (s, 3H), 1.03 – 0.89 (m, 2H), 0.65 (s, 3H); **^{13}C NMR (151 MHz, DMSO-d$_6$)** δ 198.0, 170.8, 165.4, 158.5 (J^2_{CF} = 31.7 Hz), 153.9, 123.2, 117.2 (J^1_{CF} = 302.0 Hz), 55.9, 54.7, 53.5, 53.1, 51.3, 46.3, 43.5, 42.8, 38.2, 37.7, 35.1, 35.1, 33.6, 32.8, 31.9, 31.6, 23.7, 22.7, 22.5, 20.5, 17.0, 16.9, 13.1; One ^{13}C signal not observed; **HRMS** (APCI+) $[M+H]^+$ calc. for $C_{31}H_{51}N_4O_3$, 527.3956, observed, 527.3961.

Preparation of (*E*)-9-((8*S*,9*S*,10*R*,13*S*,14*S*,17*S*)-10,13-dimethyl-3-oxo-2,3,6,7,8,9,10,11,12,13,14,15,16,17-tetradecahydro-1*H*-cyclopenta[a]phenanthren-17-yl)-5-(3-morpholinopropyl)-7-oxa-2,5,8-triazadec-8-en-6-one 2,2,2-trifluoroacetate (20i)

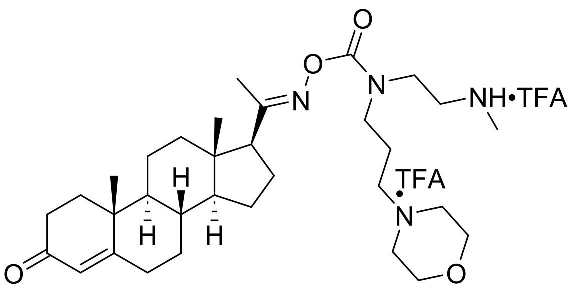

Prepared according to General Procedure F to afford the TFA salt. The product was in the form of a white solid (389 mg, 0.496 mmol, 65% yield). **^{1}H NMR (600 MHz, DMSO-d$_6$)** δ 10.6 (s, 1H), 10.3 (s, 1H), 8.85 (s, 1H), 8.60 (s, 1H), 5.64 (s, 1H), 4.04 – 3.83 (m, 2H), 3.71 – 3.57 (m, 2H), 3.55 – 3.48 (m, 2H), 3.45 – 3.26 (m, 5H), 3.17 – 2.97 (m, 5H), 2.60 (s, 3H), 2.45 – 2.35 (m, 3H),

2.28 – 2.22 (m, 1H), 2.20 – 2.11 (m, 2H), 2.02 – 1.85 (m, 7H), 1.84 – 1.77 (m, 1H), 1.71 – 1.49 (m, 5H), 1.42 – 1.30 (m, 2H), 1.27 – 1.16 (m, 2H), 1.15 (s, 3H), 1.03 – 0.90 (m, 2H), 0.65 (s, 3H); **^{13}C NMR (151 MHz, DMSO-d$_6$)** δ 198.0, 170.8, 164.6, 158.1 (J^2_{CF} = 31.7 Hz), 154.2, 123.2, 117.4 (J^1_{CF} = 302.0 Hz), 63.4, 55.9, 54.7, 53.7, 53.1, 51.2, 46.7, 44.3, 43.4, 42.8, 38.2, 37.7, 35.1, 35.1, 33.6, 32.9, 31.9, 31.6, 23.7, 22.5, 20.5, 17.0, 16.9, 13.1. One ^{13}C signal not observed; **HRMS** (APCI+) $[M+H]^+$ calc. for $C_{32}H_{53}N_4O_4$, 557.4061, observed, 557.4065.

Preparation of (*E*)-9-((8*S*,9*S*,10*R*,13*S*,14*S*,17*S*)-10,13-dimethyl-3-oxo-2,3,6,7,8,9,10,11,12,13,14,15,16,17-tetradecahydro-1*H*-cyclopenta[a]phenanthren-17-yl)-5-(2-morpholinoethyl)-7-oxa-2,5,8-triazadec-8-en-6-one 2,2,2-trifluoroacetate (20j)

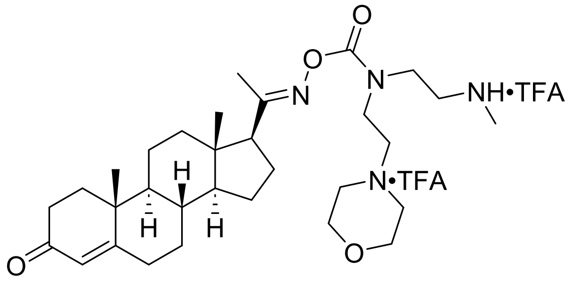

Prepared according to General Procedure F to afford the TFA salt. The product was in the form of a white solid (295 mg, 0.383 mmol, 81% yield). **^{1}H NMR (600 MHz, DMSO-d$_6$)** δ 8.86 (br s, 1H), 8.59 (br s, 1H), 5.64 (s, 1H), 4.04 – 3.90 (m, 1H), 3.68 – 3.38 (m, 11H), 3.18 – 3.00 (m, 4H), 2.60 (s, 3H), 2.45 – 2.36 (m, 3H), 2.28 – 2.22 (m, 1H), 2.20 – 2.10 (m, 2H), 2.00 – 1.98 (m, 1H), 1.95 (s, 3H), 1.89 – 1.84 (m, 1H), 1.84 – 1.77 (m, 1H), 1.71 – 1.60 (m, 3H), 1.59 – 1.50 (m, 2H), 1.43 – 1.29 (m, 2H), 1.27 – 1.16 (m, 2H), 1.15 (s, 3H), 1.03 – 0.90 (m, 2H), 0.65 (s, 3H); **^{13}C NMR (151 MHz, DMSO-d$_6$)** δ 198.0, 170.8, 165.4, 158.4 (J^2_{CF} = 31.7 Hz), 154.1, 123.2, 117.1 (J^1_{CF} = 302.0 Hz), 63.4, 55.9, 54.7, 53.1, 51.5, 46.7, 43.5, 43.2, 40.1, 38.2, 37.7, 35.1, 35.1, 33.61, 32.8, 31.9, 31.6, 23.7, 22.5, 20.5, 17.0, 16.9, 13.1. One ^{13}C signal not observed; **HRMS** (APCI+) $[M+H]^+$ calc. for $C_{31}H_{51}N_4O_4$, 543.3910, not observed.

Preparation of (*E*)-5-(2-(1*H*-imidazol-1-yl)ethyl)-9-((8*S*,9*S*,10*R*,13*S*,14*S*,17*S*)-10,13-dimethyl-3-oxo-2,3,6,7,8,9,10,11,12,13,14,15,16,17-tetradecahydro-1*H*-cyclopenta[a]phenanthren-17-yl)-7-oxa-2,5,8-triazadec-8-en-6-one 2,2,2-trifluoroacetate (20k)

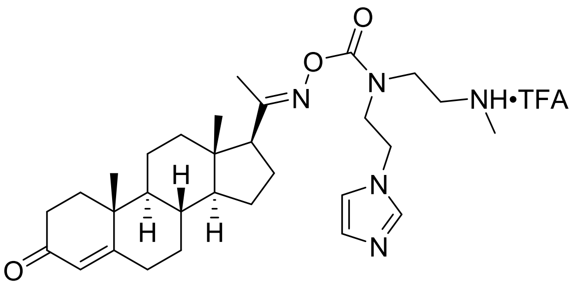

Prepared according to General Procedure F to afford the TFA salt. The product was in the form of a white solid (325 mg, 0.432 mmol, 77% yield). **^{1}H NMR (600 MHz, DMSO-d_6)** δ 8.93 (s, 1H), 8.82 (br s, 1H), 8.59 (br s, 1H), 7.69 (s, 1H), 7.55 (s, 1H), 5.64 (s, 1H), 4.35 (t, *J* = 5.8 Hz, 2H), 3.71 – 3.65 (m, 2H), 3.53 – 3.44 (m, 2H), 3.12 – 3.03 (m, 2H), 2.58 (s, 3H), 2.45 – 2.36 (m, 2H), 2.36 – 2.28 (m, 1H), 2.28 – 2.22 (m, 1H), 2.20 – 2.13 (m, 1H), 2.12 – 2.05 (m, 1H), 2.00 – 1.94 (m, 1H), 1.94 – 1.88 (m, 1H), 1.88 – 1.76 (m, 4H), 1.67 – 1.49 (m, 5H), 1.43 – 1.27 (m, 2H), 1.25 – 1.17 (m, 2H), 1.15 (s, 3H), 1.03 – 0.90 (m, 2H), 0.61 (s, 3H); **^{13}C NMR (151 MHz, DMSO-d_6)** δ 198.0, 170.8, 164.6, 158.1 (J^2_{CF} = 31.7 Hz), 154.1, 136.1, 123.2, 122.1, 121.4, , 117.4 (J^1_{CF} = 302.0 Hz), 55.9, 54.7, 53.1, 46.8, 46.4, 46.3, 43.4, 43.0, 38.2, 37.7, 35.1, 35.1, 33.6, 32.9, 31.9, 31.6, 23.7, 22.5, 20.5, 16.9, 13.1. One ^{13}C signal not observed; **HRMS** (APCI+) $[M+H]^+$ calc. for $C_{30}H_{46}N_5O_3$, 524.3595, observed, 524.3602.

Preparation of (8*S*,9*S*,10*R*,13*S*,14*S*,17*S*)-10,13-dimethyl-17-((*E*)-1-(((methyl(((*S*)-pyrrolidin-2-yl)methyl)carbamoyl)oxy)imino)ethyl)-1,2,6,7,8,9,10,11,12,13,14,15,16,17-tetradecahydro-3*H*-cyclopenta[a]phenanthren-3-one 2,2,2-trifluoroacetate (20l)

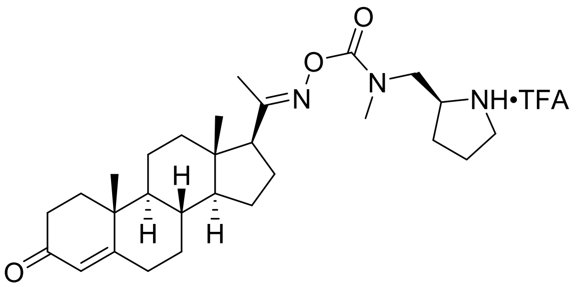

Prepared according to General Procedure F to afford the TFA salt. The product was in the form of a white solid (210 mg, 0.378 mmol, 82% yield). **^{1}H NMR (600 MHz, DMSO-d_6)** δ 9.20 (br s, 1H), 8.39 (br s, 1H), 5.64 (s, 1H), 3.72 – 3.63 (m, 1H), 3.48 (s, 3H), 3.30 – 3.23 (m, 1H), 3.18 – 3.12 (m, 1H), 2.95 (s, 3H), 2.45 – 2.35 (m, 3H), 2.28 – 2.22 (m, 1H), 2.21 – 2.11 (m, 2H), 2.08 – 2.00 (m, 1H), 1.99 – 1.95 (m, 1H), 1.93 (s, 3H), 1.91 – 1.84 (m, 2H), 1.83 – 1.77 (m, 1H), 1.70 – 1.50 (m, 5H), 1.42 – 1.28 (m, 2H), 1.26 – 1.17 (m, 2H), 1.15 (s, 3H), 1.03 – 0.90 (m, 2H), 0.65 (s, 3H); **^{13}C NMR (151 MHz, DMSO-d_6)** δ 198.0, 170.8, 164.8, 158.1 (J^2_{CF} = 31.7 Hz), 154.6, 123.2, 117.4 (J^1_{CF} = 302.0 Hz), 58.4, 55.9, 54.7, 53.1, 49.6, 44.8, 43.4, 38.2, 37.7, 35.1, 35.1, 34.7, 33.6, 32.0, 31.6, 27.3, 23.7, 22.5, 22.3, 20.6, 16.9, 16.8, 13.1; **HRMS** (APCI+) $[M+H]^+$ calc. for $C_{28}H_{44}N_3O_3$, 470.3382, observed, 470.3361.

Preparation of (8*S*,9*S*,10*R*,13*S*,14*S*,17*S*)-10,13-dimethyl-17-((*E*)-1-(((methyl(((*R*)-pyrrolidin-2-yl)methyl)carbamoyl)oxy)imino)ethyl)-1,2,6,7,8,9,10,11,12,13,14,15,16,17-tetradecahydro-3*H*-cyclopenta[a]phenanthren-3-one 2,2,2-trifluoroacetate (20m)

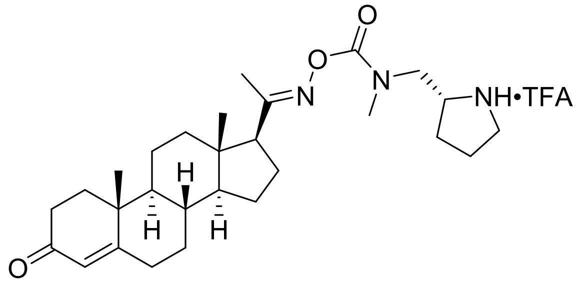

Prepared according to General Procedure F to afford the TFA salt. The product was in the form of a white solid (353 mg, 0.605 mmol, 91% yield). **^{1}H NMR (600 MHz, DMSO-d$_6$)** δ 9.25 (br s, 1H), 8.42 (br s, 1H), 5.63 (s, 1H), 3.72 – 3.63 (m, 1H), 3.60 – 3.33 (m, 3H), 3.29 – 3.25 (m, 1H), 3.18 – 3.08 (m, 1H), 2.95 (s, 3H), 2.44 – 2.35 (m, 3H), 2.28 – 2.22 (m, 1H), 2.19 – 2.12 (m, 2H), 2.08 – 2.01 (m, 1H), 2.00 – 1.84 (m, 4H), 1.92 (s, 3H), 1.83 – 1.77 (m, 1H), 1.70 – 1.50 (m, 5H), 1.42 – 1.28 (m, 2H), 1.26 – 1.19 (m, 1H), 1.15 (s, 3H), 1.18 – 1.10 (m, 1H), 1.03 – 0.89 (m, 2H), 0.65 (s, 3H); **^{13}C NMR (151 MHz, DMSO-d$_6$)** δ 198.0, 170.8, 164.8, 158.1 (J^2_{CF} = 31.7 Hz), 154.5, 123.2, 117.4 (J^1_{CF} = 302.0 Hz), 58.4, 55.9, 54.7, 53.1, 49.7, 44.8, 43.4, 38.2, 37.7, 35.1, 35.1, 34.7, 33.6, 31.9, 31.6, 27.3, 23.7, 22.5, 22.2, 20.6, 16.9, 16.8, 13.1; **HRMS** (APCI+) [M+H]$^+$ calc. for $C_{28}H_{44}N_3O_3$, 470.3377, observed, 470.3383.

Preparation of (8*S*,9*S*,10*R*,13*S*,14*S*,17*S*)-10,13-dimethyl-17-((*E*)-1-(((methyl(2-((*S*)-pyrrolidin-2-yl)ethyl)carbamoyl)oxy)imino)ethyl)-1,2,6,7,8,9,10,11,12,13,14,15,16,17-tetradecahydro-3*H*-cyclopenta[a]phenanthren-3-one 2,2,2-trifluoroacetate (20n)

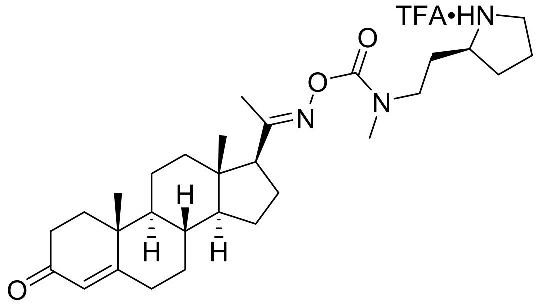

Prepared according to General Procedure F to afford the TFA salt. The product was in the form of a white solid (296 mg, 0.495 mmol, 95% yield). **^{1}H NMR (600 MHz, DMSO-d$_6$)** δ 9.21– 8.90 (m, 1H), 8.63 (br s, 1H), 5.63 (s, 1H), 3.51 (s, 1H), 3.41 – 3.35 (m, 1H), 3.32 (t, *J* = 7.2 Hz, 2H), 3.22 – 3.11 (m, 2H), 2.94 – 2.82 (m, 2H), 2.45 – 2.32 (m, 3H), 2.28 – 2.22 (m, 1H), 2.19 – 2.07 (m, 3H), 2.00 – 1.94 (m, 3H), 1.91 (s, 3H), 1.90 – 1.75 (m, 4H), 1.70 – 1.50 (m, 6H), 1.41 – 1.28 (m, 2H), 1.26 – 1.16 (m, 1H), 1.15 (s, 3H), 1.03 – 0.90 (m, 2H), 0.64 (s, 3H); **^{13}C NMR (151 MHz, DMSO-d$_6$)** δ 198.0, 170.9, 164.4, 158.1 (J^2_{CF} = 31.7 Hz),154.0, 123.2, 117.1 (J^1_{CF} = 302.0 Hz), 57.2, 55.9, 54.7, 53.1, 45.9, 44.4, 43.4, 38.2, 37.8, 35.1, 35.1, 33.6, 32.0, 31.6, 29.5, 23.7, 23.0, 22.5, 20.6, 16.9, 16.7, 13.1. Two ^{13}C signals not observed; **HRMS** (APCI+) [M+H]$^+$ calc. for $C_{29}H_{46}N_3O_3$, 484.3534, observed, 484.3541.

Preparation of (8*S*,9*S*,10*R*,13*S*,14*S*,17*S*)-17-((*E*)-1-((((((*S*)-azetidin-2-yl)methyl)(methyl)carbamoyl)oxy)imino)ethyl)-10,13-dimethyl-1,2,6,7,8,9,10,11,12,13,14,15,16,17-tetradecahydro-3*H*-cyclopenta[a]phenanthren-3-one 2,2,2-trifluoroacetate (20o)

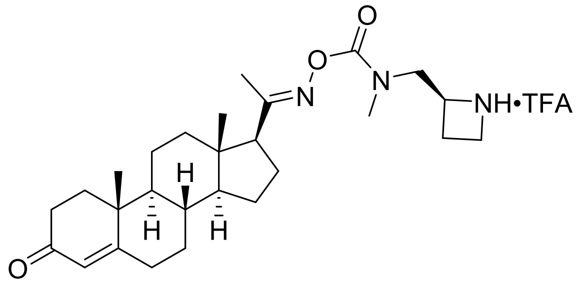

Prepared according to General Procedure F to afford the TFA salt. The product was in the form of a white solid (241 mg, 0.423 mmol, 87% yield). **^{1}H NMR (600 MHz, DMSO-d$_6$)** δ 8.82 (br s, 1H), 8.64 (br s, 1H), 5.64 (s, 1H), 4.63 – 4.54 (m, 1H), 3.98 – 3.86 (m, 1H), 3.77 – 3.65 (m, 2H), 3.58 – 3.46 (m, 1H), 2.93 (s, 3H), 2.44 – 2.29 (m, 5H), 2.28 – 2.22 (m, 1H), 2.19 – 2.10 (m, 2H), 2.00 – 1.94 (m, 1H), 1.92 (s, 3H), 1.89 – 1.84 (m, 1H), 1.84 – 1.77 (m, 1H), 1.70 – 1.50 (m, 5H), 1.42 – 1.28 (m, 2H), 1.26 – 1.19 (m, 1H), 1.18 – 1.11 (m, 1H), 1.14 (s, 3H), 1.03 – 0.90 (m, 2H), 0.64 (s, 3H); **^{13}C NMR (151 MHz, DMSO-d$_6$)** δ 198.0, 170.8, 165.0, 154.6, 157.9 (J^2_{CF} = 31.7 Hz), 123.2, 117.4 (J^1_{CF} = 302.0 Hz), 58.4, 55.9, 54.7, 53.1, 51.0, 43.4, 42.8, 38.2, 37.7, 35.1, 35.1, 33.6, 31.9, 31.6, 23.7, 22.8, 22.5, 20.6, 16.9, 16.8, 13.1. One ^{13}C signal not observed; **HRMS** (APCI+) $[M+H]^+$ calc. for $C_{27}H_{42}N_3O_3$, 456.3221, observed, 456.3220.

Preparation of (8*S*,9*S*,10*R*,13*S*,14*S*,17*S*)-10,13-dimethyl-17-((*E*)-1-(((methyl((*R*)-1-((*S*)-pyrrolidin-2-yl)ethyl)carbamoyl)oxy)imino)ethyl)-1,2,6,7,8,9,10,11,12,13,14,15,16,17-tetradecahydro-3*H*-cyclopenta[a]phenanthren-3-one 2,2,2-trifluoroacetate (20p)

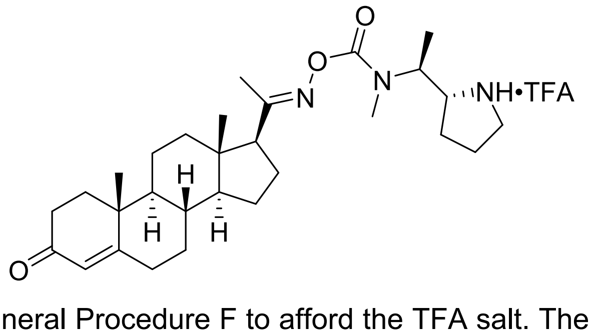

Prepared according to General Procedure F to afford the TFA salt. The product was in the form of a white solid (163 mg, 0.274 mmol, 80% yield). **^{1}H NMR (400 MHz, CDCl$_3$)** δ 10.79 – 8.61 (m, 2H), 5.72 (d, *J* = 1.6 Hz, 1H), 4.45 – 4.35 (m, 1H), 3.84 (s, 1H), 3.35 (s, 2H), 2.89 (s, 3H), 2.48 – 2.20 (m, 6H), 2.16 – 1.82 (m, 9H), 1.81 – 1.65 (m, 3H), 1.64 – 1.49 (m, 2H), 1.47 – 1.21 (m, 7H), 1.20 – 1.09 (m, 4H), 1.00 (dtd, *J* = 23.1, 12.0, 4.1 Hz, 2H), 0.71 (s, 3H); **^{13}C NMR (151 MHz,**

$CDCl_3$) δ 199.7, 171.3, 165.9, 162.2 (J^2_{CF} = 36.2 Hz), 155.9, 124.0, 116.4 (J^1_{CF} = 291.3 Hz), 63.4, 62.0, 56.9, 55.5, 53.8, 52.4, 46.0, 45.6, 44.2, 38.7, 35.8 (d), 34.0, 32.9, 31.9, 31.0, 29.8, 27.6, 25.0, 24.8, 24.2, 23.6, 23.2, 21.1, 17.5, 17.2, 15.7, 14.2, 13.6. Multiple peak splitting in ^{13}C spectrum; **HRMS** (APCI+) $[M+H]^+$ calc. for $C_{29}H_{46}N_3O_3$, 484.3534, observed, 484.3545.

Preparation of (8*S*,9*S*,10*R*,13*S*,14*S*,17*S*)-17-((*E*)-1-((((((*S*)-5-azaspiro[2.4]heptan-6-yl)methyl)(methyl)carbamoyl)oxy)imino)ethyl)-8,10,13-trimethyl-1,2,6,7,8,9,10,11,12,13,14,15,16,17-tetradecahydro-3*H*-cyclopenta[a]phenanthren-3-one 2,2,2-trifluoroacetate (20q)

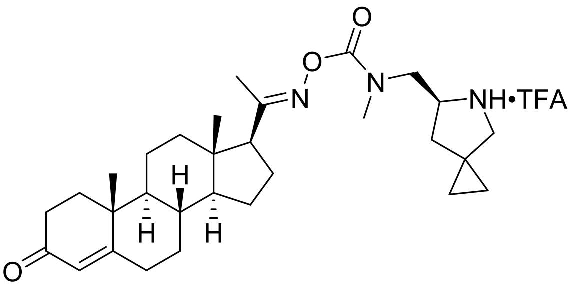

Prepared according to General Procedure F to afford the TFA salt. The product was in the form of a white solid (188 mg, 0.302 mmol, 92% yield). **^{1}H NMR (600 MHz, $CDCl_3$)** δ 11.55 (s, 1H), 8.53 (s, 1H), 5.72 (s, 1H), 4.30 (s, 1H), 3.97 – 3.86 (m, 1H), 3.48 – 3.38 (m, 1H), 3.25 (s, 2H), 3.04 (s, 3H), 2.45 – 2.18 (m, 6H), 2.04 – 1.97 (m, 2H), 1.96 (s, 3H), 1.93 – 1.82 (m, 3H), 1.80 – 1.64 (m, 3H), 1.63 – 1.50 (m, 2H), 1.43 (qd, *J* = 13.1, 3.8 Hz, 1H), 1.35 – 1.22 (m, 2H), 1.18 (s, 3H), 1.17 – 1.10 (m, 1H), 1.09 – 1.01 (m, 1H), 0.96 (ddd, *J* = 12.3, 10.6, 4.1 Hz, 1H), 0.79 – 0.66 (m, 7H); **^{13}C NMR (151 MHz, $CDCl_3$)** δ 199.6, 171.2, 166.4, 162.2 (J^2_{CF} = 35.3 Hz), 157.4, 124.0, 116.8 (J^1_{CF} = 292.6 Hz), 59.5, 56.8, 55.4, 53.8, 51.8, 51.6, 44.3, 38.7, 38.5, 36.4, 35.8, 35.7, 34.1, 32.9, 31.9, 24.2, 23.2, 21.1, 20.4, 17.5, 17.0, 13.5, 11.3, 10.7. Two ^{13}C signal not observed; **HRMS** (APCI+) $[M+H]^+$ calc. for $C_{30}H_{46}O_3N_3$, 496.3534, observed, 496.3549.

Preparation of (8*S*,9*S*,10*R*,13*S*,14*S*,17*S*)-17-((*E*)-1-((((((*S*)-4,4-difluoropyrrolidin-2-yl)methyl)(methyl)carbamoyl)oxy)imino)ethyl)-10,13-dimethyl-1,2,6,7,8,9,10,11,12,13,14,15,16,17-tetradecahydro-3*H*-cyclopenta[a]phenanthren-3-one 2,2,2-trifluoroacetate (20r)

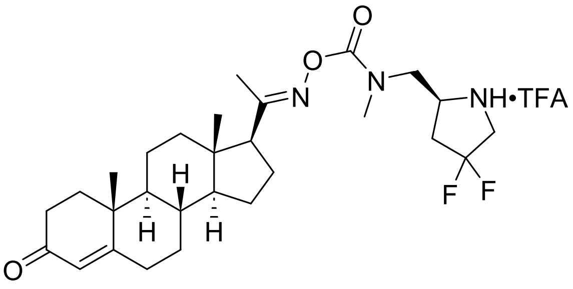

Prepared according to General Procedure F to afford the TFA salt. The product was in the form of a white solid (176 mg, 0.284 mmol, 86% yield). **^{1}H NMR (600 MHz, CDCl$_3$)** δ 9.44 (bs, 2H), 5.70 (s, 1H), 4.11 (t, *J* = 9.0 Hz, 1H), 4.02 (dd, *J* = 15.1, 8.3 Hz, 1H), 3.90 (q, *J* = 13.2 Hz, 1H), 3.83 – 3.71 (m, 1H), 3.49 – 3.40 (m, 1H), 3.00 (s, 3H), 2.61 – 2.46 (m, 2H), 2.45 – 2.22 (m, 5H), 2.12 (q, *J* = 11.3 Hz, 1H), 2.00 (ddd, *J* = 13.3, 5.1, 3.2 Hz, 1H), 1.92 (s, 3H), 1.85 (tt, *J* = 12.8, 3.0 Hz, 2H), 1.77 – 1.66 (m, 3H), 1.54 (dtd, *J* = 33.1, 12.4, 3.6 Hz, 2H), 1.41 (qd, *J* = 13.1, 4.0 Hz, 1H), 1.33 – 1.20 (m, 2H), 1.18 – 1.09 (m, 4H), 1.09 – 0.99 (m, 1H), 0.95 (ddd, *J* = 12.2, 10.7, 4.1 Hz, 1H), 0.68 (s, 3H); **^{13}C NMR (151 MHz, CDCl$_3$)** δ 199.6, 171.2, 166.3, 162.3 (J^2_{CF} = 35.2 Hz), 156.4, 126.8 (J^{32}_{CF2} = 252.4, 246.5 Hz), 124.0, 116.6 (J^1_{CF} = 292.0 Hz), 57.5, 56.5, 55.3, 53.5, 51.9 (J^{31}_{CF} = 34.5 Hz), 50.0, 44.3, 38.7, 38.3, 37.7 (J^{33}_{CF} = 24.6 Hz), 35.7, 35.4, 34.0, 32.9, 31.9, 24.2, 23.2, 21.0, 17.4, 16.8, 13.4. One ^{13}C signal not observed; **^{19}F NMR (565 MHz, CDCl$_3$)** δ -75.41 (s), -91.91 (dt, *J* = 238.2, 16.9 Hz), -97.37 – -99.68 (m); **HRMS** (APCI+) [M+H]$^+$ calc. for $C_{28}H_{42}O_3N_3F_2$, 506.3189, observed, 506.3186.

Preparation of (8*S*,9*S*,10*R*,13*S*,14*S*,17*S*)-10,13-dimethyl-17-((*E*)-1-(((methyl((1-(methylamino)cyclopropyl)methyl)carbamoyl)oxy)imino)ethyl)-1,2,6,7,8,9,10,11,12,13,14,15,16,17-tetradecahydro-3*H*-cyclopenta[a]phenanthren-3-one 2,2,2-trifluoroacetate (20s)

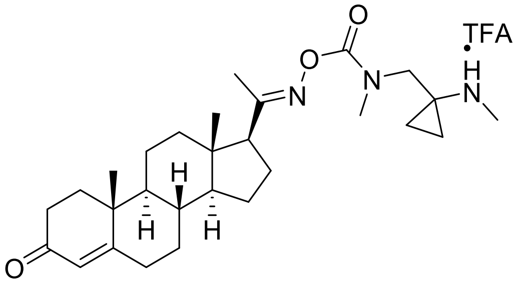

Prepared according to General Procedure F to afford the TFA salt. The product was in the form of a white solid (184 mg, 0.316 mmol, 90% yield). **^{1}H NMR (800 MHz, CDCl$_3$)** δ 9.54 (s, 1H), 5.72 (s, 1H), 3.57 (q, *J* = 15.6 Hz, 2H), 3.05 – 3.02 (m, 3H), 2.81 (s, 3H), 2.44 – 2.31 (m, 4H), 2.29 – 2.22 (m, 2H), 2.01 (ddt, *J* = 13.5, 5.5, 2.8 Hz, 1H), 1.97 – 1.95 (m, 3H), 1.92 (dt, *J* = 12.3, 3.4 Hz, 1H), 1.86 (dq, *J* = 11.3, 2.9 Hz, 1H), 1.80 – 1.66 (m, 3H), 1.62 – 1.52 (m, 2H), 1.46 – 1.40 (m, 1H), 1.39 – 1.30 (m, 3H), 1.27 (tdq, *J* = 12.3, 6.4, 2.8 Hz, 1H), 1.19 – 1.12 (m, 4H), 1.05 (tdt, *J* = 12.5, 6.8, 3.6 Hz, 1H), 0.97 (ddt, *J* = 15.1, 12.5, 3.4 Hz, 1H), 0.84 (dd, *J* = 4.8, 3.0 Hz, 2H), 0.72 (d, *J* = 3.1 Hz, 3H); **^{13}C NMR (201 MHz, CDCl$_3$)** δ 199.7, 171.3, 171.3, 166.1, 162.4 (J^2_{CF} = 34.8 Hz), 156.5, 124.0, 116.67 (J^1_{CF} = 293.0 Hz), 63.6, 56.8, 56.1, 55.5, 53.8, 51.4, 44.2, 40.4, 38.7, 38.5, 35.8 (d), 35.6 (d), 34.0, 32.9, 32.0, 30.9, 24.3, 23.2, 21.1, 17.5, 17.0, 13.6, 9.6. Multiple peak splitting in ^{13}C spectrum; **HRMS** (APCI+) [M+H]$^+$ calc. for $C_{28}H_{44}O_3N_3$, 470.3377, observed, 470.3381.

Preparation of (8*S*,9*S*,10*R*,13*S*,14*S*,17*S*)-10,13-dimethyl-17-((*E*)-1-(((methyl((1-(methylamino)cyclobutyl)methyl)carbamoyl)oxy)imino)ethyl)-1,2,6,7,8,9,10,11,12,13,14,15,16,17-tetradecahydro-3*H*-cyclopenta[a]phenanthren-3-one (20t)

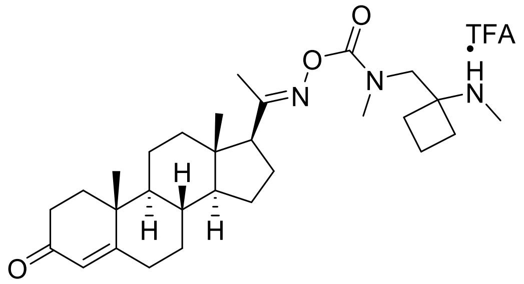

Prepared according to General Procedure F to afford the TFA salt. The product was in the form of a white solid (180 mg, 0.301 mmol, 88% yield). **^{1}H NMR (400 MHz, CDCl$_3$)** δ 9.60 (s, 2H), 5.72 (s, 1H), 3.68 (s, 2H), 3.13 (s, 3H), 2.68 (s, 3H), 2.63 – 2.49 (m, 2H), 2.47 – 2.32 (m, 4H), 2.32 – 2.18 (m, 3H), 2.17 – 1.98 (m, 5H), 1.96 (s, 3H), 1.95 – 1.82 (m, 3H), 1.81 – 1.64 (m, 3H), 1.63 – 1.21 (m, 6H), 1.18 (s, 3H), 1.15 – 0.84 (m, 3H), 0.72 (s, 3H); **^{13}C NMR (151 MHz, CDCl$_3$)** δ 199.6, 171.2, 166.3, 162.2 (J^2_{CF} = 35.0 Hz), 157.4, 124.0, 116.7 (J^1_{CF} = 292.3 Hz), 62.6, 59.3, 56.8, 56.5, 55.5, 55.4, 53.8, 52.7, 44.3, 38.7, 38.5, 37.9, 35.8 (d), 34.0, 32.9, 31.9, 31.4, 27.7 (d), 27.3, 24.2, 23.2, 21.1, 17.5, 17.1, 13.7, 13.6, 13.4 (d). Multiple peak splitting in ^{13}C spectrum; **HRMS** (ESI+) $[M+H]^+$ calc. for $C_{29}H_{46}O_3N_3$, 484.3534, observed, 484.3535.

Preparation of (8*S*,9*S*,10*R*,13*S*,14*S*,17*S*)-17-((*E*)-1-((((((2S,4S)-4-(dimethylamino)pyrrolidin-2-yl)methyl)(methyl)carbamoyl)oxy)imino)ethyl)-10,13-dimethyl-1,2,6,7,8,9,10,11,12,13,14,15,16,17-tetradecahydro-3*H*-cyclopenta[a]phenanthren-3-one 2,2,2-trifluoroacetate (20u)

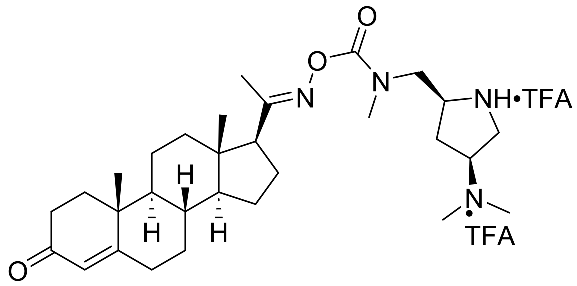

Prepared according to General Procedure F to afford the TFA salt. The product was in the form of a white solid (160 mg, 0.246 mmol, 69% yield). **^{1}H NMR (600 MHz, DMSO-d$_6$)** δ 5.63 (s, 1H), 3.82 – 3.71 (m, 1H), 3.69 – 3.22 (m, 8H), 3.00 – 2.86 (m, 3H), 2.69 – 2.53 (m, 5H), 2.44 – 2.33 (m, 4H), 2.28 – 2.22 (m, 1H), 2.19 – 2.09 (m, 2H), 2.00 – 1.94 (m, 1H), 1.93 (s, 3H), 1.90 – 1.83 (m, 1H), 1.83 – 1.73 (m, 2H), 1.69 – 1.50 (m, 5H), 1.41 – 1.29 (m, 2H), 1.26 – 1.18 (m, 1H), 1.15

(s, 3H), 1.18 – 1.10 (m, 1H), 1.03 – 0.90 (m, 2H), 0.64 (s, 3H); **^{13}C NMR (151 MHz, DMSO-d$_6$)** δ 198.0, 170.9, 164.9, 158.1 (J^2_{CF} = 31.7 Hz),154.5, 123.2, 117.4 (J^1_{CF} = 302.0 Hz), 62.8, 57.5, 55.9, 54.7, 53.1, 49.6, 45.6, 43.4, 42.0, 38.2, 37.7, 35.1, 35.1, 34.9, 33.6, 32.0, 31.6, 30.4, 23.7, 22.5, 20.6, 16.9, 16.8, 13.1. One ^{13}C signal not observed; **HRMS** (APCI+) $[M+H]^+$ calc. for $C_{30}H_{49}N_4O_3$, 513.3799, observed, 513.3804.

Preparation of (8*S*,9*S*,10*R*,13*S*,14*S*,17*S*)-17-((*E*)-1-(((((1*H*-imidazol-5-yl)methyl)(methyl)carbamoyl)oxy)imino)ethyl)-10,13-dimethyl-1,2,6,7,8,9,10,11,12,13,14,15,16,17-tetradecahydro-3*H*-cyclopenta[a]phenanthren-3-one 2,2,2-trifluoroacetate (20v)

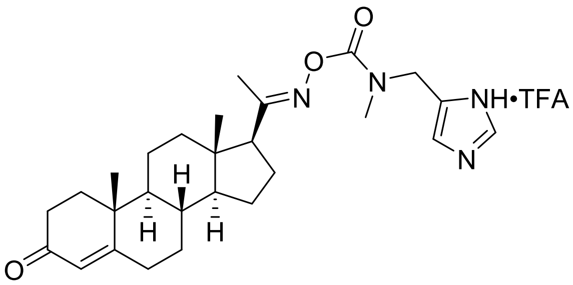

Prepared according to General Procedure F to afford the TFA salt. The product was in the form of a white solid (296 mg, 0.510 mmol, 77% yield). **^{1}H NMR (600 MHz, DMSO-d$_6$)** δ 8.97 (s, 1H), 7.59 (s, 1H), 5.63 (s, 1H), 4.52 (s, 2H), 3.50 (br s, 2H), 2.91 (s, 3H), 2.45 – 2.34 (m, 3H), 2.28 – 2.21 (m, 1H), 2.20 – 2.09 (m, 2H), 2.01 – 1.94 (m, 1H), 1.94 – 1.83 (m, 4H), 1.83 – 1.76 (m, 1H), 1.69 – 1.50 (m, 5H), 1.42 – 1.28 (m, 2H), 1.25 – 1.18 (m, 1H), 1.18 – 1.11 (m, 1H), 1.15 (s, 3H), 1.03 – 0.88 (m, 2H), 0.64 (s, 3H); **^{13}C NMR (151 MHz, DMSO-d$_6$)** δ 198.0, 170.9, 164.9, 158.1 (J^2_{CF} = 31.7 Hz), 154.0, 134.8, 129.5, 123.2, 117.6, 117.4 (J^1_{CF} = 302.0 Hz), 55.9, 54.7, 53.1, 43.4, 42.6, 40.1, 37.7, 35.1, 35.1, 33.6, 31.9, 31.6, 23.7, 22.5, 20.6, 16.9, 16.7, 13.1. One ^{13}C signal not observed; **HRMS** (APCI+) $[M+H]^+$ calc. for $C_{27}H_{39}N_4O_3$, 467.3017, observed, 467.3023.

Preparation of (8*S*,9*S*,10*R*,13*S*,14*S*,17*S*)-17-((*E*)-1-(((bis(2-aminoethyl)carbamoyl)oxy)imino)ethyl)-10,13-dimethyl-1,2,6,7,8,9,10,11,12,13,14,15,16,17-tetradecahydro-3*H*-cyclopenta[a]phenanthren-3-one bis(2,2,2-trifluoroacetate) (20w)

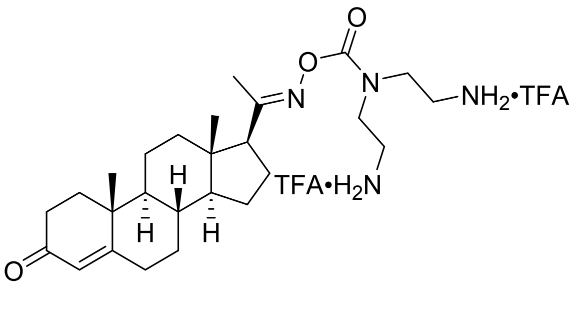

Prepared according to General Procedure F to afford the TFA salt. The product was in the form of a white solid (330 mg, 0.481 mmol, 83% yield). **^{1}H NMR (600 MHz, DMSO-d_6)** δ 8.15 – 7.83 (m, 6H), 5.63 (s, 1H), 3.53 – 3.45 (m, 4H), 3.03 – 2.98 (m, 4H), 2.45 – 2.36 (m, 3H), 2.28 – 2.22 (m, 1H), 2.19 – 2.11 (m, 2H), 2.00 – 1.97 (m, 1H), 1.95 (s, 3H), 1.89 – 1.83 (m, 1H), 1.83 – 1.77 (m, 1H), 1.70 – 1.50 (m, 5H), 1.40 – 1.29 (m, 2H), 1.26 – 1.16 (m, 2H), 1.14 (s, 3H), 1.03 – 0.90 (m, 2H), 0.65 (s, 3H); **^{13}C NMR (151 MHz, DMSO-d_6)** δ 198.0, 170.9, 165.3, 158.5 (J^2_{CF} = 31.7 Hz), 154.1, 123.2, 117.1 (J^1_{CF} = 302.0 Hz), 55.9, 54.7, 53.1, 44.9, 43.5, 38.2, 37.8, 37.2, 35.1, 35.1, 33.6, 32.0, 31.6, 23.7, 22.6, 20.6, 17.0, 16.9, 13.1; **HRMS** (APCI+) $[M+H]^+$ calc. for $C_{26}H_{43}N_4O_3$, 459.3330, observed, 459.3329.

Preparation of (*E*)-9-((8*S*,9*S*,10*R*,13*S*,14*S*,17*S*)-10,13-dimethyl-3-oxo-2,3,6,7,8,9,10,11,12,13,14,15,16,17-tetradecahydro-1*H*-cyclopenta[a]phenanthren-17-yl)-5-(2-(methylamino)ethyl)-7-oxa-2,5,8-triazadec-8-en-6-one dihydrochloride (20x)

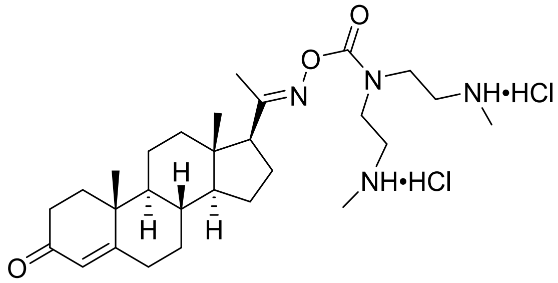

Prepared according to General Procedure F. The product was in the form of a white solid (152 mg, 0.272 mmol, 78% yield). **^{1}H NMR (600 MHz, DMSO-d_6)** δ 9.50 – 8.88 (m, 4H), 5.63 (s, 1H), 3.68 – 3.56 (m, 4H), 3.14 – 3.05 (m, 4H), 2.54 (s, 6H), 2.44 – 2.35 (m, 3H), 2.28 – 2.21 (m, 1H), 2.19 – 2.10 (m, 2H), 2.00 (s, 3H), 1.99 – 1.94 (m, 1H), 1.89 – 1.84 (m, 1H), 1.83 – 1.76 (m, 1H), 1.68 – 1.50 (m, 5H), 1.40 – 1.29 (m, 2H), 1.26 – 1.15 (m, 2H), 1.14 (s, 3H), 1.02 – 0.90 (m, 2H), 0.65 (s, 3H); **^{13}C NMR (151 MHz, DMSO-d_6)** δ 198.1, 170.9, 165.5, 154.1, 123.2, 56.0, 54.7, 53.1, 46.4, 43.7, 43.5, 38.2, 37.7, 35.1, 35.1, 33.6, 32.6, 32.0, 31.6, 23.7, 22.6, 20.6, 17.4, 16.9, 13.1; **HRMS** (APCI+) $[M+H]^+$ calc. for $C_{28}H_{47}N_4O_3$, 487.3643, not observed. Minor impurity signals visible in ^{13}C NMR due to rapid degradation of compound.

Preparation of (*E*)-9-((8*S*,9*S*,10*R*,13*S*,14*S*,17*S*)-10,13-dimethyl-3-oxo-2,3,6,7,8,9,10,11,12,13,14,15,16,17-tetradecahydro-1H-cyclopenta[a]phenanthren-17-yl)-5-(2-(dimethylamino)ethyl)-2-methyl-7-oxa-2,5,8-triazadec-8-en-6-one (20y)

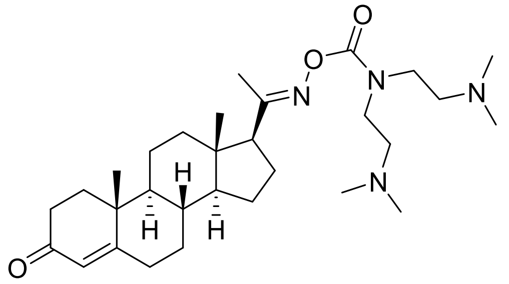

Prepared according to General Procedure E using commercially available *N*1-(2-(dimethylamino)ethyl)-*N*2,*N*2-dimethylethane-1,2-diamine. The product was in the form of a white solid (386 mg, 0.750 mmol, 62% yield). **^{1}H NMR (600 MHz, DMSO-d$_6$)** δ 5.63 (s, 1H), 3.31 (t, *J* = 6.9 Hz, 4H), 2.44 – 2.32 (m, 7H), 2.27 – 2.22 (m, 1H), 2.19 – 2.12 (m, 2H), 2.15 (s, 12H), 2.00 – 1.92 (m, 1H), 1.90 (s, 3H), 1.89 – 1.85 (m, 1H), 1.84 – 21.77 (m, 1H), 1.69 – 1.50 (m, 5H), 1.43 – 1.27 (m, 2H), 1.25 – 1.18 (m, 1H), 1.18 – 1.12 (m, 1H), 1.15 (s, 3H), 1.03 – 0.90 (m, 2H), 0.64 (s, 3H). **^{13}C NMR (151 MHz, DMSO-d$_6$)** δ 198.0, 170.9, 163.8, 153.6, 123.2, 57.3*, 55.9, 54.7, 53.1, 45.4, 45.1*, 43.4, 38.2, 37.8, 35.1, 35.1, 33.6, 31.9, 31.6, 23.7, 22.5, 20.6, 16.9, 16.8, 13.1. One ^{13}C signal not observed; **HRMS** (APCI+) $[M+H]^+$ calc. for $C_{30}H_{51}N_4O_3$, 515.3956, observed, 515.3964.

Preparation of (*E*)-2-((8*S*,9*S*,10*R*,13*S*,14*S*,17*S*)-10,13-dimethyl-3-oxo-2,3,6,7,8,9,10,11,12,13,14,15,16,17-tetradecahydro-1*H*-cyclopenta[a]phenanthren-17-yl)-11-hydroxy-6-(2-hydroxyethyl)-4-oxa-3,6,9-triazaundec-2-en-5-one hydrochloride (20z)

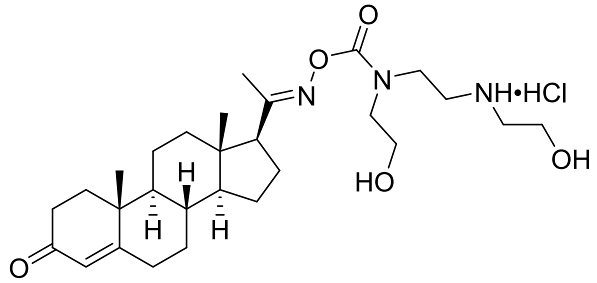

Prepared according to General Procedure E using commercially available 2,2'-(ethane-1,2-diylbis(azanediyl))bis(ethan-1-ol), followed by treatment with HCl in dioxane (4M). The product was in the form of a white solid (163 mg, 0.302 mmol, 69% yield). **^{1}H NMR (600 MHz, DMSO-d$_6$)** δ 9.18 – 8.68 (m, 2H), 5.63 (s, 1H), 5.28 (t, *J* = 5.0 Hz, 1H), 5.03 (s, 1H), 3.69 – 3.63 (m, 3H), 3.62 – 3.57 (m, 1H), 3.57 – 3.52 (m, 2H), 3.33 – 3.29 (m, 2H, masked by H_2O signal), 3.16 – 3.09 (m, 2H), 3.03 – 2.97 (m, 2H), 2.45 – 2.33 (m, 3H), 2.28 – 2.21 (m, 1H), 2.21 – 2.11 (m, 2H), 2.01 – 1.83 (m, 5H), 1.84 – 1.75 (m, 1H), 1.70 – 1.49 (m, 5H), 1.42 – 1.28 (m, 2H), 1.26 – 1.16 (m, 2H), 1.14 (s, 3H), 1.05 – 0.88 (m, 2H), 0.65 (s, 3H); **^{13}C NMR (151 MHz, DMSO-d$_6$)** δ 198.0, 170.9, 164.7, 154.2, 123.2, 59.1, 56.3, 55.9, 54.7, 53.1, 50.1, 49.2, 45.3, 44.8, 44.2, 43.4, 38.2,

37.8, 35.1, 35.1, 33.6, 31.9, 31.6, 23.7, 22.5, 20.6, 16.9, 13.1. ^{13}C peak splitting observed; **HRMS** (APCI+) $[M+H]^+$ calc. for $C_{28}H_{46}N_3O_5$, 504.3437, observed, 504.3437.

Preparation of (*E*)-1-((3*R*,8*R*,9*S*,10*S*,13*S*,14*S*,17*S*)-3-hydroxy-10,13-dimethylhexadecahydro-1*H*-cyclopenta[a]phenanthren-17-yl)ethan-1-one O-(methyl(2-(methylamino)ethyl)carbamoyl) oxime 2,2,2-trifluoroacetate (21a)

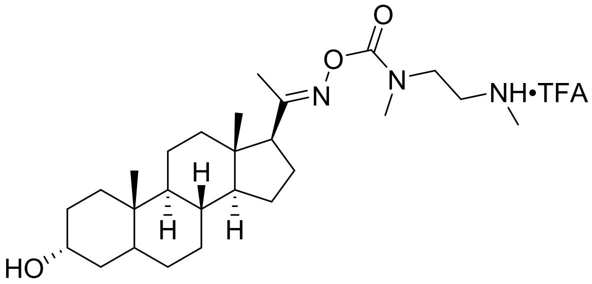

Prepared according to General Procedure F to afford the TFA salt. The product was in the form of a white solid (139 mg, 0.248 mmol, 82% yield). **^{1}H NMR (600 MHz, $CDCl_3$)** δ 9.38 (d, *J* = 74.8 Hz, 2H), 4.03 (t, *J* = 2.8 Hz, 1H), 3.87 – 3.71 (m, 1H), 3.64 – 3.50 (m, 1H), 3.30 – 3.15 (m, 2H), 2.97 (s, 3H), 2.72 (s, 3H), 2.49 (s, 2H), 2.33 (t, *J* = 9.0 Hz, 1H), 2.08 – 2.04 (m, 1H), 1.93 (s, 3H), 1.81 (dd, *J* = 8.3, 2.7 Hz, 1H), 1.73 – 1.62 (m, 4H), 1.62 – 1.41 (m, 5H), 1.40 – 1.26 (m, 3H), 1.26 – 1.09 (m, 5H), 0.95 (qd, *J* = 12.3, 5.2 Hz, 1H), 0.81 – 0.77 (m, 1H), 0.77 (s, 3H), 0.63 (s, 3H); **^{13}C NMR (151 MHz, $CDCl_3$)** δ 167.1, 162.0 (J^2_{CF} = 35.0 Hz), 156.8, 116.7 (J^1_{CF} = 292.8 Hz), 66.6, 56.7, 56.0, 54.4, 47.8, 46.5, 44.7, 39.2, 38.8, 36.2, 35.9, 35.7, 34.7, 33.6, 32.3, 32.0, 29.1, 28.6, 24.3, 23.3, 20.8, 16.8, 13.7, 11.3; **HRMS** (APCI+) $[M+H]^+$ calc. for $C_{26}H_{46}O_3N_3$, 448.3534, observed, 448.3535.

Preparation of (*E*)-1-((3*R*,8*R*,9*S*,10*S*,13*S*,14*S*,17*S*)-3-hydroxy-10,13-dimethylhexadecahydro-1*H*-cyclopenta[a]phenanthren-17-yl)ethan-1-one O-(cyclopropyl(2-(methylamino)ethyl)carbamoyl) oxime 2,2,2-trifluoroacetate (21b)

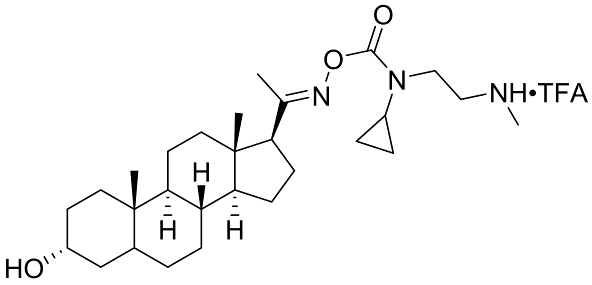

Prepared according to General Procedure F to afford the TFA salt. The product was in the form of a white solid (135 mg, 0.229 mmol, 79%). **^{1}H NMR (600 MHz, $CDCl_3$)** δ 9.43 (d, *J* = 129.7 Hz, 2H), 4.04 (t, *J* = 2.8 Hz, 1H), 3.83 (ddd, *J* = 15.4, 8.2, 3.1 Hz, 1H), 3.59 (ddd, *J* = 15.3, 6.5, 3.2 Hz, 1H), 3.38 – 3.14 (m, 2H), 2.76 (tt, *J* = 7.1, 3.6 Hz, 1H), 2.70 (s, 3H), 2.35 (t, *J* = 9.1 Hz, 1H),

2.12 – 1.95 (m, 6H), 1.83 (dd, *J* = 8.5, 2.7 Hz, 1H), 1.77 – 1.42 (m, 9H), 1.41 – 1.10 (m, 9H), 0.97 (qd, *J* = 12.2, 5.2 Hz, 1H), 0.87 – 0.77 (m, 6H), 0.77 – 0.68 (m, 2H), 0.65 (s, 3H); **^{13}C NMR (151 MHz, CDCl$_3$)** δ 167.7, 162.0 (J^2_{CF} = 34.3 Hz),158.5, 116.8 (J^1_{CF} = 292.3 Hz), 66.7, 56.6, 56.0, 54.4, 49.2, 45.4, 44.7, 39.2, 38.7, 36.3, 35.9, 35.7, 33.6, 32.4, 32.0, 29.3, 29.2, 28.6, 24.3, 23.4, 20.8, 17.0, 13.8, 11.3, 8.5, 8.0; **HRMS** (APCI+) [M+H]+ calc. for $C_{28}H_{48}O_3N_3$, 474.3701, observed, 474.3690.

Preparation of (*E*)-1-((3*R*,8*R*,9*S*,10*S*,13*S*,14*S*,17*S*)-3-hydroxy-10,13-dimethylhexadecahydro-1*H*-cyclopenta[a]phenanthren-17-yl)ethan-1-one O-(isopropyl(2-(methylamino)ethyl)carbamoyl) oxime 2,2,2-trifluoroacetate (21c)

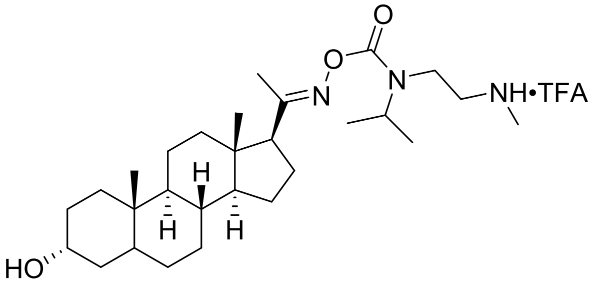

Prepared according to General Procedure F to afford the TFA salt. The product was in the form of a white solid (130 mg, 0.220 mmol, 76%). **^{1}H NMR (600 MHz, CDCl$_3$)** δ 9.55 (d, *J* = 57.2 Hz, 2H), 4.24 (s, 1H), 4.04 (t, *J* = 2.8 Hz, 1H), 3.63 (dt, *J* = 15.8, 5.7 Hz, 1H), 3.54 – 3.47 (m, 1H), 3.26 – 3.17 (m, 2H), 2.72 (s, 3H), 2.36 (t, *J* = 9.1 Hz, 1H), 2.15 – 2.04 (m, 1H), 1.94 (s, 3H), 1.88 – 1.77 (m, 2H), 1.75 – 1.64 (m, 4H), 1.64 – 1.43 (m, 5H), 1.40 – 1.25 (m, 5H), 1.24 – 1.10 (m, 9H), 0.96 (qd, *J* = 12.3, 5.3 Hz, 1H), 0.83 – 0.76 (m, 4H), 0.66 (s, 3H); **^{13}C NMR (151 MHz, CDCl$_3$)** δ 167.3, 162.2 (J^2_{CF} = 34.8 Hz), 157.3, 116.9 (J^1_{CF} = 293.8 Hz), 66.7, 56.7, 56.0, 54.4, 51.1, 48.4, 44.7, 39.2, 38.8, 36.3, 36.0, 35.7, 33.6, 32.4, 32.0, 29.1, 28.6, 24.3, 23.3, 20.8, 20.7, 17.0, 13.8, 11.3. Two ^{13}C signal not observed; **HRMS** (APCI+) [M+H]+ calc. for $C_{28}H_{50}O_3N_3$, 476.3858, observed, 476.3847.

Preparation of (*E*)-1-((3*R*,8*R*,9*S*,10*S*,13*S*,14*S*,17*S*)-3-hydroxy-10,13-dimethylhexadecahydro-1*H*-cyclopenta[a]phenanthren-17-yl)ethan-1-one O-(cyclobutyl(2-(methylamino)ethyl)carbamoyl) oxime 2,2,2-trifluoroacetate (21d)

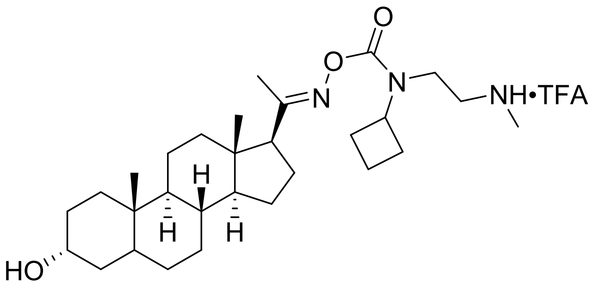

Prepared according to General Procedure F to afford the TFA salt. The product was in the form of a white solid (133 mg, 0.222 mmol, 78%). **^{1}H NMR (600 MHz, CDCl$_3$)** δ 9.44 (d, *J* = 97.4 Hz, 2H), 4.34 – 4.27 (m, 1H), 4.04 (p, *J* = 2.8 Hz, 1H), 3.83 – 3.75 (m, 1H), 3.67 – 3.60 (m, 1H), 3.17 (s, 2H), 2.71 (s, 3H), 2.35 (t, *J* = 9.0 Hz, 1H), 2.26 – 2.15 (m, 2H), 2.13 – 2.00 (m, 3H), 1.95 (s, 3H), 1.82 (dd, *J* = 8.4, 2.7 Hz, 1H), 1.73 – 1.44 (m, 11H), 1.38 – 1.29 (m, 3H), 1.28 – 1.07 (m, 6H), 0.96 (qd, *J* = 12.3, 5.3 Hz, 1H), 0.84 – 0.79 (m, 1H), 0.77 (s, 3H), 0.64 (s, 3H); **^{13}C NMR (151 MHz, CDCl$_3$)** δ 167.3, 162.0 (J^2_{CF} = 32.6 Hz), 157.1, 116.9 (J^1_{CF} = 289.7 Hz), 66.7, 56.6, 56.0, 54.1, 51.0, 50.4, 44.7, 40.5, 39.2, 38.7, 36.3, 36.2, 35.9, 35.7, 33.6, 32.4, 32.0, 29.3, 29.2, 29.1, 28.6, 24.3, 23.3, 20.8, 17.0, 14.7, 13.7, 11.3; **HRMS** (APCI+) [M+H]+ calc. for $C_{29}H_{50}O_3N_3$, 488.3858, observed, 488.3846.

Preparation of (*E*)-1-((3*R*,8*R*,9*S*,10*S*,13*S*,14*S*,17*S*)-3-hydroxy-10,13-dimethylhexadecahydro-1*H*-cyclopenta[a]phenanthren-17-yl)ethan-1-one O-((2-(methylamino)ethyl)(3-morpholinopropyl)carbamoyl) oxime 2,2,2-trifluoroacetate (21j)

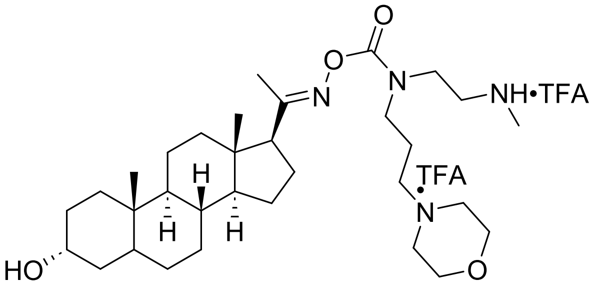

Prepared according to General Procedure F to afford the TFA salt. The product was in the form of a white solid (131 mg, 0.193 mmol, 75%). **^{1}H NMR (600 MHz, DMSO-d$_6$)** δ 8.66 (s, 2H), 4.18 (s, 1H), 3.80 (t, *J* = 3.0 Hz, 1H), 3.77 – 3.57 (m, 3H), 3.52 (s, 2H), 3.42 – 3.23 (m, 8H), 3.09 (t, *J* = 6.3 Hz, 2H), 2.59 (s, 3H), 2.37 (t, *J* = 9.1 Hz, 1H), 2.12 (td, *J* = 12.8, 6.6 Hz, 1H), 1.93 (s, 3H), 1.82 (dt, *J* = 12.0, 3.5 Hz, 2H), 1.67 – 1.59 (m, 3H), 1.58 – 1.44 (m, 4H), 1.39 – 1.09 (m, 12H), 0.96 – 0.86 (m, 1H), 0.73 (s, 4H), 0.59 (s, 3H); **^{13}C NMR (151 MHz, DMSO-d$_6$)** δ 164.9, 158.3 (J^2_{CF} = 31.2 Hz), 154.3, 117.2 (J^1_{CF} = 299.7 Hz), 64.0, 56.1, 55.4, 53.9, 51.8, 46.6, 43.7, 38.5, 38.2, 35.7, 35.6, 35.2, 32.8, 32.0, 31.7, 28.6, 28.2, 23.7, 22.5, 20.3, 17.0, 13.2, 11.1. Two ^{13}C signal not observed; **HRMS** (ESI+) [M+H]+ calc. for $C_{32}H_{57}O_4N_4$, 561.4374, observed, 561.4371.

Preparation of (*E*)-1-((3*R*,8*R*,9*S*,10*S*,13*S*,14*S*,17*S*)-3-hydroxy-10,13-dimethylhexadecahydro-1H-cyclopenta[a]phenanthren-17-yl)ethan-1-one O-((2-(1*H*-imidazol-1-yl)ethyl)(2-(methylamino)ethyl)carbamoyl) oxime 2,2,2-trifluoroacetate (21k)

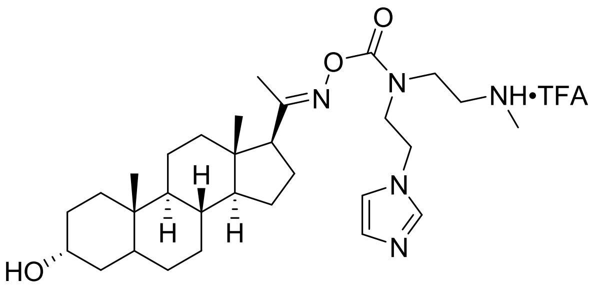

Prepared according to General Procedure F to afford the TFA salt. The product was in the form of a white solid (128 mg, 0.199 mmol, 74%). **^{1}H NMR (400 MHz, DMSO-d$_6$)** δ 9.13 (s, 1H), 8.78 (d, *J* = 100.5 Hz, 2H), 7.77 (s, 1H), 7.64 (s, 1H), 4.38 (t, *J* = 5.6 Hz, 2H), 3.81 (s, 1H), 3.74 – 3.67 (m, 2H), 3.55 – 3.44 (m, 4H), 3.09 (s, 2H), 2.58 (s, 3H), 2.32 (s, 1H), 2.07 (d, *J* = 9.3 Hz, 1H), 1.94 – 1.77 (m, 4H), 1.68 – 1.46 (m, 7H), 1.41 – 1.08 (m, 11H), 0.91 (q, *J* = 10.5 Hz, 1H), 0.73 (s, 4H), 0.55 (s, 3H); **^{13}C NMR (151 MHz, DMSO-d$_6$)** δ 164.8, 157.9 (J^2_{CF} = 31.3 Hz), 154.1, 135.9, 122.3, 117.2 (J^1_{CF} = 300.2 Hz), 64.0, 56.0, 55.4, 53.9, 46.4, 43.6, 42.9, 38.5, 38.1, 35.7, 35.6, 35.2, 32.9, 32.0, 31.7, 28.6, 28.2, 23.7, 22.4, 20.3, 17.0, 13.2, 11.2. Two ^{13}C signal not observed; **HRMS** (APCI+) [M+H]+ calc. for $C_{30}H_{50}O_3N_5$, 528.3919, observed, 528.3909.

7.5 NMR Spectra of Final Compounds

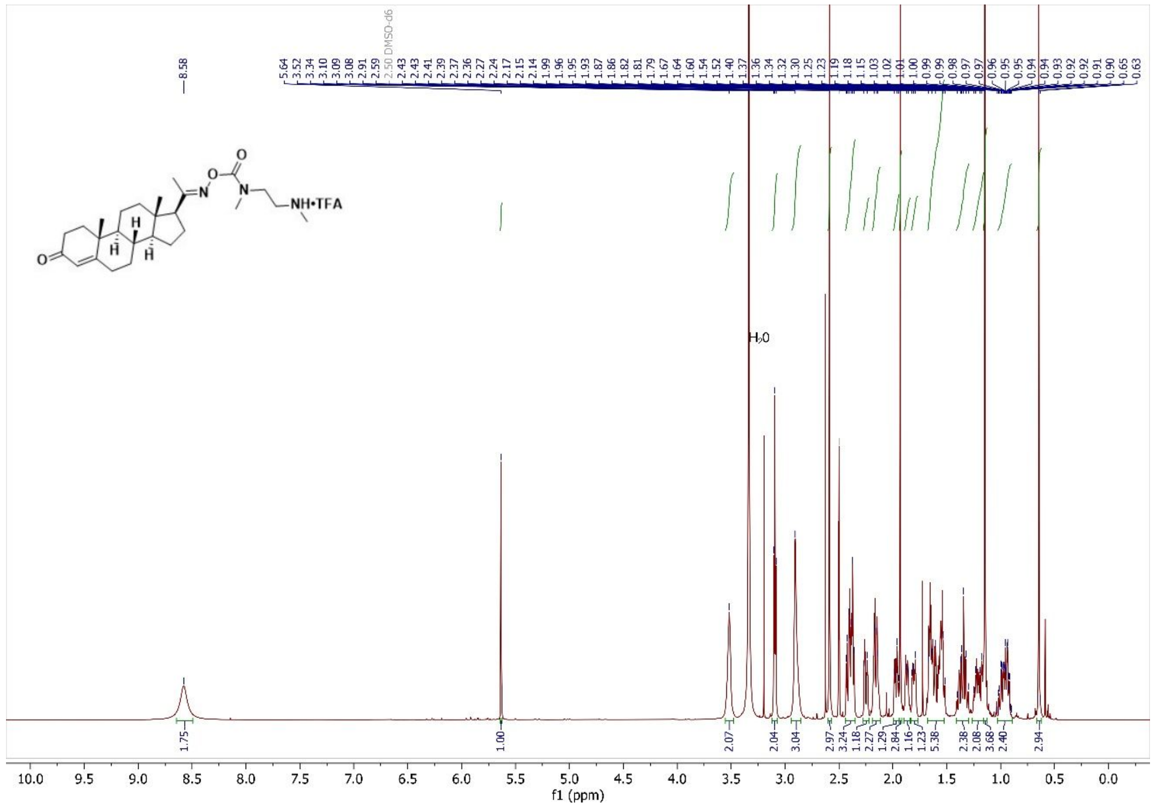

^{1}H NMR of Compound **20a** (600 MHz, DMSO-d_6)

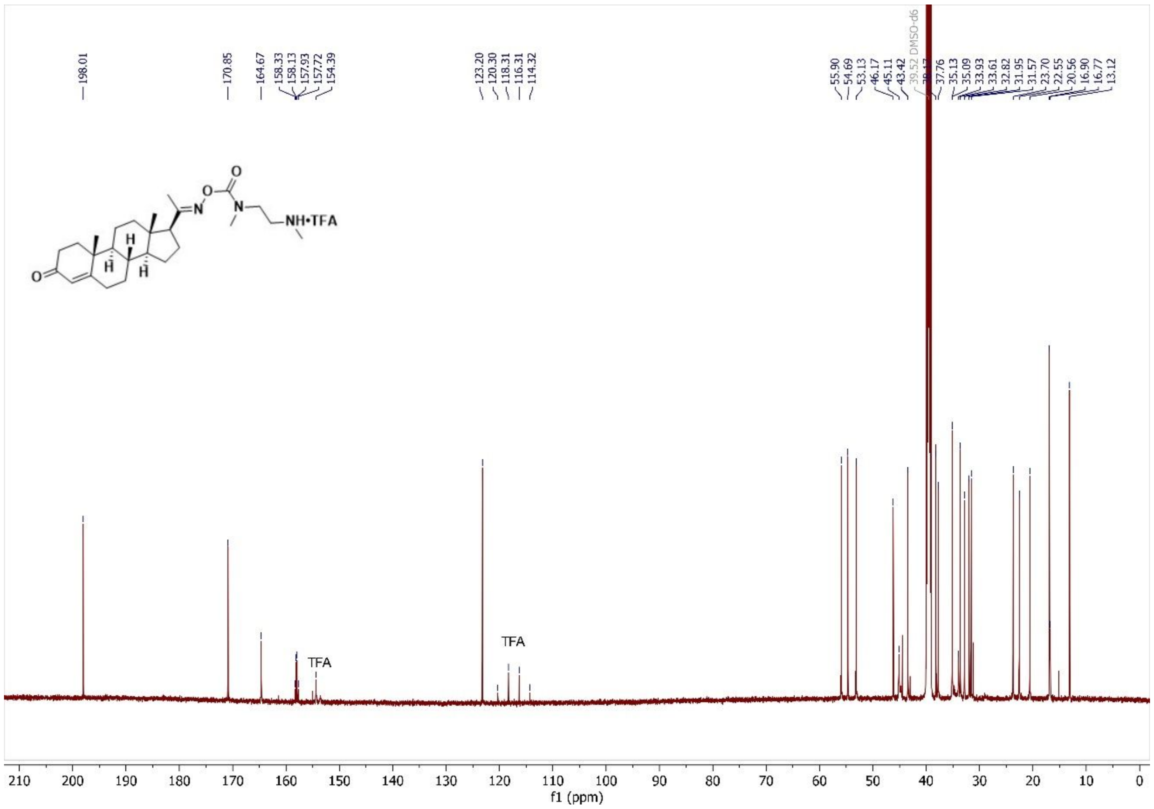

^{13}C NMR of Compound **20a** (151 MHz, DMSO-d_6)

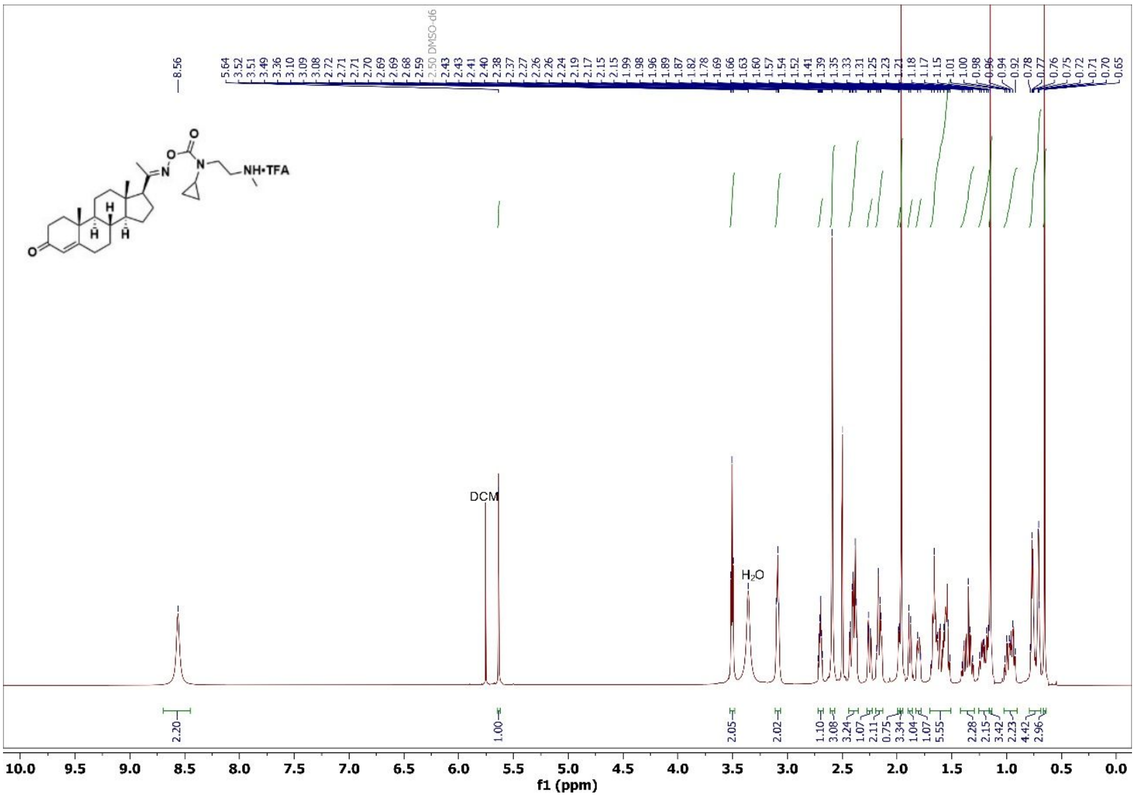

^{1}H NMR of Compound **20b** (600 MHz, DMSO-d_6)

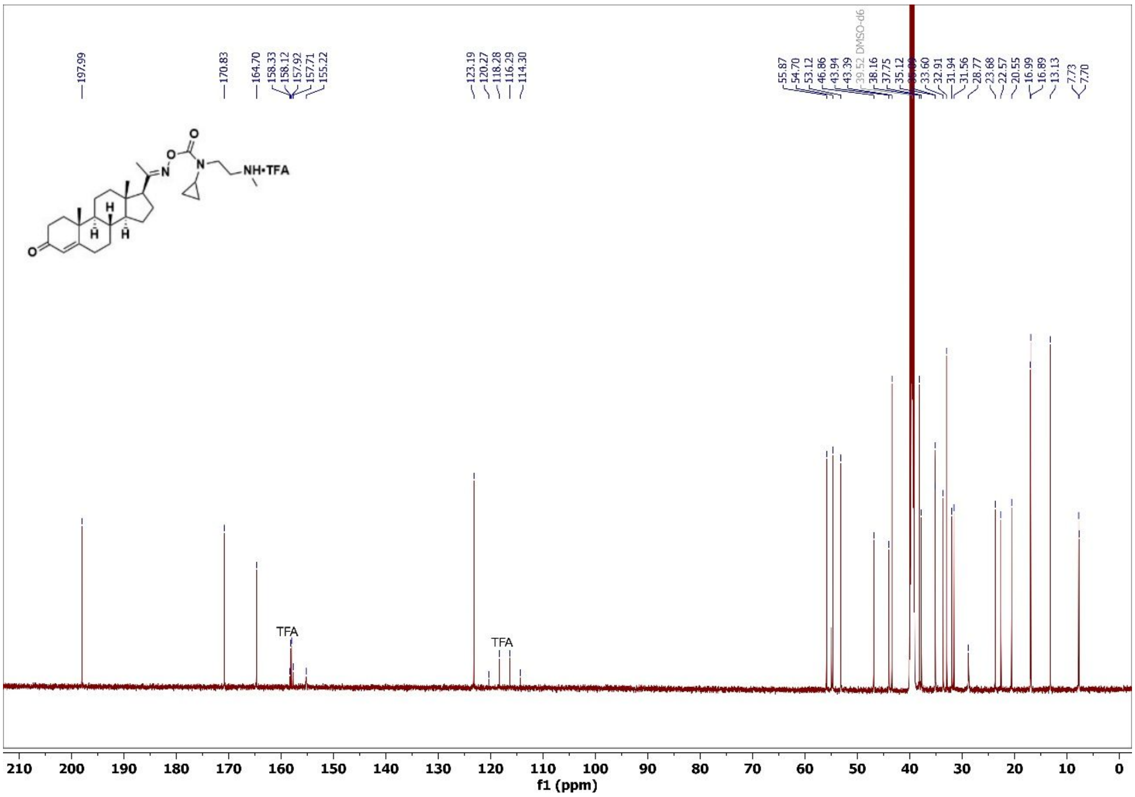

^{13}C NMR of Compound **20b** (151 MHz, DMSO-d_6)

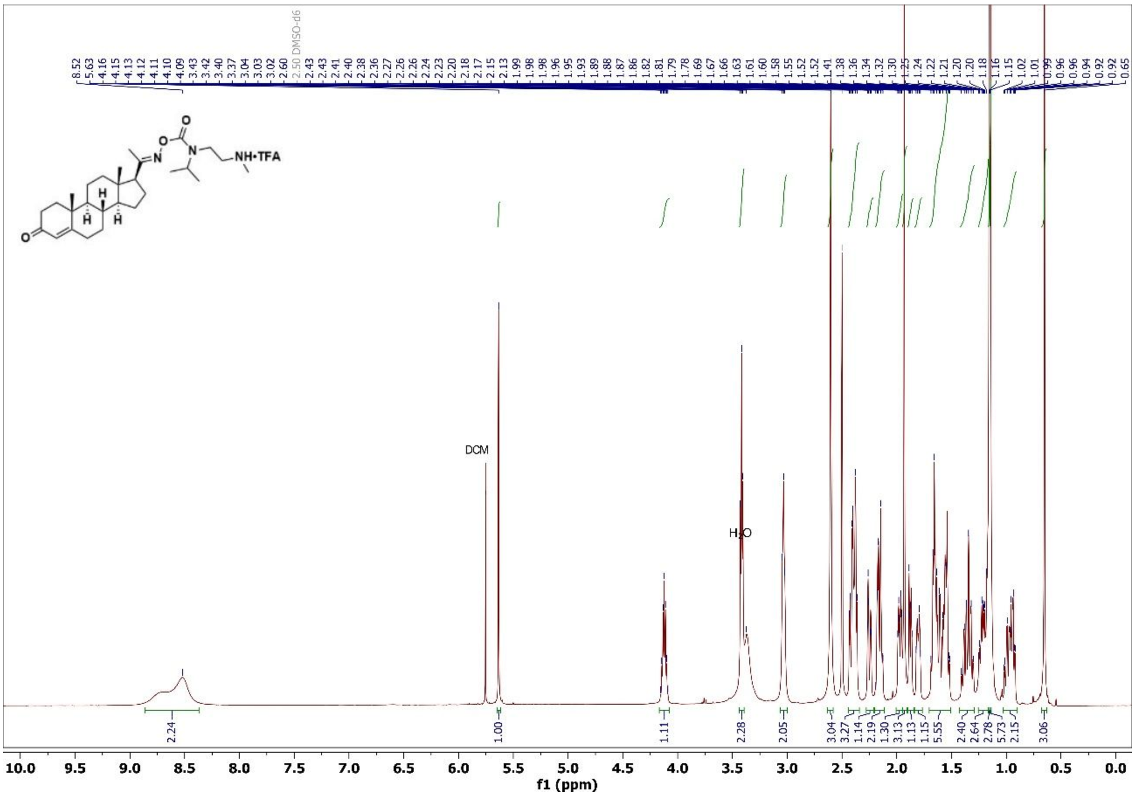

^{1}H NMR of Compound **20c** (600 MHz, DMSO-d_6)

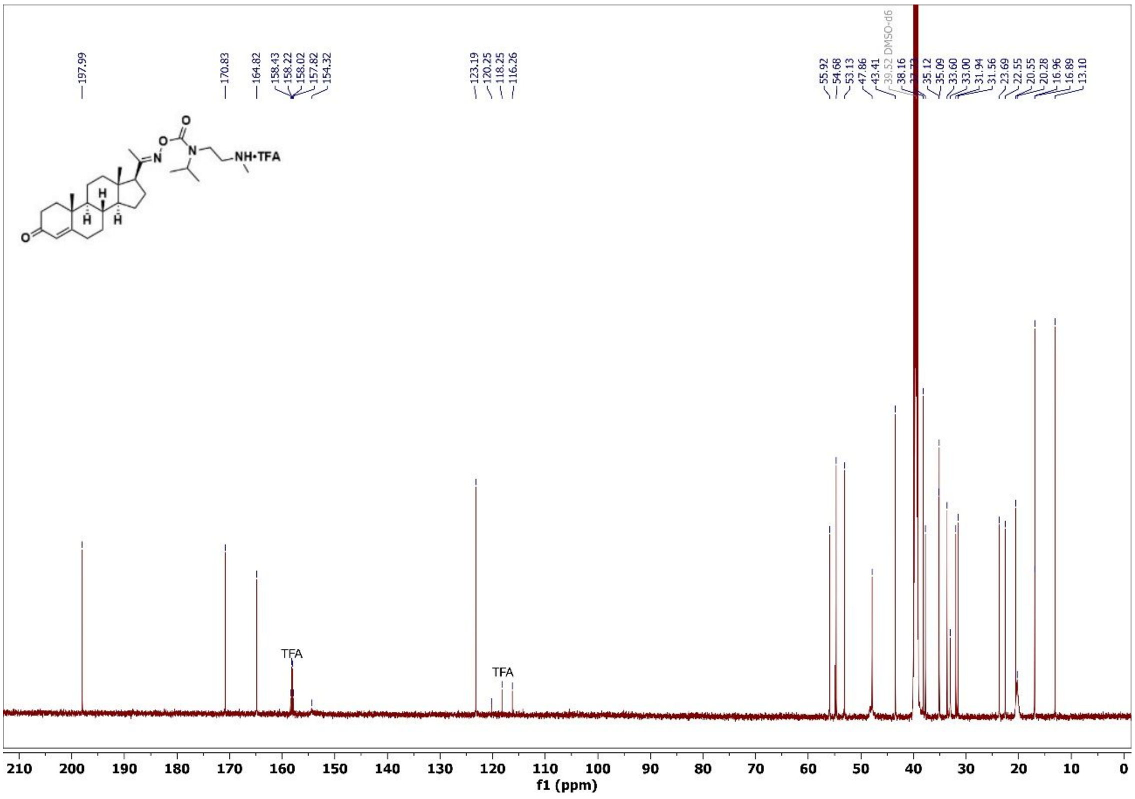

^{13}C NMR of Compound **20c** (151 MHz, DMSO-d_6)

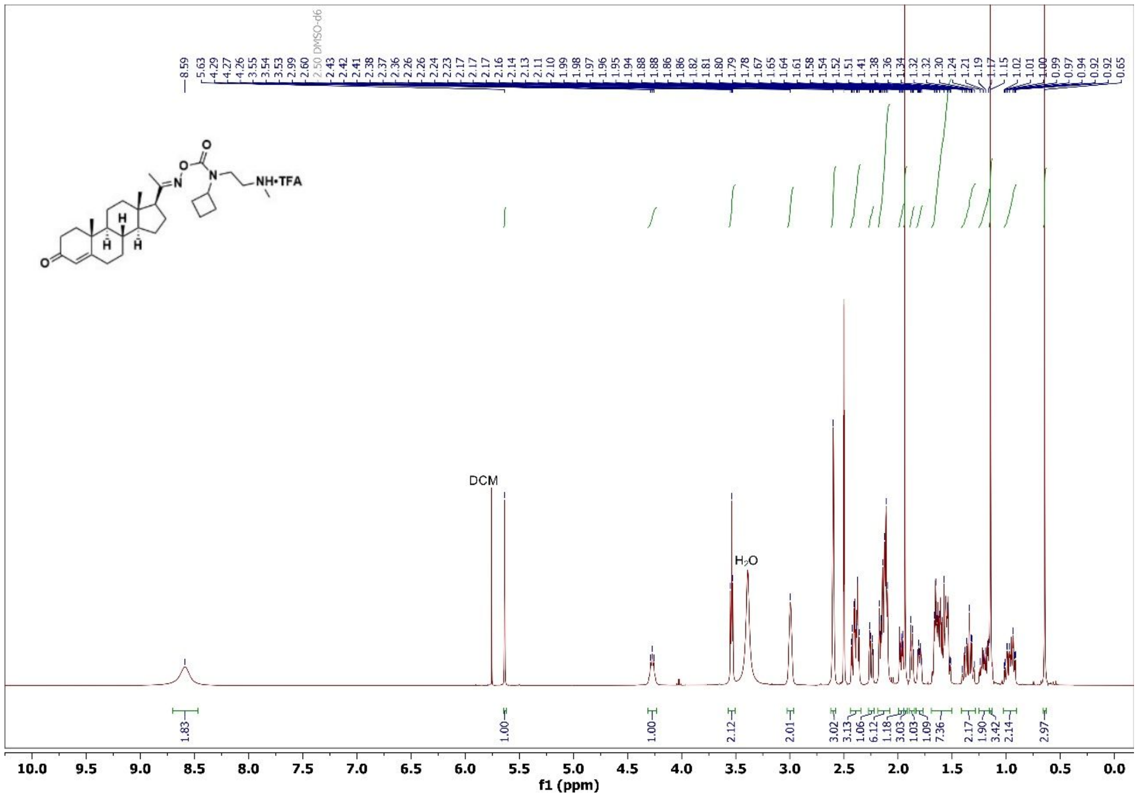

^{1}H NMR of Compound **20d** (600 MHz, DMSO-d_6)

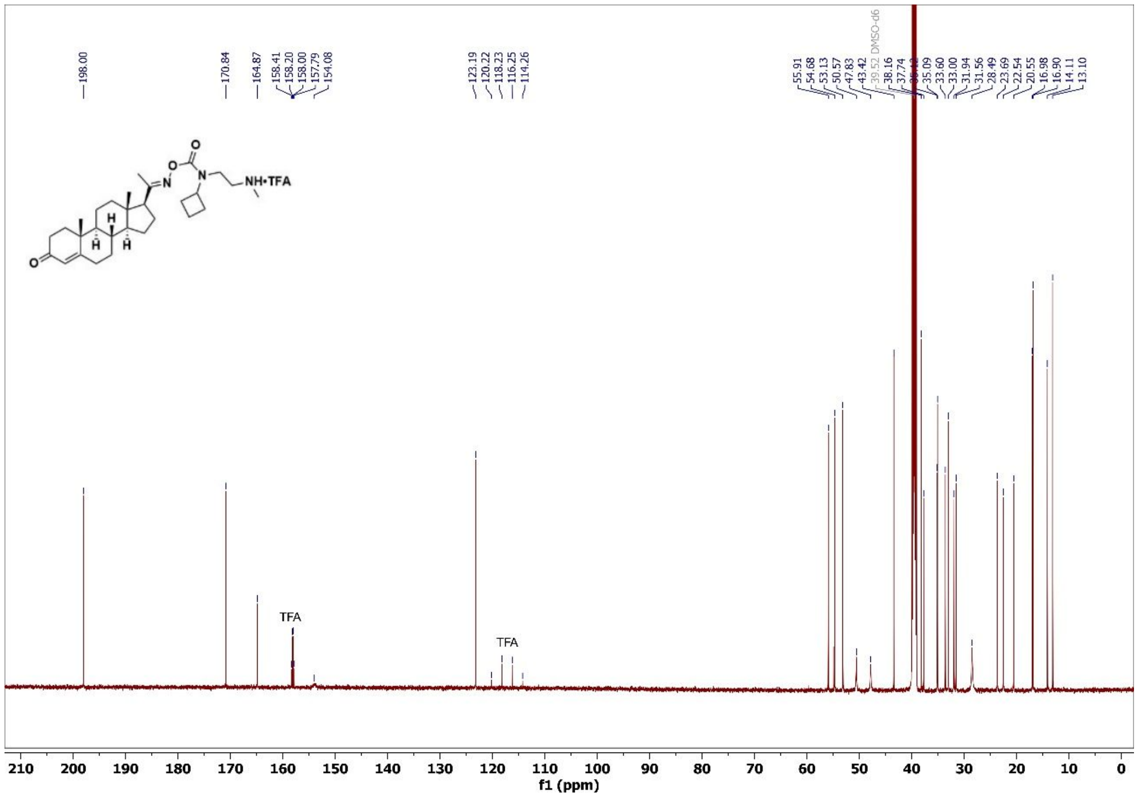

^{13}C NMR of Compound **20d** (151 MHz, DMSO-d_6)

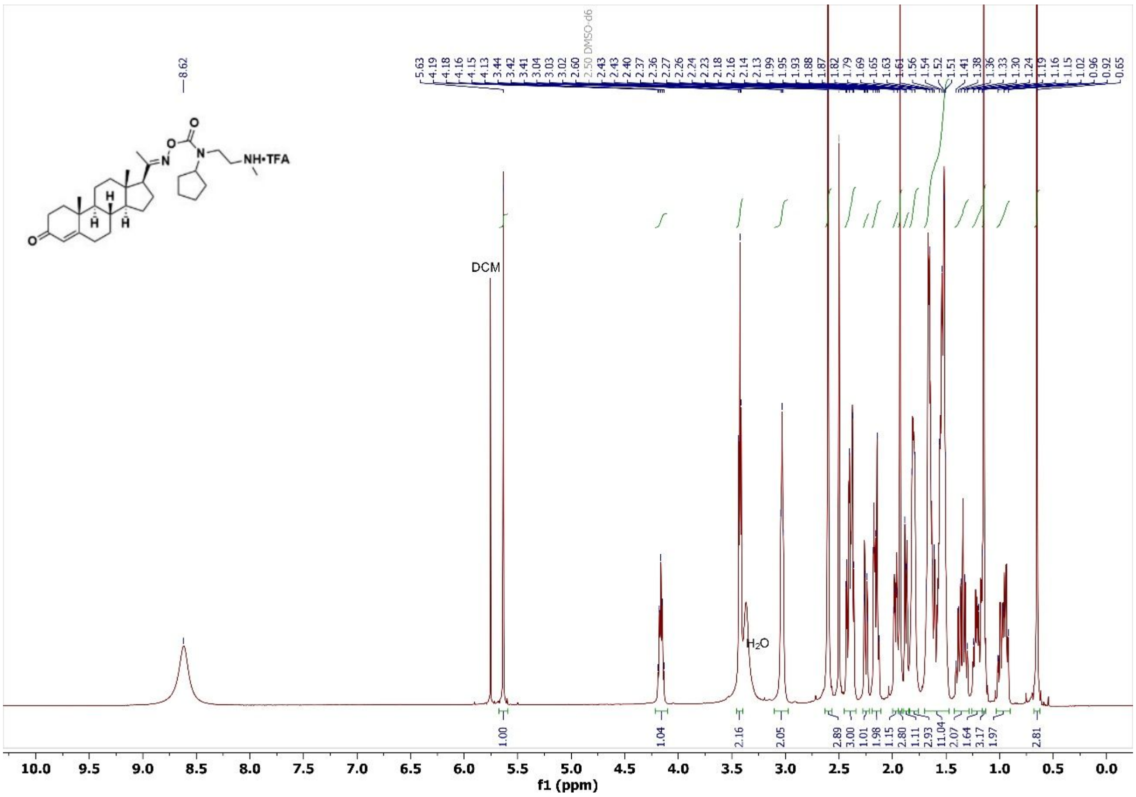

^1H NMR of Compound **20e** (600 MHz, DMSO-d_6)

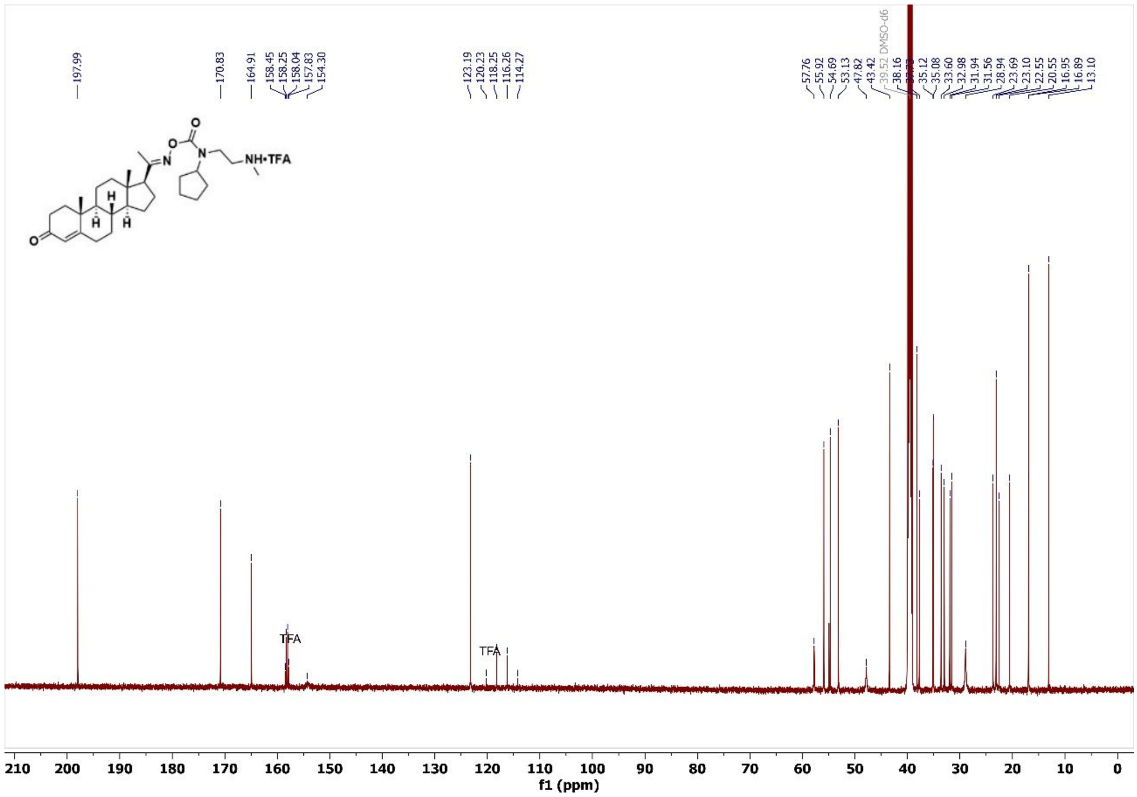

^{13}C NMR of Compound **20e** (151 MHz, DMSO-d_6)

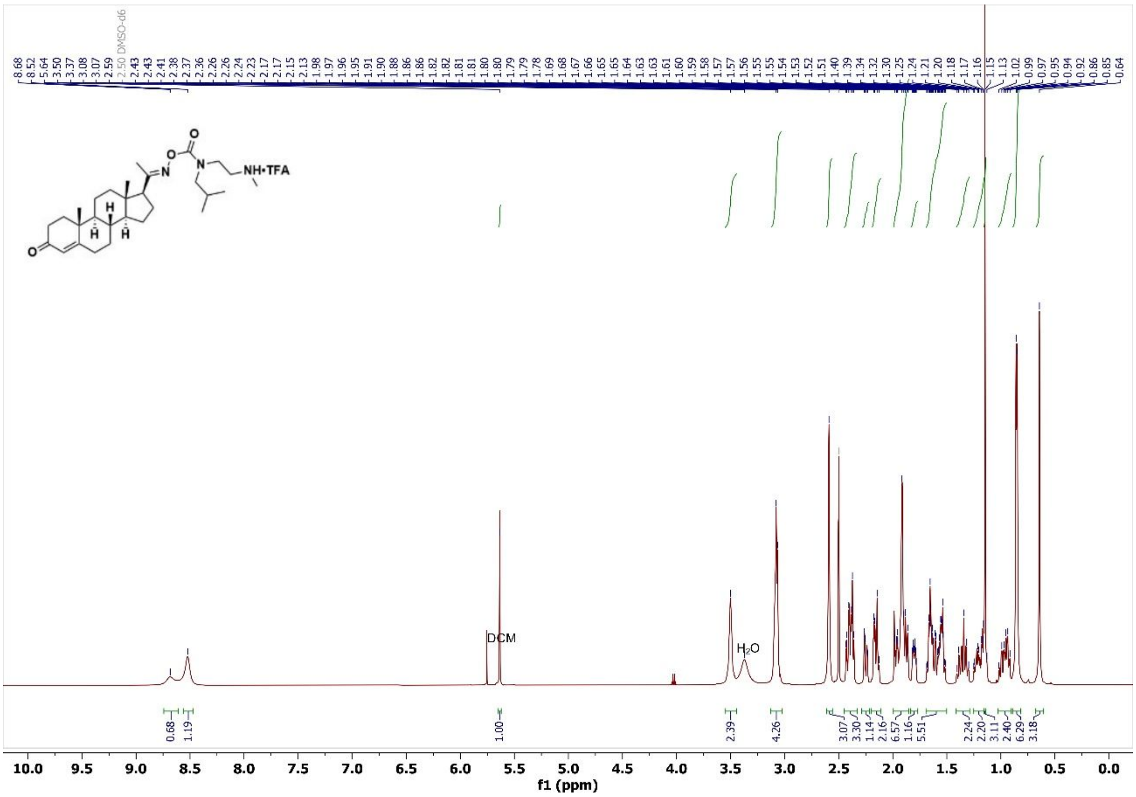

^{1}H NMR of Compound **20f** (600 MHz, DMSO-d_6)

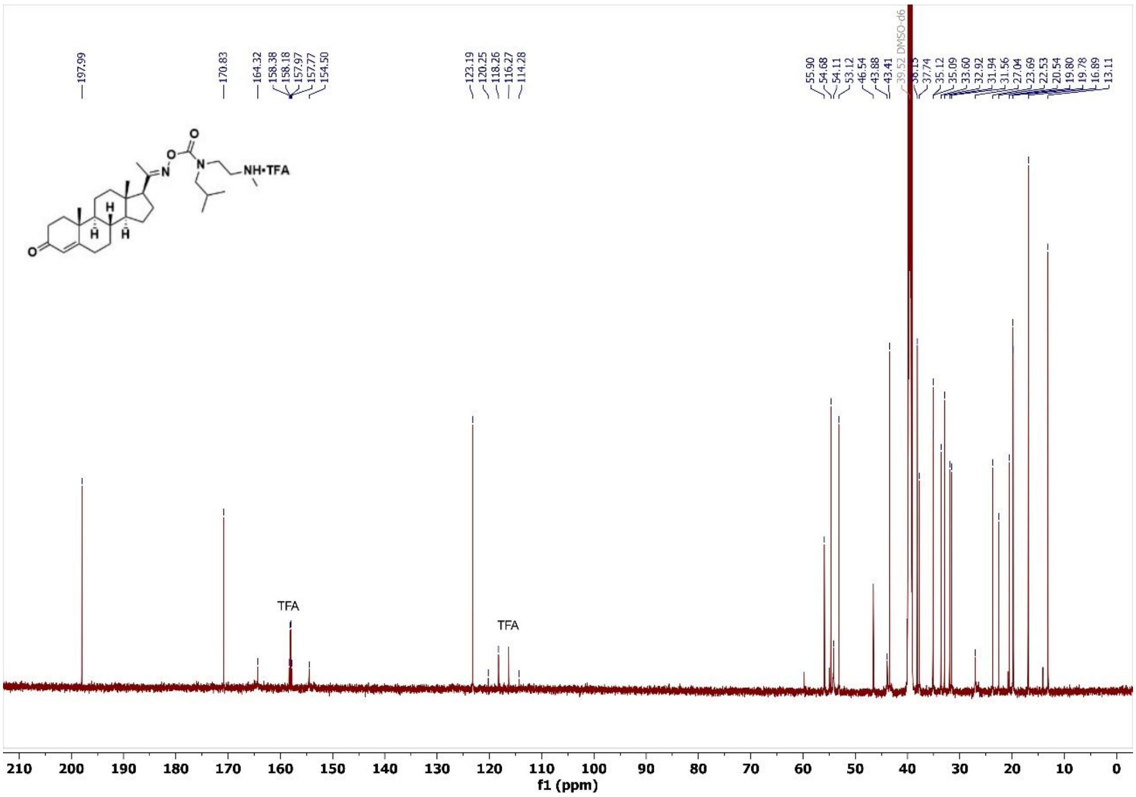

^{13}C NMR of Compound **20f** (151 MHz, DMSO-d_6)

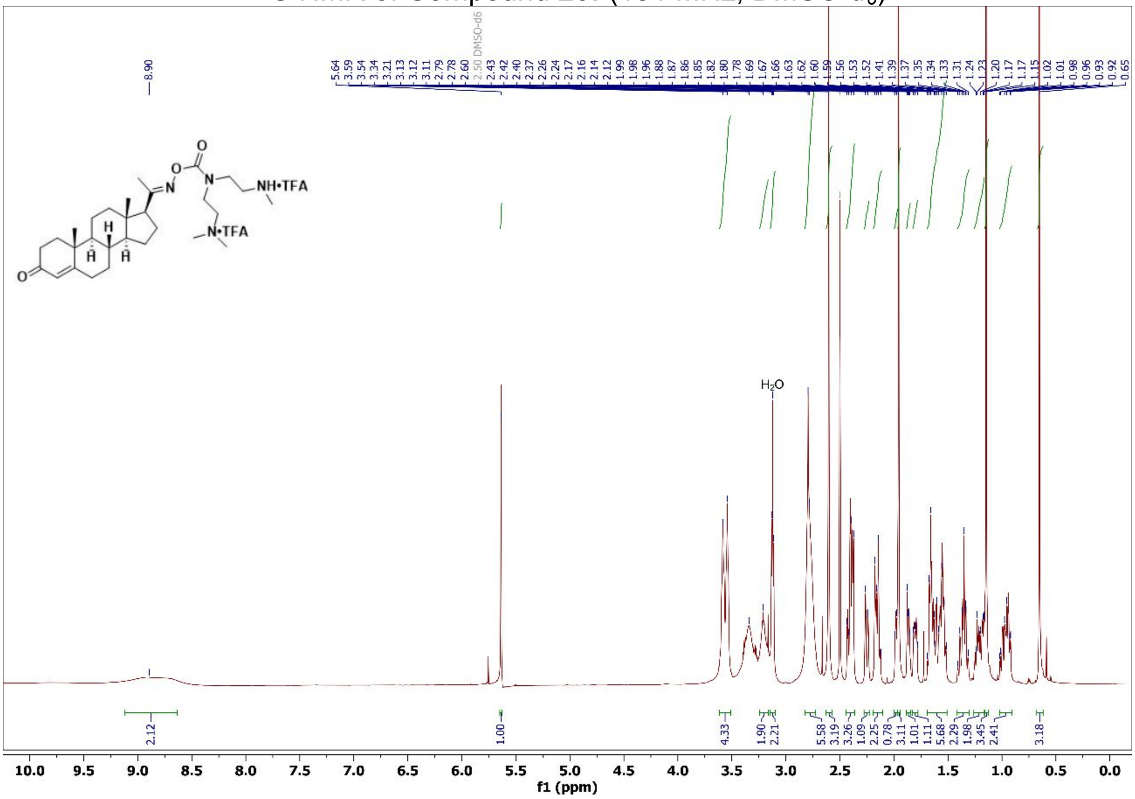

^{1}H NMR of Compound **20g** (600 MHz, DMSO-d_6)

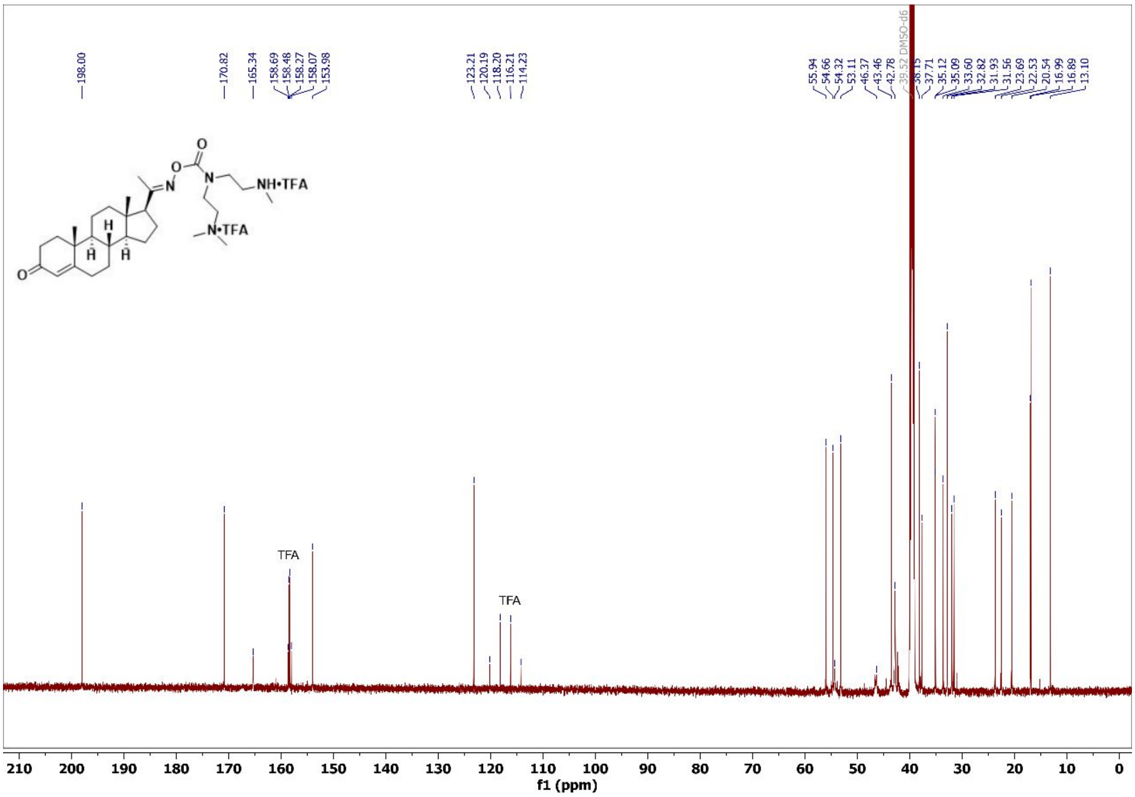

^{13}C NMR of Compound **20g** (151 MHz, DMSO-d_6)

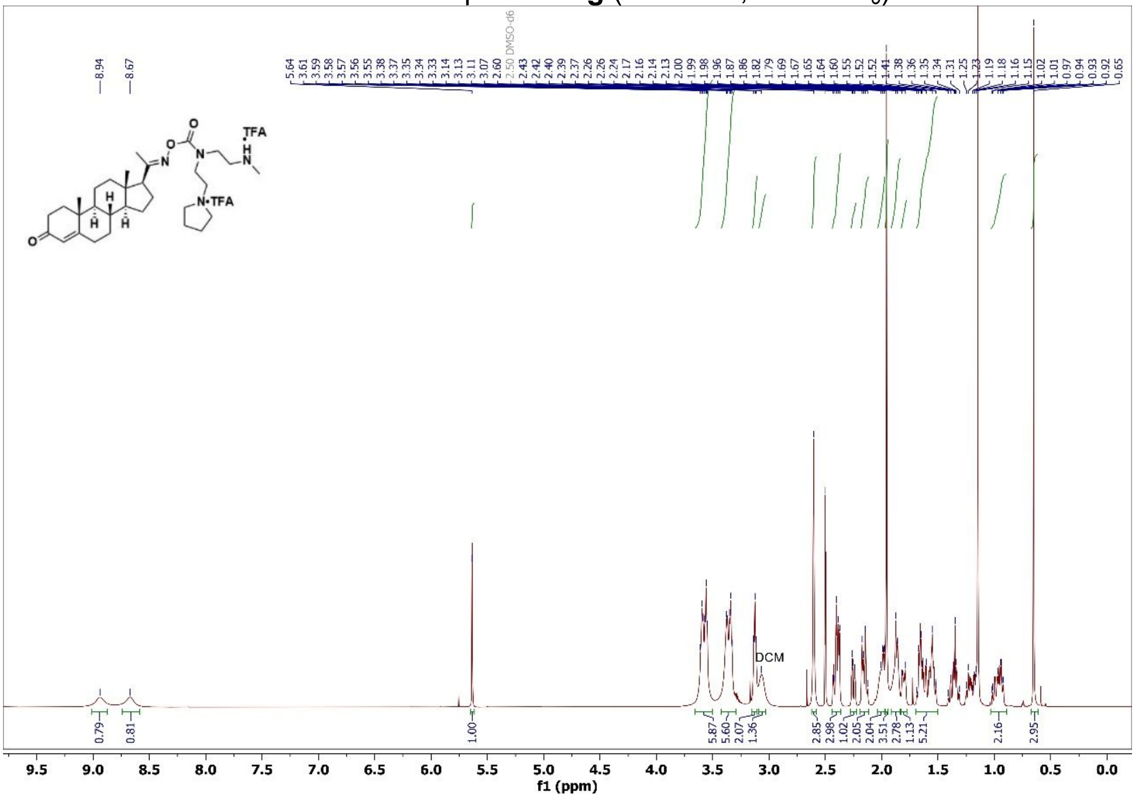

^{1}H NMR of Compound **20h** (600 MHz, DMSO-d_6)

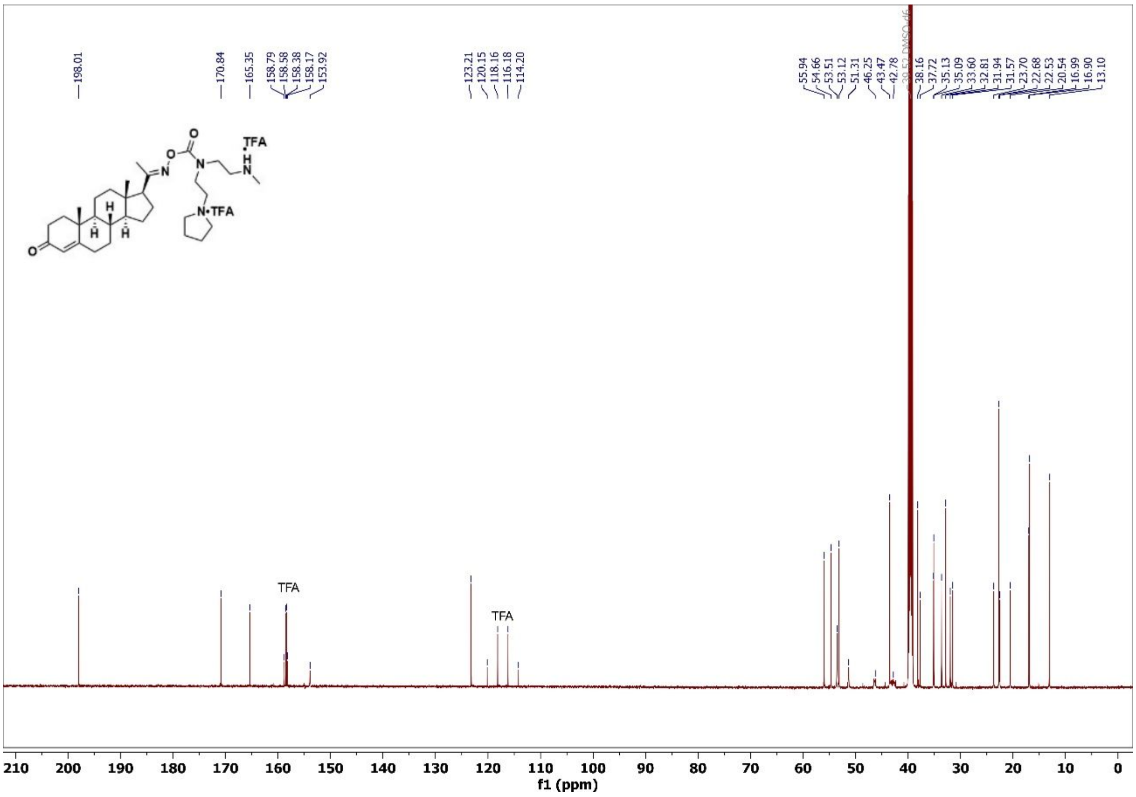

^{13}C NMR of Compound **20h** (151 MHz, DMSO-d_6)

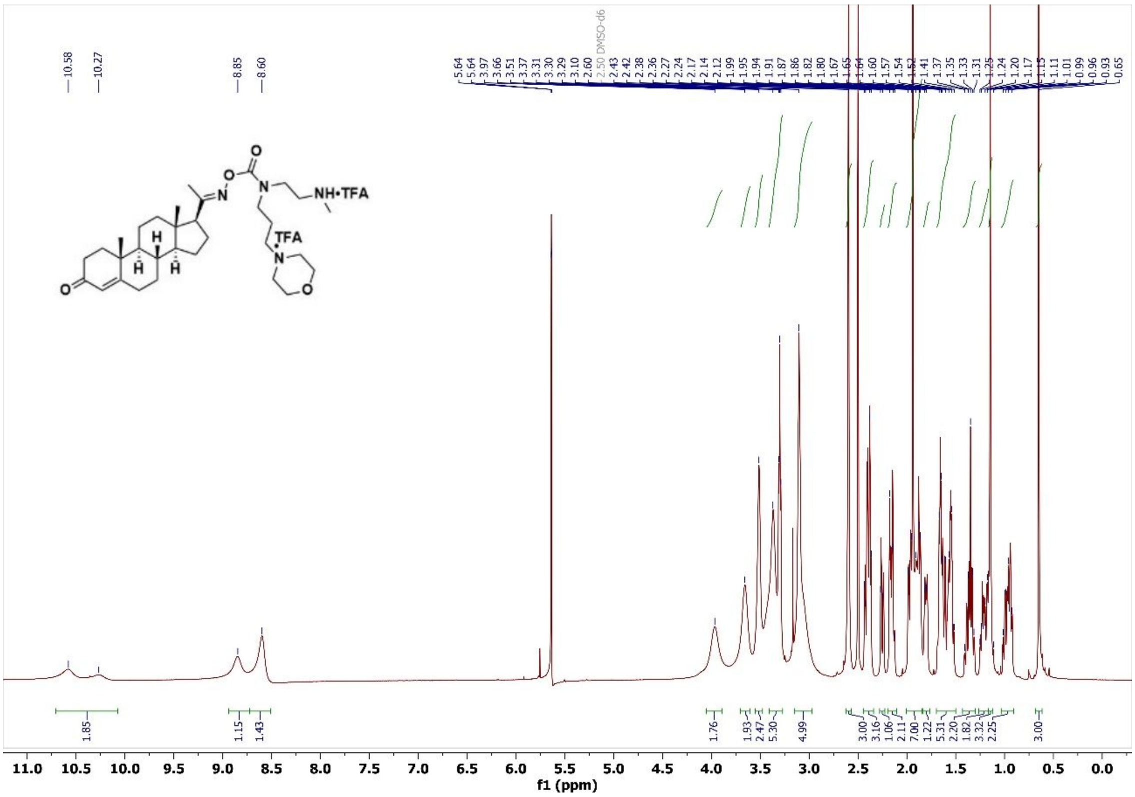

^{1}H NMR of Compound **20i** (600 MHz, DMSO-d_6)

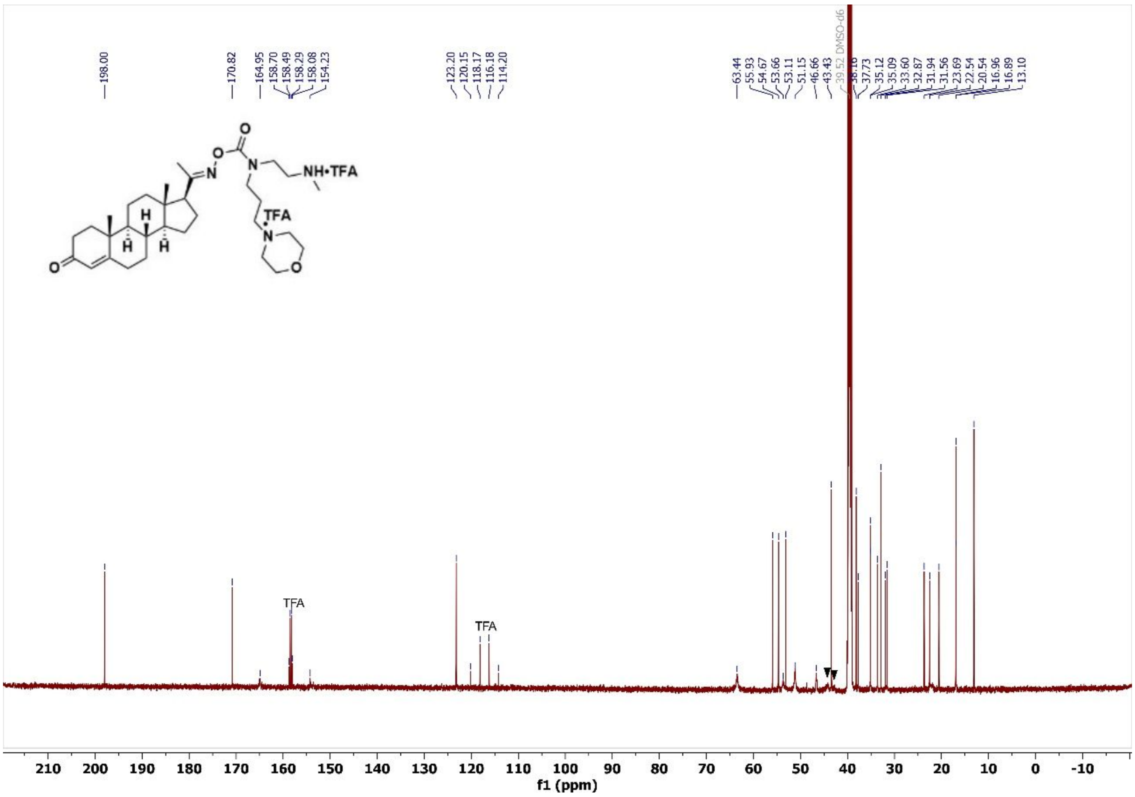

^{13}C NMR of Compound **20i** (151 MHz, DMSO-d_6)

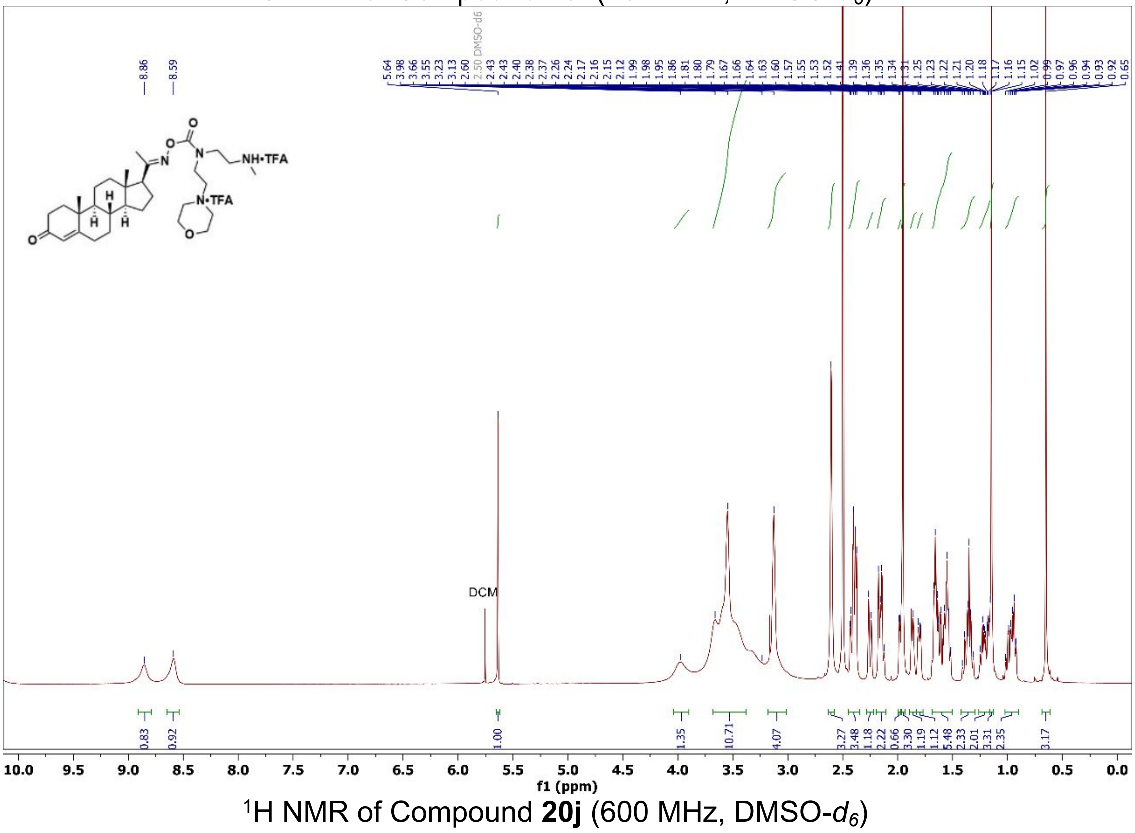

^{1}H NMR of Compound **20j** (600 MHz, DMSO-d_6)

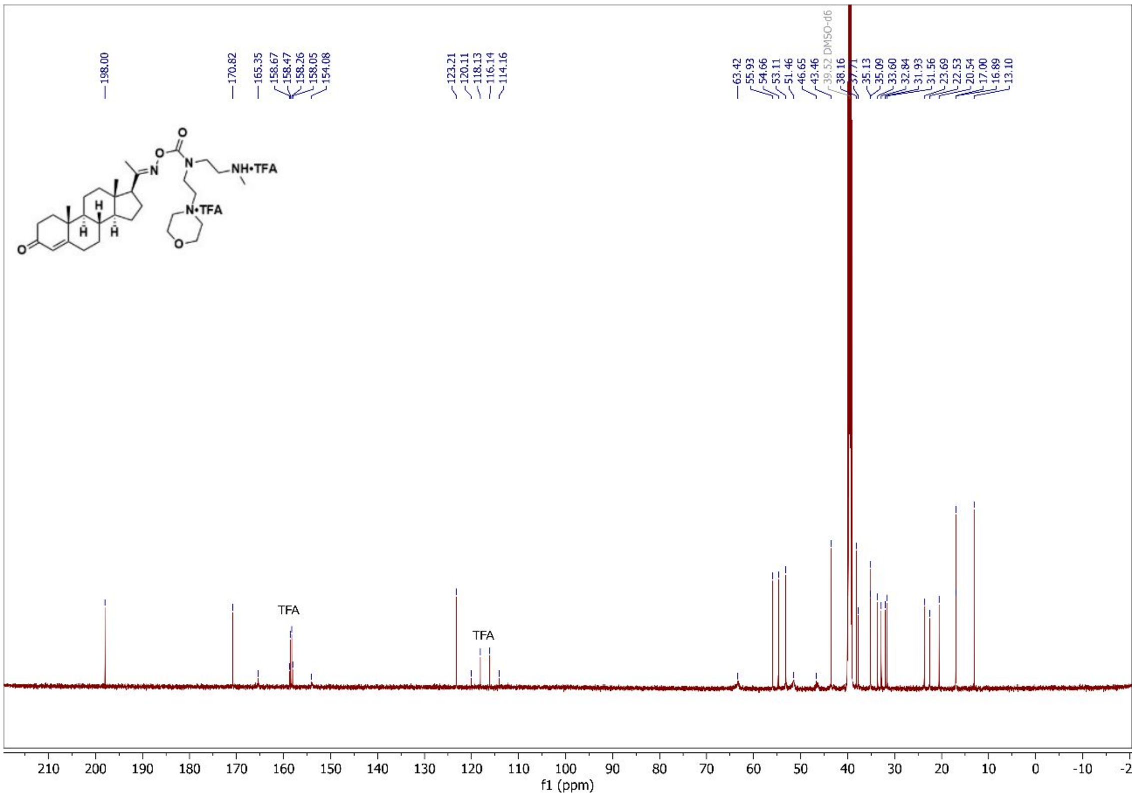

^{13}C NMR of Compound **20j** (151 MHz, DMSO-d_6)

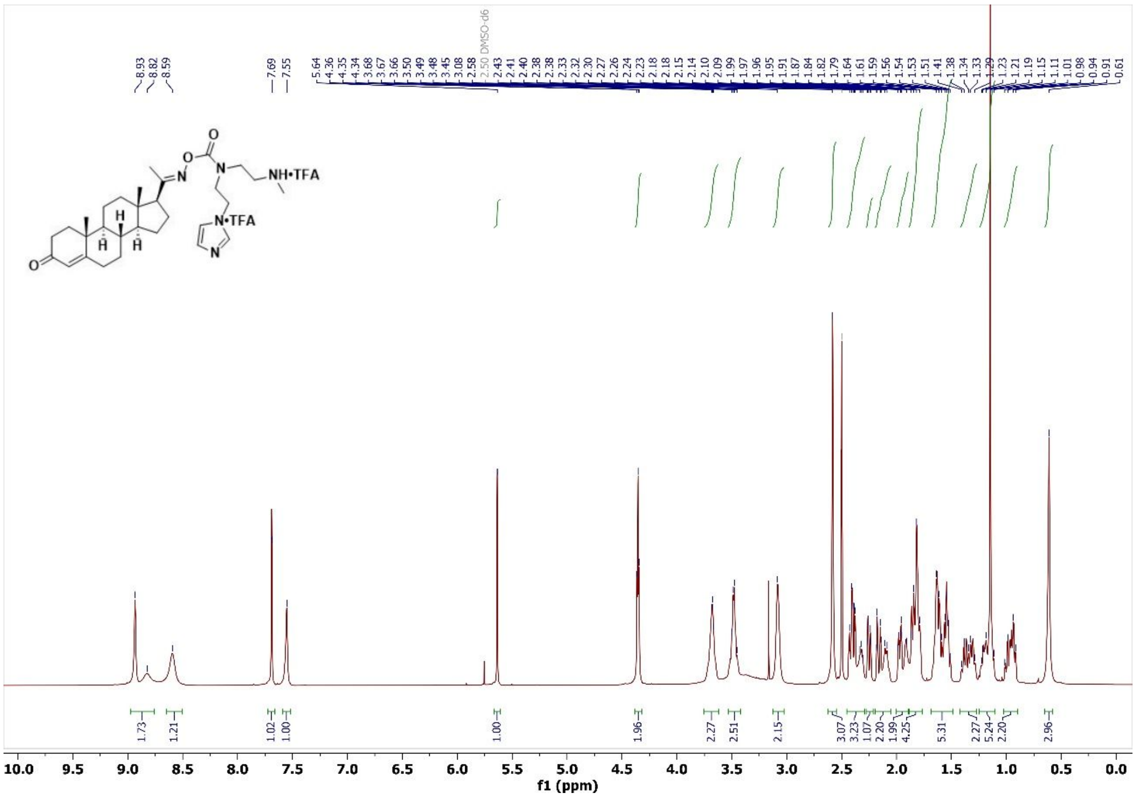

^{1}H NMR of Compound **20k** (600 MHz, DMSO-d_6)

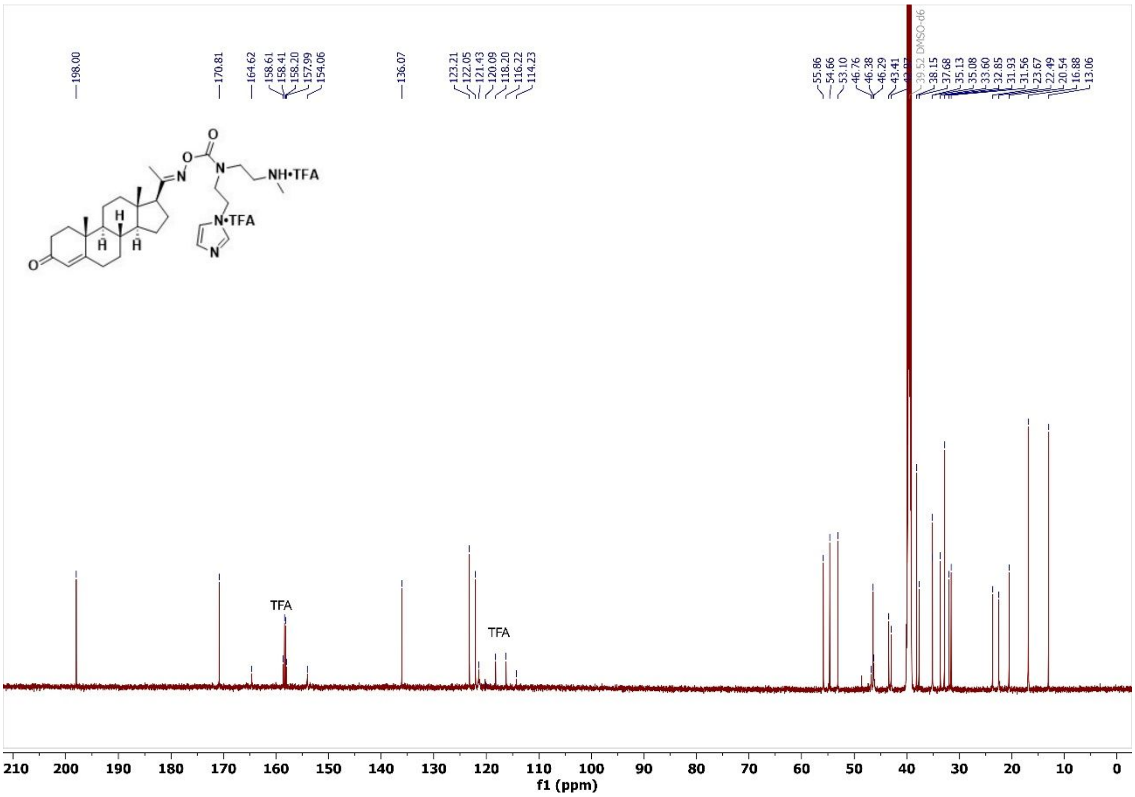

^{13}C NMR of Compound **20k** (151 MHz, DMSO-d_6)

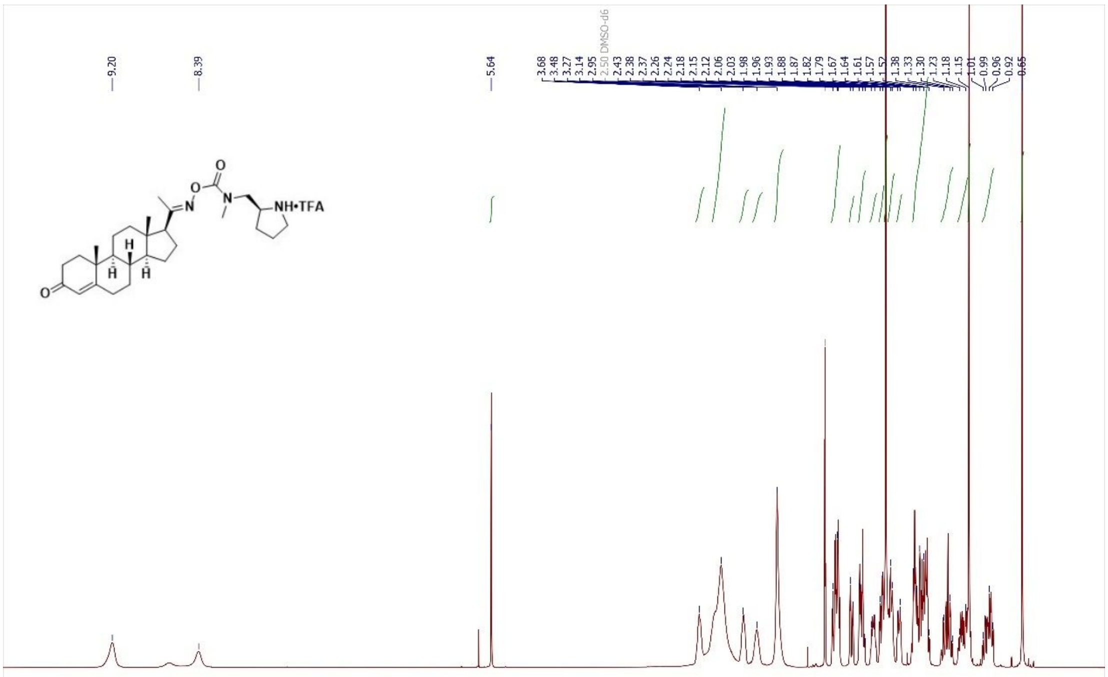

^{1}H NMR of Compound **20l** (600 MHz, DMSO-d_6)

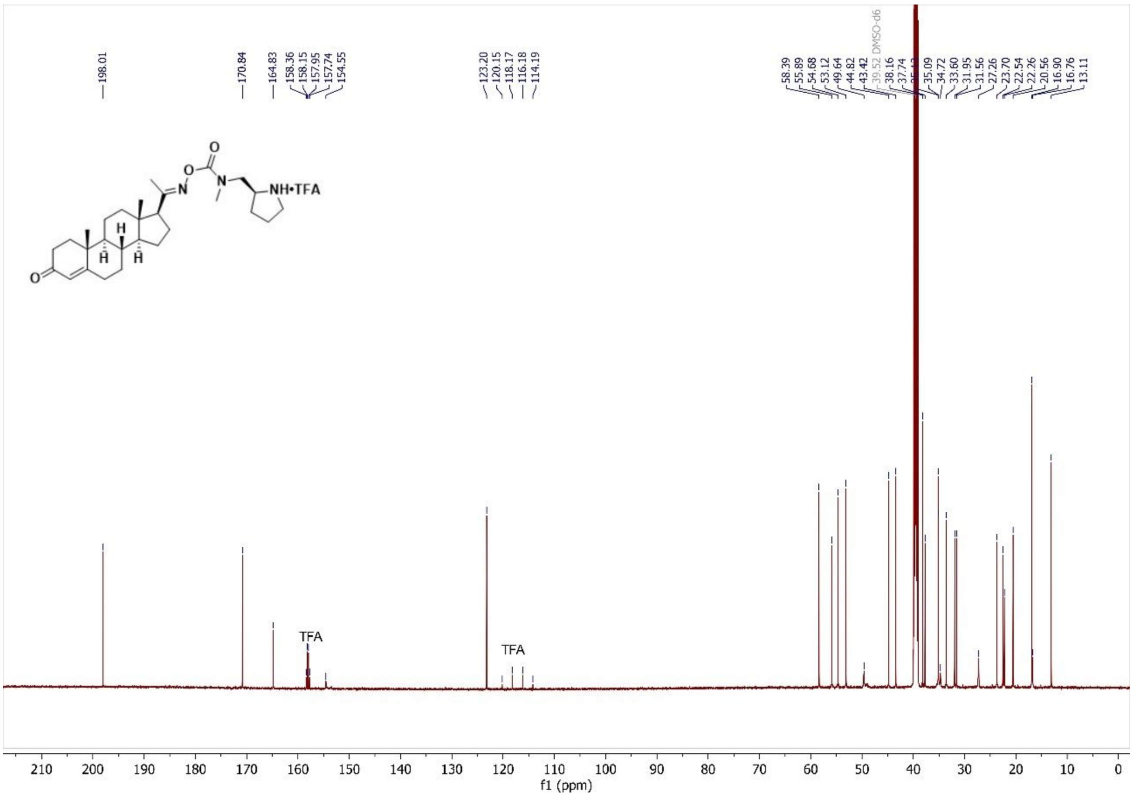

^{13}C NMR of Compound **20l** (151 MHz, DMSO-d_6)

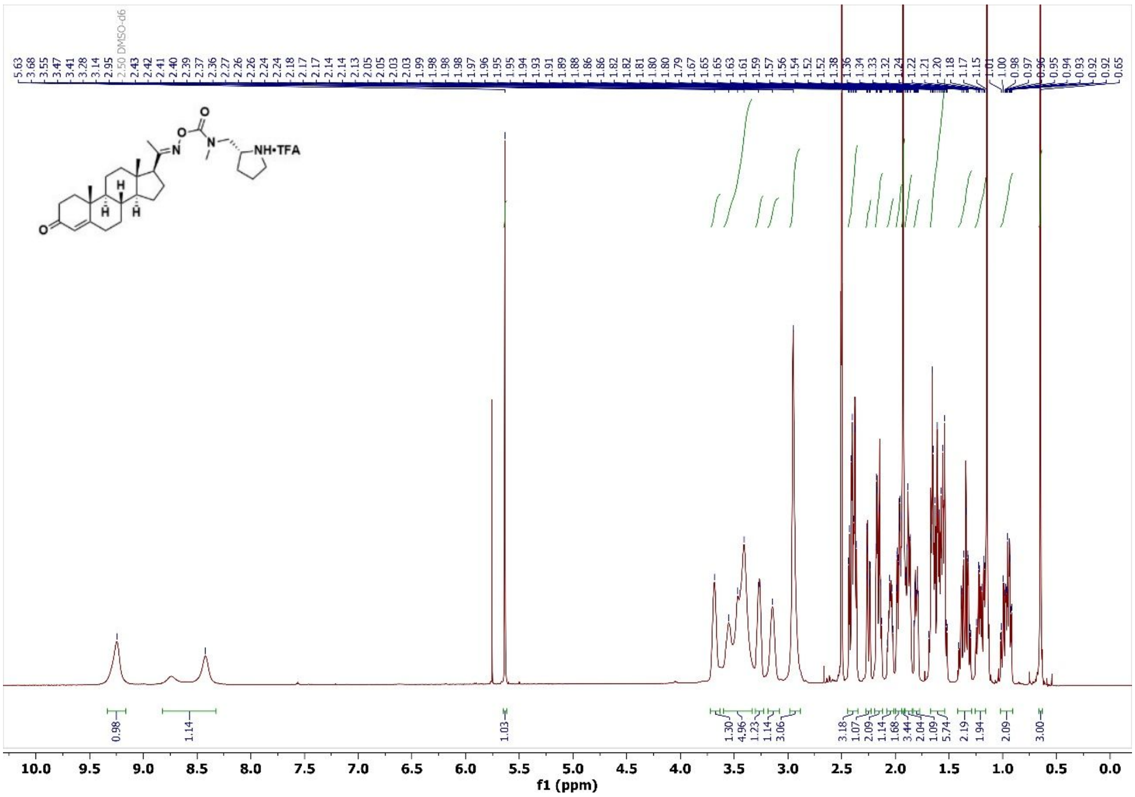

^{1}H NMR of Compound **20m** (600 MHz, DMSO-d_6)

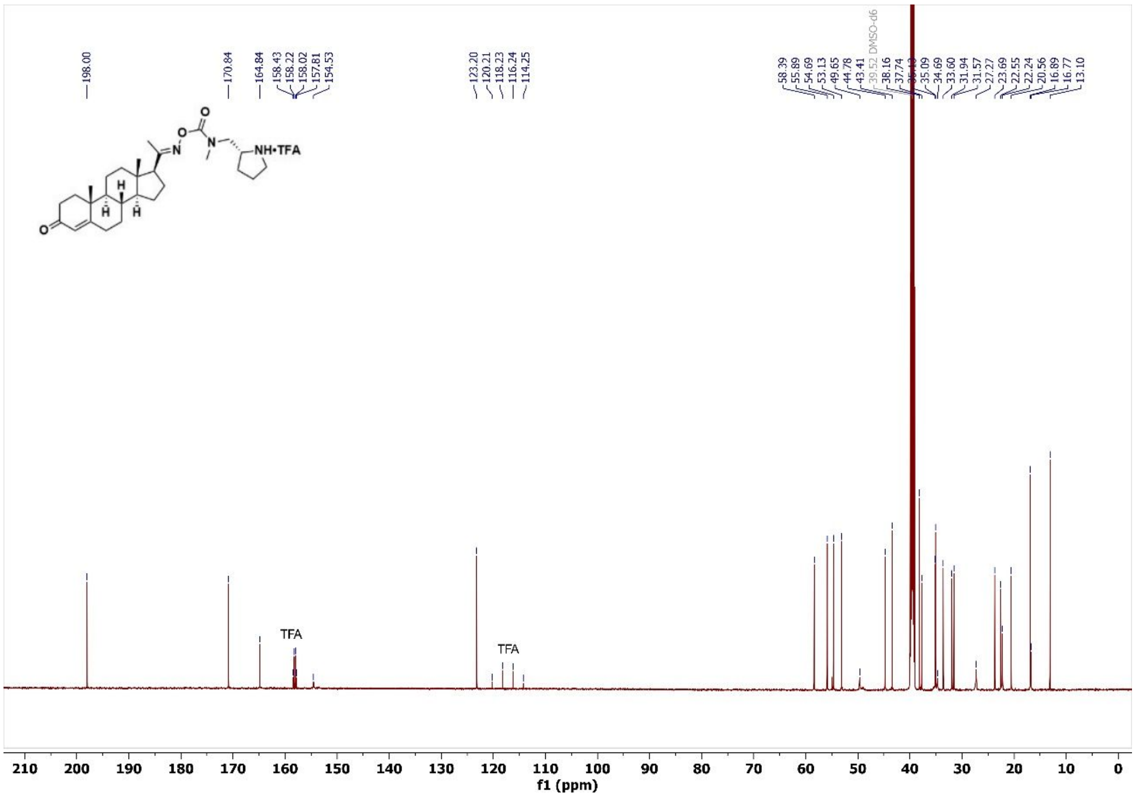

^{13}C NMR of Compound **20m** (151 MHz, DMSO-d_6)

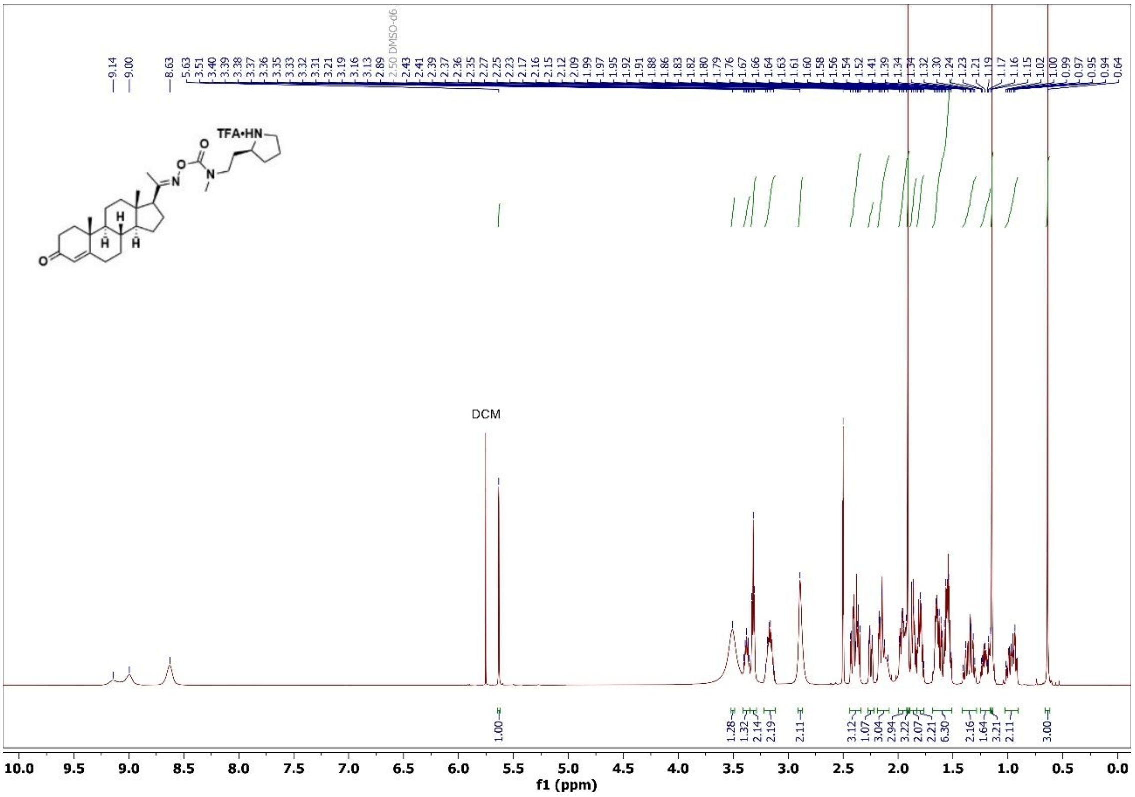

^{1}H NMR of Compound **20n** (600 MHz, DMSO-d_6)

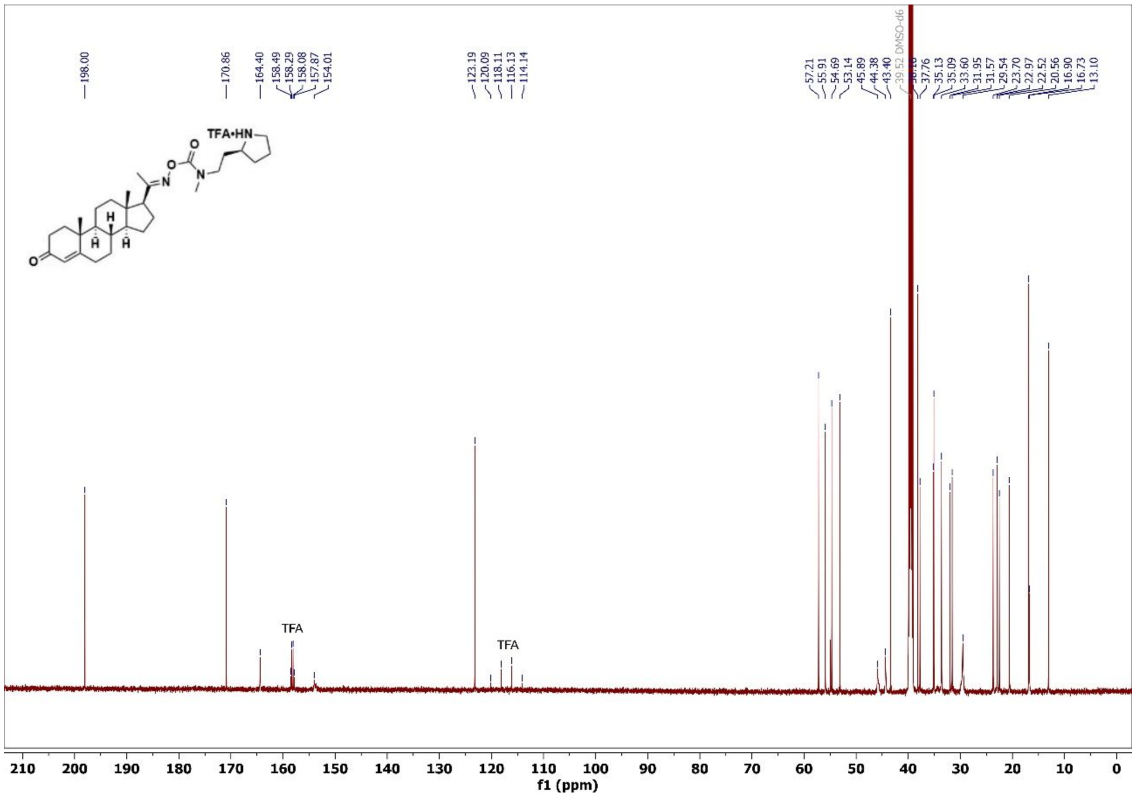

^{13}C NMR of Compound **20n** (151 MHz, DMSO-d_6)

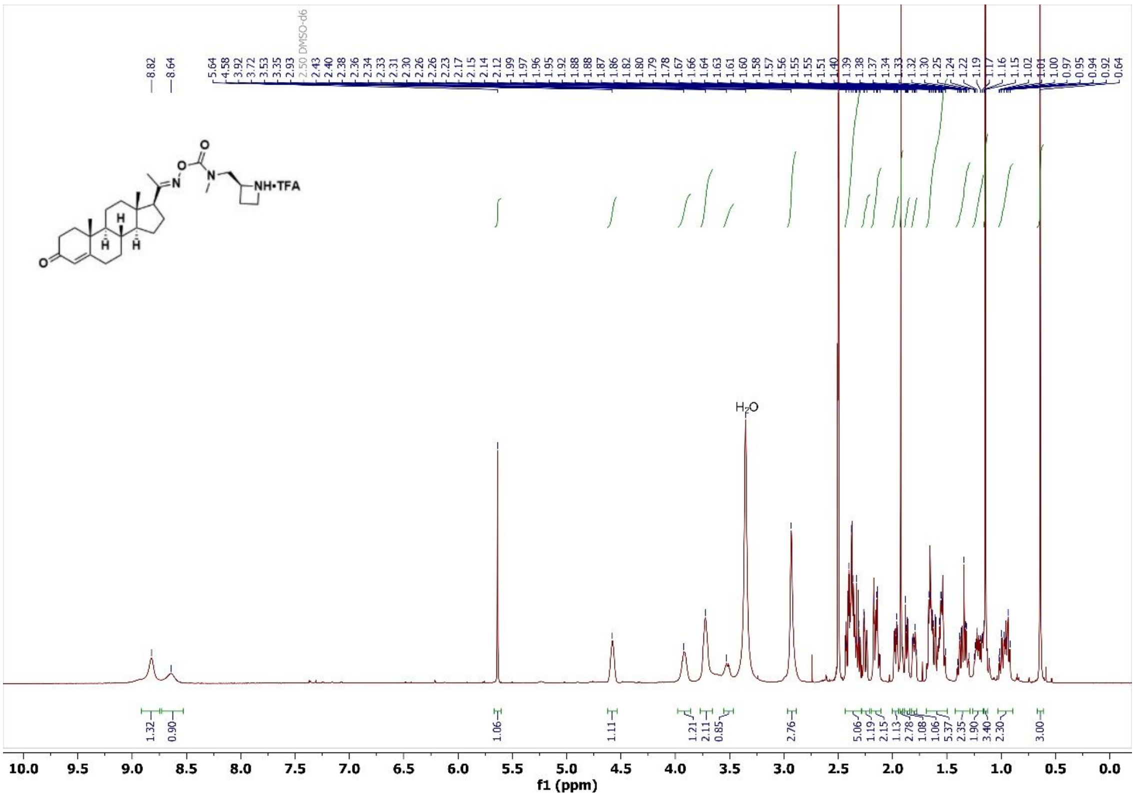

^{1}H NMR of Compound **20o** (600 MHz, DMSO-d_6)

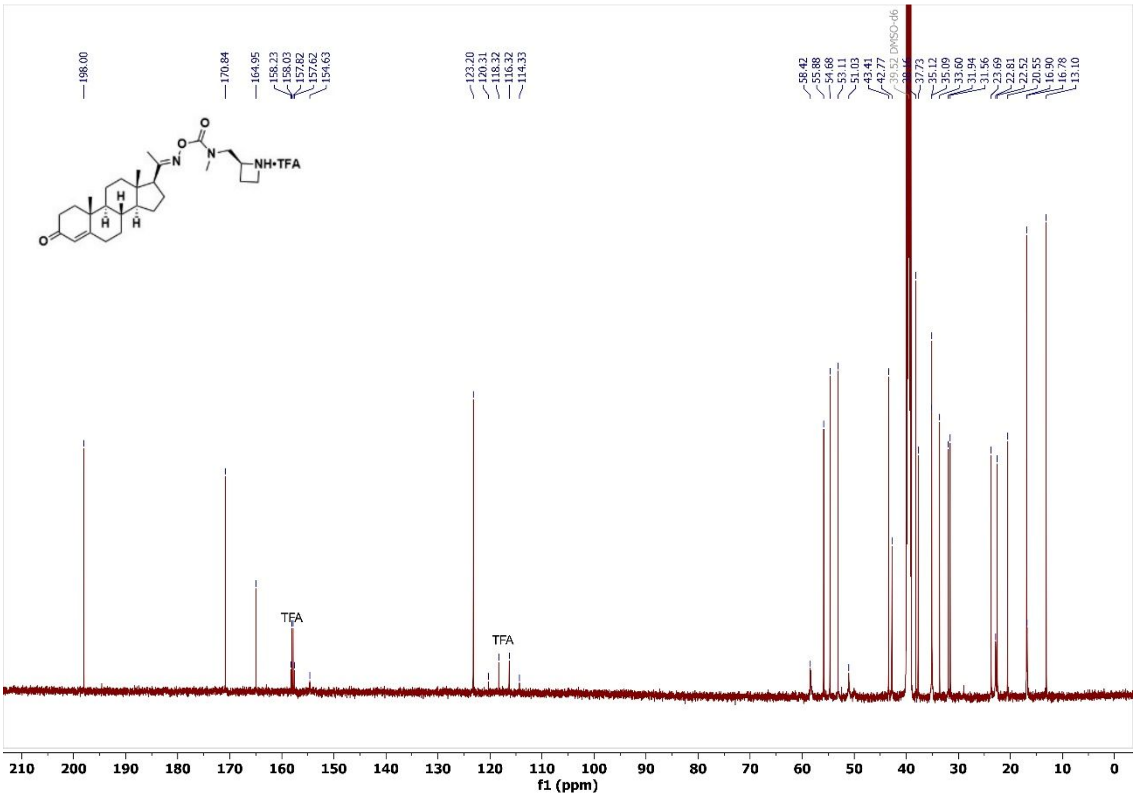

^{13}C NMR of Compound **20o** (151 MHz, DMSO-d_6)

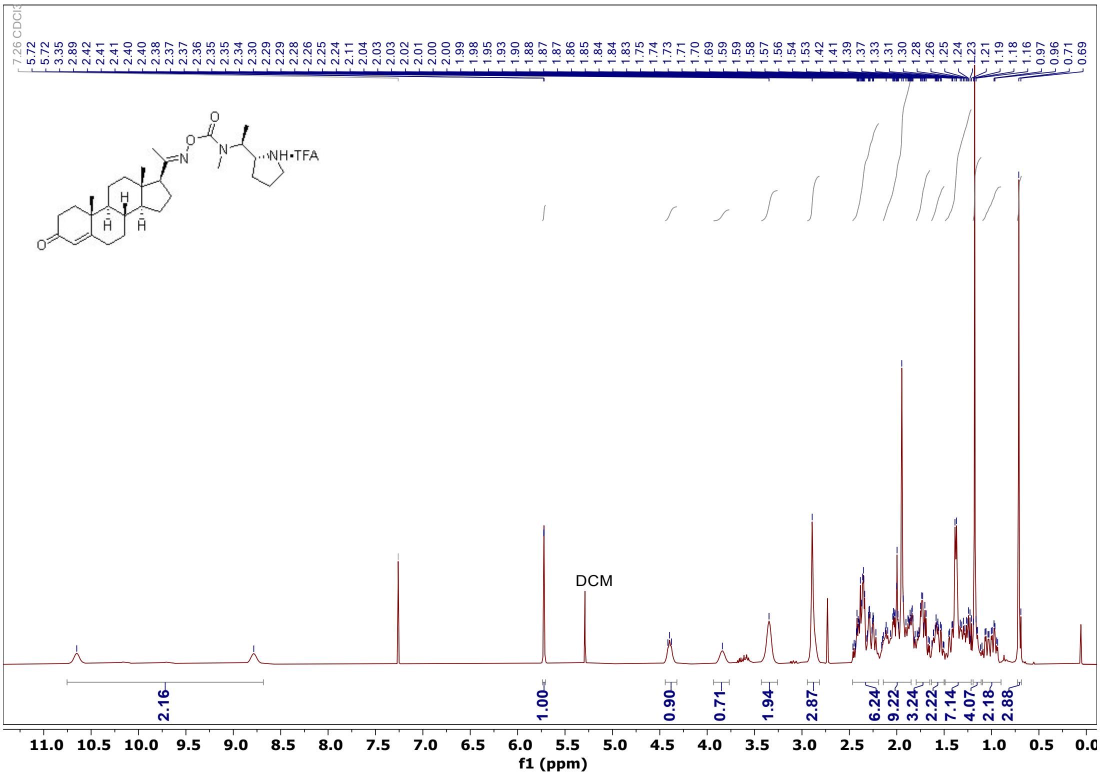

^{1}H NMR of Compound **20p** (400 MHz, $CDCl_3$)

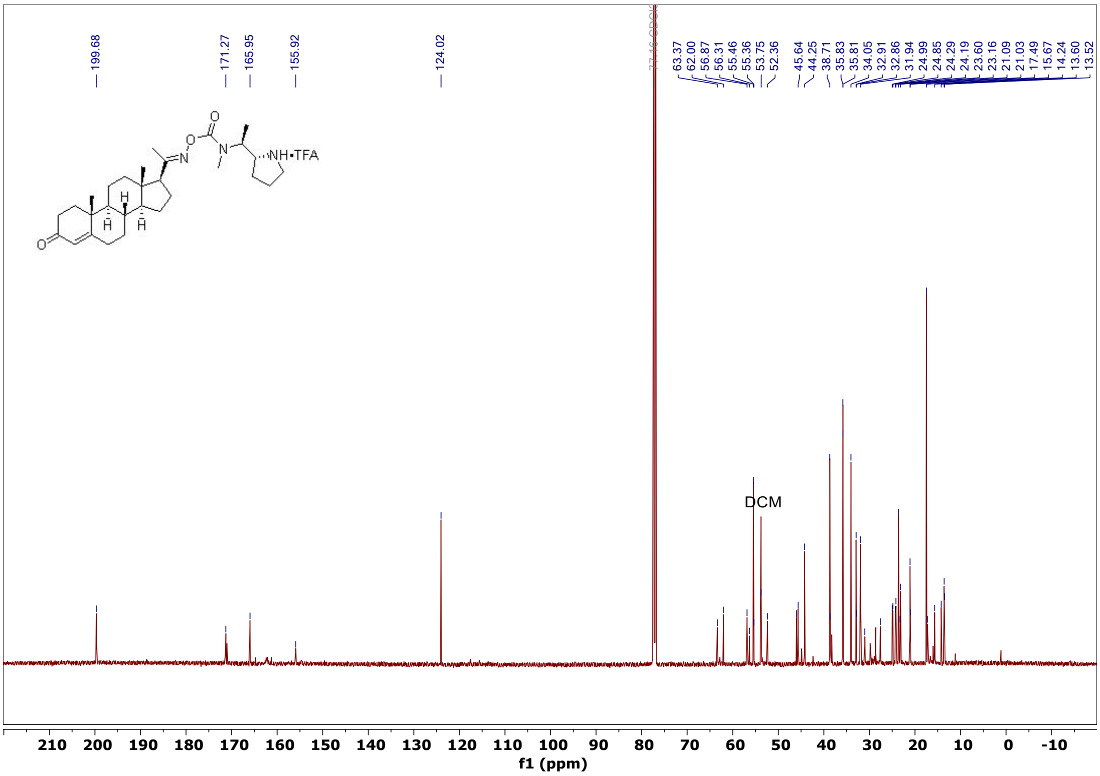

^{13}C NMR of Compound **20p** (151 MHz, $CDCl_3$)

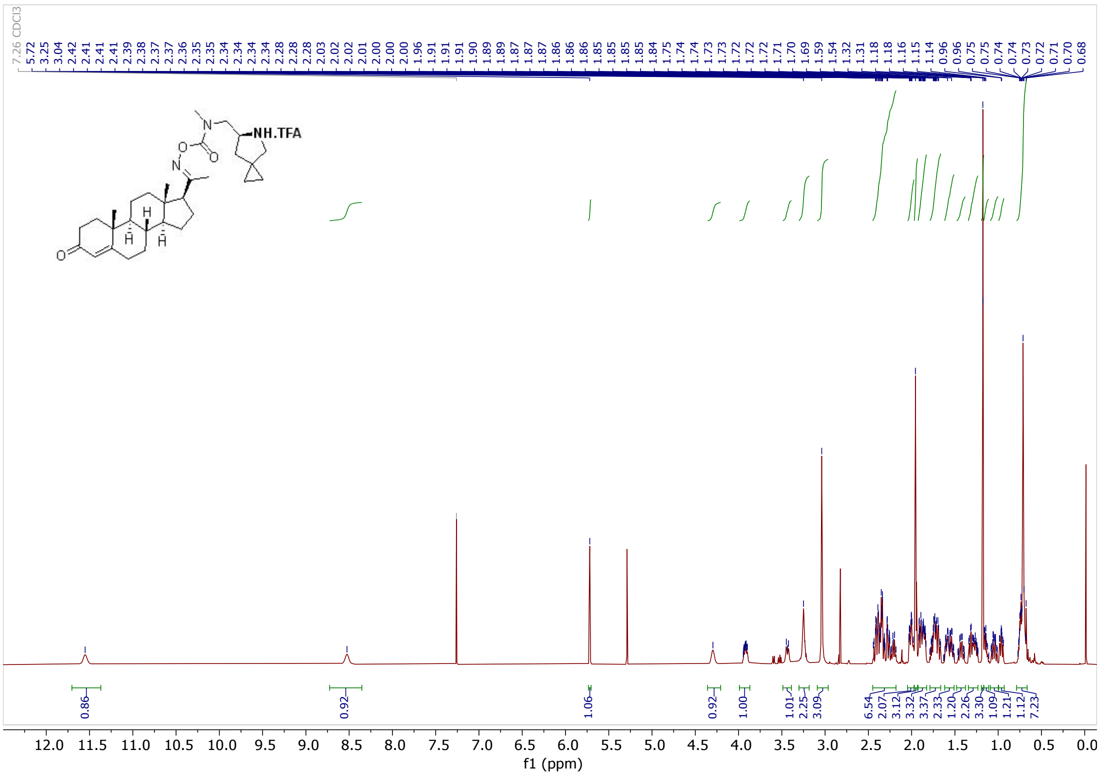

^{1}H NMR of Compound **20q** (00 MHz, $CDCl_3$)

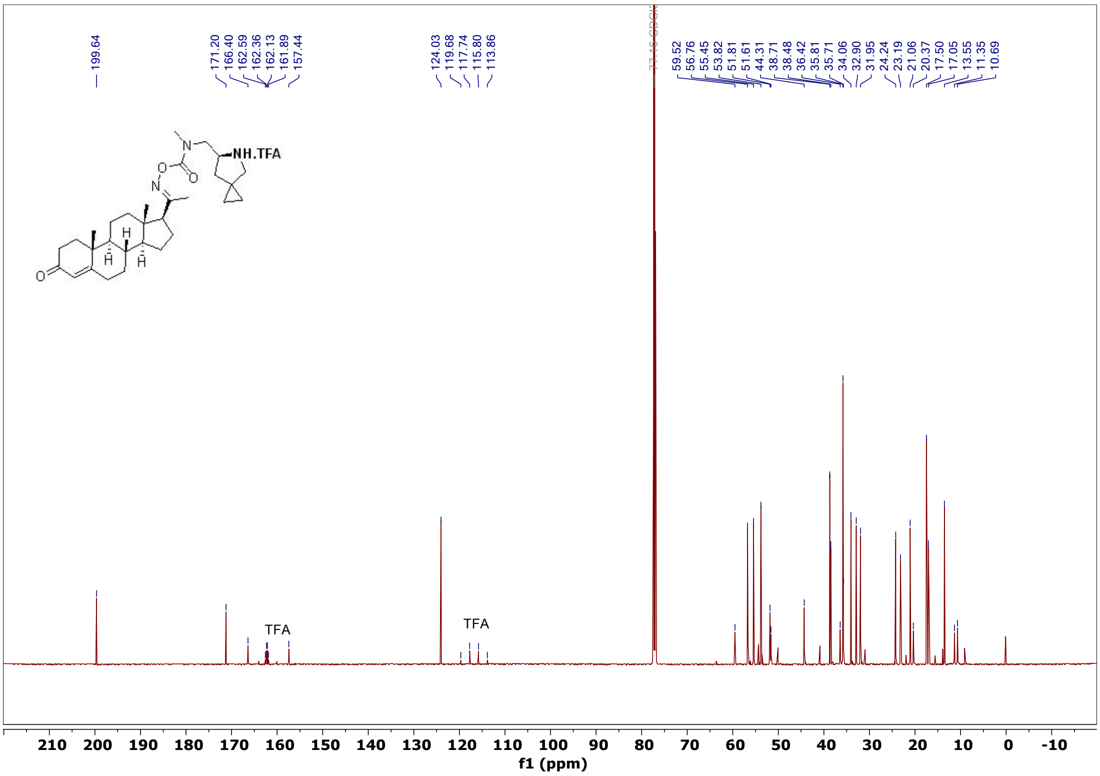

^{13}C NMR of Compound **20q** (151 MHz, $CDCl_3$)

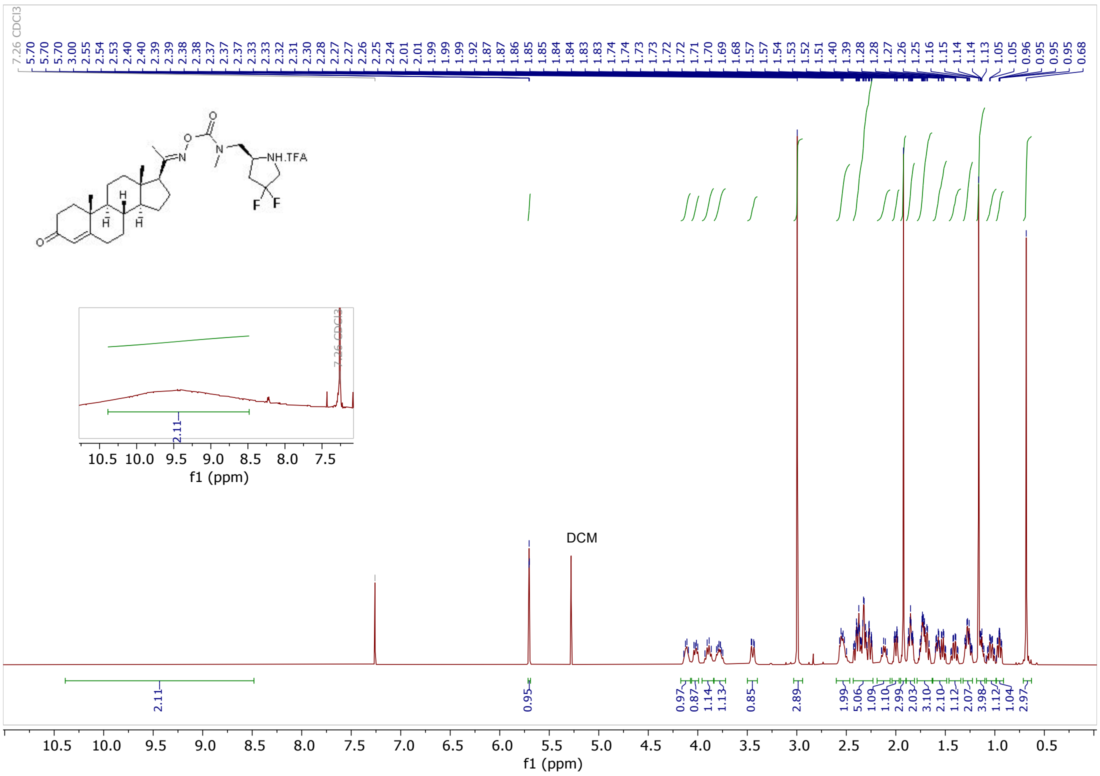

^{1}H NMR of Compound **20r** (600 MHz, $CDCl_3$)

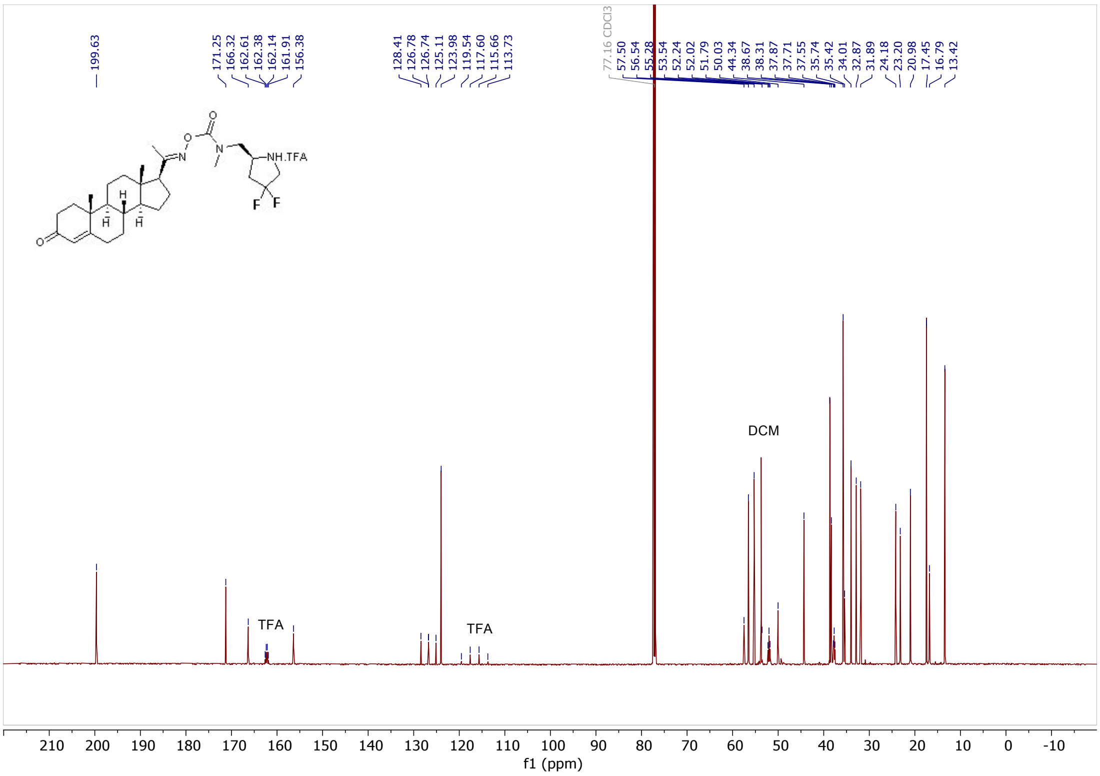

^{13}C NMR of Compound **20r** (151 MHz, $CDCl_3$)

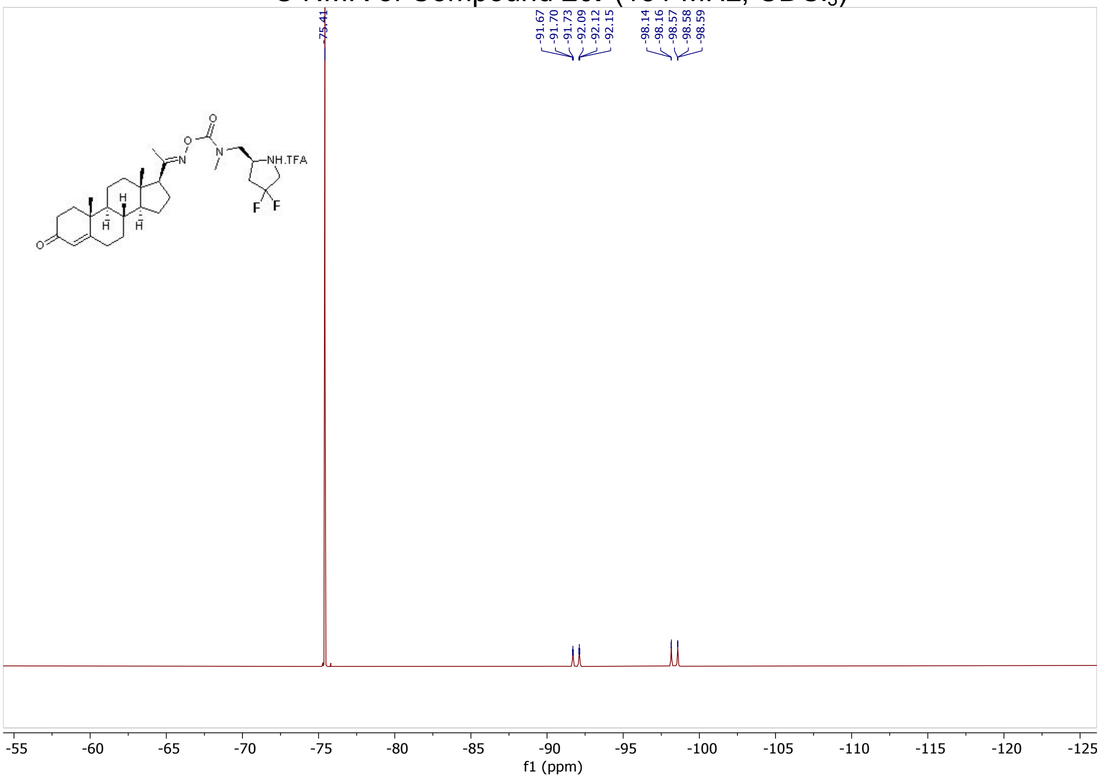

^{19}F NMR of Compound **20r** (151 MHz, $CDCl_3$)

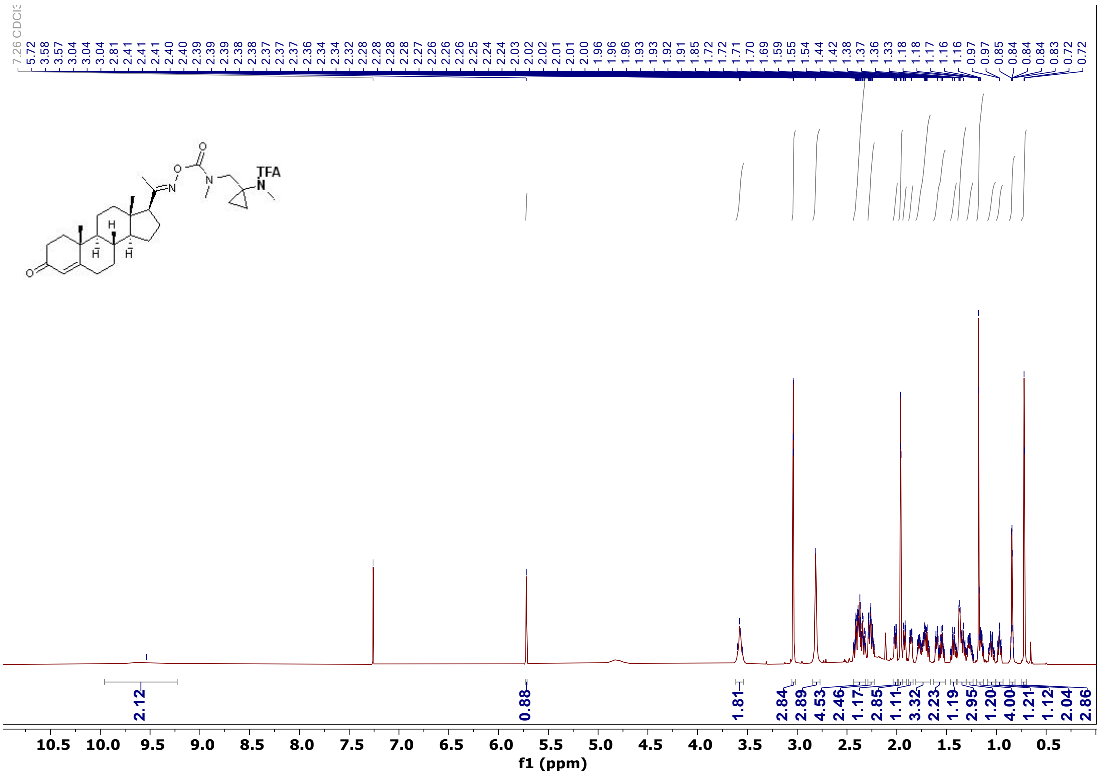

^{1}H NMR of Compound **20s** (600 MHz, $CDCl_3$)

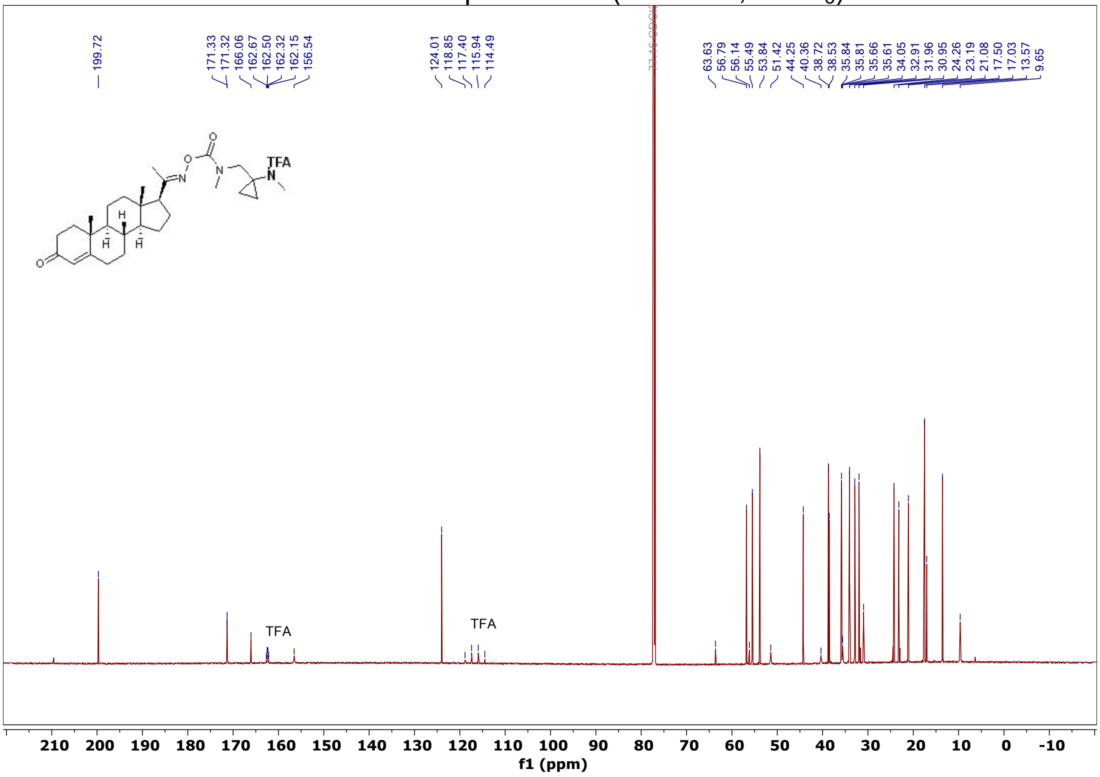

^{13}C NMR of Compound **20s** (151 MHz, $CDCl_3$)

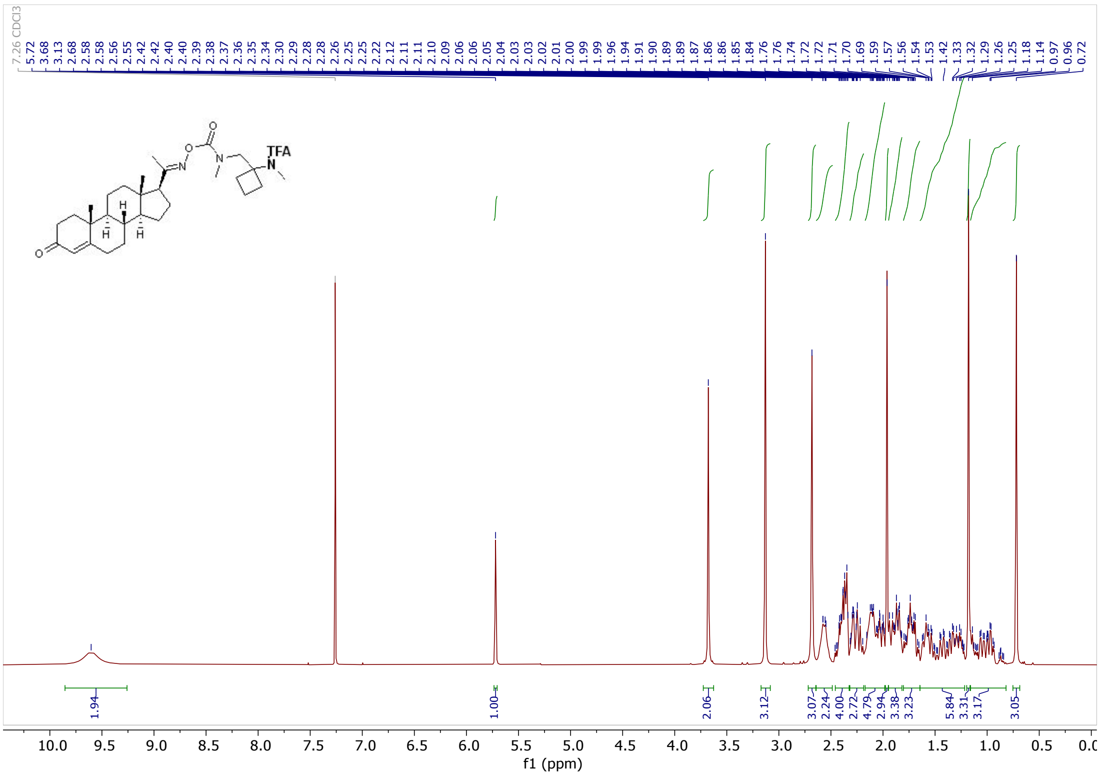

^{1}H NMR of Compound **20t** (600 MHz, $CDCl_3$)

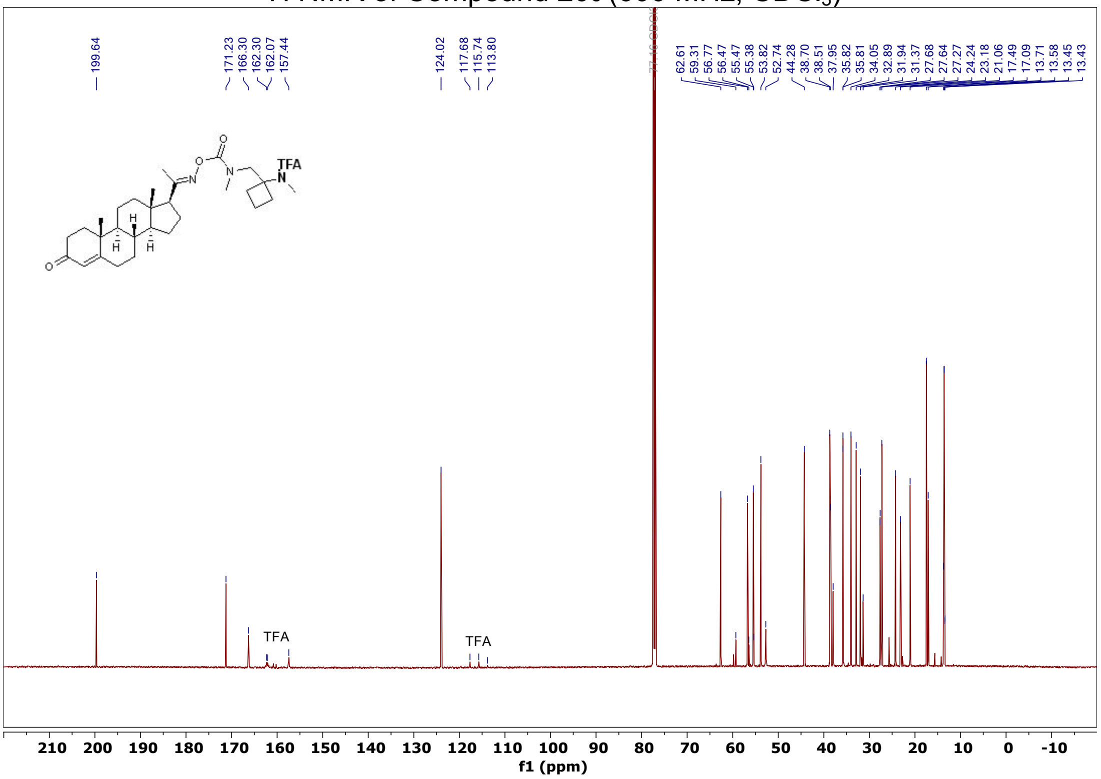

^{13}C NMR of Compound **20t** (151 MHz, $CDCl_3$)

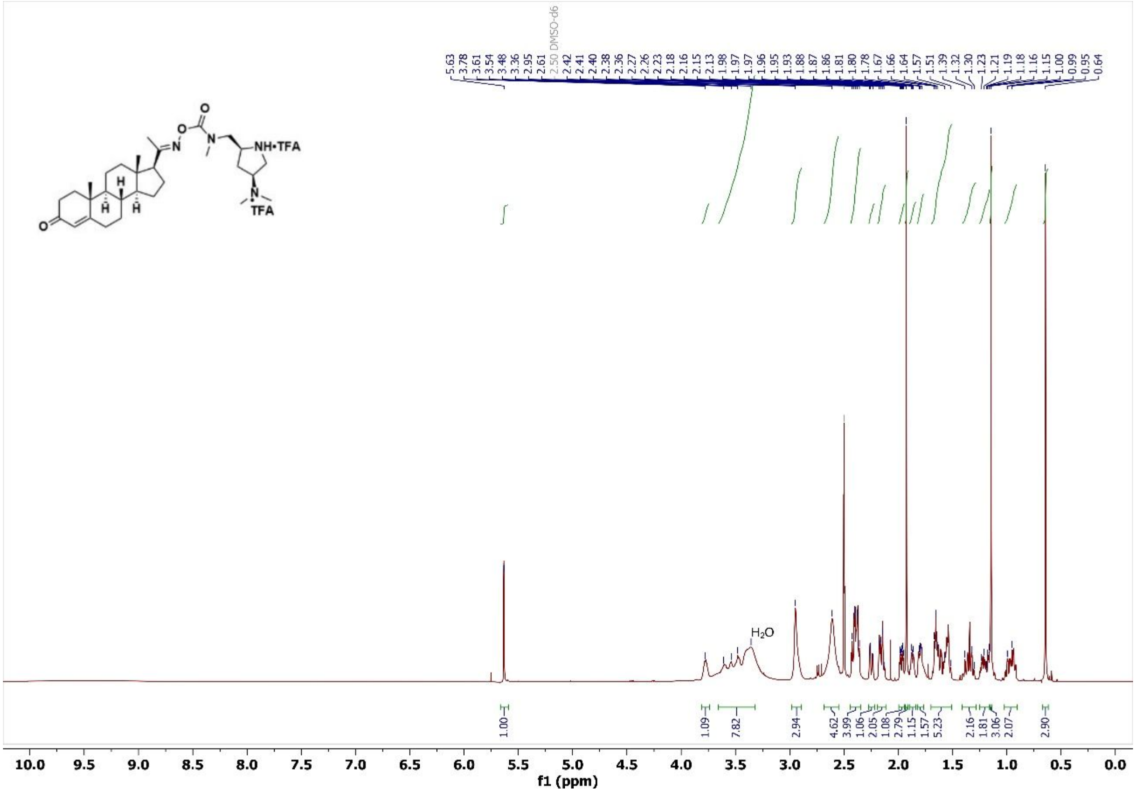

^{1}H NMR of Compound **20u** (600 MHz, DMSO-d_6)

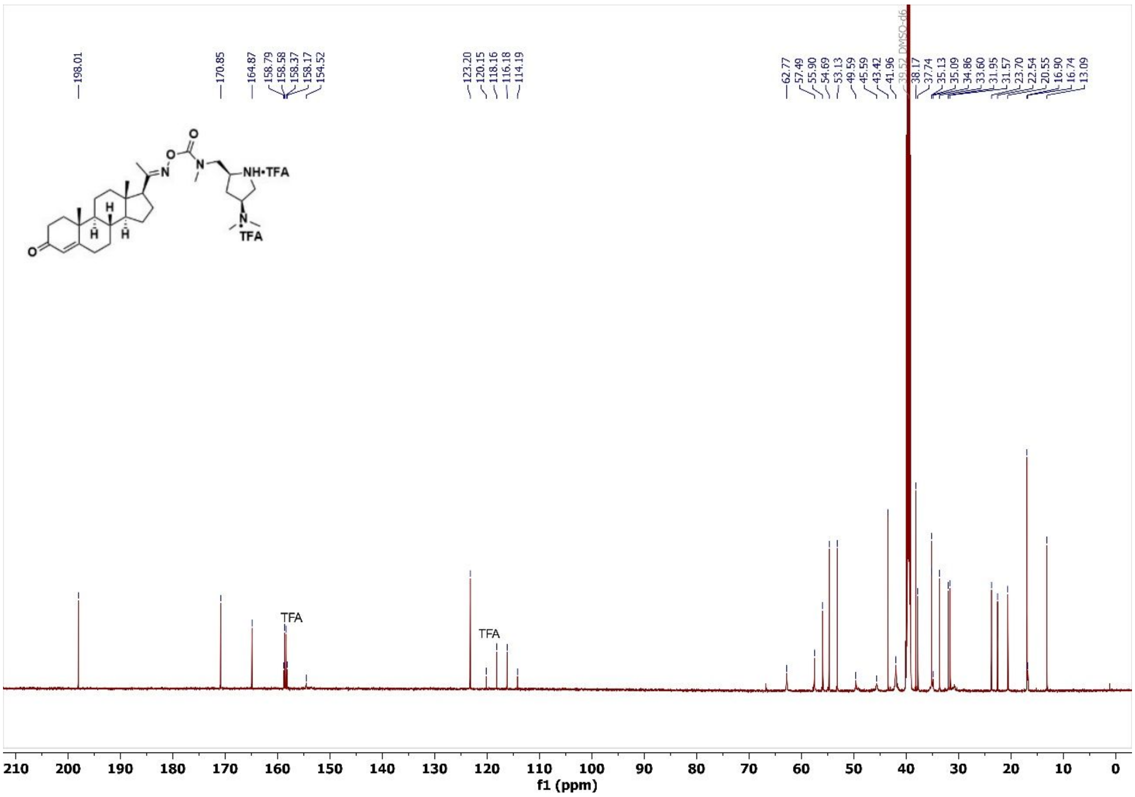

^{13}C NMR of Compound **20u** (151 MHz, DMSO-d_6)

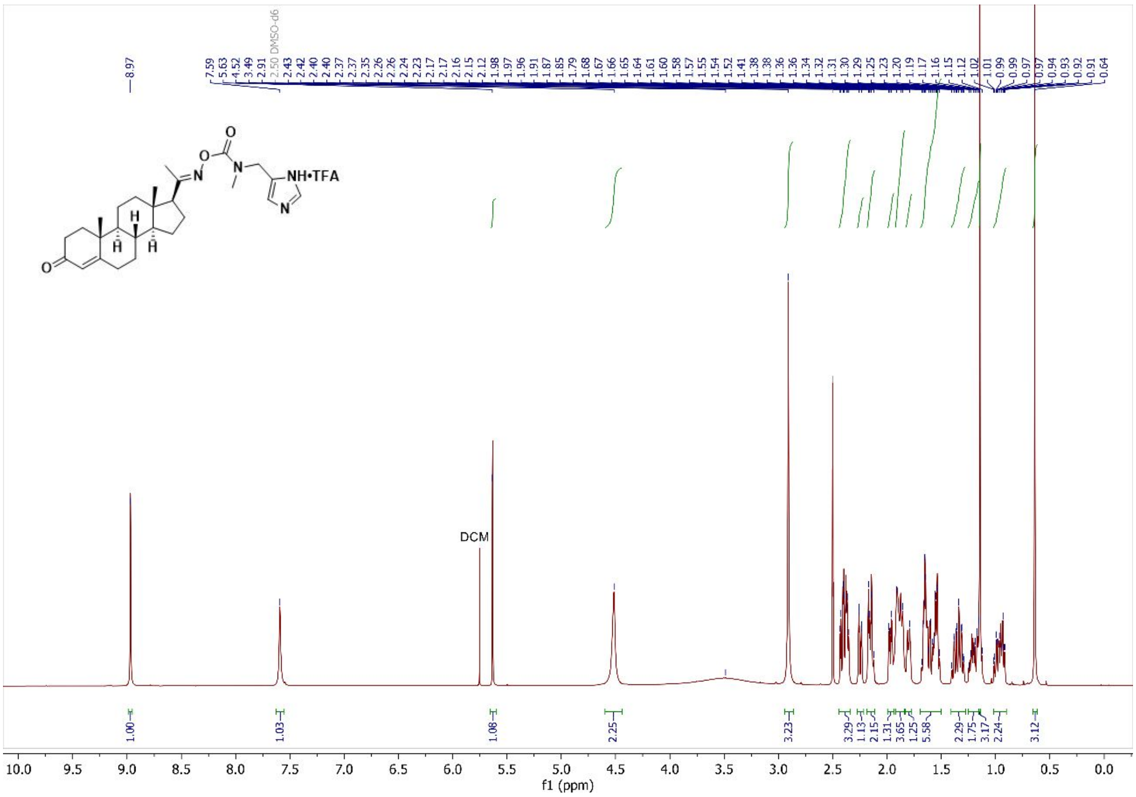

^{1}H NMR of Compound **20v** (600 MHz, DMSO-d_6)

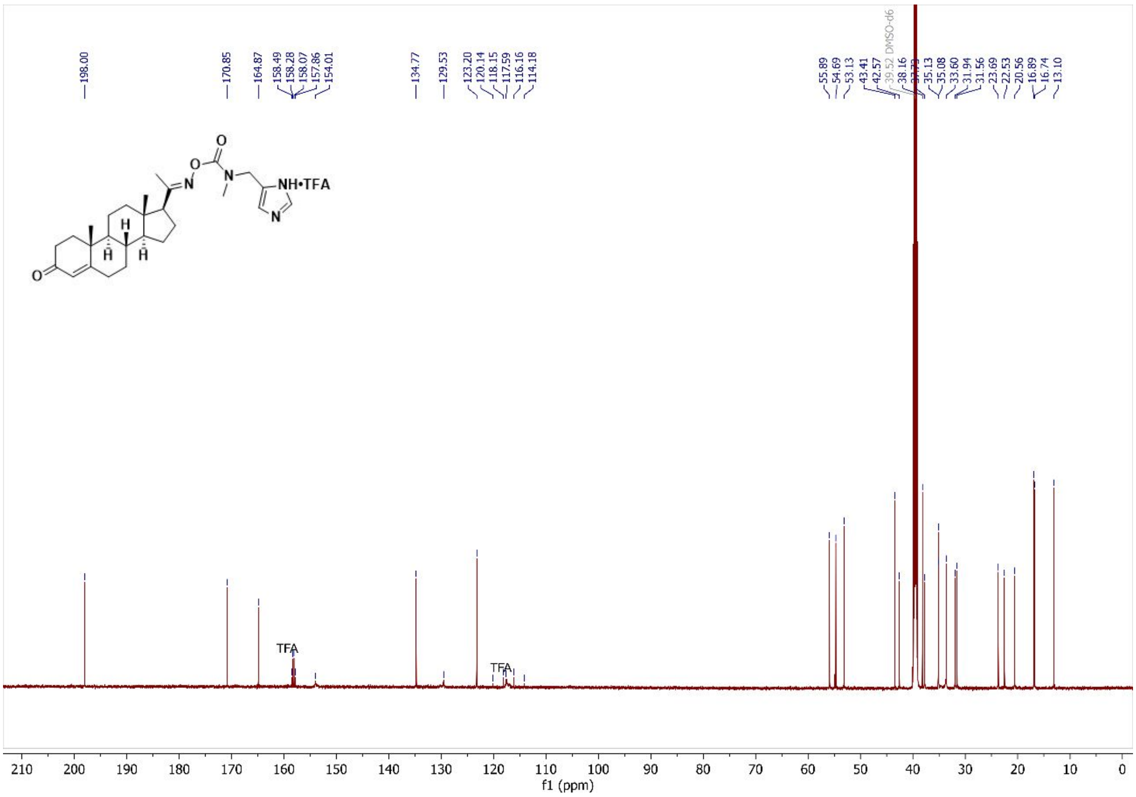

^{13}C NMR of Compound **20v** (151 MHz, DMSO-d_6)

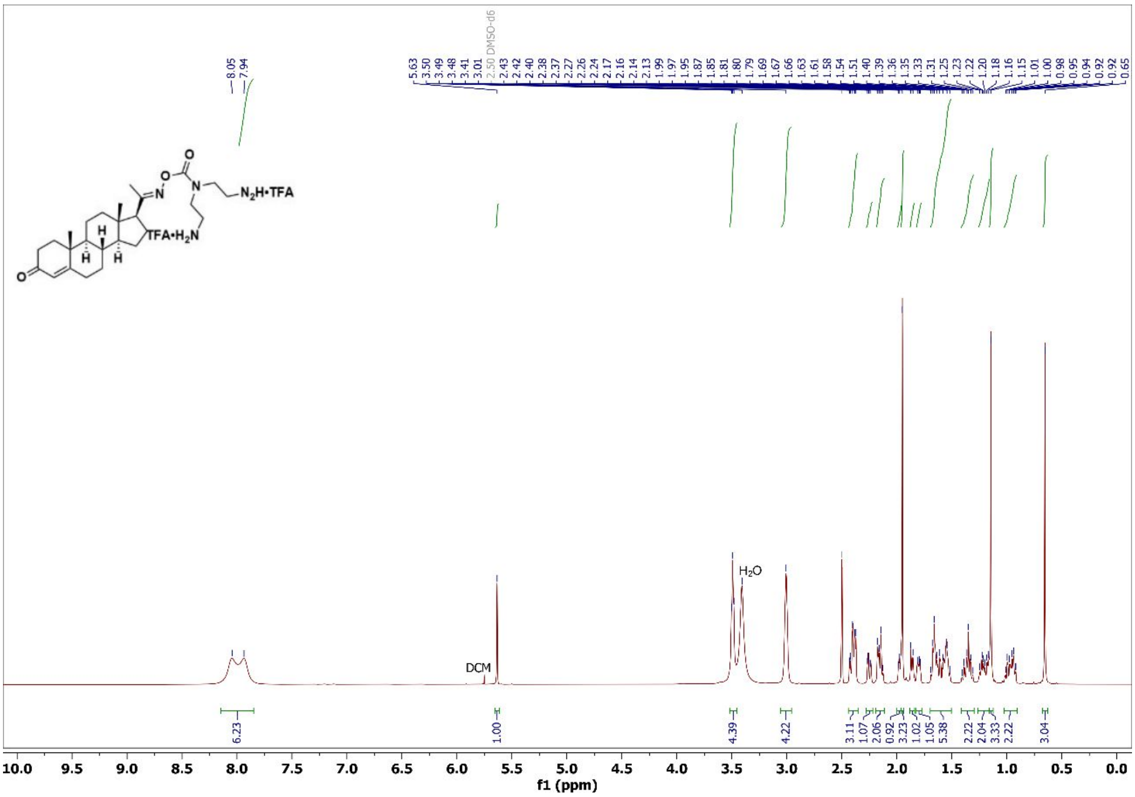

^{1}H NMR of Compound **17w** (600 MHz, DMSO-d_6)

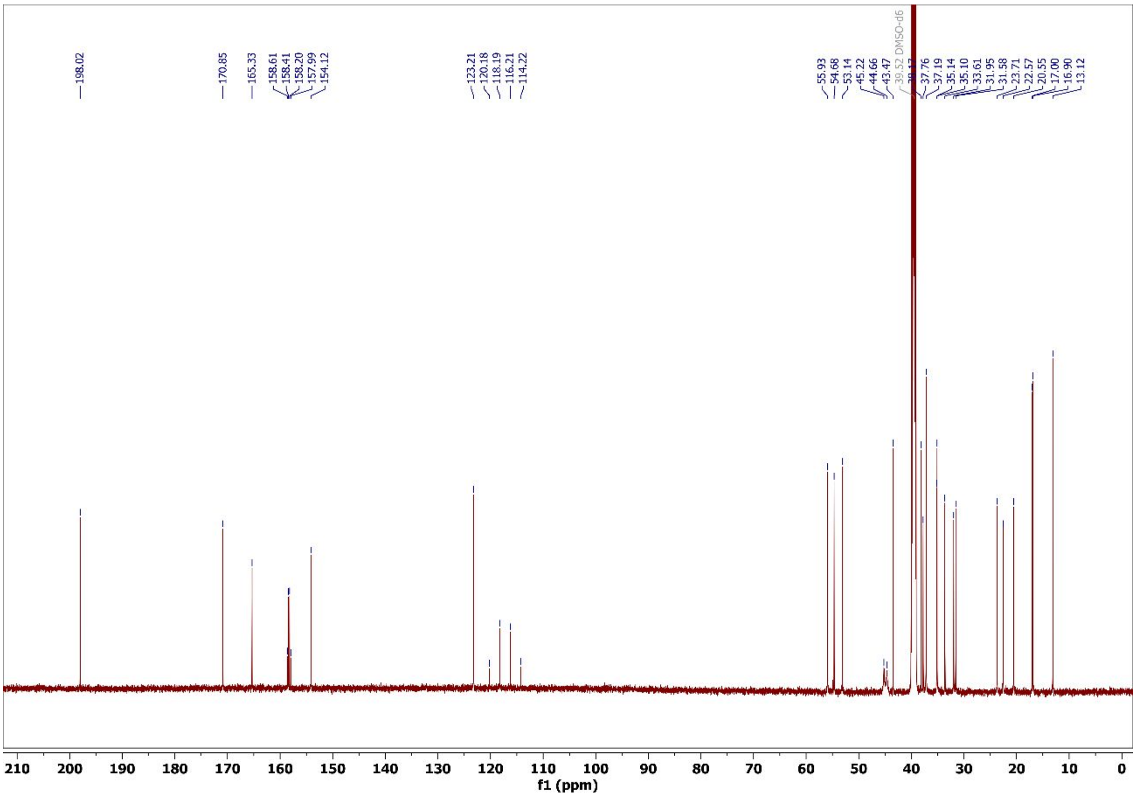

^{13}C NMR of Compound **17w** (151 MHz, DMSO-d_6)

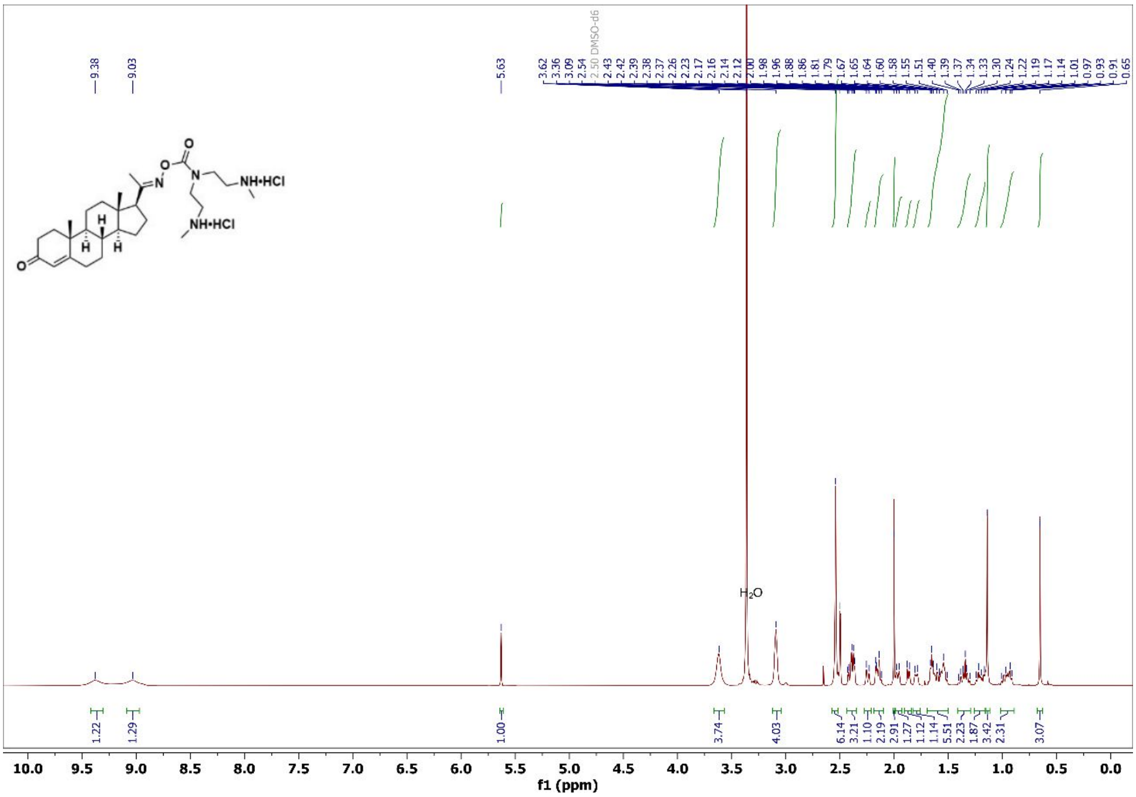

^{1}H NMR of Compound **17x** (600 MHz, DMSO-d_6)

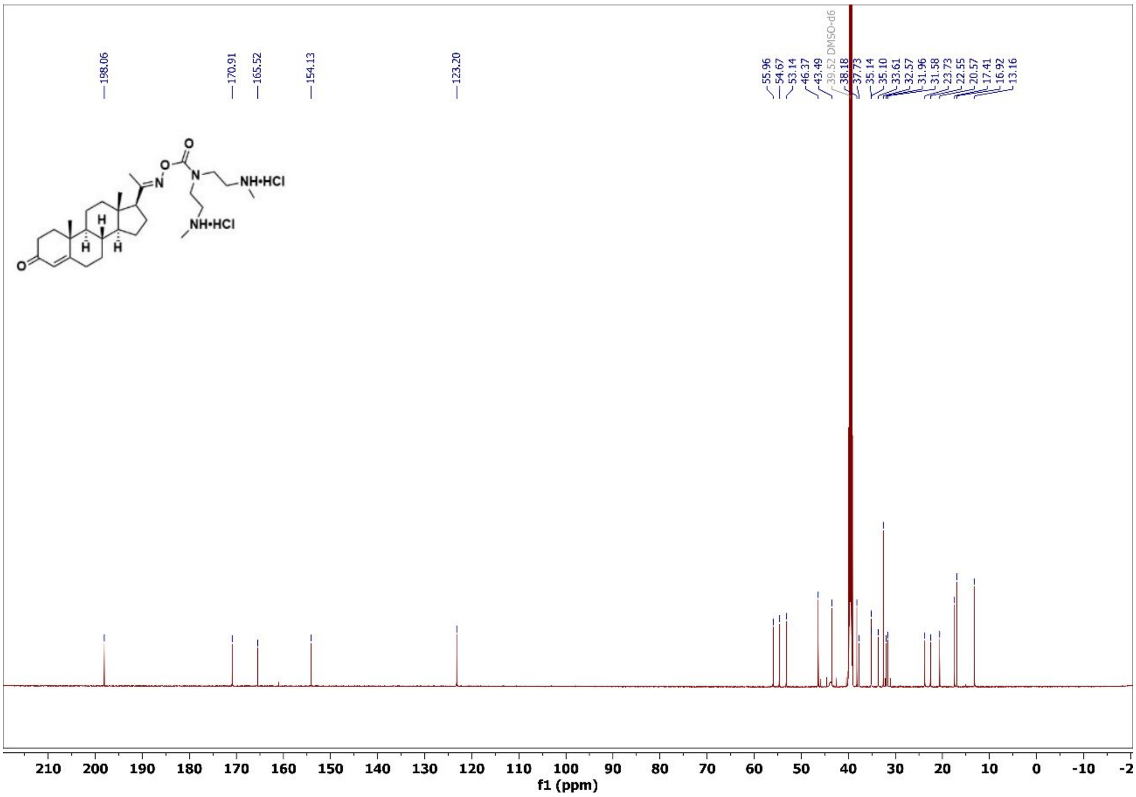

^{13}C NMR of Compound **17x** (151 MHz, DMSO-d_6)

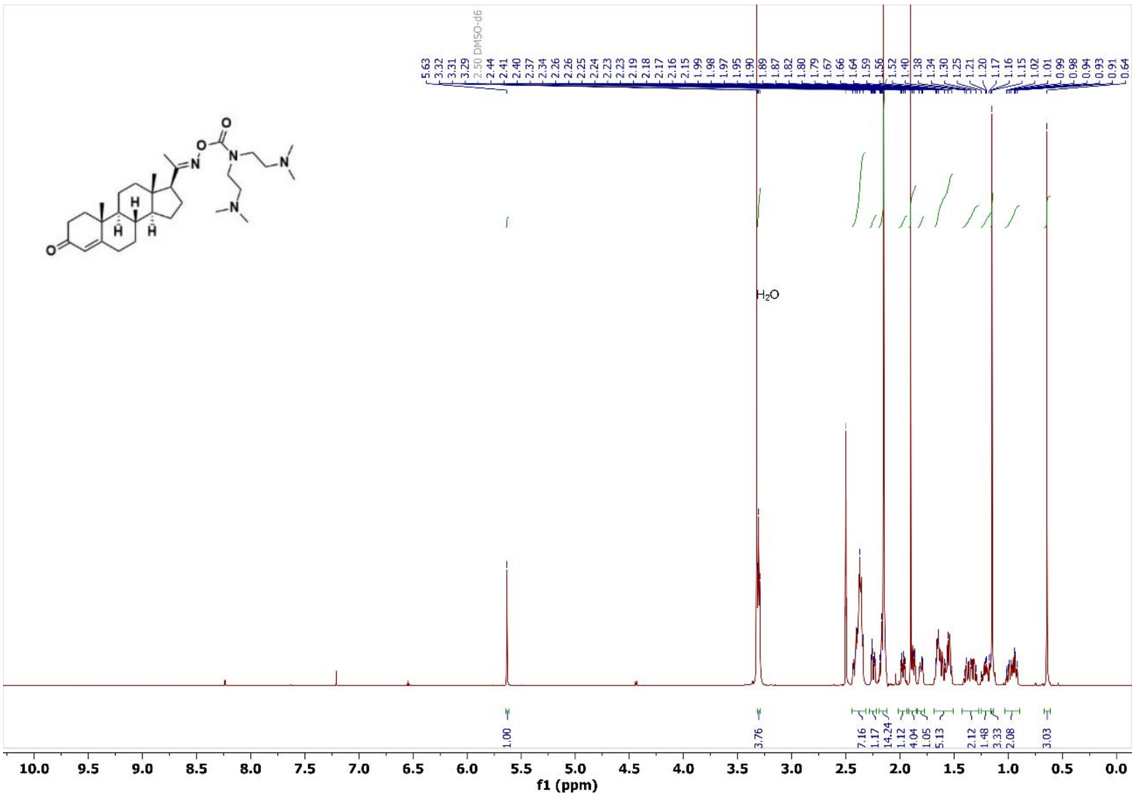

^{1}H NMR of Compound **17y** (600 MHz, DMSO-d_6)

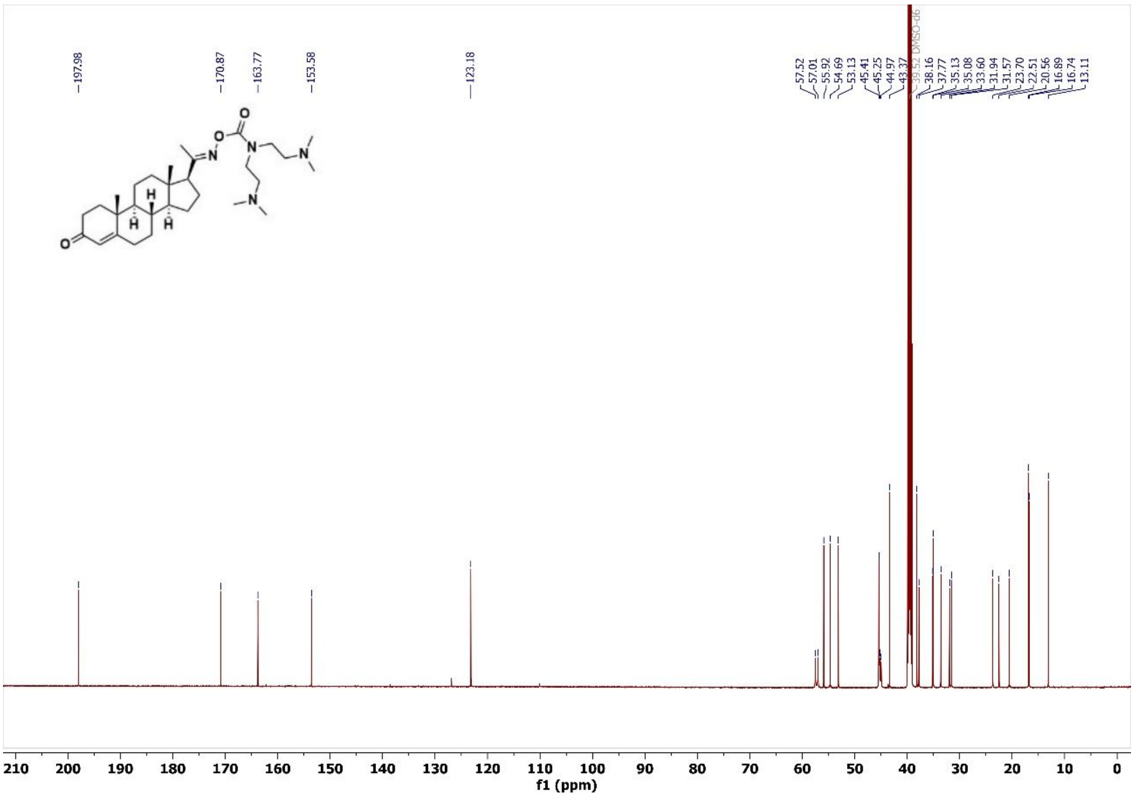

^{13}C NMR of Compound **17y** (151 MHz, DMSO-d_6)

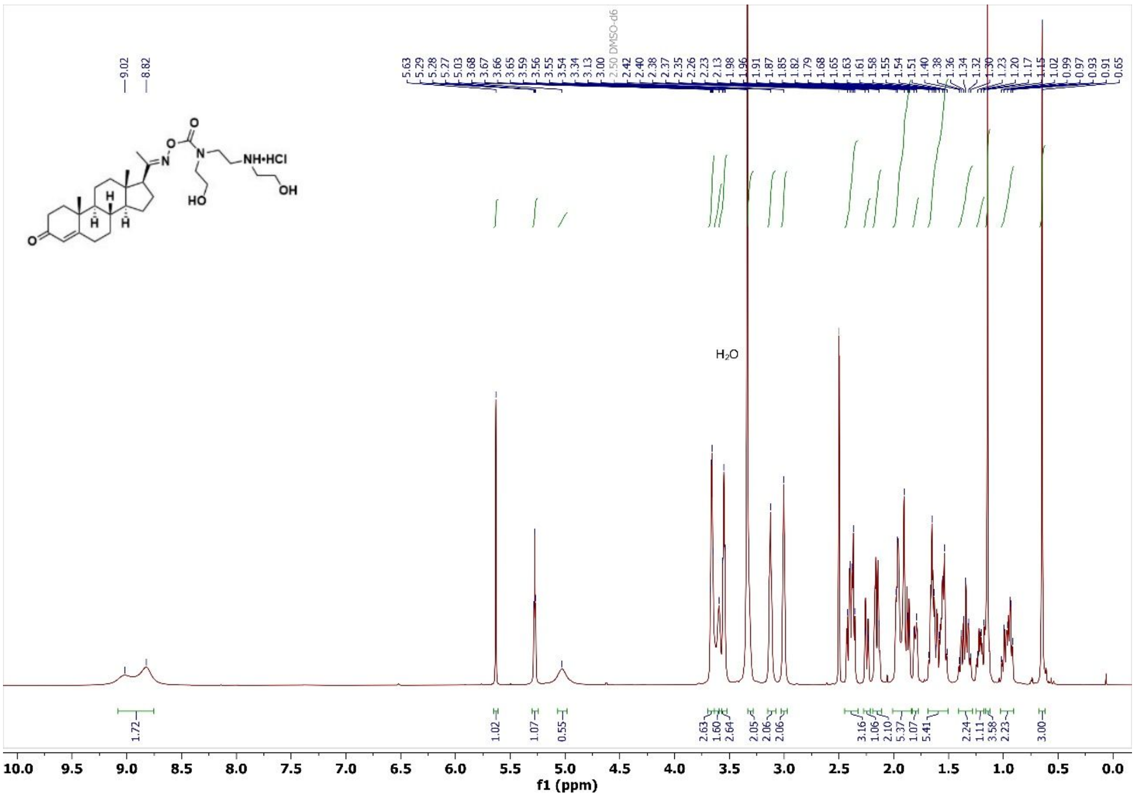

^{1}H NMR of Compound **17z** (600 MHz, DMSO-d_6)

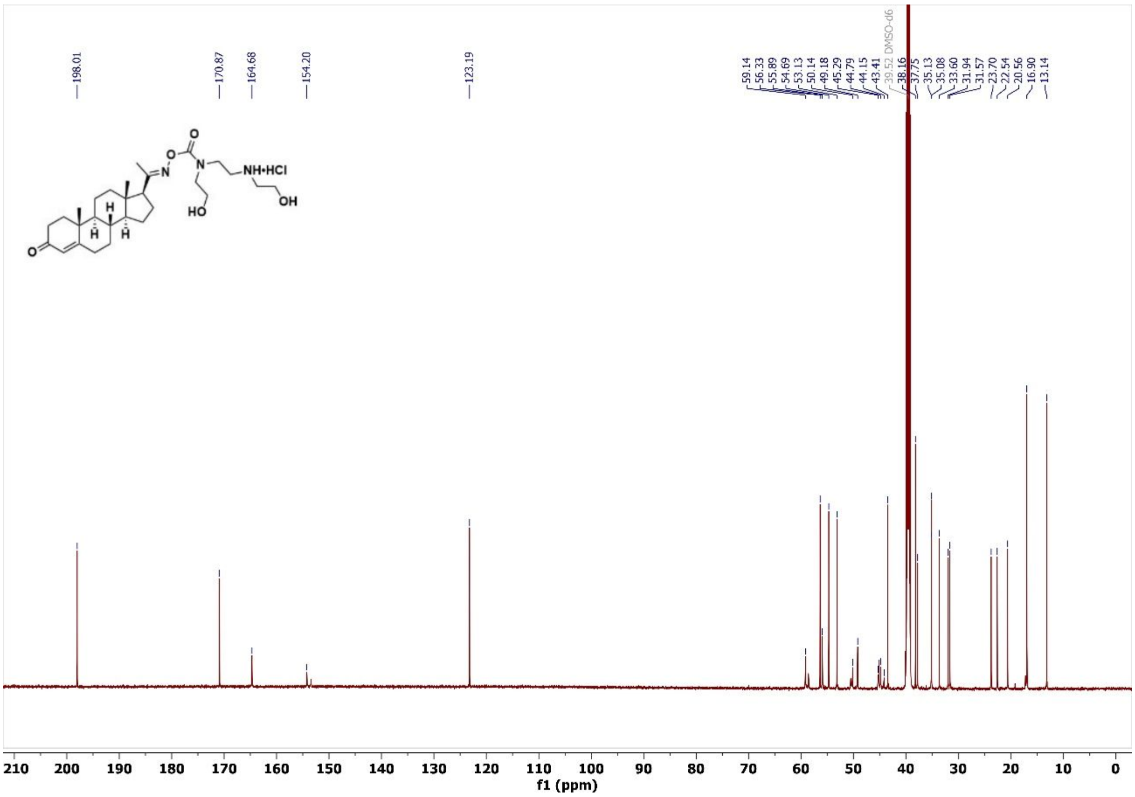

^{13}C NMR of Compound **17z** (151 MHz, DMSO-d_6)

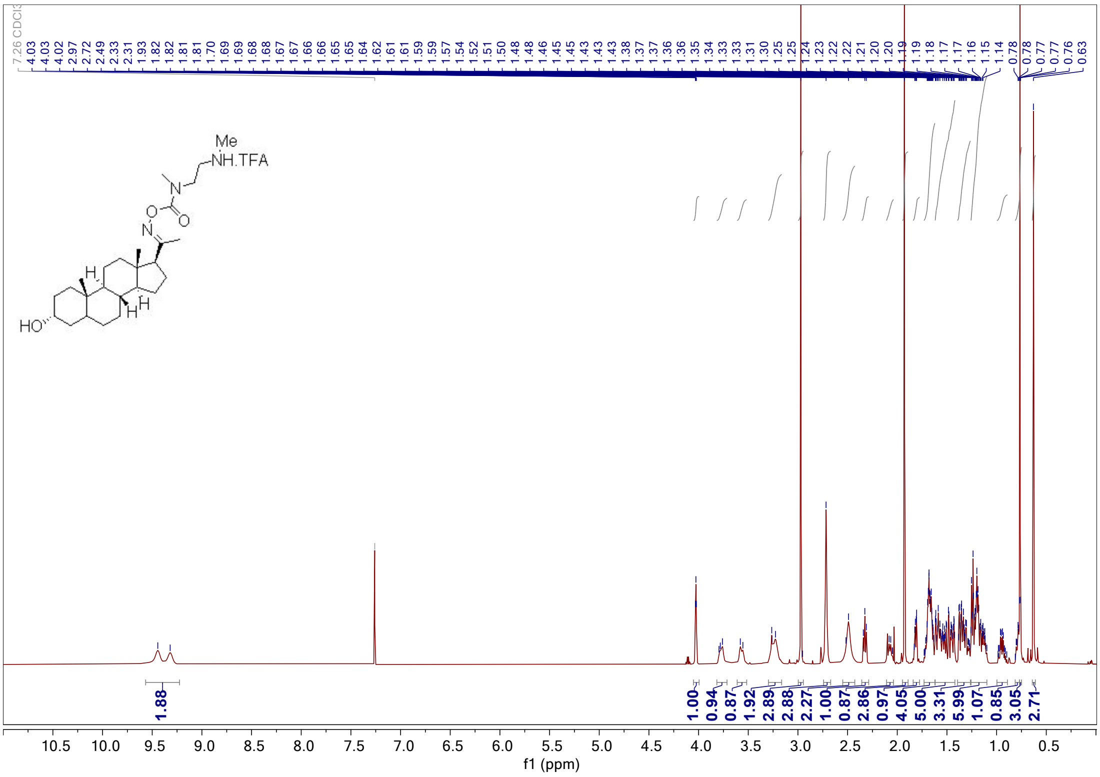

^{1}H NMR of Compound **21a** (600 MHz, $CDCl_3$)

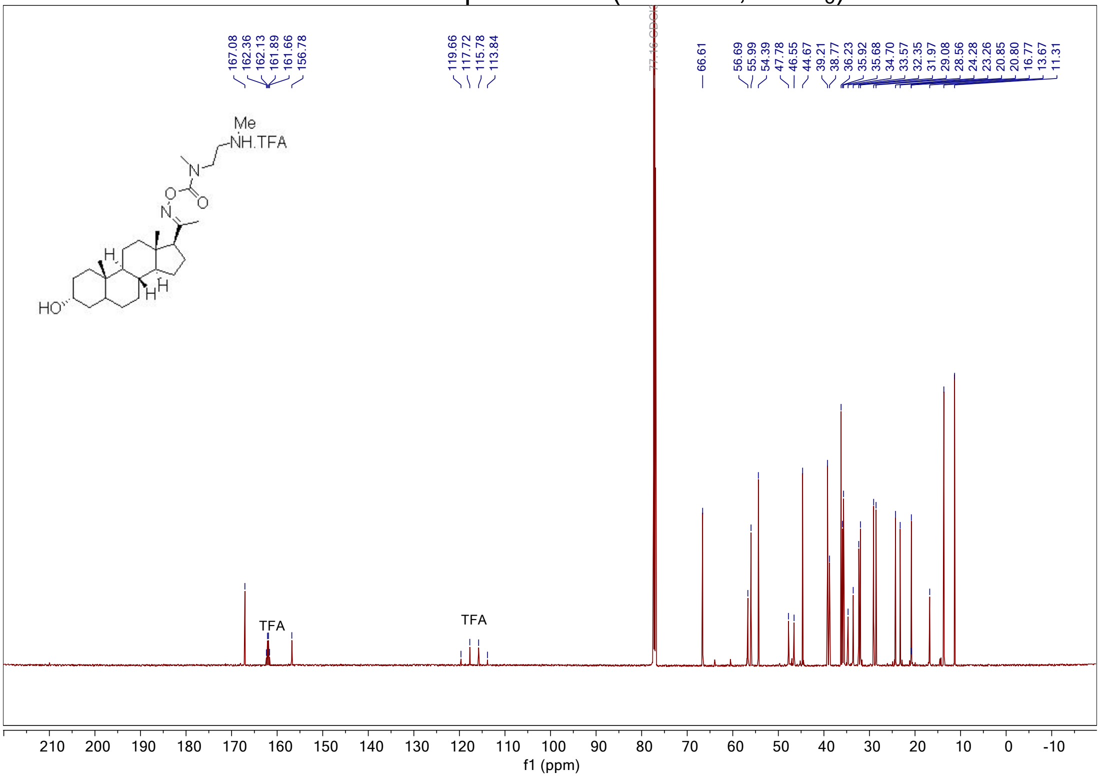

^{13}C NMR of Compound **21a** (151 MHz, DMSO-d_6)

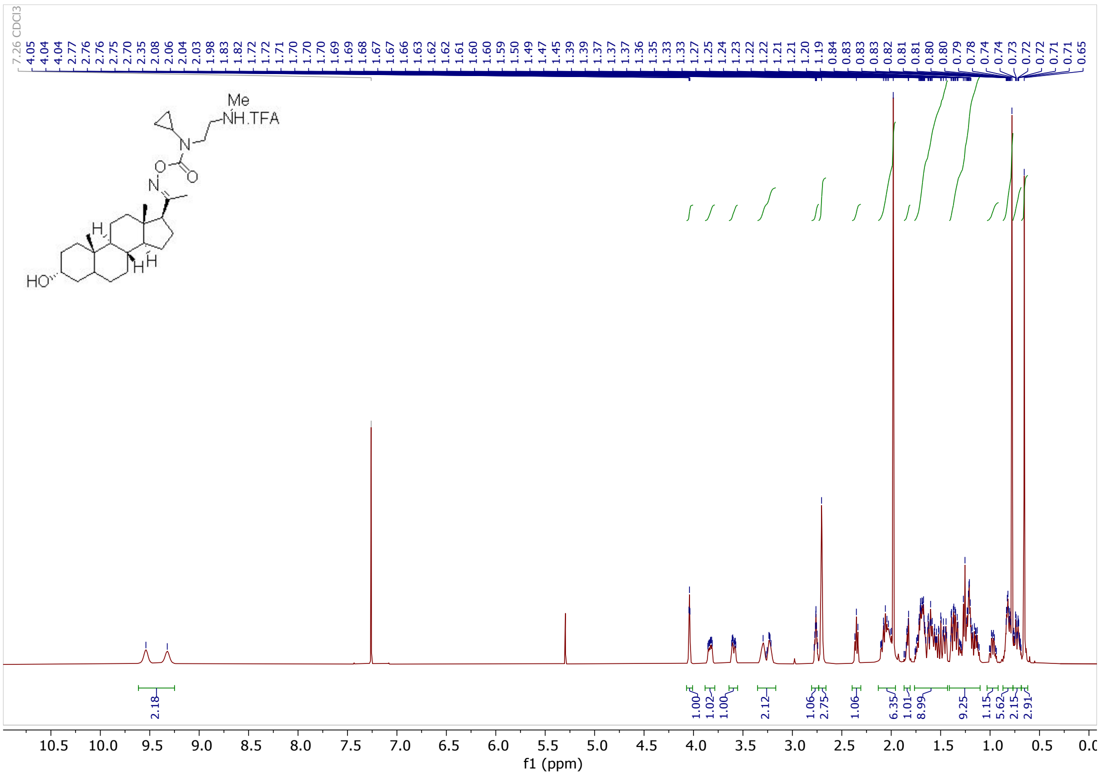

^{1}H NMR of Compound **21b** (600 MHz, $CDCl_3$)

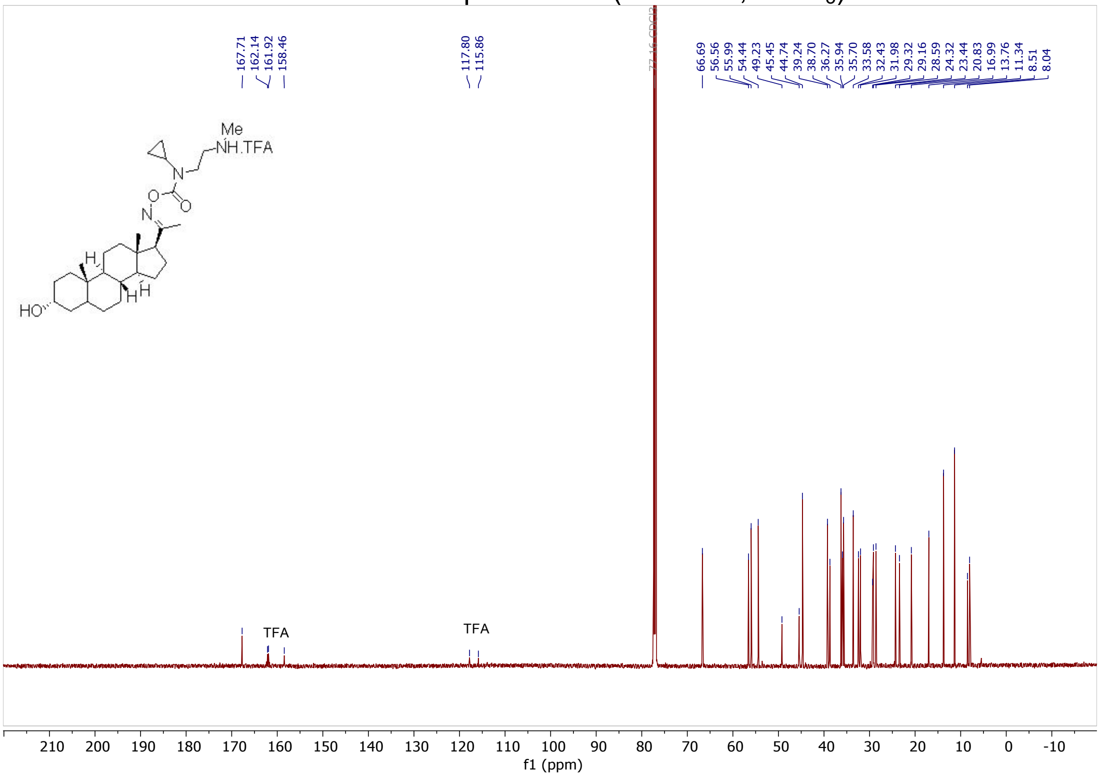

^{13}C NMR of Compound **21b** (151 MHz, $CDCl_3$)

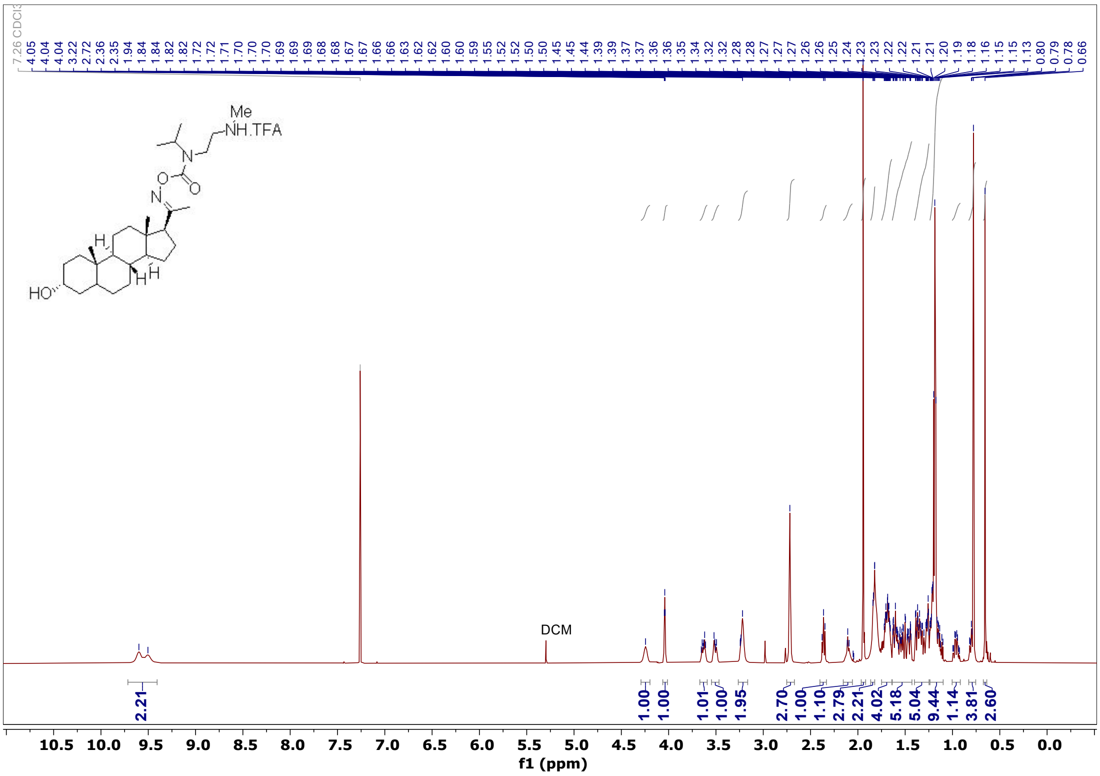

^{1}H NMR of Compound **21c** (600 MHz, $CDCl_3$)

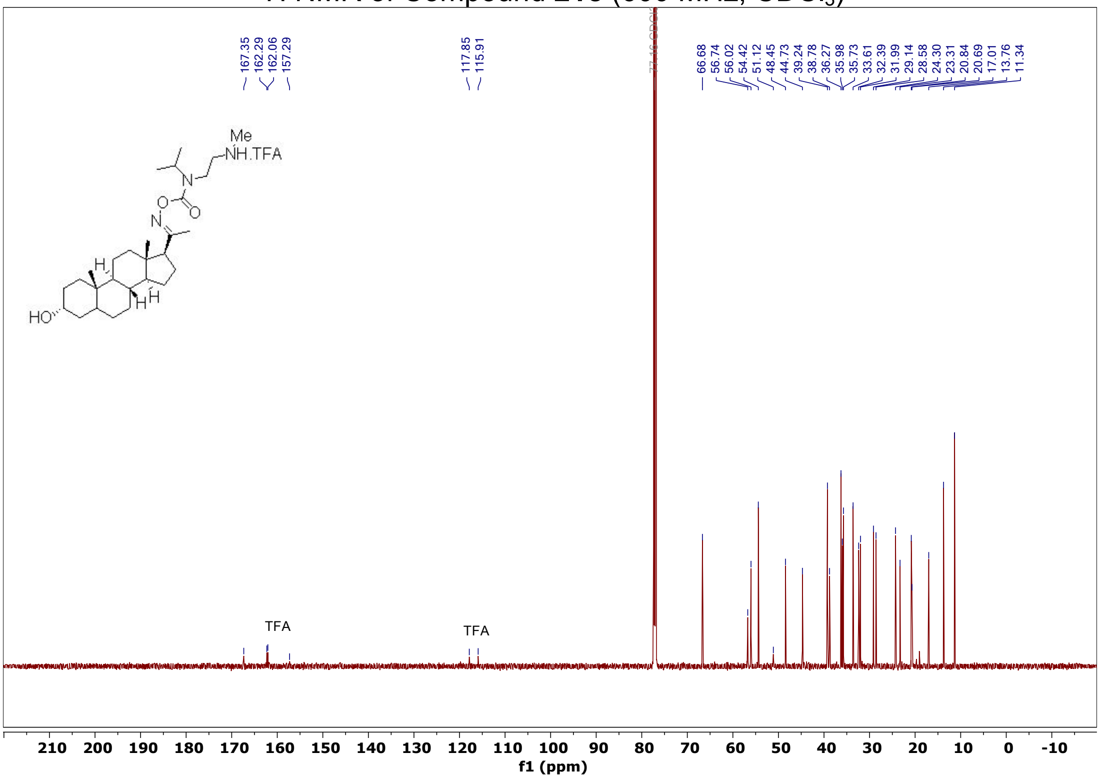

^{13}C NMR of Compound **21c** (151 MHz, $CDCl_3$)

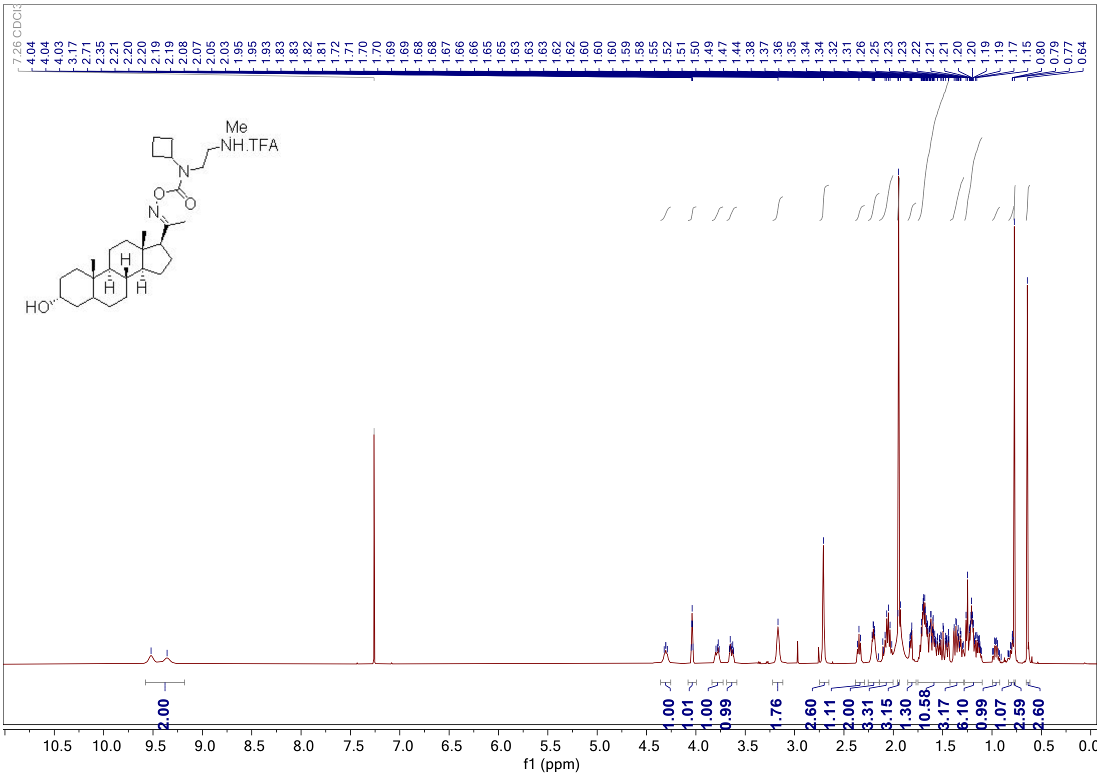

^{1}H NMR of Compound **21d** (600 MHz, $CDCl_3$)

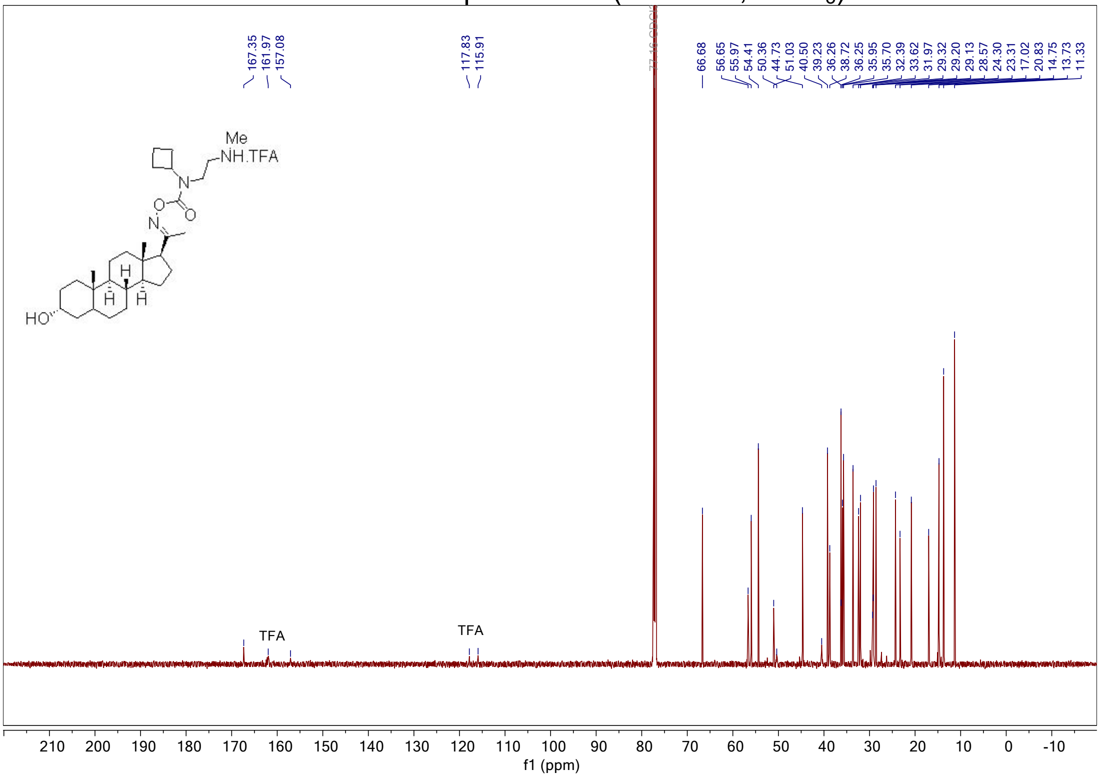

^{13}C NMR of Compound **21d** (151 MHz, $CDCl_3$)

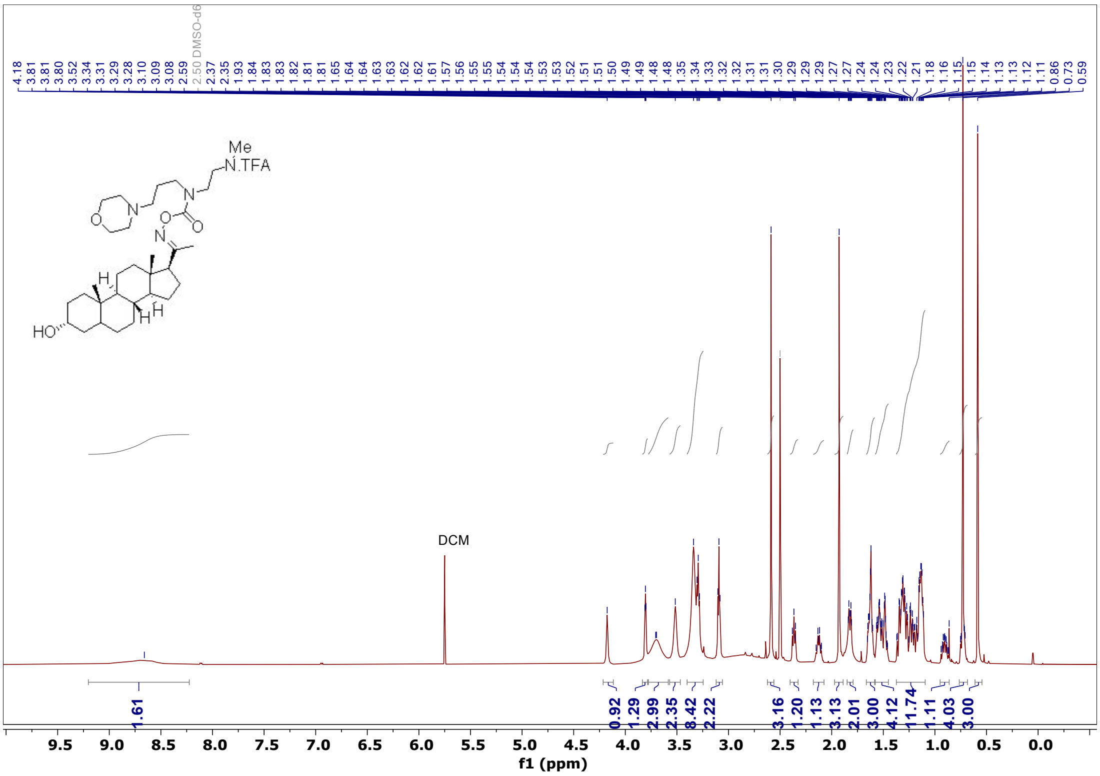

^{1}H NMR of Compound **21j** (600 MHz, DMSO-d_6)

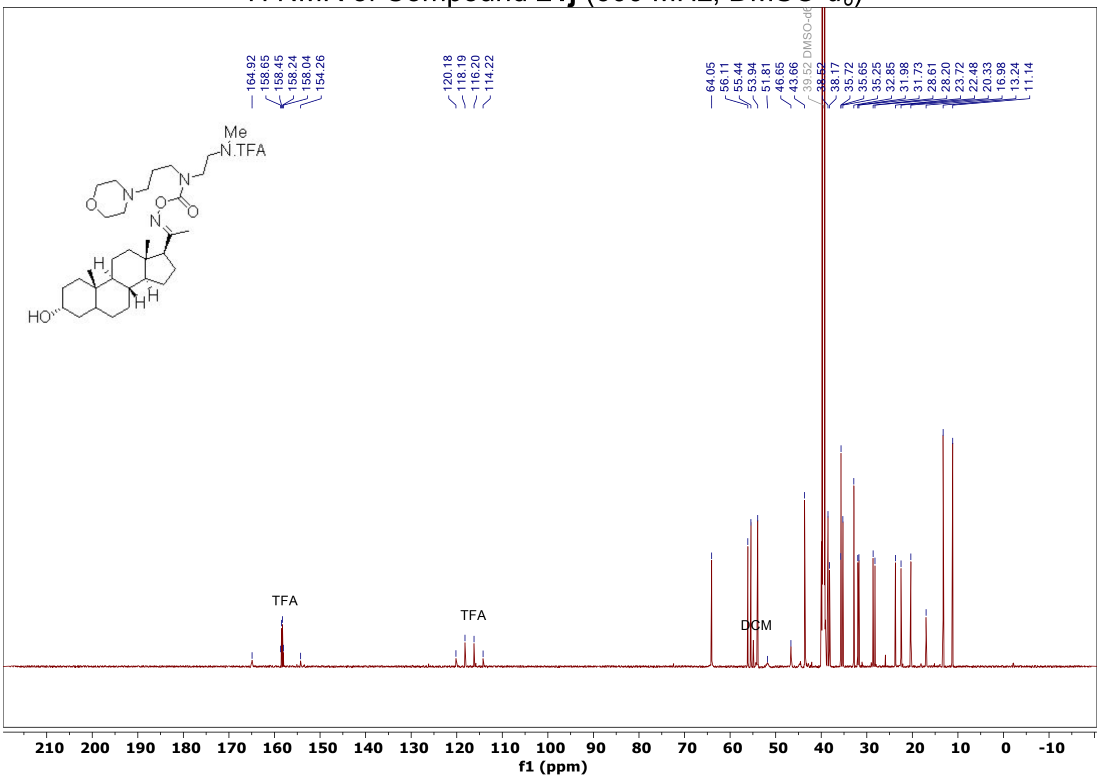

^{13}C NMR of Compound **21j** (151 MHz, DMSO-d_6)

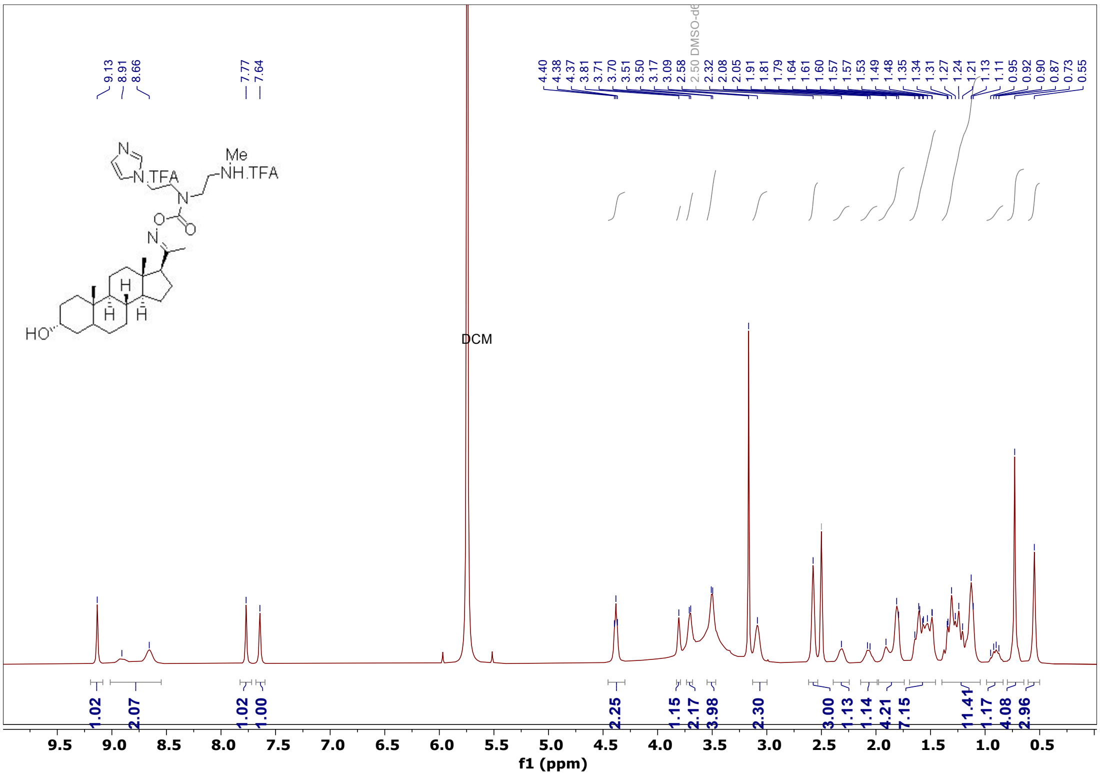

^{1}H NMR of Compound **21k** (400 MHz, DMSO-d_6)

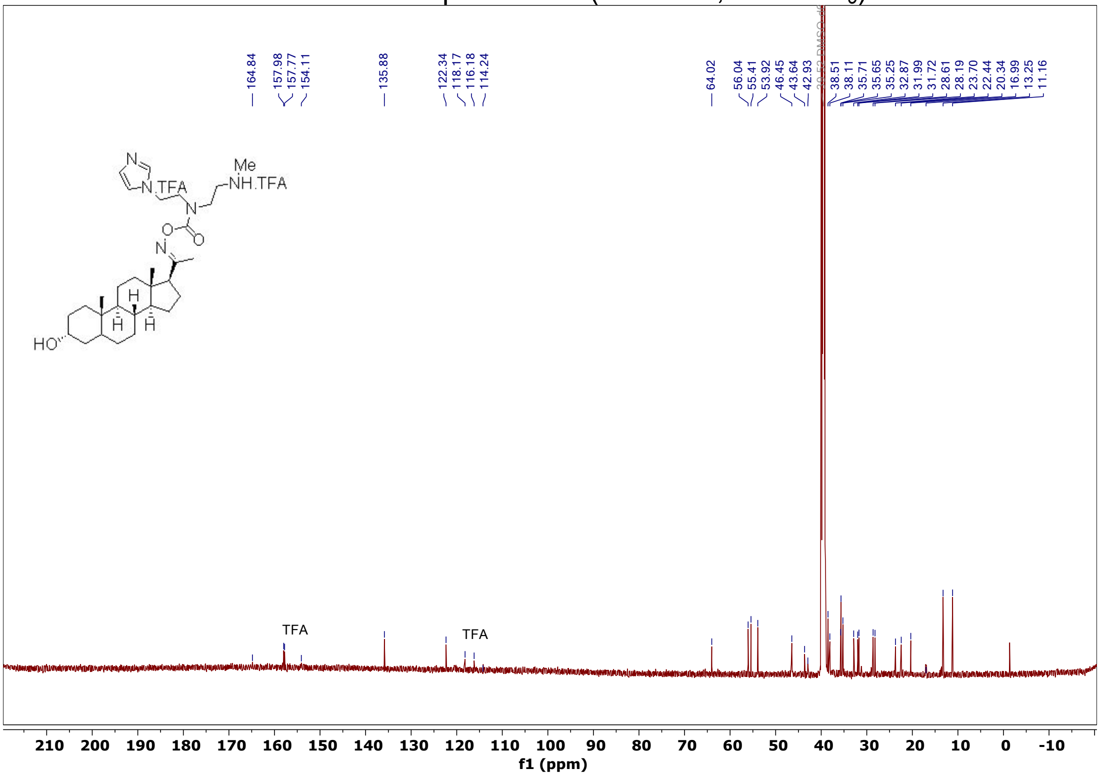

^{13}C NMR of Compound **21k** (151 MHz, DMSO-d_6)

7.6 Representative HPLC Traces of Select Final Compounds

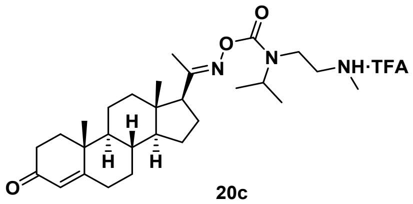

20c
Exact Mass: 471.35

MS Spectrum

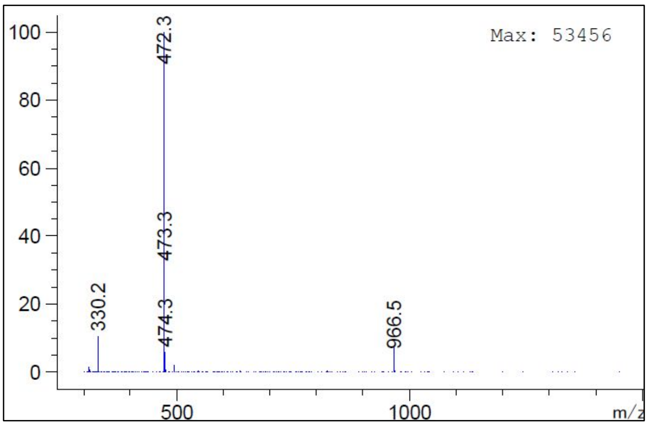

Method: 25–95% MeCN in H_2O (0.1% Formic acid) over 6 min

Signal 2: DAD1 B, Sig=254,4 Ref=360,100

Peak #	RetTime [min]	Type	Width [min]	Area [mAU*s]	Height [mAU]	Area %
1	4.213	BB	0.0831	339.67621	63.31548	100.0000

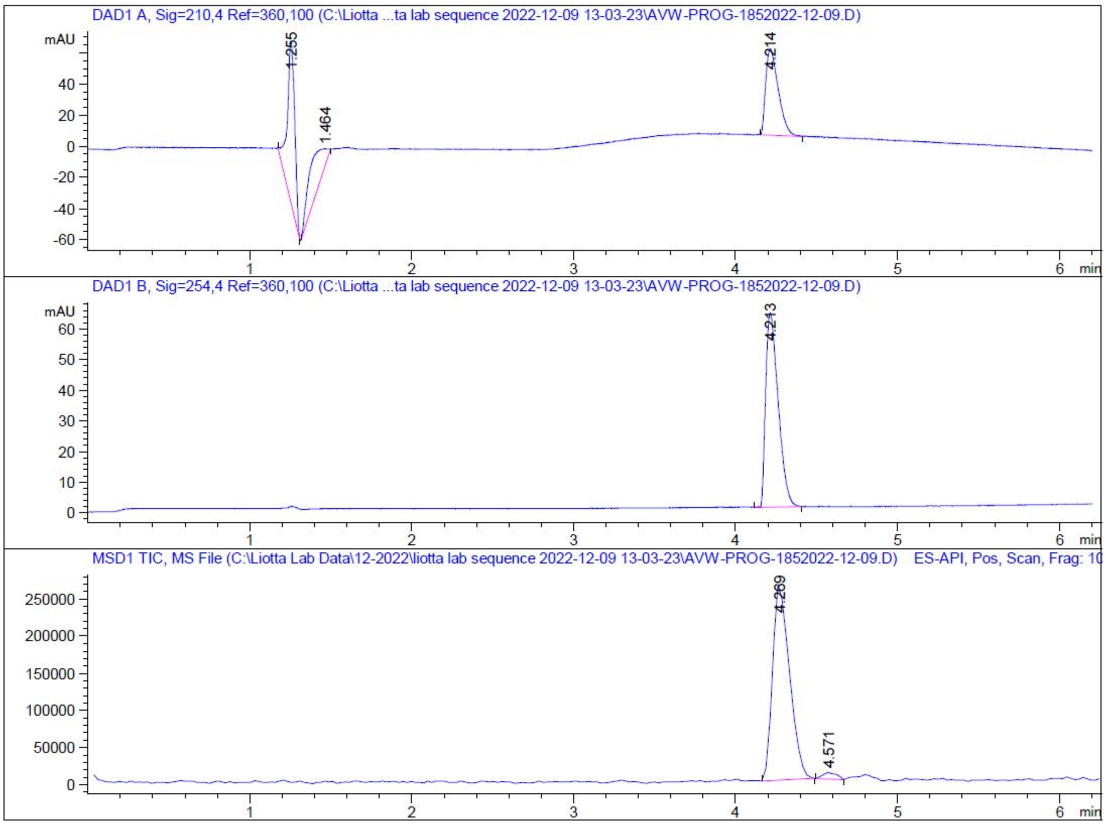

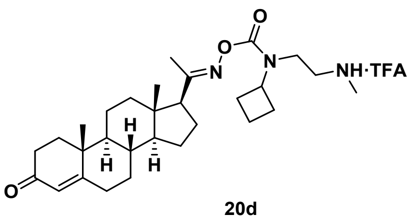

20d
Exact mass: 483.35

MS Spectrum

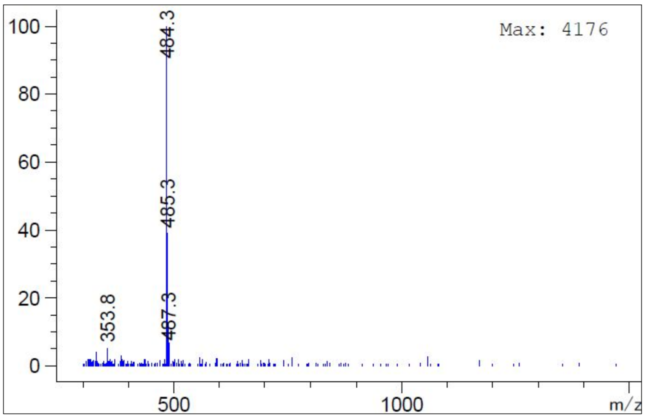

Method: 50–95% MeCN in H_2O (0.1% Formic acid) over 6 min

Signal 2: DAD1 B, Sig=254,4 Ref=360,100

Peak #	RetTime [min]	Type	Width [min]	Area [mAU*s]	Height [mAU]	Area %
1	1.637	BB	0.0894	496.79886	80.06100	100.0000

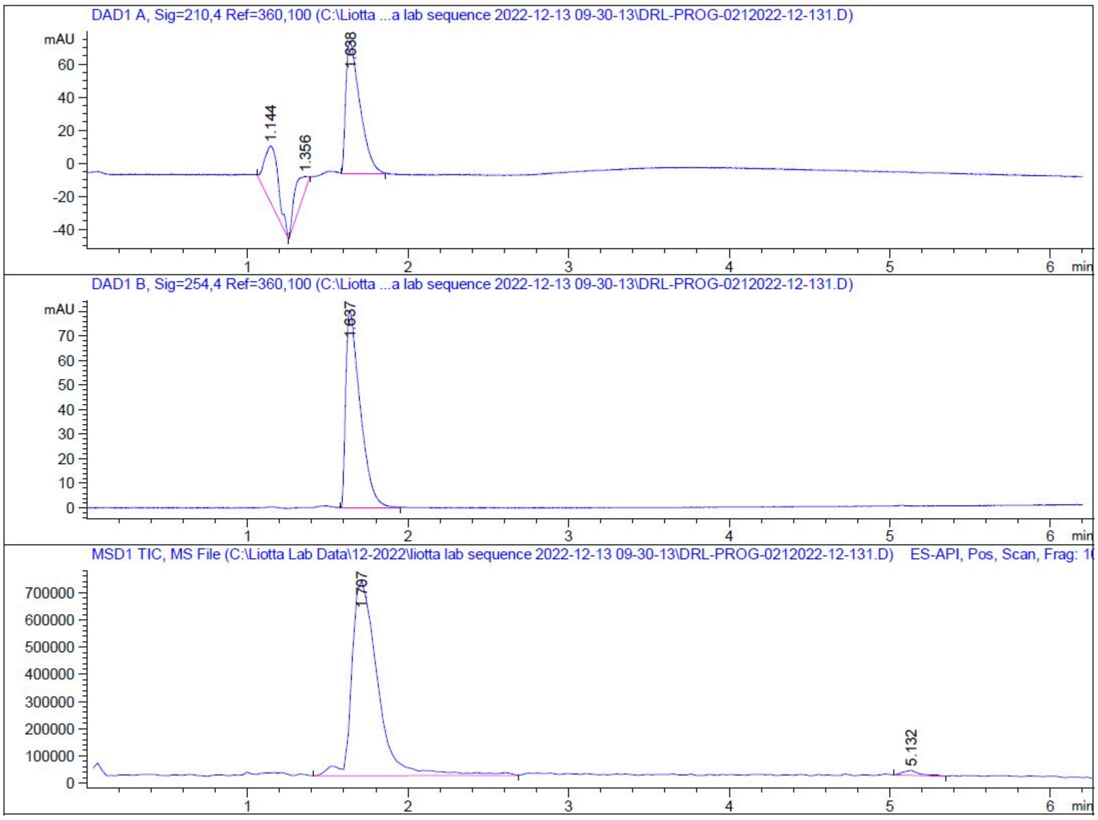

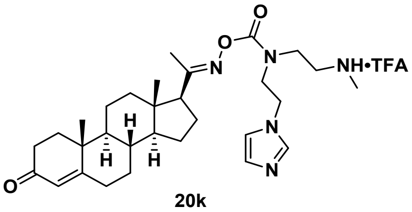

20k
Exact mass: 523.35

MS Spectrum

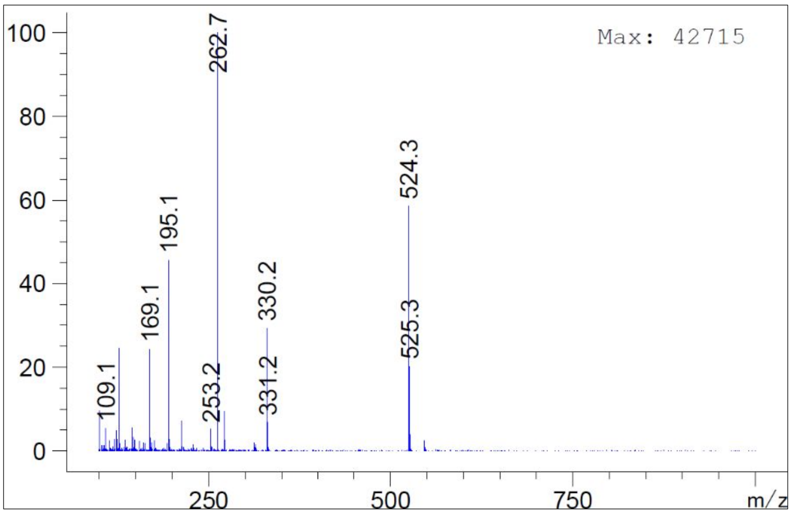

Method: 10–95% MeCN in H_2O (0.1% Formic acid) over 6 min

Signal 2: DAD1 B, Sig=254,4 Ref=360,100

Peak #	RetTime [min]	Type	Width [min]	Area [mAU*s]	Height [mAU]	Area %
1	4.701	BB	0.0479	330.51730	107.36646	100.0000

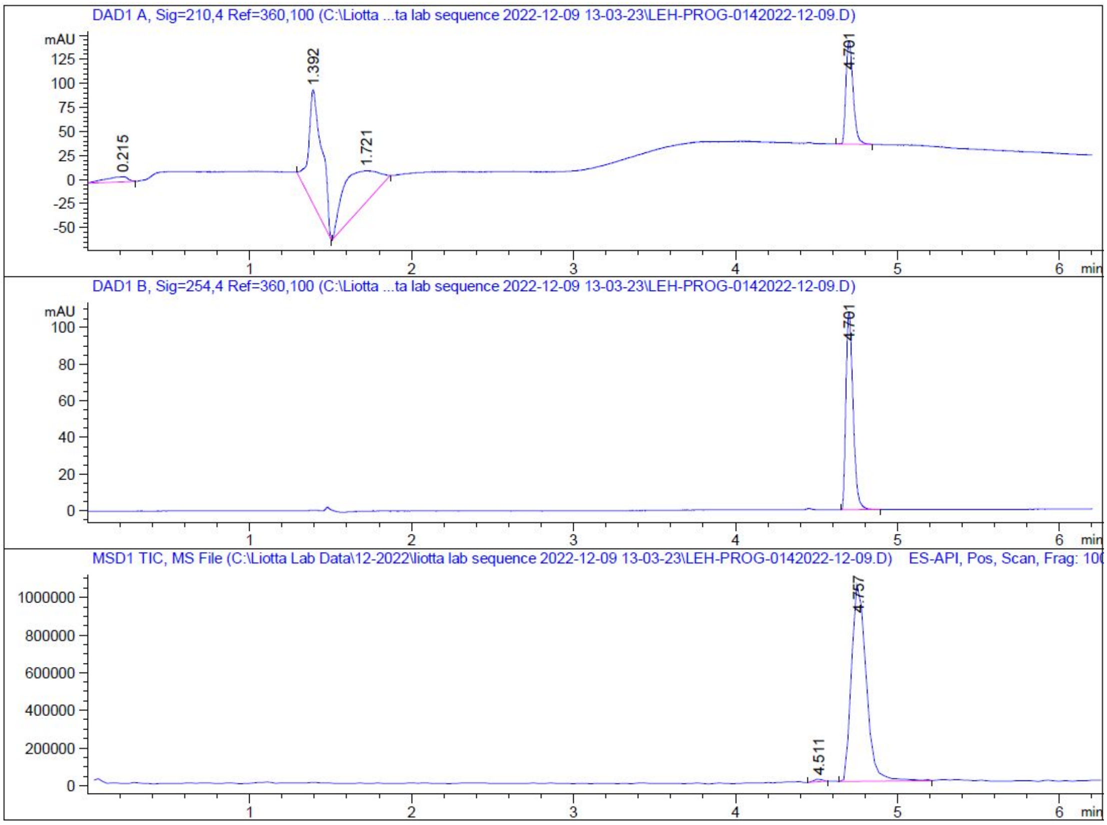

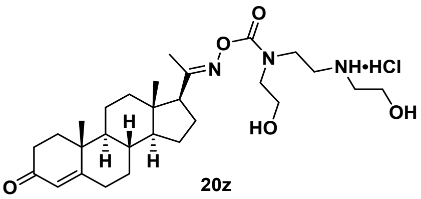

20z
Exact mass: 503.34

MS Spectrum

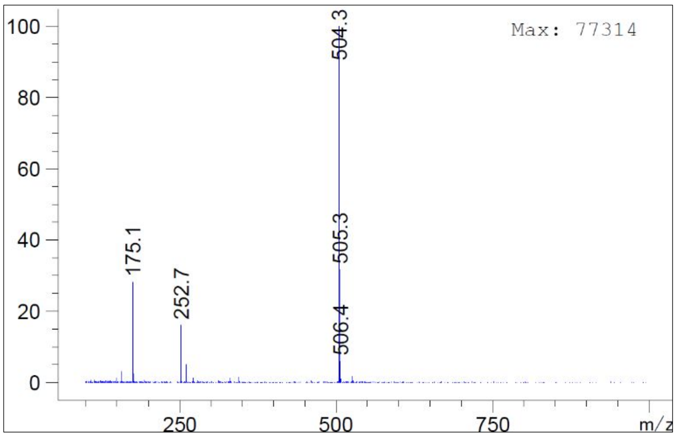

Method: 25–95% MeCN in H_2O (0.1% Formic acid) over 6 min

Signal 2: DAD1 B, Sig=254,4 Ref=360,100

Peak #	RetTime [min]	Type	Width [min]	Area [mAU*s]	Height [mAU]	Area %
1	4.434	BB	0.0567	364.66406	100.88381	100.0000

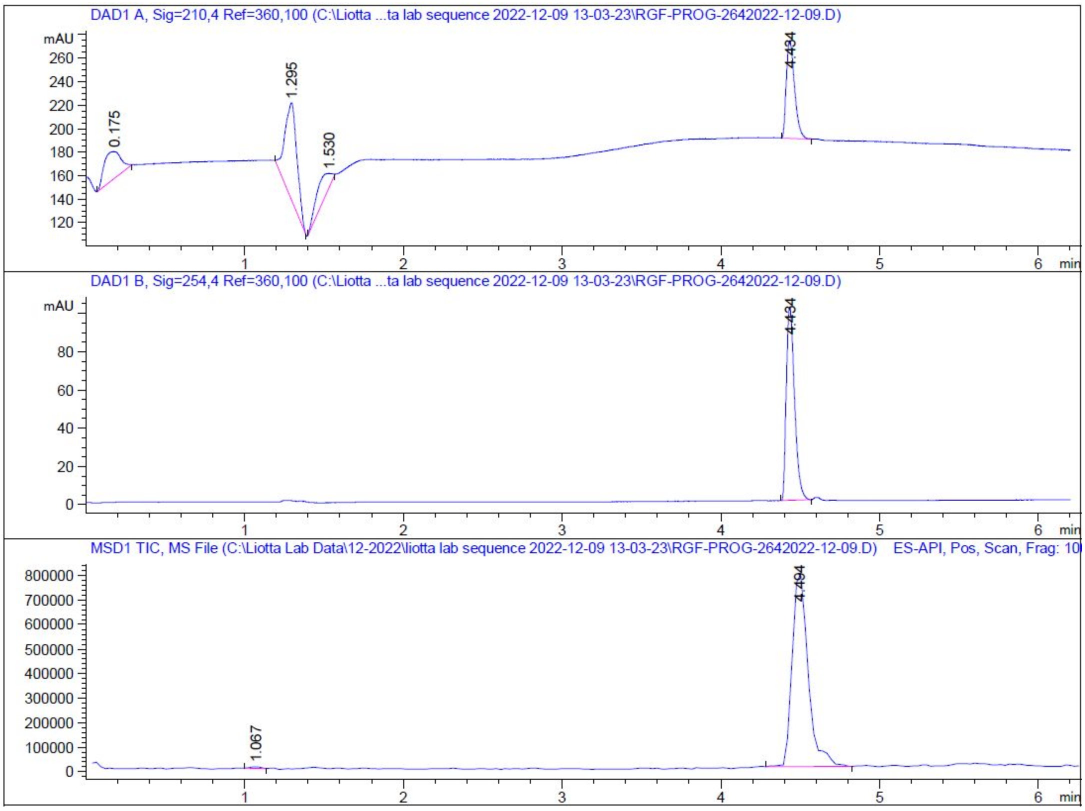

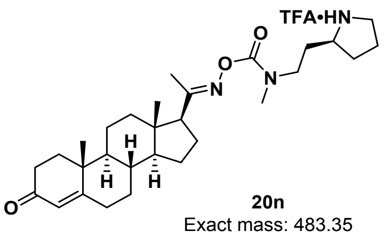

20n
Exact mass: 483.35

MS Spectrum

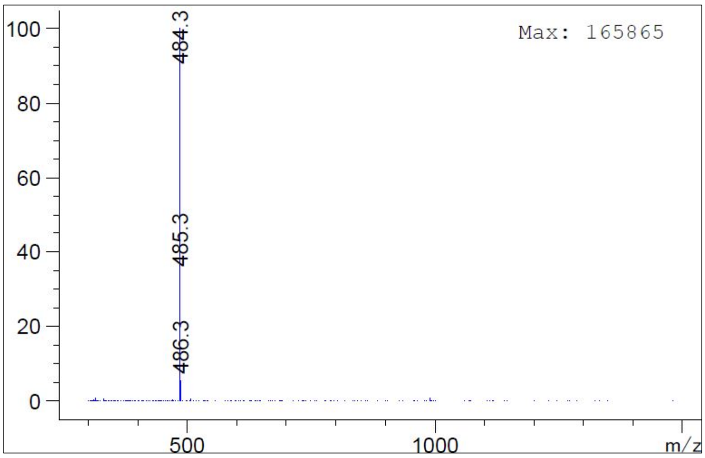

Method: 25–95% MeCN in H_2O (0.1% Formic acid) over 6 min

```
Signal 2: DAD1 B, Sig=254,4 Ref=360,100

Peak RetTime Type  Width     Area      Height     Area
  #   [min]        [min]   [mAU*s]     [mAU]        %
----|-------|----|-------|----------|----------|--------|
   1   3.932 BB    0.0814  231.64925   43.32899 100.0000
```

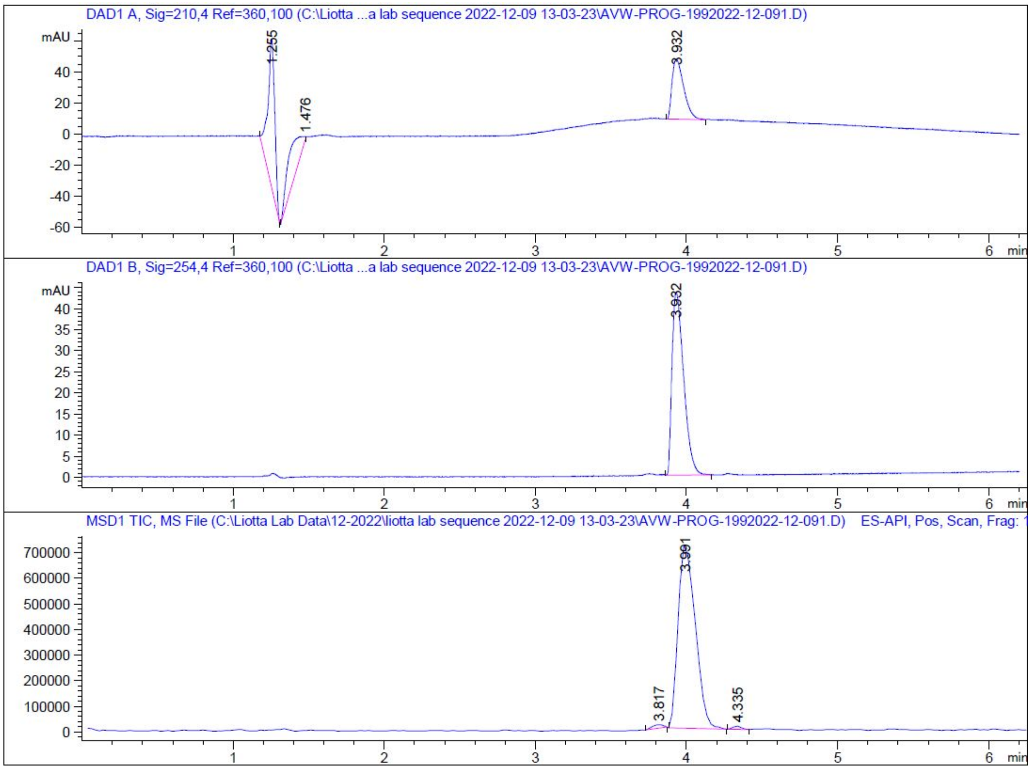

8. References

(1) Schrödinger Release 2025-2: Maestro. Schrödinger: New York, NY, 2025.

(2) Mohamadi, F.; Richards, N. G. J.; Guida, W. C.; Liskamp, R.; Lipton, M.; Caufield, C.; Chang, G.; Hendrickson, T.; Still, W. C. Macromodel—an integrated software system for modeling organic and bioorganic molecules using molecular mechanics. *Journal of Computational Chemistry* **1990**, *11* (4), 440-467. DOI: https://doi.org/10.1002/jcc.540110405.

(3) Lu, C.; Wu, C.; Ghoreishi, D.; Chen, W.; Wang, L.; Damm, W.; Ross, G. A.; Dahlgren, M. K.; Russell, E.; Von Bargen, C. D.; et al. OPLS4: Improving Force Field Accuracy on Challenging Regimes of Chemical Space. *Journal of Chemical Theory and Computation* **2021**, *17* (7), 4291-4300. DOI: 10.1021/acs.jctc.1c00302.

(4) Bochevarov, A. D.; Harder, E.; Hughes, T. F.; Greenwood, J. R.; Braden, D. A.; Philipp, D. M.; Rinaldo, D.; Halls, M. D.; Zhang, J.; Friesner, R. A. Jaguar: A high-performance quantum chemistry software program with strengths in life and materials sciences. *International Journal of Quantum Chemistry* **2013**, *113* (18), 2110-2142. DOI: https://doi.org/10.1002/qua.24481.

(5) Johnston, R. C.; Yao, K.; Kaplan, Z.; Chelliah, M.; Leswing, K.; Seekins, S.; Watts, S.; Calkins, D.; Chief Elk, J.; Jerome, S. V.; et al. Epik: pKa and Protonation State Prediction through Machine Learning. *Journal of Chemical Theory and Computation* **2023**, *19* (8), 2380-2388. DOI: 10.1021/acs.jctc.3c00044.

(6) Dal Corso, A.; Frigoli, M.; Prevosti, M.; Mason, M.; Bucci, R.; Belvisi, L.; Pignataro, L.; Gennari, C. Advanced Pyrrolidine-Carbamate Self-Immolative Spacer with Tertiary Amine Handle Induces Superfast Cyclative Drug Release. *ChemMedChem* **2022**, *17* (15). DOI: 10.1002/cmdc.202200279.

(7) Deetz, M. J.; Forbes, C. C.; Jonas, M.; Malerich, J. P.; Smith, B. D.; Wiest, O. Unusually Low Barrier to Carbamate C−N Rotation. *The Journal of Organic Chemistry* **2002**, *67* (11), 3949-3952. DOI: 10.1021/jo025554u.

(8) Basso, E. A.; Oliveira, P. R.; Wiectzycoski, F.; Pontes, R. M.; Fiorin, B. C. NMR and theoretical study of the (CO)–N rotational barrier in the isomers cis- and trans- 2-N,N-dimethylaminecyclohexyl 1-N′,N′-dimethylcarbamate. *Journal of Molecular Structure* **2005**, *753* (1), 139-146. DOI: https://doi.org/10.1016/j.molstruc.2005.06.001.

(9) Fritzemeier, R. G.; van der Westhuyzen, A. E.; D'Erasmo, M.; Sharma, S. K.; Bartsch, P.; Hodson, L. E.; Liu, K.; Wali, B.; Sayeed, I.; Liotta, D. C. Neurotherapeutic Potential of Water-Soluble pH-Responsive Prodrugs of EIDD-036 in Traumatic Brain Injury. *Journal of Medicinal Chemistry* **2023**, *66* (8), 5397-5414. DOI: 10.1021/acs.jmedchem.2c01484.

(10) Magaraci, F.; Jimenez; Rodrigues, C.; Rodrigues, J. C. F.; Braga, M. V.; Yardley, V.; de Luca-Fradley, K.; Croft, S. L.; de Souza, W.; Ruiz-Perez, L. M.; et al. Azasterols as Inhibitors of

Sterol 24-Methyltransferase in Leishmania Species and Trypanosoma cruzi. *Journal of Medicinal Chemistry* **2003**, *46* (22), 4714-4727. DOI: 10.1021/jm021114j.
